# Epidemiological Trends of Urolithiasis at the Global, Regional, and National Levels: A Population-Based Study

**DOI:** 10.1155/2022/6807203

**Published:** 2022-03-30

**Authors:** Xiaoyuan Qian, Junlai Wan, Jinzhou Xu, Chenqian Liu, Mingliang Zhong, Jiaqiao Zhang, Ying Zhang, Shaogang Wang

**Affiliations:** ^1^Department of Urology, Tongji Hospital, Tongji Medical College, Huazhong University of Science and Technology, Wuhan 430030, China; ^2^Department of Orthopedics, Tongji Hospital, Tongji Medical College, Huazhong University of Science and Technology, Wuhan 430030, China; ^3^Department of Nephrology, Tongji Hospital of Tongji Medical College, Huazhong University of Science and Technology, Wuhan 430030, China

## Abstract

**Background:**

Urolithiasis is common worldwide and can predispose to urinary tract infections and renal failure. We aimed to explore the global, regional, and national burden of urolithiasis between 1990 and 2019, stratified by sex, age, and sociodemographic index (SDI).

**Methods:**

From 1990 to 2019, data on the number of incident cases of urolithiasis, associated deaths, and disability-adjusted life years (DALYs) were extracted from the 2019 Global Burden of Disease (GBD) study. The trends for the incidence rate, mortality, and DALYs were evaluated using estimated annual percentage changes (EAPCs).

**Results:**

The incidence of urolithiasis increased by 48.57%, from 77.78 million incident cases in 1990 to 115.55 million in 2019, while its age-standardized incidence rate (ASIR) decreased. The ASIR increased slightly in the low SDI regions (EAPC = 0.33; 95% confidence interval [CI]: 0.24–0.43), while ASIRs in other SDI regions decreased. The incidence of urolithiasis by age presented a unimodal distribution, with the peak observed in patients aged between 50 years and 70 years. Urolithiasis-related mortality and DALYs also increased over time. Yet, the age-standardized death rate (ASDR) decreased by 2.05% (95% CI, −2.25% to −1.85%) per year, and the annual age-standardized DALY rate decreased by 1.77% (95% CI, −1.92% to −1.63%). The mortality and DALYs increased with age. The incidence, mortality, and DALYs were greater in males than those in females. The burden of urolithiasis showed obvious differences in its regional distribution over the past three decades.

**Conclusion:**

From 1990 to 2019, ASIR, ASDR, and age-standardized DALY rate of urolithiasis have decreased. Yet, particularly significant differences exist in the geographic, age, and sex distribution. Thus, medical resources should be rationally allocated and adjusted according to the geographic and demographic distribution of urolithiasis.

## 1. Introduction

Urolithiasis is highly prevalent in urology and can present as an asymptomatic condition or as a painful, recurrent disease. Affected by a variety of factors, such as the climate and seasonal temperature variation, dietary habits, water quality, direct occupational exposure, inheritance and genetic constitution, latitude, and comorbidities, the incidence and prevalence of urolithiasis are characterized by a distinct geographical variation [1–3]. The overall prevalence was 7.54% in mainland China between 1990 and 2016 [4], 5% to 10% in Europe in the decade before 2011, [5], 8.8% in North America between 2007 and 2010 [6], and 5.7% in Iran in 2005 [7].

The incidence and prevalence of urolithiasis have been on the rise. Although rarely life-threatening, urolithiasis can often cause intense pain and adversely affect patients' quality of life. Moreover, because of a large number of new and recurrent cases, the high rate of surgical intervention, and the advent of novel technology, global health care costs related to the management of stones are relatively high. In 2000, the treatment of urolithiasis did cost up to $5.3 billion per year in the United States alone [8]. Thus, further insight into the global urolithiasis burden is essential for the allocation of limited health resources and formulation of rational policies.

The global burden of disease (GBD) study examined the burden of hundreds of diseases and injuries in 195 countries and territories around the world and provided an opportunity to comprehensively assess different aspects of human health, including the distribution and development trends of urolithiasis [9]. For a better understanding of the trends in urolithiasis burden according to geography, age, sex, and social development index (SDI), we used data from the 2019 GBD to describe the global, regional, and national trends in the incident rate, deaths, and DALYs associated with urolithiasis from 1990 to 2019.

## 2. Methods

### 2.1. Study Data

Study data on the urolithiasis burden—the annual incidence rate, death, disability-adjusted life years (DALYs), and their age-standardized rates (ASR)—were extracted from the 2019 GBD study using the global health data exchange (GHDx) query tool (https://ghdx.healthdata.org/gbd-results-tool). We also collected data on sex, age, and SDI to investigate their influence on the urolithiasis disease burden. The SDI, which ranges from 0 to 1, is a comprehensive indicator of social and demographic development. According to the order of their SDI value, 192 countries and regions around the world are divided into low SDI, middle (low-middle, middle, high-middle) SDI, and high SDI countries. The epidemiological trends for urolithiasis were observed at the regional, national, and global levels.

### 2.2. Statistical Analysis

The trends for urolithiasis incidence and mortality rates were evaluated by calculating the annual age-standardized incidence rate (ASIR), the age-standardized death rate (ASDR), age-standardized DALYs rate, and their respective estimated annual percentage changes (EAPCs). Urolithiasis DALYs were computed as the sum of the years lived with disability and the years of life lost [10]. According to the age group construction of the standard population, the ASRs (per 100,000 population) were calculated using the following formula:

(1) $$\begin{array}{c} \text{ASR}=\frac{\sum_{i=1}^{A}a_{i}w_{i}}{\sum_{i=1}^{A}a_{i}}\times 100,000, \end{array}$$

(*a*_*i*_ refers to the incidence of the *i*^th^ age group. *w*_*i*_ denotes the number of persons (or weight) in the same age subgroup *i* of the assigned reference standard population) [11].

EAPCs is a generally well-accepted method to describe ASR using a regression model, and it quantitatively calculates the average annual rate of change of ASR for a specified period [11]. The regression line is used to estimate the natural logarithm of the rates, i.e., *y* = *α* + *βx* + *ɛ*, where *y* = ln (ASR), and *x* = the calendar year. The EAPC calculation formula, 100 × (exp(*β*) − 1), and its 95% confidence intervals (CI) can also be calculated from the linear regression model [11, 12]. All statistical data were analyzed using R version 3.6.3 (The R Foundation for Statistical Computing, Vienna, Austria), and a two-sided *P* < 0.05 was considered statistically significant.

## 3. Results

### 3.1. The Change in the Incidence of Urolithiasis

**Figure 1:**
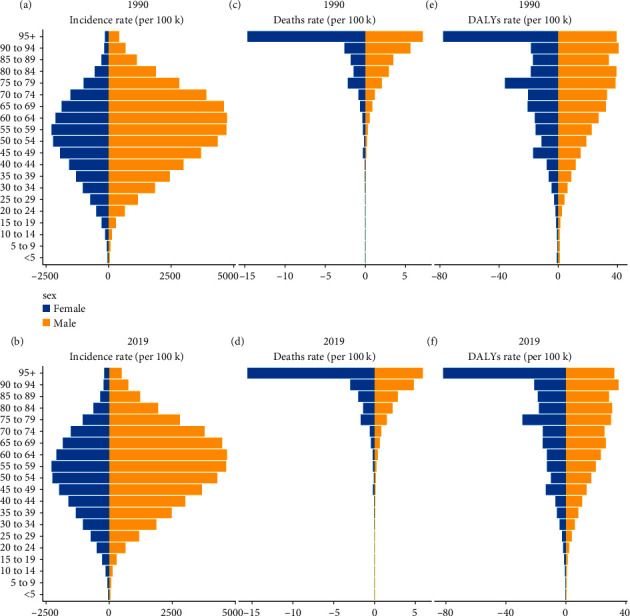
The incidence, death, and DALY rates of urolithiasis in different age groups. (a) Incidence in 1990. (b) Incidence in 2019. (c) Death rate in 1990. (d) Death rate in 2019. (e) DALY rate in 1990. (f) DALY rate in 2019.

**Figure 2:**
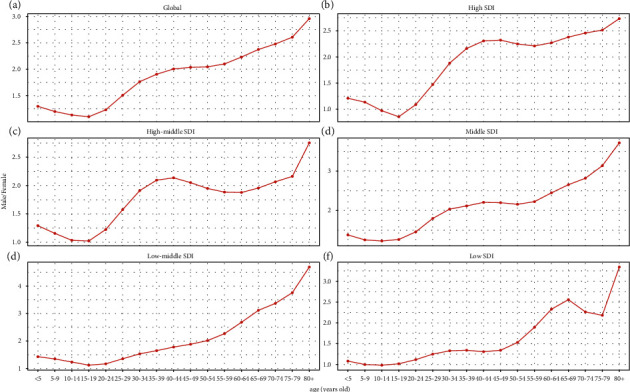
The ratio of male to female incidence in different age groups in 2019. (a) Global level. (b) High SDI regions. (c) High-middle SDI regions. (d) Middle SDI regions. (e) Middle-low SDI regions. (f) Low SDI regions. SDI: socio-demographic index.

Globally, the incidence of urolithiasis was 1.16 × 10^8^ (95% uncertainty interval (UI): 0.93–1.40 × 10^8^) in 2019 and 0.78 × 10^8^ (UI: 0.62–0.95 × 10^8^) in 1990. From 1990 to 2019, its incidence increased by 48.57% (Table 1, additional file 1: figure S1A). However, the global ASIR showed a decreasing trend, with an average annual decrease of 0.83% from 1696.18/100,000 persons (95% UI, 1358.11–2078.11) in 1990 to 1394.03/100,000 persons (95% UI: 1126.4–1688.16) in 2019 (EAPC = −0.83; 95% CI: −0.92 to −0.74) (Table 1, additional file 1: figure S1B). The annual ratio of elder (aged >60 years) to younger patients remained relatively stable each year (additional file1: figure S2A). Moreover, ASIR in both male and female patients decreased similarly, and ASIR in male patients was more than that in female patients (figures 1(a) and 1(b), additional file 1: figure S1B). Between 1990 and 2019, the incidence of urolithiasis plotted against age showed a unimodal distribution, with the peak value observed in patients aged between 50 years and 70 years (figures 1(a) and 1(b), additional file1: figures S3–S5). The male : female ratio tends to increase with age, increasing to approximately 3 in 2019 (Figure 2). However, urolithiasis tended to occur in the younger population (<60 years) (additional file1: figure S2A)

**Figure 3:**
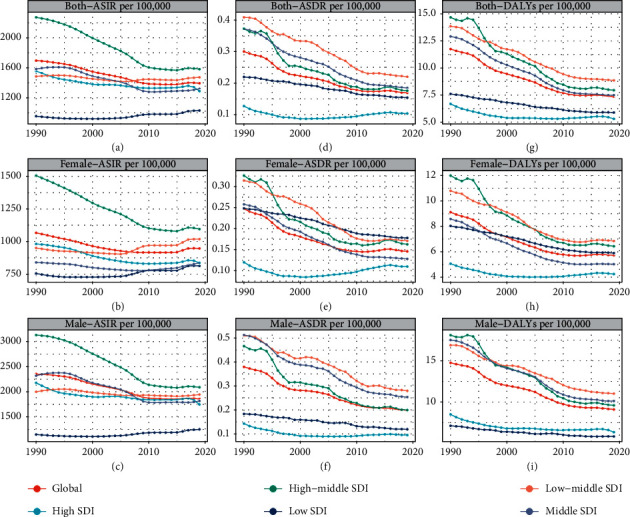
The change trends of age-standardized incidence (ASIR), age-standardized death (ASDR), and age-standardized incidence DALYs rate among different SDI countries. A–C: ASIR; D–F: ASDR; H–J: age-standardized DALYs rate.

**Figure 4:**
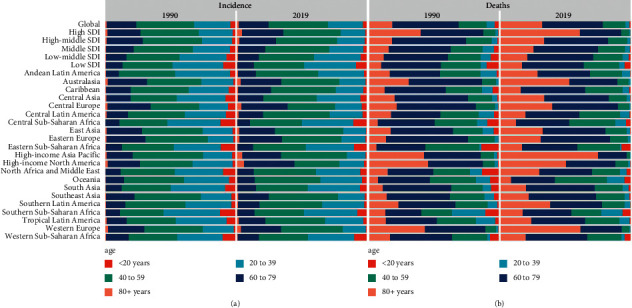
Distribution of different ages in urolithiasis incidence/death patients by region. (a) Incidence in 1990 and 2019. (b) Death rate in 1990 and 2019.

**Figure 5:**
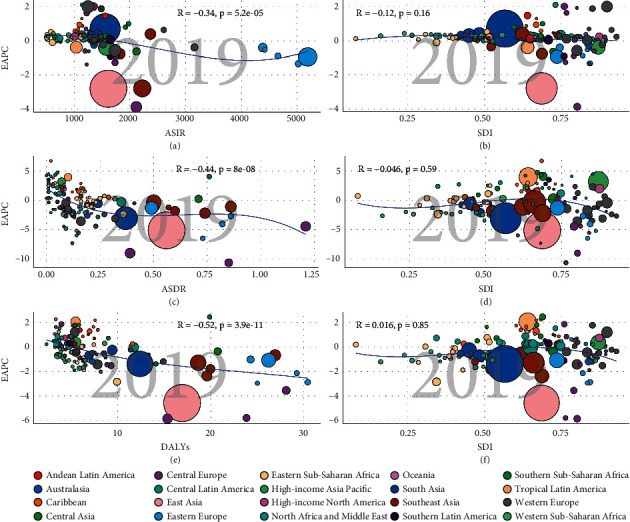
The correlation between EAPCs and urolithiasis ASR (incidence (a), death (b), and DALY (c)) in 2019 and HDI (incidence (d), death (e), and DALYs (f)) in 2019. The circles represent countries that are available on SDI values. The size of circles described the number of urolithiasis patients. The R indices Pearson's correlation coefficient and *p* values are obtained from Pearson's correlation analysis. ASR, age-standardized incidence/death/DALYs rate; EAPC, estimated annual percentage change; SDI, socio-demographic index.

With respect to the SDI level, the ASIR in the low SDI quintile presented a slightly increasing trend, with an EAPC of 0.33 (95% CI: 0.24–0.43). On the contrary, ASIRs in other quintiles decreased, with the decrease in the high-middle SDI quintile being the most significant (Table 1, figures 3(a)–3(c)). The higher the SDI level, the more the elderly patients among all urolithiasis incidence patients (figure 4(a)). There were similar trends of male : female ratio in all SDI regions (Figure 2). Meanwhile, a significant positive correlation was detected between ASIR and SDI (*R* = 0.46, *P* < 0.05) (additional file1: figure S6A). Furthermore, EAPC was negatively correlated with ASIR (*R* = −0.34, *P* < 0.05), implying that urolithiasis increased more slowly in countries with high incidence than in countries with low incidence (Figure 5(a)).

**Figure 6:**
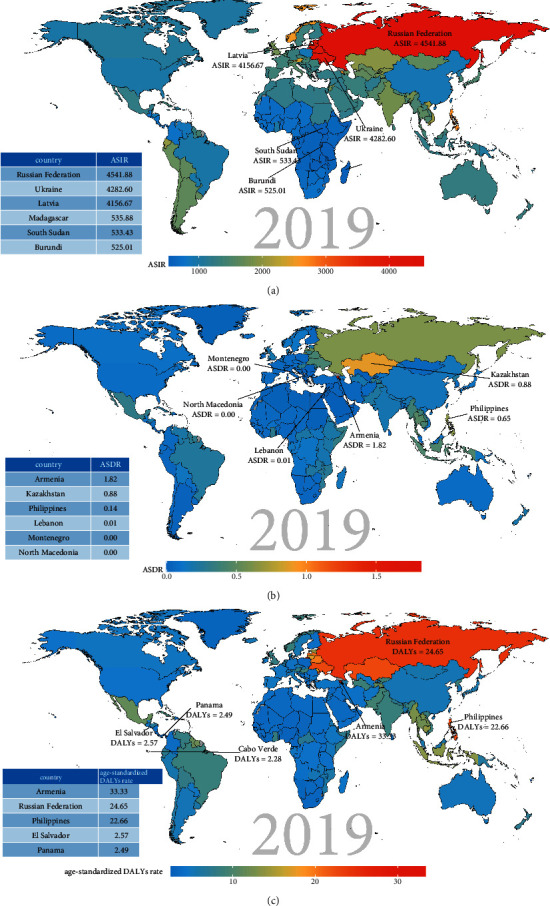
The global disease burden of urolithiasis for both sexes in 192 countries. (a) The ASIR of urolithiasis in 2019. (b) The ASDR of urolithiasis in 2019. (c) The age-standardized DALY rate of urolithiasis in 2019. ASIR, age-standardized incidence rate; ASDR, age-standardized death rate.

As for the findings by the GBD regions and countries, the ASIR of urolithiasis increased in 143 countries and 11 regions (the top three countries: Jordan, Romania, and Germany), decreased in 32 countries and 9 regions (the bottom three countries: Poland, China, and Indonesia), and remained stable in 17 countries and 1 region (Albania, Costa Rica et al.). The top three countries with high ASIRs were the Russian Federation (4541.88 per 100,000 people), Ukraine (4282.60 per 100,000 people), and Latvia (4156.67 per 100,000 people). The bottom three countries were Burundi (525.01 per 100,000 people), South Sudan (533.43 per 100,000 people), and Madagascar (535.88 per 100,000 people). The top three countries with EAPC were Jordan (2.10), Romania (2.01), and Germany (2.00), while the bottom three countries were Poland (−3.87), China (−2.8), and Indonesia (−2.17). All the above results are shown in figure 6(a) and additional file 1: tables S1–S3, figure S7A. In addition, the majority of countries had a unimodal age distribution (additional file 1: table S4).

### 3.2. The Change in the Mortality of Urolithiasis

Globally, mortality because of urolithiasis increased by 17.12% from 113.38 × 10^2^ (95%UI: 72.78–137.77) in 1990 to 132.27 × 10^2^ (95% UI: 106.16–162.67) in 2019 (Table 2, additional file 1: figure S1C). Over the past three decades, ASDR decreased significantly with an average annual decrease of 2.05% from 0.3/100,000 persons (95% UI, 0.20–0.37) in 1990 to 0.17/100,000 persons (95% UI: 0.14–0.21) in 2019 (EAPC = −2.05; 95% CI: −2.25 – −1.85) (Table 2). The death cases increased rapidly with age, especially for the elderly (figure 1(b); additional file 1: figures S8–S10). ASDR in both male and female patients decreased significantly, and the ASDR of male patients was higher than that of female patients between 1990 and 2019 (Table 2, figure 1(b), additional file 1: figure S1C). The mortality ratio of male and female patients fluctuated with age (additional file 1: figure S11).

With respect to the SDI, ASDRs in the different SDI regions decreased (Table 2, figure 3(b)). EAPC was negatively associated with ASDR (*R* = −0.44, *P* < 0.05). However, a correlation between SDI and EAPC or ASDR was not revealed (figure 5(b), additional file 1: figure S6B). Contrary to the change in ASIR, older patients (>60 years) died from urolithiasis (figure 4(b), additional file1: figure S1A). The number of annual young deaths decreased year by year, however, the number of elderly deaths increased gradually, irrespective of SDI regions, especially in patients >95 years of age (additional file 1: figures S1C & S8–S10).

At the level of GBD regions and countries, 69 countries and 7 regions had an increased ASDR, 104 countries and 11 regions had a reduced ASDR, and 19 countries and 3 regions had a stable ASDR. The top three ASDRs were those of Armenia, Kazakhstan, and the Philippines, while the bottom three were those of North Macedonia, Montenegro, and Lebanon. Details are described in figure 6(b), additional file 1: tables S1, S2, & S5, figure S7B. The death cases increased rapidly with age irrespective of sex in the majority of countries (additional file 1: table S7).

### 3.3. The Change in DALYs of Urolithiasis

On a global level, there were 5,167,310 (95% UI, 3,741,330 to 6,357,170) DALYs in 1990 and 6,043,090 (95% UI, 4,773,540 to 7,451,940) DALYs in 2019, an increase of 16.95%. The age-standardized DALYs rate demonstrated a downward trend with an EAPC of −1.77 (95% CI, −1.92 to −2.21), declining from 11.75/100,000 persons (95% UI, 8.57–14.39) in 1990 to 7.35/100,000 persons (95% UI, 5.82–9.04) in 2019 (Table 3). The age-standardized DALYs rate of males was higher than that of female cases between 1990 and 2019 (Table 3). Investigating from the SDI standpoint, all four SDI regions witnessed a drop in the age-standardized DALYs rate, and DALYs were not associated with SDI (Table 3, additional file 1: figure S6C). Besides, there was no significant correlation between EAPC and SDI (*R* = −0.016, *p* = 0.85), and there was a negative association between EAPC and the age-standardized DALYs rate (figure 5(c), R = −0.52, *p* < 0.01). The DALYs rate rose in both male and female patients with age (figure 1(c)). Compared with 1990, there was no obvious decrease in DALYs in elderly patients in 2019, and similar trends in the age distribution of mortality were seen in DALYs (additional file 1: figures S12–S14). The DALY ratio of male and female patients also fluctuated with age (additional file 1: figure S15).

At the level of GBD regions and countries, 73 countries and 6 regions had an increased age-standardized DALYs rate, 104 countries and 11 regions had a decreased age-standardized DALYs rate, and 15 countries and 4 regions had a stable age-standardized DALYs rate. The top three age-standardized DALYs rates were seen in Armenia, Russian Federation, and the Philippines. The bottom three age-standardized DALYs rates were seen in Cabo Verde, Panama, and El Salvador. Details are displayed in figure 6(c), additional file 1: tables S1, S2, & S6, and additional file 1: figure S7C. Similarly, The DALYs increased rapidly with age, irrespective of sex in almost every country (additional file 1: table S8).

## 4. Discussion

Currently, urolithiasis remains a major global public health problem and warrants our attention. Several previous epidemiological studies on urolithiasis have mainly focused on individual regions or countries [5, 13, 14], however, global epidemiological data on urolithiasis are lacking. Based on the 2019 GBD study, we described the incidence, mortality, and DALY of urolithiasis at the global, regional, and national levels with their corresponding current trends and survival patterns from 1990 to 2019.

Our findings demonstrated that though the global incidence of urolithiasis increased by 48.57% in 2019 compared with its incidence in 1990, the ASIR actually decreased. There are several explanations for this trend. Zhu et al. reported that the increase in incidence might be attributed to population growth and the aging process of the population [15]. Advances in the detection of both symptomatic and asymptomatic stones by modern imaging methods and the widespread use of CT scans also contributed to the increased incidence of kidney stones in the study by Kittanamongkolchai et al. [16].

We found that urolithiasis incidence was associated with age distribution, which has not changed markedly over the last 30 years, and that the 40 to 60 years age group in both adult males and females was more likely to suffer from urolithiasis. This result is consistent with that of other scholars [8, 17]. The shift to middle age-preponderance of urolithiasis can be attributed to multiple factors, such as changing work, diets, and lifestyles [5, 18, 19]. For example, to prevent or treat osteoporosis, women aged between 50 years and 79 years often consume calcium and vitamin D supplements, which can cause hypercalciuria. Consequently, this population may have a high incidence of urolithiasis [20]. Consistent with previous studies [3, 19, 21, 22], male predominance in the incidence of urolithiasis was also revealed in our study. From 1990 to 2019, the male : female ratio of urolithiasis incidence has gradually increased to approximately 3. This gender disparity may be associated with changes in the diet and an increase in metabolic syndromes, such as diabetes and obesity [3].

EAPC was found to be negatively associated with ASIR, implying that urolithiasis increased more slowly in countries with high incidence than in countries with low incidence. The ASIR also differed between SDI quintiles. ASIR was higher in high SDI, middle-high SDI, middle SDI, and low-middle regions than in low SDI regions. Except in low regions, ASIR had a decreasing global pattern in other quintiles. Interestingly, ASIR of the Russian Federation and its neighboring countries, such as Ukraine and Latvia, was substantially higher than that in others. Meanwhile, African countries—Madagascar, South Sudan, and Burundi—had a lower ASIR. This phenomenon is likely to be because of an increase in meat consumption and the prevalence of diabetes and obesity, all of which are risk factors for urolithiasis [23]. Another study pointed out that the diversity and greater numbers of oxalate-degrading bacteria existing in the gastrointestinal tract played a crucial role in preventing urolithiasis in Black South Africans [24].

Although mortality and DALY in both males and females increased in 2019, the ASDR and age-standardized DALY rate decreased year by year. This decreasing pattern in the ASDR and age-standardized DALY rate of urolithiasis over the last three decades is intertwined with surgical innovations and better treatment guidelines [25]. Surgical treatment might not be the first choice, especially for stones in the inferior pole of the kidney, and pharmacological treatment can be beneficial for spontaneous stone passage. Moreover, it is technological advances that have made it possible to opt for less invasive surgical interventions that are safe, effective, and associated with shorter recovery times and lesser discomfort [26].

The proportion of the elderly in both ASDR and age-standardized DALYs rate of urolithiasis increased. It may be because of their poor tolerance to severe infections and susceptibility to complications, including decreased bone density, cardiovascular disease, and chronic kidney disease [27]. Similarly, there was a demonstrable negative relationship among EAPC, the change in ASDR and age-standardized DALYs rate from 1990 to 2019, and their baseline value in 1990. The higher the ASDR and age-standardized DALYs rate in 1990, the more they changed. This finding may be explained by the fact that countries with higher ASDR and age-standardized DALYs rates were more likely to prioritize urolithiasis prevention programs because of public health concerns. However, in our study, the SDI level did not have an impact on ASDR and age-standardized DALYs rate, which may indicate that better healthcare is less important for a decrease in ASDR and age-standardized DALYs rate. Moreover, as seen in a prior study, we also found a significant regional distribution [15]. For instance, ASDR and age-standardized DALYs rates were very high in Eastern Europe, Central Asia, and Southeast Asia, while extremely low in North Africa, the Middle East, Southern Latin America, and Southern Sub-Saharan Africa.

Despite the GBD study providing high-quality estimates of the global urolithiasis burden, several limitations are inevitable. Firstly, limited data can be obtained from lower SDI countries, and the data we extracted is not representative. Therefore, the data only reveals the general situation of a population in specific regions and countries. Secondly, different countries and regions have different diagnoses, screening standards, and monitoring systems for urolithiasis. Thus, there may be differences in the quality of data. Thirdly, because of the lack of data on risk factors, risk factors for urolithiasis were not assessed.

## 5. Conclusions

Over the last 30 years, ASIR, ASDR, and age-standardized DALYs rates of urolithiasis have decreased. Yet, significant differences in the geographic, age, and sex distributions were observed. Thus, based on the current epidemiological characteristics of urolithiasis, medical resources should be rationally allocated or adjusted.

## Figures and Tables

**Table 1:** The incident cases and ASIR in 1990 and 2019 and its current trends from 1990 to 2019.

	1990		2019		1990–2019
	Incident cases no. *∗*102 (95% UI)	ASIR per 100,000 no. (95% UI)	Incident cases no. *∗*102 (95% UI)	ASIR per 100,000 no. (95% UI)	EAPC no. (95% CI)
Overall	777,757.57 [622,391.16–951,267.51]	1696.18 [1358.11–2078.11]	1,155,521.4 [930,451.3–1,401,804.02]	1394.03 [1126.4–1688.16]	−0.83 [−0.92 to −0.74]
Sex					
Female	249,942.36 [199,717.79–305,779.86]	1066.85 [851.17–1305.09]	394,466.96 [316,443.64–479,042.56]	947.22 [761.21–1148.43]	−0.83 [−0.92 to −0.74]
Male	527,815.22 [421,541.28–644,728.87]	2353.15 [1878.96–2879.17]	761,054.44 [610,284.55–921,683.98]	1856.87 [1495.27–2245.34]	−0.83 [−0.92 to −0.74]
Sociodemographic index					
High SDI	145,954.54 [115,092.47–180,095.64]	1556.68 [1228.01–1924.36]	175,249.86 [143,148.65–211,860.66]	1288.65 [1053.86–1544.09]	−0.47 [−0.56 to −0.38]
High-middle SDI	261,665.25 [209,262.28–320,011.49]	2273.31 [1819.79–2776.75]	295,371.67 [234,109.15–360,701.08]	1582.15 [1273.68–1924.87]	−1.52 [−1.66 to −1.39]
Middle SDI	211,170.93 [167,561.15–259,150.52]	1582.66 [1255.22–1938.68]	356,833.63 [286,464.56–435,292.92]	1319.12 [1062.99–1600.96]	−0.96 [v1.09 to −0.84]
Low-middle SDI	124,476.95 [99,936.92–152,749.55]	1485.55 [1193.43–1813.78]	244,903.47 [195,173.97–300,902.16]	1473.13 [1171.09–1804.8]	−0.12 [−0.18 to −0.05]
Low SDI	34,193.36 [26,981.21–42,070.53]	954.92 [755.16–1176.74]	82,575.81 [64952.83–102,069.74]	1030.45 [809.77–1273.81]	0.33 [0.24 to 0.43]
Region					
Andean Latin America	4592.35 [3666.61–5711.27]	1609.5 [1290.27–1977.8]	11,073.03 [9169.33–13,239.26]	1772.43 [1472.6–2110.69]	0.52 [0.44 to 0.6]
Australasia	3140.31 [2465.53–3872.12]	1405.3 [1096.4–1739.12]	4769.7 [3737.57–5898.03]	1283.37 [1004.68–1573.74]	−0.35 [−0.44 to −0.26]
Caribbean	3141.27 [2478.56–3868.37]	1056.51 [830.22–1310.52]	6319.37 [4959.54–7881.91]	1239.73 [979.28–1540.36]	0.66 [0.63 to 0.7]
Central Asia	10,146.28 [8051.77–12,407.22]	1755.54 [1403.55–2151.02]	16,552.56 [13,150.52–20,320.44]	1787.98 [1435.54–2174.91]	0.05 [0.02 to 0.08]
Central Europe	23,202.84 [18,302.32–28,555.11]	1657.22 [1324.62–2032.82]	17,735.73 [14,611.9–21,439.47]	1178.91 [977.08–1400.97]	−0.71 [−1 to −0.42]
Central Latin America	11,169.98 [8874.72–13,714.68]	974.87 [774.06–1202.2]	25,837.65 [20,622.08–31,272.76]	1012.43 [810.44–1222.61]	0.13 [−0.21 to 0.48]
Central sub-Saharan Africa	1990.39 [1562.96–2465.6]	533.21 [417.75–663.53]	5315.77 [4130.91–6572.04]	575.37 [446.63–711.22]	0.31 [0.24 to 0.37]
East Asia	168,619.46 [132,241.51–208,877.18]	1592.83 [1245.33–1984.66]	185,313.78 [147,859.26–226,692.4]	901.81 [727.27–1088.77]	−2.68 [−2.96 to −2.4]
Eastern Europe	138,768.69 [112,392.18–168,155.88]	5143.77 [4155.8–6201.33]	127,338.57 [102,014.28–156,010.91]	4433.72 [3542.49–5414.66]	−0.69 [−0.85 to −0.53]
Eastern sub-Saharan Africa	6479.93 [5146.44–7948.33]	548.62 [431.96–674.35]	15432.53 [12133.22–19039.5]	565.68 [444.29–692.31]	0.15 [0.09 to 0.2]
High-income Asia Pacific	31,236.93 [23,822.16–39,297.37]	1536.37 [1181.32–1920.86]	39,470.15 [31,610.91–48,358.91]	1475.15 [1172.93–1795.88]	−0.27 [−0.35 to −0.18]
High-income North America	50,604.28 [39,648.82–62,952.81]	1621.04 [1270.22–2010.69]	47,402.27 [40,694.01–55,617.51]	982.95 [843.8–1137.38]	−2.02 [−2.34 to −1.69]
North Africa and middle East	29,136.66 [22,817.49–36,153.48]	1159.44 [904.44–1445.58]	74,482.11 [58,347.67–93,542.37]	1250.71 [985.87–1553.28]	0.29 [0.26 to 0.32]
Oceania	441.17 [343.55–553.77]	978.39 [758.56–1220.19]	1096.74 [847.74–1389.26]	1033.1 [799.54–1296.18]	0.14 [0.09 to 0.19]
South Asia	130,374.21 [102,728.35–162,126]	1518.02 [1206.38–1880.74]	307,303.25 [241,418.47–382,421.15]	1757.72 [1382.75–2184.63]	0.64 [0.54 to 0.74]
Southeast Asia	63,038.83 [50,129.6–76,385.12]	1904.34 [1511.83–2313.01]	118,032.07 [95,434.17–142,760.02]	1652.63 [1348.2–1979.06]	−0.82 [−0.95 to −0.69]
Southern Latin America	7819.94 [6065.58–9817.15]	1646.58 [1275.16–2070.5]	12394.17 [9577.27–15596.51]	1674.47 [1295.15–2119.25]	−0.21 [−0.28 to −0.14]
Southern sub-Saharan Africa	2887.71 [2272.83–3570.66]	701.37 [552.78–863.37]	5461.43 [4302.99–6791.1]	725.49 [574.19–893.26]	0.11 [0.07 to 0.15]
Tropical Latin America	12,924 [10,385.26–15,774.68]	1034.66 [833.81–1253.92]	24,369.28 [19.765.47–29,260.19]	969.93 [789.43–1165.79]	-0.36 [-0.45 to −0.28]
Western Europe	69,354.2 [54,788.18–85,768.48]	1490.85 [1183–1846.74]	88,055.46 [70,368.82–108,735.24]	1490.21 [1181.37–1829.24]	0.53 [0.38 to 0.69]
Western sub-Saharan Africa	8688.15 [6911.46–10654.41]	688.96 [543.93–847.52]	21,765.77 [17,278.95–26,674.36]	735.82 [579.31–902.54]	0.28 [0.22 to 0.34]

ASIR: age-standardized incidence rate; EAPC: estimated annual percentage change; CI: confidence interval; UI: uncertainty interval.

**Table 2:** The death cases and ASDR in 1990 and 2019 and its up-to-date trends from 1990 to 2019.

	1990		2019		1990–2019
	Deaths cases no. *∗*10^2^ (95% UI)	ASDR per 100,000 No. (95% UI)	Deaths cases no. *∗*10^2^ (95% UI)	ASDR per 100,000 no. (95% UI)	EAPC no. (95% CI)
Overall	113.38 [72.78–137.77]	0.3 [0.2–0.37]	132.79 [106.16–162.67]	0.17 [0.14–0.21]	−2.05 [−2.25 to −1.85]
Sex					
Female	51.84 [35.12–61.16]	0.25 [0.17–0.29]	63.48 [51.75–82.92]	0.15 [0.12–0.19]	−2.05 [−2.25 to −1.85]
Male	61.54 [30.77–80.34]	0.38 [0.2–0.5]	69.31 [44.97–91.42]	0.2 [0.13–0.26]	−2.05 [−2.25 to −1.85]
Sociodemographic index					
High SDI	13.33 [11.03–16.31]	0.13 [0.1–0.16]	22.41 [17.67–30.28]	0.1 [0.08–0.14]	−0.09 [−0.53 to 0.34]
High-middle SDI	37.9 [29.85–43.71]	0.37 [0.29–0.43]	35.05 [30.24–43.39]	0.18 [0.15–0.22]	−2.76 [−3.08 to −2.44]
Middle SDI	34.58 [16.81–44.28]	0.37 [0.18–0.48]	41.14 [28.21–52.7]	0.18 [0.13–0.24]	−2.61 [−2.74 to −2.47]
Low-middle SDI	22.93 [8.3–33.21]	0.41 [0.15–0.62]	27.55 [13.96–36.62]	0.22 [0.11–0.29]	−2.38 [−2.52 to −2.24]
Low SDI	4.59 [2.68–6.9]	0.22 [0.12–0.36]	6.57 [4.16–9.61]	0.15 [0.1–0.23]	−1.35 [−1.4 to −1.29]
Region					
Andean Latin America	0.15 [0.07–0.21]	0.07 [0.04–0.1]	0.36 [0.17–0.5]	0.06 [0.03–0.09]	0.16 [−0.05 to 0.38]
Australasia	0.47 [0.37–0.56]	0.2 [0.16–0.24]	0.55 [0.44–0.74]	0.1 [0.08–0.14]	−2.15 [−2.74 to −1.56]
Caribbean	0.35 [0.27–0.44]	0.13 [0.1–0.17]	0.92 [0.68–1.23]	0.18 [0.13–0.24]	1.6 [1.43 to 1.77]
Central Asia	1.2 [0.84–1.85]	0.27 [0.19–0.42]	2.43 [1.87–3.56]	0.44 [0.32–0.69]	1.74 [1.38 to 2.11]
Central Europe	5.94 [5.14–8.83]	0.42 [0.36–0.62]	1.44 [1.14–1.85]	0.07 [0.05–0.08]	−6.31 [−7.33 to −5.27]
Central Latin America	1.85 [1.49–2.17]	0.22 [0.17–0.26]	4.91 [3.94–6.88]	0.21 [0.17–0.29]	−0.14 [−0.38 to 0.09]
Central sub-Saharan Africa	0.31 [0.15–0.54]	0.14 [0.06–0.23]	0.56 [0.29–0.95]	0.11 [0.05–0.19]	−0.78 [−0.92 to −0.63]
East Asia	40.73 [18.5–51.36]	0.55 [0.25–0.69]	26.96 [18.32–36.57]	0.15 [0.1–0.2]	−4.93 [−5.23 to −4.63]
Eastern Europe	17.99 [15.39–23.08]	0.65 [0.56–0.83]	18.93 [15.49–22.84]	0.55 [0.45–0.66]	−0.84 [−1.3 to −0.37]
Eastern sub-Saharan Africa	1.4 [0.82–2.44]	0.23 [0.13–0.37]	2.3 [1.32–4.12]	0.2 [0.12–0.34]	−0.54 [−0.58 to −0.5]
High-income Asia Pacific	1.23 [0.92–1.52]	0.07 [0.05–0.09]	7.05 [5.04–9.64]	0.11 [0.09–0.15]	2.6 [2.32 to 2.88]
High-income North America	3.35 [2.65–4.14]	0.09 [0.07–0.11]	6.24 [5.12–8.49]	0.09 [0.08–0.13]	0.8 [0.43 to 1.18]
North Africa and middle East	0.48 [0.28–0.81]	0.03 [0.01–0.06]	1.16 [0.7–1.51]	0.03 [0.02–0.04]	1.06 [0.78 to 1.35]
Oceania	0.05 [0.02–0.08]	0.17 [0.06–0.28]	0.09 [0.03–0.15]	0.12 [0.05–0.2]	−1.22 [−1.32 to −1.12]
South Asia	16.18 [7.39–25.81]	0.33 [0.15–0.56]	20.82 [12.24–30.82]	0.16 [0.1–0.25]	−2.65 [−2.76 to −2.54]
Southeast Asia	12.07 [3.2–17.8]	0.5 [0.15–0.74]	21.85 [6.95–29.47]	0.4 [0.13–0.55]	−0.88 [−1.01 to −0.74]
Southern Latin America	0.12 [0.1–0.16]	0.03 [0.02–0.04]	0.34 [0.26–0.44]	0.04 [0.03–0.05]	1.13 [0.72 to 1.54]
Southern sub-Saharan Africa	0.17 [0.11–0.23]	0.05 [0.04–0.07]	0.29 [0.2–0.39]	0.05 [0.04–0.07]	−0.17 [−0.86 to 0.52]
Tropical Latin America	0.88 [0.67–1.06]	0.1 [0.07–0.12]	5.52 [4.33–9.31]	0.23 [0.18–0.39]	4 [3.75 to 4.25]
Western Europe	7.68 [5.71–8.68]	0.13 [0.1–0.15]	8.84 [7.29–12.37]	0.08 [0.07–0.12]	−1.11 [−1.57 to −0.66]
Western sub-Saharan Africa	0.79 [0.3–1.32]	0.11 [0.04–0.19]	1.24 [0.53–1.75]	0.08 [0.03–0.11]	−1.29 [−1.39 to −1.2]

ASDR: age-standardized death rate; EAPC: estimated annual percentage change; CI: confidence interval; UI: uncertainty interval.

**Table 3:** The DALYs and age-standardized DALYs rate in 1990 and 2019 and its up-to-date trends from 1990 to 2019.

	1990		2019		1990–2019
	DALY no. *∗* 10^3^ (95% UI)	Age-standardized DALY rate per 100,000 no. (95% UI)	DALY no. *∗* 10^3^ (95% UI)	Age-standardized DALY rate per 100,000 no. (95% UI)	EAPC no. (95% CI)
Overall	5167.31 [3741.33–6357.17]	11.75 [8.57–14.39]	6043.09 [4773.54–7451.94]	7.35 [5.82–9.04]	−1.77 [−1.92 to −1.63]
Sex					
Female	2081.58 [1524.74–2490.71]	9.11 [6.73–10.91]	2420.08 [1939.13–2990.58]	5.72 [4.58–7.07]	−1.77 [−1.92 to −1.63]
Male	3085.73 [2071.94–3928.78]	14.76 [9.92–18.82]	3623.01 [2744.39–4545.58]	9.1 [6.92–11.34]	−1.77 [−1.92 to −1.63]
Sociodemographic index					
High SDI	646.46 [496.31–818.12]	6.68 [5.07–8.53]	802.64 [626.22–1005.97]	5.3 [3.99–6.75]	−0.5 [−0.69 to −0.31]
High-middle SDI	1639.97 [1331.4–1970.73]	14.68 [11.99–17.61]	1525.21 [1218.89–1871.38]	7.94 [6.33–9.79]	−2.41 [−2.62 to −2.21]
Middle SDI	1587.67 [1017.33–1981.05]	12.91 [8.23–16.13]	1943.09 [1505–2400.21]	7.49 [5.81–9.17]	−2.12 [−2.26 to −1.98]
Low-middle SDI	1047.82 [568.37–1355.54]	13.86 [7.44–18.79]	1359.64 [975.4–1738.97]	8.85 [6.24–11.25]	−1.74 [−1.83 to −1.64]
Low SDI	243.48 [169.25–332.3]	7.6 [5.21–10.43]	409.03 [295.81–547.9]	5.88 [4.33–7.87]	−0.96 [−1.01 to −0.9]
Region					
Andean Latin America	17.25 [11.89–23.21]	6.32 [4.46–8.49]	39.33 [28.3–52.39]	6.4 [4.62–8.44]	0.28 [0.19 to 0.37]
Australasia	17.77 [14.28–21.92]	7.78 [6.25–9.65]	21.81 [16.67–28.01]	5.37 [4.04–6.95]	−1.13 [−1.42 to −0.83]
Caribbean	19.29 [14.76–24.78]	6.61 [5.13–8.3]	41.58 [32.06–53.23]	8.14 [6.26–10.45]	1.08 [0.98 to 1.18]
Central Asia	57.19 [42.34–76.35]	10.83 [8.05–14.57]	95.98 [75.68–120.04]	12.42 [9.88–15.89]	0.29 [0.12 to 0.47]
Central Europe	193.89 [159.39–263.24]	13.51 [11.06–18.26]	74.32 [56.83–96.06]	4.52 [3.4–5.95]	−3.18 [−3.6 to −2.76]
Central Latin America	88.28 [74.03–105.22]	8.21 [6.86–9.78]	205.88 [167.4–263.13]	8.24 [6.7–10.57]	0.06 [−0.2 to 0.32]
Central sub-Saharan Africa	16.32 [10.42–26.29]	5.02 [3.11–7.83]	32.89 [21.94–47.33]	4.33 [2.77–6.34]	−0.54 [−0.64 to −0.45]
East Asia	1621.28 [961.22–2002.29]	16.61 [9.81–20.36]	1075.6 [841.94–1343.94]	5.35 [4.17–6.69]	−4.43 [−4.69 to −4.16]
Eastern Europe	809.3 [661.25–979.1]	29.44 [24.01–35.53]	729.69 [586.18–893.37]	23.61 [18.69–29.23]	−1.09 [−1.36 to −0.82]
Eastern sub-Saharan Africa	67.41 [44.29–111.06]	6.37 [4.24–10.05]	106.78 [69.37–162.09]	4.83 [3.18–7.5]	−1.09 [−1.16 to −1.03]
High-income Asia Pacific	109.59 [77.09–148.23]	5.48 [3.91–7.35]	188.89 [145.15–237.34]	5.7 [4.18–7.45]	0.25 [0.16 to 0.34]
High-income North America	197.48 [147.12–256.27]	6.16 [4.55–8.08]	234.06 [187.71–290.06]	4.52 [3.59–5.63]	−1.01 [−1.29 to −0.72]
North Africa and middle East	98.26 [67.05–135.2]	3.95 [2.73–5.46]	235.59 [159.04–328.15]	4.1 [2.82–5.65]	0.2 [0.16 to 0.25]
Oceania	3.03 [1.69–4.27]	7.31 [3.99–10.28]	6.2 [3.82–8.72]	6.25 [3.86–8.8]	−0.58 [−0.63 to −0.52]
South Asia	842.78 [536.3–1134.41]	11.47 [7.08–16.51]	1355.04 [979.19–1806.73]	8.33 [6–11.1]	−1.2 [−1.31 to −1.1]
Southeast Asia	530.34 [243.98–712.9]	17.17 [7.93–23.59]	857.45 [460.68–1090.19]	13.06 [6.92–16.54]	−1.15 [−1.25 to −1.06]
Southern Latin America	24.78 [16.71–34.52]	5.23 [3.53–7.3]	41.49 [28.43–57.6]	5.54 [3.78–7.74]	−0.07 [−0.17 to 0.02]
Southern sub-Saharan Africa	15.18 [11.11–19.23]	3.71 [2.85–4.68]	25.02 [18.71–32.63]	3.46 [2.63–4.44]	−0.22 [−0.6 to 0.15]
Tropical Latin America	64.63 [51.08–80.92]	5.43 [4.34–6.76]	211.87 [169.2–298.86]	8.55 [6.84–12.13]	2.07 [1.93 to 2.2]
Western Europe	326.16 [254–411.6]	6.55 [5.04–8.41]	368.39 [275.06–474.37]	5.55 [3.99–7.28]	−0.03 [−0.21 to 0.14]
Western sub-Saharan Africa	47.11 [29.76–65.05]	4.12 [2.55–5.79]	95.22 [65.48–126.5]	3.61 [2.42–4.79]	−0.45 [−0.49 to −0.42]

DALYs: disability adjusted life-years; EAPC: estimated annual percentage change; CI: confidence interval; UI: uncertainty interval.

## Data Availability

The data used in this study is available from the global health data exchange query tool (https://ghdx.healthdata.org/gbd-results-tool).

## Abbreviations

ASR: Age-standardized rate
ASIR: Age-standardized incidence rate
ASDR: Age-standardized death rate
EAPC: Estimated annual percentage changes
CI: Confidence interval
DALYs: Disability-adjusted life years
GBD: Global burden of disease study
SDI: Sociodemographic index
UI: Uncertainty interval
YLD: Years lived with disability
YLL: Years of life lost.

## Supplementary Materials

Supplementary Materials The supplementary material file 1: the supplementary tables and figures are already cited in our manuscript “Epidemiological trends of urolithiasis at the global, regional, and national levels: a population-based study.” The supplementary material file 2: figures are already cited in the manuscript “Epidemiological trends of urolithiasis at the global, regional, and national levels: a population-based study.”
