# Supplementary material for: Epidemiological Trends of Urolithiasis at the Global, Regional, and National Levels: A Population-Based Study
Source: Int J Clin Pract. 2022 Mar 30;2022:6807203. doi: 10.1155/2022/6807203 (PMC9159214; doi:10.1155/2022/6807203)
Supplement: Supplementary Materials — The supplementary material file 1: the supplementary tables and figures are already cited in our manuscript “Epidemiological trends of urolithiasis at the global, regional, and national levels: a population-based study.” The supplementary material file 2: figures are already cited in the manuscript “Epidemiological trends of urolithiasis at the global, regional, and national levels: a population-based study.” [file 6807203.f1.zip › 6807203.f1/The supplementary material file 1.pdf]

ORIGINAL RESEARCH

Xiaoyuan Qian et al

Epidemiological trends of urolithiasis at the global, regional, and national levels: a population-based study

Author names: Xiaoyuan Qian[1†], Junlai Wan[2†], Jinzhou Xu[1], Chenqian Liu[1], Mingliang Zhong[1], Jiaqiao Zhang[1], Ying Zhang[3*], Shaogang Wang[1*]

[1]Author affiliation

Department of Urology, Tongji Hospital, Tongji Medical College, Huazhong University of Science and Technology, Wuhan 430030, China;

[2]Author Affiliation

Department of Orthopedics, Tongji Hospital, Tongji Medical College, Huazhong University of Science and Technology, Wuhan 430030, China

[3]Author affiliation

Department of Nephrology, Tongji Hospital of Tongji Medical College, Huazhong University of Science and Technology, Wuhan 430030, China

Supplementary Tables

Supplementary Table S1 Three countries with the largest and lowest number of incidence, death, or DALYs.

Supplementary Table S2. Three regions with the largest and lowest number of incidence, death, or DALYs.

Supplementary Table S3. The incident cases and age-standardized incidence rate of Urolithiasis in 1990 and 2019, and its temporal trends from 1990 to 2019.

Supplementary Table S4. Age distribution of incidence (per 100,000) for Urolithiasis in different countries in 2019.

Supplementary Table S5. The death cases and age-standardized death rate of Urolithiasis in 1990 and 2019, and its temporal trends from 1990 to 2019.

Supplementary Table S6. The DALY and age-standardized DALY rate of Urolithiasis in 1990 and 2019, and its temporal trends from 1990 to 2019.

Supplementary Table S7. Age distribution of death rate (per 100,000) for Urolithiasis in different countries in 2019.

Supplementary Table S8. Age distribution of DALYs rate (per 100,000) for Urolithiasis in different countries in 2019.

Supplementary Figures

Supplementary figure 1. The incident cases (A), age standardized incidence (B), death (C) and DALYs (D) rates of urolithiasis between 1990 and 2019 both males and females.

Supplementary figure 2 The proportion of different ages in urolithiasis incidence (A) and death (B) by years.

Supplementary figure 3. Distribution of different ages in urolithiasis incidence in global population.

Supplementary figure 4. Distribution of different ages in urolithiasis incidence in female subjects.

Supplementary figure 5. Distribution of different ages in urolithiasis incidence in male subjects.

Supplementary figure 6. The age standardized incidence (A), death (B) and DALY (C) rates of urolithiasis per 100,000 population among regions based on SDI in 2019.

Supplementary figure 7 The global EAPC of Urolithiasis both sex in 192 countries. A. The EAPC of ASIR; B. The EAPC of ASDR; C. The EAPC of DALYs.

Supplementary figure 8. Distribution of different ages in urolithiasis death rate in global level.

Supplementary figure 9. Distribution of different ages in urolithiasis death rate in females.

Supplementary Figure 10. Distribution of different ages in urolithiasis death rate in males.

Supplementary figure 11. The ratio of male to female ASDR among different age groups in global. SDI, socio-demographic index.

Supplementary figure 12. Distribution of different ages in urolithiasis DALYs in global level.

Supplementary figure 13. Distribution of different ages in urolithiasis DALYs in females.

Supplementary Figure 14. Distribution of different ages in urolithiasis DALYs in males.

Supplementary figure 15. The ratio of male to female ASDR among different age groups in global. SDI, socio-demographic index.

Supplementary Table S1 Three countries with the largest and lowest number of incidences, death, or DALYs.

Supplementary Table S1 Three countries with the largest and lowest number of incidences, death, or DALYs.

Measure	Top three countries			Bottom three countries		
2019 ASR (per 100,000 people)						
ASIR	Russian Federation 4541.88	Ukraine 4282.6	Latvia 4156.67	Burundi 525.01	South Sudan 533.43	Madagascar 535.88
ASDR	Armenia 1.82	Kazakhstan 0.88	Philippines 0.65	North Macedonia 0	Montenegro 0	Lebanon 0.01
Age Standardized DALYs Rate	Armenia 33.33	Russian Federation 24.65	Philippines 22.66	Cabo Verde 2.28	Panama 2.49	El Salvador 2.57
1990-2019 increase times						
Incidence (cases)	United Arab Emirates 753.74	Qatar 748.84	Jordan 568.93	Poland -51.49	Bulgaria -38.55	Lithuania -27.4
Death (cases)	Lebanon Inf	Djibouti Inf	Panama Inf	Albania -100	Bulgaria -92.93	Poland -84.62
DALYs (Year)	United Arab Emirates 769.84	Qatar 712.5	Jordan 482.42	Bulgaria -83.4	Poland -73.31	Czechia -70.99
EAPC						
Incidence	Jordan 2.1	Romania 2.01	Germany 2	Poland -3.87	China -2.8	Indonesia -2.79
Death	Costa Rica 7.18	Jamaica 6.74	Turkmenistan 4.77	Bulgaria -10.59	Poland -8.98	Guam -8.97
DALYs	Trinidad and Tobago 2.84	Armenia 2.46	Jamaica 2.27	Poland -5.84	Bulgaria -5.78	China -4.57

ASIR: age-standardized incidence; ASDR: age-standardized death; DALYs: disability adjusted life-years; EAPC: estimated annual percentage change.

Supplement Table S2. Three regions with the largest and lowest number of incidences, deaths, or DALYs.

Supplementary Table S2 Three regions with the largest and lowest number of incidences, death, or DALYs.

Measure	Top three regions			Bottom three regions		
2019ASR (per 100,000 people)						
ASIR	Eastern Europe 4433.72	Central Asia 1787.98	Andean Latin America 1772.43	Eastern Sub-Saharan Africa 565.68	Central Sub-Saharan Africa 575.37	Southern Sub-Saharan Africa 725.49
ASDR	Eastern Europe 0.55	Central Asia 0.44	Southeast Asia 0.4	North Africa and Middle East 0.03	Southern Latin America 0.04	Southern Sub-Saharan Africa 0.05
Age Standardized DALY Rate	Eastern Europe 23.61	Southeast Asia 13.06	Central Asia 12.42	Southern Sub-Saharan Africa 3.46	Western Sub-Saharan Africa 3.61	North Africa and Middle East 4.1
1990-2019 increase in the number of cases/years (-fold)						
Incidence(cases)	Central Sub-Saharan Africa 1.67	North Africa and Middle East 1.56	Western Sub-Saharan Africa 1.51	Central Europe -0.24	Eastern Europe -0.08	High-income North America -0.06
Death(cases)	Tropical Latin America 5.27	High-income Asia Pacific 4.73	Southern Latin America 1.83	Central Europe -0.76	East Asia -0.34	High-middle SDI -0.08
DALY(Year)	Tropical Latin America 2.28	North Africa and Middle East 1.4	Central Latin America 1.33	Central Europe -0.62	East Asia -0.34	Eastern Europe -0.1
EAPCs						
Inceidence	Caribbean 0.66	South Asia 0.64	Western Europe 0.53	East Asia -2.68	High-income North America -2.02	High-middle SDI -1.52
Death	Tropical Latin America 4	High-income Asia Pacific 2.6	Central Asia 1.74	Central Europe -6.31	East Asia -4.93	High-middle SDI -2.76
DALYs	Tropical Latin America 2.07	Caribbean 1.08	Central Asia 0.29	East Asia -4.43	Central Europe -3.18	High-middle SDI -2.41

Supplementary Table S3. The incident cases and age-standardized incidence rate of Urolithiasis in 1990 and 2019, and its temporal trends from 1990 to 2019.

Supplementary Table S3. The incident cases and age-standardized incidence rate of Urolithiasis in 1990 and 2019, and its temporal trends from 1990 to 2019

Nation	Sex	Incident cases No. (95% UI)		Change absolute number (%)	ASIR per 100,000 No. (95% UI)		1990-2019 EAPC No. (95% CI)
		1990	2019		1990	2019	
Afghanistan	Both	907.88 [707.54-1114.7]	2927.22 [2271.55-3627.71]	222.42	1097.81 [856.58-1366.24]	1184.27 [927.24-1467.36]	0.28 [0.22 to 0.34]
Albania	Both	365.18 [289.41-446.61]	432.55 [341.59-537.76]	18.45	1278.5 [1014.03-1562.27]	1284.26 [1027.1-1572.4]	0.02 [-0.02 to 0.06]
Algeria	Both	1996.9 [1573.77-2474.22]	5188.99 [4015.42-6502.67]	159.85	1142.03 [884.94-1429.78]	1214.32 [938.28-1510.07]	0.22 [0.18 to 0.25]
American Samoa	Both	3.52 [2.76-4.44]	5.56 [4.26-6.98]	57.95	1035.56 [816.08-1288.58]	1038.32 [802.58-1300.76]	-0.13 [-0.2 to -0.06]
Andorra	Both	9.01 [7.03-11.32]	17.25 [13.16-21.92]	91.45	1430.06 [1112.26-1784.26]	1417.66 [1105.13-1764.58]	-0.04 [-0.07 to -0.02]
Angola	Both	377.72 [295.32-469.04]	1161.37 [907.72-1439.47]	207.47	538.34 [422.35-665.29]	574.59 [446.56-710.03]	0.27 [0.2 to 0.34]
Antigua and Barbuda	Both	5.26 [4.11-6.48]	12.06 [9.28-15.21]	129.28	1007.28 [775.5-1244.96]	1120.5 [878.39-1394.75]	0.43 [0.4 to 0.47]
Argentina	Both	5263.32 [4063.12-6648.36]	8109.33 [6259.62-10191.86]	54.07	1646.15 [1267.08-2085.16]	1664.07 [1287.41-2095.93]	0.06 [0.03 to 0.09]
Armenia	Both	635.75 [506.77-774.09]	981.2 [738.09-1510.95]	54.34	1977.83 [1596.22-2383.12]	2540.63 [1939.79-3808.42]	1.01 [0.87 to 1.15]
Australia	Both	2531.34 [1979.29-3103.48]	4022.53 [3122.08-5016.6]	58.91	1355.96 [1054.72-1677.63]	1282.28 [986.27-1592.12]	-0.25 [-0.31 to -0.18]
Austria	Both	3022.61 [2725.08-3293.82]	2944.13 [2107.91-4057.29]	-2.6	3156.38 [2860.3-3443.99]	2409.47 [1729.79-3299.85]	-0.39 [-0.58 to -0.21]
Azerbaijan	Both	1011.37 [795.6-1252.55]	1942.62 [1506.64-2435.28]	92.08	1561.18 [1224.2-1926.4]	1670.51 [1320.01-2064.77]	0.28 [0.21 to 0.35]
Bahamas	Both	21.55 [16.9-27.09]	48.81 [37.7-60.72]	126.5	1014.65 [793.52-1255.32]	1110.49 [867.16-1373.13]	0.36 [0.34 to 0.38]
Bahrain	Both	52.88 [40.32-69.23]	256.44 [193.32-337.06]	384.95	1202.04 [917.83-1505.48]	1292.07	0.32 [0.28 to 0.36]

						[1004.2-1626.35]	
Bangladesh	Both	10050.75 [7830.94-12696.4]	23059.21 [18033.75-28549.76]	129.43	1330.7 [1044.12-1667.32]	1484.37 [1161.51-1837.85]	0.4 [0.34 to 0.45]
Barbados	Both	26.28 [20.99-32.36]	48.05 [37.62-59.67]	82.84	1067.59 [843.17-1315.32]	1196 [946.51-1469.55]	0.44 [0.37 to 0.5]
Belarus	Both	5383.8 [4404.95-6487.92]	5300.71 [4220.23-6603.49]	-1.54	4382.06 [3588.86-5277.56]	4017.99 [3181.04-4977.84]	-0.4 [-0.51 to -0.29]
Belgium	Both	1806.5 [1401.19-2254.35]	2336.89 [1800.48-2899.3]	29.36	1486.33 [1151.45-1866.12]	1545.25 [1189.04-1909.61]	1.88 [1.02 to 2.74]
Belize	Both	12.39 [9.83-15.11]	43.25 [34.07-53.72]	249.07	1038.72 [815.9-1283.29]	1195.09 [938.73-1476.8]	0.55 [0.5 to 0.61]
Benin	Both	180.15 [142.44-219.9]	529.09 [419.4-645.24]	193.69	624.96 [491.76-766.27]	677.31 [530.21-834.42]	0.34 [0.26 to 0.42]
Bermuda	Both	6.9 [5.36-8.65]	10.49 [8.11-13.36]	52.03	1021.67 [791.76-1272.93]	1122.78 [873.55-1400.88]	0.33 [0.32 to 0.35]
Bhutan	Both	58 [45.71-73.99]	111.48 [87.42-139.51]	92.21	1324.69 [1048.92-1648.43]	1504.04 [1184.36-1864.77]	0.45 [0.4 to 0.49]
Bolivia (Plurinational State of)	Both	737.07 [569.1-945.7]	1808.23 [1401.48-2294.9]	145.33	1594.5 [1238.57-2015.07]	1661.89 [1284.24-2086.54]	0.18 [0.12 to 0.24]
Bosnia and Herzegovina	Both	593.67 [470.2-725.82]	557.85 [439.96-696.02]	-6.03	1242.34 [988.97-1507.91]	1270.68 [1012.5-1552.54]	0.11 [0.08 to 0.15]
Botswana	Both	58.78 [46.59-72.35]	148.48 [115.43-188.54]	152.6	642.21 [505.79-791.62]	671.78 [520.67-827.97]	0.13 [0.04 to 0.22]
Brazil	Both	12643.44 [10160.92-15433.44]	23733.77 [19275.63-28476.62]	87.72	1036.9 [835.66-1256.84]	969.89 [790.08-1165.75]	-0.38 [-0.46 to -0.29]
Brunei Darussalam	Both	28.84 [22.45-37.08]	70.34 [54.26-89.2]	143.9	1538.37 [1199.75-1905.92]	1496.08 [1168.31-1849.76]	-0.12 [-0.19 to -0.06]
Bulgaria	Both	1932.22 [1523.43-2542.28]	1187.29 [935.58-1480.29]	-38.55	1734.17 [1391.66-2208.49]	1267.09 [1013.2-1537.38]	-1.27 [-1.54 to -0.99]
Burkina Faso	Both	378.66 [300.41-460.67]	976.69 [770.87-1190.24]	157.93	633.41 [502.19-773.91]	686.69 [539.8-844.24]	0.34 [0.29 to 0.39]
Burundi	Both	179.33 [142.38-221.94]	404.77 [317.25-501.1]	125.71	514.89 [403.89-631.71]	525.01 [408.44-646.9]	0.08 [0.03 to 0.12]
Cabo Verde	Both	14.62 [11.68-17.93]	35.74 [28.04-44.08]	144.46	621.14 [493.82-766.21]	686.98 [540-847.34]	0.41 [0.34 to 0.49]
Cambodia	Both	850.68 [690.7-1025.39]	2205.25 [1745.45-2700.61]	159.23	1397.7 [1137.76-1667.82]	1496.36 [1193.87-1823.68]	0.12 [0.09 to 0.16]
Cameroon	Both	423.54 [333.09-518.41]	1329.44	213.89	636.33 [497.63-781.18]	685.95 [536.64-844.09]	0.31 [0.23 to 0.39]

			[1042.91-1635.86]				
Canada	Both	3127.12 [2490.01-3859.73]	5318.75 [4191.62-6698.9]	70.08	1006.97 [803.18-1232.13]	1057.58 [842.25-1298.52]	0.25 [0.22 to 0.28]
Central African Republic	Both	102.6 [80.46-126.82]	218.53 [171.03-268.32]	112.99	532.32 [418.22-660.14]	569.1 [439.49-699.28]	0.29 [0.23 to 0.34]
Chad	Both	234.84 [185.25-286.8]	623.54 [492.94-755.19]	165.52	625.77 [490.57-768.12]	697.84 [545.71-858.78]	0.44 [0.36 to 0.53]
Chile	Both	2024.5 [1572.38-2579.71]	3632.58 [2818.69-4632.47]	79.43	1650.14 [1270.17-2085.88]	1697.13 [1313.63-2162.36]	-0.91 [-1.15 to -0.67]
China	Both	164604.38 [129044.93-204082.93]	176849.19 [140990.66-216234.74]	7.44	1614.23 [1262.27-2011.52]	889.9 [717.04-1075.44]	-2.8 [-3.08 to -2.51]
Colombia	Both	1862.2 [1456.52-2317.12]	3972.46 [3071.66-4928.2]	113.32	744.48 [585.26-915.81]	767.44 [597.06-949.94]	0.14 [0.1 to 0.17]
Comoros	Both	15.55 [12.3-19.01]	33.57 [26.33-41.26]	115.88	515.08 [405.39-628.73]	541.33 [422.81-665.08]	0.2 [0.17 to 0.23]
Congo	Both	88.88 [69.48-109.76]	252.14 [197.89-316.03]	183.69	534.73 [416.09-662.13]	581.49 [456.32-722.66]	0.35 [0.28 to 0.41]
Costa Rica	Both	173.1 [134.7-217.21]	390.35 [303.98-485.74]	125.51	748.92 [580.12-936]	749.51 [583.25-927.12]	0.01 [-0.02 to 0.04]
Cote d'Ivoire	Both	477.44 [375.8-591.3]	1250.03 [975.9-1539.18]	161.82	646.59 [508.11-796.19]	696.7 [546.19-858.21]	0.32 [0.24 to 0.41]
Croatia	Both	724.36 [575.35-898.62]	828.67 [689.07-967.73]	14.4	1253.56 [1004.1-1521.24]	1411.12 [1182.58-1629.74]	0.22 [-0.08 to 0.52]
Cuba	Both	1166.19 [911.89-1441.88]	2224.3 [1727.05-2803.06]	90.73	1093.39 [855.35-1344.78]	1386.08 [1104.01-1723.71]	0.95 [0.89 to 1]
Cyprus	Both	100.75 [78.89-124.49]	207.16 [160.87-260.66]	105.62	1224.18 [953.02-1516.51]	1210.05 [946.15-1518.88]	-1.38 [-2.11 to -0.65]
Czechia	Both	2081.43 [1671.32-2550.29]	1857.88 [1469.67-2311.54]	-10.74	1699.62 [1372.39-2054.31]	1332.17 [1060.66-1621.44]	0.44 [0.03 to 0.85]
Democratic People's Republic of Korea	Both	1923.34 [1490.21-2397.37]	3332.01 [2567.26-4185.91]	73.24	966.06 [757.56-1194.75]	1015.16 [797.59-1264.19]	0.13 [0.1 to 0.16]
Democratic Republic of the Congo	Both	1366.27 [1072.62-1687.66]	3538.75 [2753.94-4388.96]	159.01	531.71 [416.79-659.57]	575.14 [446.78-708.7]	0.31 [0.25 to 0.38]
Denmark	Both	821.63 [647.23-1011.39]	1070.17 [841.06-1329.03]	30.25	1286.26 [1009.26-1595.42]	1344.58 [1057.84-1665.52]	0.22 [0.17 to 0.26]
Djibouti	Both	15 [11.69-18.69]	55.71 [43.65-69.79]	271.4	513.15 [402.1-628.74]	550.04 [429.48-677.65]	0.3 [0.25 to 0.34]

Dominica	Both	6.1 [4.84-7.47]	8.84 [6.78-11.02]	44.92	990.98 [781.04-1232.92]	1103.14 [856.57-1367.7]	0.41 [0.38 to 0.45]
Dominican Republic	Both	521.87 [409.9-647.94]	1147.1 [885.89-1430.84]	119.81	1007.28 [787.19-1250.64]	1089.59 [840.64-1363.63]	0.31 [0.27 to 0.34]
Ecuador	Both	1173.94 [1015.84-1367.25]	3418.63 [3158.17-3681.28]	191.21	1559.46 [1356.06-1793.94]	2023 [1868.57-2177.03]	1.48 [1.25 to 1.71]
Egypt	Both	4893.82 [3834.88-6078.85]	10869.06 [8416.42-13536.5]	122.1	1144.55 [893.69-1432.2]	1218.21 [950.84-1508.08]	0.23 [0.19 to 0.27]
El Salvador	Both	274.36 [214.82-338.96]	439.77 [345.08-544.46]	60.29	733.7 [569.29-911.56]	734.69 [576.84-911.72]	0.03 [-0.01 to 0.06]
Equatorial Guinea	Both	15.49 [12.29-19.28]	55.89 [43.53-70.22]	260.81	529.91 [419.92-657.41]	577.55 [451.96-717.34]	0.37 [0.31 to 0.44]
Eritrea	Both	94.75 [74.38-117.22]	256.53 [200.22-316.07]	170.74	513.22 [403.42-633.97]	543.6 [427.58-670.23]	0.23 [0.2 to 0.26]
Estonia	Both	833.81 [684.36-999.19]	701.64 [555.63-873.03]	-15.85	4417.9 [3616.38-5300.21]	3939.19 [3090.59-4861.57]	-0.53 [-0.65 to -0.4]
Eswatini	Both	33.4 [26.47-41.26]	63.09 [49.87-78.71]	88.89	639.36 [506.97-792.08]	673.04 [531.21-827.5]	0.2 [0.17 to 0.24]
Ethiopia	Both	1976.76 [1565.48-2423.42]	4247.36 [3345.01-5253.61]	114.86	611.27 [480.56-753.66]	601.15 [472.33-737.84]	-0.1 [-0.15 to -0.05]
Fiji	Both	54.78 [42.16-69.45]	93.99 [71.86-117.94]	71.58	953.26 [734.48-1194.19]	1019.49 [784.61-1280.35]	0.22 [0.17 to 0.26]
Finland	Both	732.06 [560.79-921.58]	902.99 [692.84-1143.08]	23.35	1195.66 [915.14-1510.17]	1226.94 [949.83-1534.6]	-0.6 [-1.36 to 0.18]
France	Both	9017.65 [7045.92-11288.16]	11616.95 [8967.78-14634.92]	28.82	1353.16 [1055.09-1690.07]	1371.42 [1053.58-1713.18]	0.05 [0.02 to 0.09]
Gabon	Both	39.43 [31.07-48.47]	89.1 [69.23-110.28]	125.97	540.89 [421.54-668.6]	587.82 [455.3-728.15]	0.35 [0.3 to 0.41]
Gambia	Both	38.1 [30.07-46.95]	100.86 [79.24-123.16]	164.72	647.26 [509.66-786.42]	686.49 [539.84-841.85]	0.26 [0.18 to 0.33]
Germany	Both	12627.56 [9679.57-15855.97]	15304.31 [11755.3-19316.76]	21.2	1247.63 [959.57-1568.43]	1300.93 [1008.39-1616.51]	2 [1.36 to 2.64]
Ghana	Both	802.67 [623.23-1090.17]	2191.38 [1693.38-3114.34]	173.01	897.24 [687.12-1315.17]	999.72 [761.04-1545.52]	0.34 [0.18 to 0.5]
Greece	Both	1712.56 [1313.06-2150.39]	1957.17 [1505.24-2465.13]	14.28	1360.02 [1051.37-1698.05]	1370.26 [1060.65-1712.81]	0.06 [0.03 to 0.08]
Greenland	Both	5.22 [4.07-6.51]	6.89 [5.37-8.75]	31.99	987.25 [778.35-1212.06]	1013.92 [800.31-1249]	0.11 [0.08 to 0.15]
Grenada	Both	7.41 [6.01-9]	18.68 [14.75-22.9]	152.09	1136.11 [915.69-1400.33]	1573.6 [1250.21-1902.1]	1.31 [1.2 to 1.43]

Guam	Both	12.11 [9.45-15.28]	19.46 [14.7-24.63]	60.69	1050.49 [811.43-1307.74]	1048.85 [802.35-1317.85]	-0.13 [-0.22 to -0.04]
Guatemala	Both	395.67 [311.94-488.24]	1072.51 [845.19-1337.95]	171.06	749.6 [590.26-928.82]	747.78 [589.19-921.95]	-0.03 [-0.06 to -0.01]
Guinea	Both	266.73 [211.28-323.4]	547.73 [436.07-667.73]	105.35	630.97 [498.68-772.93]	686.68 [539.54-843.62]	0.37 [0.28 to 0.46]
Guinea-Bissau	Both	39.57 [31.7-48.67]	83.32 [65.51-102.89]	110.56	637.23 [499.78-780.83]	670.31 [526.92-818.13]	0.23 [0.14 to 0.32]
Guyana	Both	59.24 [47.18-73.32]	97.25 [77.5-118.44]	64.16	1068.72 [852.73-1313.02]	1286.55 [1037.63-1563.12]	0.68 [0.58 to 0.78]
Haiti	Both	460.26 [365.07-562.7]	1104.54 [870.25-1377.18]	139.98	1010.5 [801.39-1240.76]	1089.33 [863.28-1337]	0.33 [0.29 to 0.36]
Honduras	Both	254.61 [202.35-311.8]	701.88 [558.26-860.58]	175.67	881.65 [696.88-1078.81]	907.3 [716.55-1111.34]	-0.01 [-0.15 to 0.13]
Hungary	Both	2267.12 [1824.8-2898.19]	1918.31 [1576.38-2347.99]	-15.39	1743.52 [1412.31-2170.94]	1419.87 [1164.68-1700.44]	-0.9 [-1.01 to -0.79]
Iceland	Both	33.06 [25.84-41.37]	54.57 [42.13-67.67]	65.06	1265.19 [983.89-1575.75]	1275.79 [995.02-1577.9]	-0.62 [-0.84 to -0.39]
India	Both	107107.13 [84666.34-133036.17]	252913.59 [198829.54-314446.62]	136.13	1555.76 [1239.59-1926.8]	1821.01 [1434.25-2260.91]	0.71 [0.53 to 0.88]
Indonesia	Both	29227.43 [22990.91-35721.74]	33197.48 [26628.63-39453.81]	13.58	2219.91 [1747.05-2720.3]	1221.13 [995.99-1451.71]	-2.79 [-3.21 to -2.38]
Iran (Islamic Republic of)	Both	5046.4 [3971.59-6211.55]	12351.02 [9562.41-15637.73]	144.75	1244.55 [971.16-1551.16]	1321.27 [1034.54-1647.31]	0.22 [0.15 to 0.28]
Iraq	Both	1334.37 [1056.25-1635.86]	4340.91 [3411.54-5417.78]	225.32	1157.28 [909.38-1432.04]	1216.19 [956.79-1515.1]	0.15 [0.11 to 0.19]
Ireland	Both	495.54 [387.95-610.28]	839.62 [643.23-1055.81]	69.44	1371.53 [1063.21-1701.53]	1376.24 [1066.09-1717.13]	0.04 [0.01 to 0.06]
Israel	Both	617.35 [479.8-770.42]	1381.9 [1080.17-1709.18]	123.84	1343.34 [1035.81-1680.34]	1416.27 [1100.08-1761.17]	0.25 [0.22 to 0.27]
Italy	Both	12339.43 [9613.41-15354.02]	13633.7 [11067.82-16774.69]	10.49	1733.39 [1357.48-2164.5]	1553.49 [1250.12-1877.97]	-0.57 [-0.65 to -0.49]
Jamaica	Both	184.5 [145.27-227.66]	349.2 [274.46-432.35]	89.27	1010.25 [778.4-1260.28]	1165.08 [920.28-1442.38]	0.59 [0.55 to 0.62]

Japan	Both	24922.86 [18856.84-31447.6]	27038.97 [22099.47-32826.33]	8.49	1579.43 [1216.08-1973.87]	1487.85 [1195.07-1792.3]	-0.39 [-0.49 to -0.28]
Jordan	Both	276.64 [216.61-344.7]	1850.53 [1364.66-2686.79]	568.93	1143.8 [881.28-1425.93]	1792.23 [1331.12-2622.06]	2.1 [1.72 to 2.49]
Kazakhstan	Both	3142.24 [2495.11-3860.26]	3739.06 [3010.21-4566.8]	18.99	2117.87 [1675.94-2607.86]	1947.07 [1579.25-2360.53]	-0.63 [-0.76 to -0.5]
Kenya	Both	794.85 [628.41-974.92]	2242.37 [1768.87-2766.1]	182.11	582.84 [456.96-714.69]	610.41 [479.37-750.16]	0.53 [0.36 to 0.7]
Kuwait	Both	183.92 [140.43-243.23]	661.48 [502.81-859.8]	259.66	1210.93 [940.48-1519.11]	1246.6 [959.4-1548.12]	0.12 [0.09 to 0.14]
Kyrgyzstan	Both	701.44 [556.81-857.49]	1164.81 [919.04-1430.87]	66.06	1923.5 [1526.96-2360.06]	1907.44 [1520.62-2345.03]	0.81 [0.48 to 1.14]
Lao People's Democratic Republic	Both	383.3 [311.06-462.39]	843.08 [662.28-1040.98]	119.95	1431.19 [1160.33-1726.98]	1415.83 [1131.06-1730.5]	-0.2 [-0.26 to -0.13]
Latvia	Both	1534.87 [1263.73-1835.46]	1169.08 [968.02-1408.37]	-23.83	4653.72 [3823.81-5570.62]	4156.67 [3404.74-5049.01]	-0.88 [-1.08 to -0.67]
Lebanon	Both	310.55 [241.27-387.46]	645.65 [496.55-813.64]	107.91	1134.76 [884.94-1415.86]	1202.23 [927.53-1500.36]	0.22 [0.19 to 0.25]
Lesotho	Both	86.74 [69.02-106.03]	125.71 [98.82-156.39]	44.93	641.71 [507.23-786.51]	669.73 [527.81-819.32]	0.17 [0.11 to 0.23]
Liberia	Both	88.28 [69.77-108.06]	234.15 [183.23-287.74]	165.24	644.28 [504.61-785.79]	694.88 [542.45-859.58]	0.34 [0.26 to 0.42]
Libya	Both	333.06 [260.26-414.57]	903.62 [696.02-1159.26]	171.31	1173.43 [921.76-1453.92]	1214.85 [946.64-1515.64]	0.16 [0.12 to 0.2]
Lithuania	Both	2147.62 [1779.62-2542.75]	1559.2 [1236.65-1942.71]	-27.4	5022.74 [4162.8-5935.79]	3993.76 [3171.52-4918.5]	-1.33 [-1.5 to -1.17]
Luxembourg	Both	71.06 [54.64-88.4]	127.87 [98.33-161.51]	79.95	1504.71 [1153.94-1879.41]	1558.26 [1215.2-1963.17]	0.06 [-0.65 to 0.78]
Madagascar	Both	390.89 [307.17-476.65]	1001.97 [785.14-1249.08]	156.33	514.49 [403.97-634.66]	535.88 [419.71-656.23]	0.16 [0.12 to 0.21]
Malawi	Both	309.35 [243.74-382.91]	642.2 [504.78-794.8]	107.6	515 [403.84-635.84]	538.94 [423.5-660.74]	0.2 [0.17 to 0.23]
Malaysia	Both	1641.61 [1263.97-2057.5]	4413.98 [3377.64-5497.27]	168.88	1279.74 [980.88-1607.34]	1400.71 [1073.18-1746.84]	0.33 [0.27 to 0.39]
Maldives	Both	17.34 [13.35-21.4]	76.34 [58.08-97.93]	340.25	1357.43 [1048.21-1698.81]	1538.13 [1172.88-1933.53]	0.44 [0.31 to 0.57]
Mali	Both	363.2 [288.99-442.53]	902.47 [718.29-1095.76]	148.48	638.39 [504.82-780.4]	694.13 [540.49-852.61]	0.32 [0.26 to 0.39]

Malta	Both	62.56 [48.49-77.58]	94.32 [73.04-117.69]	50.77	1489.4 [1154.15-1843.73]	1544.27 [1202.82-1918.41]	1.92 [1.25 to 2.59]
Marshall Islands	Both	2.61 [2.07-3.29]	5.43 [4.23-6.79]	108.05	1034.16 [815.14-1277.12]	1073.78 [846.91-1337.84]	0.07 [0.05 to 0.09]
Mauritania	Both	87.44 [69.7-106.58]	198.44 [157.51-242.03]	126.94	643.1 [503.28-783.64]	701.94 [551.05-861.3]	0.33 [0.27 to 0.4]
Mauritius	Both	120.67 [93.51-150.5]	234.89 [177.75-302.69]	94.65	1292.66 [1003.38-1627.83]	1381.01 [1056.36-1735.85]	0.22 [0.17 to 0.27]
Mexico	Both	6753.18 [5371.15-8298.09]	15866.02 [12693.8-19032.39]	134.94	1168.03 [925.73-1441.21]	1226.77 [988.55-1468.54]	0.2 [-0.45 to 0.85]
Micronesia (Federated States of)	Both	6.5 [5.09-8.09]	9.8 [7.56-12.17]	50.77	990.65 [775.97-1231.17]	1043.5 [812.48-1295.87]	0.14 [0.12 to 0.17]
Mongolia	Both	247.64 [196.53-306.94]	558.64 [433.8-702.46]	125.59	1590.74 [1248.03-1948.25]	1658.27 [1301.87-2047.56]	0.19 [0.12 to 0.26]
Montenegro	Both	83.06 [65.61-101.76]	100.01 [78.9-123.67]	20.41	1280.76 [1018.6-1562.55]	1285.18 [1030.46-1572.79]	0.03 [0 to 0.06]
Morocco	Both	2183.21 [1710.08-2704.43]	4531.87 [3460.29-5677.39]	107.58	1132.73 [874.05-1409.33]	1201.07 [926.12-1497.88]	0.21 [0.16 to 0.26]
Mozambique	Both	454.91 [359.62-559.95]	1014.66 [806.32-1250.61]	123.05	515.51 [405.23-635.63]	562.38 [442.82-693.55]	0.37 [0.34 to 0.4]
Myanmar	Both	4528.78 [3675.28-5460.76]	8050.94 [6383.54-9799.73]	77.77	1551.16 [1256.07-1858.9]	1476.21 [1184.5-1788.06]	-0.35 [-0.44 to -0.26]
Namibia	Both	65.64 [51.64-80.97]	135.97 [106.22-169.95]	107.15	642.44 [505.03-793.75]	665.14 [524.36-824.5]	0.15 [0.09 to 0.21]
Nepal	Both	1892.55 [1485.03-2376.66]	4009.03 [3143.09-4930.76]	111.83	1309.13 [1033.24-1640.25]	1445.54 [1131.2-1789]	0.36 [0.3 to 0.42]
Netherlands	Both	2712.15 [2151.25-3358.43]	3411.44 [2659.96-4280.02]	25.78	1518.48 [1195.86-1888.49]	1448.71 [1133.74-1803.4]	-0.23 [-0.27 to -0.2]
New Zealand	Both	608.97 [479.45-758.12]	747.17 [627.11-887.27]	22.69	1655.45 [1296.13-2071.05]	1288.37 [1082.49-1508.08]	-0.83 [-1.06 to -0.6]
Nicaragua	Both	172.19 [135.2-214.34]	435.9 [337.7-549.14]	153.15	735.36 [572.89-913.31]	749.44 [582.73-924.68]	0.11 [0.07 to 0.14]
Niger	Both	298.44 [235.97-365.12]	859.41 [683.89-1039.99]	187.97	639.89 [503.84-781.72]	683.57 [536.7-838.42]	0.29 [0.21 to 0.37]
Nigeria	Both	4396.26 [3482.88-5377.95]	10400.07 [8222.98-12765.35]	136.57	706.16 [558.87-868.67]	732.17 [579.07-897.2]	0.18 [0.1 to 0.26]

North Macedonia	Both	263.54 [210.14-322.54]	353.93 [279.3-440.05]	34.3	1280.32 [1026.85-1566.27]	1289.99 [1034.39-1578.54]	0.05 [0.02 to 0.08]
Northern Mariana Islands	Both	4.09 [3.12-5.31]	6.16 [4.54-7.95]	50.61	1058.48 [815.58-1321.94]	1061.68 [814.6-1327]	-0.02 [-0.05 to 0.02]
Norway	Both	1347.55 [1057.81-1654.32]	1767.67 [1384.45-2198.98]	31.18	2605.46 [2057.64-3211.68]	2537.56 [1985.32-3154.17]	0.64 [0.41 to 0.87]
Oman	Both	173.34 [134.16-224.34]	623.34 [474.63-831.3]	259.61	1210.34 [940.7-1520.44]	1309.32 [1014.21-1643.65]	0.31 [0.24 to 0.38]
Pakistan	Both	11265.77 [8819.77-14005.3]	27209.93 [21309.79-34129.52]	141.53	1410.4 [1109.74-1752.55]	1542.81 [1202.33-1922.15]	0.31 [0.22 to 0.41]
Palestine	Both	141.41 [111.64-174.61]	465.8 [362.17-586.15]	229.4	1123.17 [868.15-1405.33]	1208.65 [943.36-1513.2]	0.28 [0.25 to 0.31]
Panama	Both	142.02 [111.98-176.17]	317.9 [244.02-395.5]	123.84	749.55 [581.96-932.81]	754.15 [581.71-936.92]	0.02 [-0.01 to 0.05]
Papua New Guinea	Both	275.08 [213.86-343.96]	784.93 [603.99-994.55]	185.35	973.82 [755.39-1215.32]	1031.41 [795.86-1294.47]	0.16 [0.1 to 0.21]
Paraguay	Both	280.56 [220.62-349.07]	635.51 [499.62-789.27]	126.51	943.67 [738.08-1163.44]	961.65 [759.04-1188.35]	0.08 [0.06 to 0.1]
Peru	Both	2681.34 [2067.6-3415.79]	5846.17 [4496.49-7426.47]	118.03	1635.69 [1264.27-2071.98]	1684.65 [1297.75-2127.67]	0.14 [0.09 to 0.18]
Philippines	Both	9440.02 [7616.17-11362.15]	26316.29 [21160.32-31827.67]	178.77	2365.26 [1900.48-2848.35]	2696.74 [2177.83-3255.03]	0.4 [0.24 to 0.57]
Poland	Both	8983.12 [6920.23-11333.35]	4358.15 [3766.19-5051.79]	-51.49	2112.96 [1642.61-2634.18]	873.44 [762.46-999.21]	-3.87 [-4.49 to -3.24]
Portugal	Both	1386.2 [1074.4-1737.74]	1884.09 [1652.32-2181.37]	35.92	1180.14 [918.4-1476.43]	1249.49 [1091.66-1438.16]	0.3 [0.26 to 0.35]
Puerto Rico	Both	362.49 [281.22-452.02]	506.54 [388.11-642.54]	39.74	1012.68 [781.72-1269.84]	1087.25 [840.82-1357.17]	0.29 [0.26 to 0.32]
Qatar	Both	54.69 [41.06-73.85]	464.23 [353.43-609.67]	748.84	1251.05 [975.44-1570.6]	1363.09 [1062.01-1706.32]	0.38 [0.34 to 0.42]
Republic of Korea	Both	5833.98 [4500.72-7375.2]	11194.01 [8486.94-14161.07]	91.88	1397.08 [1078.97-1754.82]	1443.72 [1109.59-1807.66]	0.1 [0.06 to 0.14]
Republic of Moldova	Both	1743.73	1972.57	13.12	3746.5 [2984.79-4570.33]	3918.49	0.31 [0.24 to 0.38]

		[1382.72-2129.57]	[1558.73-2467.52]			[3057.48-4877.04]	
Romania	Both	3505.33 [2779.85-4322.72]	3419.41 [2666.93-4261.98]	-2.45	1350.93 [1075.78-1655.13]	1354.04 [1075.96-1656.63]	2.01 [1.34 to 2.69]
Russian Federation	Both	93039.77 [75226.46-113130.48]	90606.58 [72773.88-111108.13]	-2.62	5238.69 [4218.41-6314.79]	4541.88 [3648.94-5522]	-0.62 [-0.8 to -0.45]
Rwanda	Both	231.48 [184.66-284.9]	505.52 [397.03-627.93]	118.39	525.06 [410.46-643.69]	542.34 [427.05-670.24]	0.13 [0.08 to 0.18]
Saint Lucia	Both	10.5 [8.38-12.78]	25.52 [19.77-31.89]	143.05	1051.09 [826.03-1296.45]	1187.62 [933.68-1471.61]	0.47 [0.45 to 0.49]
Saint Vincent and the Grenadines	Both	8.6 [6.83-10.54]	16.99 [13.36-20.94]	97.56	1086.5 [858.72-1337.62]	1276 [1014.06-1571.81]	0.65 [0.61 to 0.69]
Samoa	Both	11.11 [8.71-13.65]	18.78 [14.47-23.29]	69.04	1006.99 [785.02-1246.56]	1067.93 [827.24-1324.48]	0.13 [0.1 to 0.16]
Sao Tome and Principe	Both	4.99 [4.01-6.07]	10.88 [8.59-13.38]	118.04	634.07 [505.44-778.77]	696.66 [549.24-858.51]	0.36 [0.3 to 0.42]
Saudi Arabia	Both	1367.88 [1064.73-1730.92]	5121.7 [3895.82-6755.78]	274.43	1198.47 [934.96-1496.56]	1273.28 [989.02-1597.24]	0.22 [0.18 to 0.26]
Senegal	Both	298.35 [237.35-362.89]	724.61 [571.43-884.76]	142.87	636.32 [500.79-784.54]	683.92 [535.09-841.96]	0.32 [0.24 to 0.4]
Serbia	Both	1318.07 [1042.3-1624.77]	1392.65 [1091.29-1729.76]	5.66	1224.07 [975.78-1491.42]	1245.04 [993.33-1518.06]	1.63 [1.11 to 2.16]
Seychelles	Both	7.69 [6.14-9.53]	18.61 [14.55-23.15]	142	1313.35 [1038.67-1642.68]	1509.98 [1197.97-1850.35]	0.47 [0.42 to 0.52]
Sierra Leone	Both	159.82 [126.72-195.06]	382.6 [303.42-470.36]	139.39	634.26 [501.13-775.24]	689.39 [545.14-853.67]	0.36 [0.27 to 0.45]
Singapore	Both	451.26 [353.12-569.76]	1166.82 [879.64-1483.48]	158.57	1475.38 [1151.98-1845.83]	1460 [1121.81-1834.12]	-0.02 [-0.09 to 0.04]
Slovakia	Both	813.98 [657.1-992.53]	994.42 [777.9-1235.28]	22.17	1442.9 [1158.93-1747.18]	1416.67 [1127.33-1728.94]	0.23 [-0.6 to 1.07]
Slovenia	Both	271.76 [215.71-332.32]	334.61 [262.02-417.46]	23.13	1210.66 [967.73-1467.35]	1212.3 [964.1-1480.7]	0.7 [0.18 to 1.22]
Solomon Islands	Both	21.12 [16.65-26.32]	51.24 [39.72-64.55]	142.61	1023.8 [806.72-1266.95]	1050.73 [819.23-1312.54]	0.01 [-0.02 to 0.04]
Somalia	Both	239.27 [188.37-299.3]	663.45 [520.6-819.84]	177.28	525.29 [412.33-642.65]	542.22 [422.76-665.05]	0.13 [0.11 to 0.16]
South Africa	Both	2176.02 [1716.56-2711.05]	4186.32 [3284.48-5192.76]	92.38	714.12 [561.63-879.16]	740.36 [584.86-910.43]	0.12 [0.07 to 0.17]

South Sudan	Both	184.63 [144.88-228.21]	331.11 [260.5-410.15]	79.34	497.78 [390.8-614.51]	533.43 [416.22-657.46]	0.26 [0.22 to 0.31]
Spain	Both	6028.83 [4705.61-7516.07]	8919.32 [6859.73-11222.45]	47.94	1351.29 [1058.79-1690.45]	1388.66 [1084.79-1732.65]	0.12 [0.1 to 0.14]
Sri Lanka	Both	1858.41 [1428.26-2322.33]	3470.37 [2680.43-4361.37]	86.74	1321.85 [1015.11-1647.53]	1354.93 [1049.65-1685.03]	0.09 [0.02 to 0.15]
Sudan	Both	1524.17 [1202.03-1900.71]	3624.84 [2818.41-4545.32]	137.82	1125.89 [870.78-1407.81]	1193.14 [927.08-1475.74]	0.22 [0.18 to 0.26]
Suriname	Both	36.2 [28.89-44.16]	91.38 [72.65-111.63]	152.43	1148.04 [915.69-1402.14]	1429.08 [1150.25-1721.64]	0.88 [0.76 to 1]
Sweden	Both	1702.18 [1323.7-2095.53]	1866.73 [1476.28-2331.73]	9.67	1541.09 [1200.52-1894.11]	1348.1 [1057-1661.9]	-0.14 [-0.43 to 0.15]
Switzerland	Both	1249.07 [972.43-1558.57]	1793.38 [1371.09-2253.98]	43.58	1477.16 [1144.37-1842.46]	1503.18 [1160.4-1867.68]	0.24 [-0.17 to 0.66]
Syrian Arab	Both	924.04 [727.89-1136.71]	1772.83 [1371.93-2230.28]	91.86	1138.87 [884.63-1409.61]	1194.85 [934.41-1495.66]	0.21 [0.18 to 0.23]
Tajikistan Both	Both	625.19 [497.01-772.61]	1434.87 [1139.72-1776.74]	129.51	1612.29 [1275.62-1970.71]	1742.18 [1401.67-2129.84]	0.29 [0.26 to 0.33]
Thailand	Both	8317.68 [6660.83-9995.3]	15956.28 [12735.99-19814.25]	91.84	1824.82 [1468.04-2202.06]	1608.4 [1301.43-1968.53]	-0.76 [-0.94 to -0.58]
Timor-Leste	Both	63.66 [49.86-79.61]	130.82 [104.2-160.73]	105.5	1306.52 [1045.35-1613.49]	1370.39 [1085.12-1697.64]	0.16 [0.1 to 0.22]
Togo	Both	134.78 [106.58-165.96]	385 [301.77-477.22]	185.65	630.75 [502.91-769.46]	665.35 [525.65-817.76]	0.25 [0.16 to 0.34]
Tonga	Both	6.47 [5.07-7.97]	8.91 [6.91-11.05]	37.71	952.95 [748.19-1187.94]	1029.01 [801.66-1287.89]	0.24 [0.21 to 0.27]
Trinidad and Tobago	Both	124.89 [98.31-153.84]	320.35 [240.03-533.22]	156.51	1271.59 [983.96-1570.85]	1768.54 [1338.69-2882.31]	1.52 [1.29 to 1.75]
Tunisia	Both	752.88 [589.35-928.99]	1615.39 [1243.63-2038.77]	114.56	1143.41 [884.55-1418.63]	1212.34 [946.37-1517.71]	0.2 [0.17 to 0.23]
Turkey	Both	5525.16 [4360.98-6868.43]	11630.34 [9102.79-14693.51]	110.5	1132.32 [891.87-1412.05]	1232.54 [971.68-1540.71]	0.34 [0.31 to 0.36]
Turkmenistan	Both	433.8 [343.36-540.22]	837.47 [655.33-1035.46]	93.05	1548.21 [1218.54-1895.93]	1668.62	0.31 [0.23 to 0.4]

						[1315.54-2051.08]	
Uganda	Both	514.6 [405.41-634.68]	1312.08 [1026.08-1614.26]	154.97	506.91 [396.42-625.03]	536.57 [417.29-660.79]	0.21 [0.17 to 0.25]
Ukraine	Both	34085.09 [27545.42-41696.62]	26028.78 [20652.93-32158.42]	-23.64	5185.73 [4190.92-6278.78]	4282.6 [3377.63-5271.76]	-0.94 [-1.11 to -0.78]
United Arab Emirates	Both	214.72 [161.44-288.58]	1833.16 [1352.58-2476.01]	753.74	1247.16 [953.67-1567.2]	1337.56 [1047.6-1680.12]	0.28 [0.25 to 0.31]
United Kingdom	Both	11391.58 [8898.91-14127.61]	15833.33 [12810.85-19179.88]	38.99	1645.59 [1282.38-2047.69]	1792.3 [1447.42-2165.2]	0.83 [0.6 to 1.06]
United Republic of Tanzania	Both	823.66 [650.12-1008.99]	2062.76 [1615.42-2546.13]	150.44	509.95 [399.86-624.39]	540.14 [422.76-658.6]	0.23 [0.18 to 0.28]
United States of America	Both	47470.78 [37171.95-59148.02]	42075.88 [36334.18-49001.38]	-11.36	1690.06 [1325.57-2098.28]	974.46 [844.52-1120.12]	-2.25 [-2.6 to -1.89]
United States Virgin Islands	Both	12.61 [10.05-15.56]	23.63 [17.82-29.58]	87.39	1210.1 [963.56-1474.27]	1571.62 [1217.85-1928.31]	1.19 [0.92 to 1.46]
Uruguay	Both	531.81 [412.12-666.17]	651.63 [505.58-819.33]	22.53	1639.67 [1263.3-2080.34]	1676.55 [1305.44-2117.06]	0.1 [0.07 to 0.14]
Uzbekistan	Both	2496.08 [1955.02-3087.16]	5359.68 [4217.91-6683.24]	114.72	1592.65 [1242.96-1967.21]	1679.65 [1334.46-2080.39]	0.21 [0.15 to 0.26]
Vanuatu	Both	9.39 [7.32-11.8]	23.15 [17.74-29.01]	146.54	961.91 [741.15-1194]	1018.25 [774.9-1274.89]	0.17 [0.11 to 0.23]
Venezuela (Bolivarian Republi of)	Both	1142.66 [907.23-1412.47]	2640.88 [2080.93-3274.78]	131.12	831.17 [653.94-1019.67]	860.12 [684.06-1054.01]	0.02 [-0.07 to 0.12]
Viet Nam	Both	6497.76 [5131.48-7977.18]	22963.11 [17264.55-30850.75]	253.4	1368.93 [1079-1693.7]	2074.64 [1579.98-2770.63]	1.85 [1.53 to 2.17]
Yemen	Both	919.15 [728.93-1140.54]	2728.01 [2141.17-3435.02]	196.8	1127.75 [878.28-1407.55]	1198.72 [930.36-1489.5]	0.22 [0.17 to 0.26]
Zambia	Both	250.15 [195.86-307.92]	646.13 [509.83-810.74]	158.3	533.17 [421.02-655.07]	542.89 [426.97-666.88]	0.05 [0.01 to 0.1]
Zimbabwe	Both	467.13 [370.48-577.49]	801.86 [635.6-996.27]	71.66	678.92 [538.53-830.83]	687.19 [547.16-846.74]	0 [-0.02 to 0.02]
Afghanistan	Male	530.16 [412.47-649.27]	1841.97 [1419.55-2322.59]	247.44	1363.7 [1049.67-1702.91]	1442.52 [1127.26-1803.49]	0.21 [0.16 to 0.27]
Albania	Male	185.68 [146.69-227.65]	221.24 [173.74-275.34]	19.15	1301.69 [1028.02-1589.91]	1308.67	0.01 [-0.04 to

						[1040.33-1600.14]	0.06]
Algeria	Male	1222.64 [959.33-1540.72]	3212.85 [2475.47-4069.63]	162.78	1392.48 [1070.16-1761.38]	1481.75 [1142.16-1854.09]	0.23 [0.19 to 0.27]
American Samoa	Male	2.43 [1.9-3.07]	3.78 [2.89-4.77]	55.56	1374.86 [1086.14-1712.19]	1432.4 [1107.29-1802.26]	0.01 [-0.05 to 0.07]
Andorra	Male	6.75 [5.26-8.52]	12.88 [9.83-16.44]	90.81	2015.39 [1567.6-2536.3]	2023.35 [1566.48-2528.63]	0.02 [0 to 0.05]
Angola	Male	216.75 [168.56-269.75]	630.54 [489.69-781.65]	190.91	625.3 [491.5-779.89]	687.85 [536.92-850.76]	0.36 [0.29 to 0.43]
Antigua and Barbuda	Male	3.29 [2.55-4.1]	7.61 [5.82-9.68]	131.31	1349.4 [1035.88-1680.03]	1470.47 [1135.04-1846.28]	0.33 [0.3 to 0.37]
Argentina	Male	3293.21 [2533.21-4191.67]	5090.54 [3939.05-6413.35]	54.58	2130.12 [1634.83-2712.06]	2160.18 [1677.51-2720.65]	0.08 [0.06 to 0.11]
Armenia	Male	422.61 [330.44-517.68]	649.23 [487.24-1041.28]	53.62	2861.34 [2292.97-3472.28]	3696.88 [2796.35-5806.66]	1 [0.82 to 1.18]
Australia	Male	1841.9 [1435.37-2269.98]	2924.39 [2273.34-3679.65]	58.77	2012.79 [1568.04-2475.77]	1875.87 [1448.97-2347.86]	-0.31 [-0.39 to -0.23]
Austria	Male	2109.03 [1962.73-2254.13]	2175.38 [1536.56-3001.93]	3.15	4737.48 [4406.2-5059.26]	3578.5 [2511.31-4905.68]	-0.26 [-0.47 to -0.06]
Azerbaijan	Male	635.09 [497.16-794.21]	1247.23 [969.76-1560.54]	96.39	2119.84 [1669.86-2628.31]	2239.47 [1768.92-2754.82]	0.24 [0.16 to 0.32]
Bahamas	Male	13.51 [10.56-16.87]	31.56 [24.33-39.38]	133.6	1371.73 [1072.27-1704.34]	1516.36 [1186.94-1872.83]	0.39 [0.37 to 0.42]
Bahrain	Male	39.11 [29.4-52.46]	195.94 [146.29-259.64]	401	1385.26 [1065.26-1737.41]	1484.03 [1154.44-1874.79]	0.27 [0.24 to 0.3]
Bangladesh	Male	6337.54 [4962.92-7974.31]	14496.56 [11433.63-17898.69]	128.74	1644.28 [1296.28-2053.25]	1914.32 [1505.22-2366.83]	0.56 [0.49 to 0.63]
Barbados	Male	17.08 [13.72-21.06]	32.81 [25.82-40.85]	92.1	1504.29 [1195.49-1862.55]	1686.25 [1336.99-2081.03]	0.43 [0.34 to 0.53]
Belarus	Male	3451.05 [2796.83-4155.36]	3353.9 [2666.21-4179.56]	-2.82	6435.61 [5264.21-7711.92]	5655.88 [4525.53-7016.08]	-0.55 [-0.65 to -0.45]
Belgium	Male	1311.88	1706.69	30.09	2195.88 [1703.95-2753]	2262.35	1.96 [1.06 to 2.88]

		[1015.98-1644.33]	[1304.05-2126.74]			[1739.4-2797.87]	
Belize	Male	8.11 [6.39-9.89]	28.18 [22.16-35.05]	247.47	1351.23 [1056.09-1670.69]	1590.64 [1243.19-1975.38]	0.63 [0.6 to 0.67]
Benin	Male	99.36 [78.88-121.1]	305.45 [241.76-372.45]	207.42	769.39 [604.76-950.6]	864.49 [677.47-1072.33]	0.46 [0.37 to 0.55]
Bermuda	Male	4.36 [3.36-5.52]	6.92 [5.26-8.89]	58.72	1359.8 [1046.61-1713.09]	1491.41 [1142.13-1871.61]	0.35 [0.33 to 0.37]
Bhutan	Male	36.85 [29.25-46.1]	73.05 [57.27-90.94]	98.24	1671.81 [1330.18-2072.07]	1920.69 [1514.75-2374.08]	0.47 [0.41 to 0.53]
Bolivia (Plurinational State of)	Male	465.44 [359.12-600.3]	1154.88 [887.74-1470.98]	148.13	2091.89 [1624.71-2624.14]	2150.55 [1652.93-2703.68]	0.13 [0.07 to 0.18]
Bosnia and Herzegovina	Male	295.28 [232.79-361.74]	282.29 [221.57-356.49]	-4.4	1289.15 [1032.95-1568.87]	1297.82 [1035.68-1589.52]	0.02 [-0.02 to 0.06]
Botswana	Male	36.97 [29.34-45.51]	95.68 [74.04-122.16]	158.8	873.5 [688.2-1072.57]	910.73 [707.38-1121.7]	0.29 [0.21 to 0.37]
Brazil	Male	7126.4 [5703.61-8655.77]	13416.74 [10855.69-16130.66]	88.27	1237.12 [993.37-1495.37]	1151.05 [935.98-1380.8]	-0.41 [-0.5 to -0.32]
Brunei Darussalam	Male	22.84 [17.66-29.81]	53.47 [40.9-67.77]	134.11	2222.34 [1726.24-2755.36]	2170.88 [1688.63-2704.39]	-0.1 [-0.14 to -0.06]
Bulgaria	Male	1073.37 [835.56-1400.36]	606.01 [477.83-760.29]	-43.54	1956.32 [1555.99-2488.98]	1298.66 [1043.32-1581.21]	-1.71 [-2.04 to -1.38]
Burkina Faso	Male	207.72 [166.99-251.8]	545.68 [431.29-666.38]	162.7	774.64 [619.56-956.54]	876.34 [683.44-1091.78]	0.49 [0.41 to 0.57]
Burundi	Male	64.58 [50.61-79.94]	163.41 [128.45-202.51]	153.03	394.22 [305.95-492.8]	418.72 [326.78-520.37]	0.23 [0.18 to 0.28]
Cabo Verde	Male	8.07 [6.53-9.88]	21.83 [17.07-27.11]	170.51	796.48 [628.55-981.3]	894.37 [701.08-1116.15]	0.47 [0.39 to 0.55]
Cambodia	Male	645.33 [526.85-776.87]	1710.95 [1362.42-2085.87]	165.13	2480.79 [2013.9-2961.96]	2589.28 [2092.26-3133.34]	0.01 [-0.04 to 0.06]
Cameroon	Male	239.91 [188.36-292.54]	782.2 [609.51-968.08]	226.04	769.91 [600.54-950.88]	867.49 [673.36-1081.7]	0.5 [0.4 to 0.6]
Canada	Male	1766.81 [1402.76-2179.64]	3249.25 [2532.8-4120.87]	83.9	1183.27 [946.69-1456.84]	1261.21 [1001.36-1568.07]	0.32 [0.29 to 0.36]
Central African Republic	Male	56.46 [44.62-69.65]	122.05 [94.59-149.32]	116.17	626.57 [496.46-774.51]	681.14 [523.44-829.44]	0.33 [0.27 to 0.38]
Chad	Male	132.67 [104.4-162.66]	371.23 [292.59-450.78]	179.81	772.42 [597.95-960.4]	862.7 [668.86-1080.22]	0.45 [0.35 to 0.55]
Chile	Male	1254.01 [973.43-1584.2]	2315.41 [1790.02-2931.53]	84.64	2145.57 [1644.12-2694.23]	2219.03 [1717.59-2806.92]	-0.89 [-1.13 to -0.65]

China	Male	122600.4 [96334.69-152385.27]	122002.03 [97221.41-148030.76]	-0.49	2383.9 [1869.55-2971.21]	1233.8 [998.24-1484.71]	-3.06 [-3.37 to -2.75]
Colombia	Male	1102.86 [864.95-1375.32]	2246.06 [1757.16-2791.25]	103.66	889.94 [698.68-1097.05]	917.34 [721.64-1131.31]	0.14 [0.11 to 0.16]
Comoros	Male	5.77 [4.52-7.13]	12.59 [9.79-15.5]	118.2	396.38 [307.05-492.66]	418.15 [323.24-516.7]	0.21 [0.17 to 0.26]
Congo	Male	48.67 [38.43-60.5]	144.1 [112.92-180.21]	196.08	633.41 [494.98-782.74]	686.54 [545.24-845.83]	0.31 [0.25 to 0.38]
Costa Rica	Male	103.23 [80.16-129.64]	221.04 [173.51-274.11]	114.12	890.59 [690.31-1114.79]	898.27 [704.56-1109.08]	0.04 [0.01 to 0.07]
Cote d'Ivoire	Male	287.55 [224.51-353.08]	771.06 [604.29-939.51]	168.15	773.84 [600.91-963.42]	866.29 [672.82-1080.6]	0.46 [0.36 to 0.56]
Croatia	Male	374.2 [294.1-466.16]	474.78 [382.98-572.91]	26.88	1362.65 [1089.98-1670.01]	1631.06 [1329.57-1942.21]	0.5 [0.11 to 0.88]
Cuba	Male	767.35 [601.59-942.76]	1566.53 [1227.47-1974.51]	104.15	1453.52 [1137.13-1793.01]	1957.27 [1547.71-2453.52]	1.17 [1.11 to 1.23]
Cyprus	Male	73.04 [57.32-90.5]	147.48 [114.58-187.72]	101.92	1811.42 [1414.83-2242.7]	1768.86 [1373.7-2222.47]	-1.51 [-2.28 to -0.74]
Czechia	Male	1073.13 [862.13-1336.93]	980.38 [765.09-1223.74]	-8.64	1894.33 [1534.05-2333.6]	1380.05 [1091.55-1697.26]	0.87 [0.24 to 1.49]
Democratic People's Republic of Korea	Male	1270.46 [982.66-1589.58]	2293.33 [1778.23-2887.96]	80.51	1432.98 [1135.12-1761.73]	1442.45 [1141.97-1795.82]	-0.06 [-0.13 to 0.02]
Democratic Republic of the Congo	Male	742.38 [584.7-924.61]	2010.33 [1561.29-2493.14]	170.8	624.11 [486.16-772.41]	682.76 [529.75-842.29]	0.33 [0.28 to 0.39]
Denmark	Male	596.15 [470.25-734.91]	802.37 [629.58-997.81]	34.59	1904.82 [1502.08-2359.58]	1999.22 [1572.53-2472.2]	0.22 [0.14 to 0.3]
Djibouti	Male	6.23 [4.78-7.85]	22.29 [17.11-28.11]	257.78	396.36 [307.66-489.77]	421.13 [326.53-523.57]	0.23 [0.18 to 0.29]
Dominica	Male	3.9 [3.11-4.78]	6.07 [4.69-7.67]	55.64	1344.5 [1049.46-1667.05]	1449.62 [1130.35-1789.35]	0.29 [0.25 to 0.33]
Dominican Republic	Male	330.46 [261.9-409.54]	756.89 [580.03-948.13]	129.04	1334.77 [1055.8-1664.69]	1453.81 [1116.39-1816.69]	0.32 [0.28 to 0.35]
Ecuador	Male	741.82 [653.58-850.57]	2017.73 [1858.15-2181.83]	172	1994.89 [1776.86-2254.97]	2447.14 [2258.52-2640.66]	1.29 [1.05 to 1.53]
Egypt	Male	3038.48 [2376.16-3831.85]	6893.49 [5290.13-8691.14]	126.87	1386.36 [1076.72-1736.62]	1473.64 [1137.68-1839.84]	0.23 [0.19 to 0.26]

El Salvador	Male	156.72 [123.18-193.02]	234.01 [184.58-288.63]	49.32	875.11 [683.71-1083.2]	889.07 [699.27-1099.74]	0.07 [0.05 to 0.1]
Equatorial Guinea	Male	8.13 [6.47-10]	31.81 [24.85-40.54]	291.27	632.36 [497.8-782.63]	696.16 [546.16-863.78]	0.36 [0.3 to 0.42]
Eritrea	Male	34.49 [26.53-43.43]	95.79 [73.66-120.31]	177.73	394.3 [308.8-490.84]	417.29 [323.07-520.55]	0.22 [0.17 to 0.26]
Estonia	Male	529.89 [428.35-639.28]	443.19 [352.5-549.62]	-16.36	6613.07 [5440.89-7907.13]	5333.8 [4213.27-6563.31]	-0.94 [-1.07 to -0.81]
Eswatini	Male	20.67 [16.37-25.52]	40.48 [31.97-50.68]	95.84	873.33 [694.11-1075.57]	937.89 [741.96-1153.33]	0.26 [0.23 to 0.29]
Ethiopia	Male	710.09 [557.7-868.94]	1617.39 [1268.14-1999.9]	127.77	438.53 [342.13-540.77]	460.71 [360.16-570.77]	0.18 [0.1 to 0.26]
Fiji	Male	37.06 [28.72-46.97]	64.06 [49.11-80.53]	72.85	1286.36 [997.66-1612.87]	1384.04 [1067.55-1728.99]	0.22 [0.18 to 0.26]
Finland	Male	519.3 [396.94-658.09]	661.9 [508.04-840.52]	27.46	1763.35 [1352.71-2219.73]	1782.74 [1376.55-2231.2]	-0.73 [-1.42 to -0.04]
France	Male	6459.05 [5024.25-8134.85]	8396.55 [6432.77-10654.15]	30	1998.49 [1556.41-2507.66]	2017.86 [1537.63-2526.06]	0.03 [-0.01 to 0.07]
Gabon	Male	22.49 [17.82-27.7]	50.27 [39.05-62.3]	123.52	635.88 [498.25-783.48]	699.87 [543.8-869.25]	0.37 [0.32 to 0.43]
Gambia	Male	22.69 [18.05-27.72]	59.13 [46.84-72.33]	160.6	774.71 [606.89-944.85]	865.44 [679.88-1071.8]	0.47 [0.37 to 0.56]
Germany	Male	8996.62 [6868.15-11328.5]	11270.23 [8690.77-14304.54]	25.27	1844.25 [1426.26-2300.84]	1896.9 [1466.83-2363.71]	2 [1.35 to 2.66]
Ghana	Male	540.65 [411.71-820.01]	1491.16 [1133.03-2408.08]	175.81	1334.59 [1013.54-2172.91]	1608.78 [1213.79-2801.53]	0.59 [0.38 to 0.8]
Greece	Male	1235.85 [940.01-1556.84]	1423.07 [1088.66-1806.62]	15.15	2004.38 [1537.54-2514.79]	2016.92 [1548.66-2526.08]	0.05 [0.02 to 0.08]
Greenland	Male	3.08 [2.4-3.87]	4.28 [3.32-5.48]	38.96	1120.25 [880.06-1387.51]	1147.16 [899.05-1425.19]	0.1 [0.06 to 0.14]
Grenada	Male	5.01 [4.09-6.05]	14.08 [10.91-17.29]	181.04	1647.76 [1337.33-2020.81]	2335.42 [1831.58-2851.94]	1.41 [1.23 to 1.59]
Guam	Male	8.24 [6.36-10.49]	13.49 [10.22-17.12]	63.71	1322.55 [1019.15-1660]	1412.12 [1082.83-1782.25]	0.16 [0.13 to 0.2]
Guatemala	Male	233.26 [184.31-290.82]	604.54 [475.32-742.37]	159.17	897.72 [706.44-1109.92]	902.67 [709.97-1115.16]	0.01 [-0.01 to 0.02]
Guinea	Male	153.58 [121.42-186.75]	317.62 [252.73-386.3]	106.81	771.82 [604.8-952.22]	863.93 [677.82-1074.7]	0.45 [0.36 to 0.54]
Guinea-Bissau	Male	21.6 [17.19-26.65]	46.8 [36.91-57.31]	116.67	768.77 [604.23-954.54]	857.58 [672-1068.48]	0.47 [0.36 to 0.58]

Guyana	Male	38.27 [30.51-46.91]	65.94 [52.62-80.48]	72.3	1444.01 [1153.91-1766.74]	1809.8 [1459.01-2200.64]	0.82 [0.71 to 0.94]
Haiti	Male	294.29 [231.37-358.86]	689.98 [546.31-854.48]	134.46	1354.47 [1069.01-1652.26]	1483.84 [1183.43-1813.79]	0.39 [0.35 to 0.43]
Honduras	Male	159.5 [128.08-197.61]	415.46 [328.94-508.56]	160.48	1134.92 [887.94-1403.14]	1145.94 [899.11-1408.54]	-0.15 [-0.32 to 0.03]
Hungary	Male	1140.1 [910.54-1489.32]	1013.19 [827.24-1242.17]	-11.13	1922.82 [1553.89-2474.75]	1558.25 [1279.26-1878.89]	-0.95 [-1.03 to -0.86]
Iceland	Male	23.86 [18.65-29.91]	40.43 [31.21-50.37]	69.45	1838.76 [1431.19-2286.76]	1854.96 [1440.81-2293.61]	-0.82 [-1.12 to -0.51]
India	Male	70300.51 [55912.26-86741.73]	161790.35 [127590.96-201440.07]	130.14	2022.41 [1621.58-2474.04]	2332.16 [1847.34-2876.03]	0.61 [0.46 to 0.75]
Indonesia	Male	24562.3 [19168.19-30024.43]	26552.11 [21314.51-31557.12]	8.1	3874.34 [3039.47-4762.55]	1976.17 [1606.68-2353.61]	-3.11 [-3.56 to -2.66]
Iran (Islamic Republic of)	Male	3168.48 [2491.16-3928.66]	7741.29 [5985.77-9875.56]	144.32	1515.31 [1183.79-1892.69]	1623.77 [1270.67-2022.07]	0.26 [0.2 to 0.32]
Iraq	Male	844.29 [669.11-1056.35]	2732.31 [2136.05-3438.81]	223.62	1430.46 [1131.94-1785.56]	1480.45 [1158.09-1856.6]	0.07 [0.04 to 0.1]
Ireland	Male	359.05 [279.4-442.9]	605.71 [463.47-772.07]	68.7	2014.12 [1558.31-2486.19]	2003.23 [1548.2-2529.47]	-0.01 [-0.05 to 0.03]
Israel	Male	436.8 [338.45-545.63]	1000.97 [783.71-1240.74]	129.16	1994.57 [1538.03-2503.51]	2093.33 [1632.32-2607.03]	0.25 [0.22 to 0.27]
Italy	Male	8425.31 [6574.87-10487.34]	9431.24 [7626.1-11595.56]	11.94	2456.83 [1921.29-3051.31]	2165.19 [1738.69-2610.8]	-0.63 [-0.74 to -0.52]
Jamaica	Male	118.07 [92.33-145.55]	236.36 [187.42-292.87]	100.19	1345.89 [1041.27-1678.79]	1598.31 [1272.35-1972.19]	0.73 [0.68 to 0.77]
Japan	Male	17851.27 [13491.89-22665.72]	19250.66 [15651.65-23316.18]	7.84	2330.44 [1788.45-2940.57]	2204.88 [1769.15-2662.37]	-0.34 [-0.43 to -0.25]
Jordan	Male	176.82 [138.77-221.74]	1229.73 [897.22-1768.64]	595.47	1378.67 [1067.56-1717.52]	2129.95 [1593.45-3060.15]	2.03 [1.67 to 2.4]
Kazakhstan	Male	1997.73 [1548.43-2554.7]	2426.96	21.49	3070.57 [2361.1-4041.39]	2818.23	-0.74 [-0.9 to

			[1934.32-2963.96]			[2275.28-3400.66]	-0.57]
Kenya	Male	297.97 [232.18-370.52]	839 [653.17-1041.87]	181.57	441.92 [345.19-547.57]	464.25 [364.42-575.02]	1.21 [0.67 to 1.75]
Kuwait	Male	136.62 [102.79-181.57]	446.04 [336.28-585.83]	226.48	1391.68 [1075.9-1746.4]	1489.35 [1156.33-1860.42]	0.26 [0.23 to 0.28]
Kyrgyzstan	Male	468.52 [375.41-578.56]	760.1 [604.79-939.47]	62.23	2752.72 [2218.06-3374.72]	2624.82 [2094.45-3224.12]	0.87 [0.45 to 1.3]
Lao People's Democratic Republic	Male	301.17 [244.28-361.59]	659.17 [517.93-815.62]	118.87	2373.87 [1927.48-2862.75]	2257.6 [1807.59-2752.12]	-0.35 [-0.42 to -0.28]
Latvia	Male	968.78 [786.3-1172.73]	779.82 [644.31-934.99]	-19.5	7075.34 [5821.34-8467.45]	6222.38 [5174.1-7425.43]	-1.12 [-1.42 to -0.81]
Lebanon	Male	185.64 [144-231.92]	376.83 [289.5-476.85]	102.99	1389.81 [1074.67-1751.57]	1484.75 [1140.14-1864.95]	0.25 [0.23 to 0.28]
Lesotho	Male	55.8 [44.18-67.96]	82.37 [64.46-103.18]	47.62	860.67 [681.58-1044.69]	916.06 [727.14-1109.39]	0.24 [0.19 to 0.29]
Liberia	Male	52.82 [41.6-64.83]	140.49 [109.78-171.87]	165.98	772.59 [601.44-953.33]	867.05 [675.99-1084.06]	0.5 [0.41 to 0.59]
Libya	Male	221.15 [172.18-278.67]	575.31 [440.89-746.75]	160.14	1391.24 [1079.17-1740.22]	1471.76 [1143.37-1841.27]	0.23 [0.21 to 0.26]
Lithuania	Male	1402.59 [1157.01-1659.53]	953.31 [746.17-1189.31]	-32.03	7632.69 [6354.28-9037.01]	5409.42 [4264.12-6652.28]	-1.58 [-1.8 to -1.36]
Luxembourg	Male	51.07 [39.14-63.91]	94.4 [72.48-120.12]	84.84	2209.36 [1698.96-2748.67]	2252.55 [1741.8-2850.75]	0.01 [-0.68 to 0.71]
Madagascar	Male	149.34 [116.95-184.41]	381.32 [297.26-473.43]	155.34	396.23 [305.6-489.06]	418.47 [323.04-519.22]	0.2 [0.15 to 0.24]
Malawi	Male	114.81 [89.87-143.31]	236.41 [183.77-293.56]	105.91	396.53 [303.42-491.49]	420.09 [327.53-520.65]	0.22 [0.17 to 0.27]
Malaysia	Male	1279.99 [978.29-1611.52]	3479.75 [2657.26-4348.14]	171.86	2022.58 [1540.7-2557.18]	2161.34 [1650.88-2696.92]	0.25 [0.18 to 0.32]
Maldives	Male	13.91 [10.64-17.27]	64.75 [49.16-83.58]	365.49	2020.99 [1550.55-2527.23]	2198.9 [1677.51-2771.7]	0.34 [0.27 to 0.4]
Mali	Male	207.59 [164.18-254.05]	537.07 [424.77-650.94]	158.72	770.74 [606.46-949.1]	869.3 [677.42-1078.21]	0.48 [0.4 to 0.56]
Malta	Male	45.34 [35.09-56.23]	70.61 [54.78-88.07]	55.73	2253.52 [1746.8-2785.38]	2276.89 [1760.31-2821.78]	1.85 [1.13 to 2.57]
Marshall Islands	Male	1.75 [1.37-2.21]	3.66 [2.85-4.6]	109.14	1345.69 [1057.5-1668.73]	1421.72 [1106.23-1762.71]	0.14 [0.09 to 0.19]
Mauritania	Male	49.67 [39.25-60.68]	117.06 [92.5-143.79]	135.68	774.01 [602.46-948.81]	878.45 [682.65-1098.99]	0.51 [0.42 to 0.6]

Mauritius	Male	93.76 [72.8-117.17]	183.35 [138.79-236.81]	95.55	2087.6 [1622.31-2632.19]	2199.43 [1681.53-2793.84]	0.17 [0.13 to 0.21]
Mexico	Male	4218.06 [3378.46-5167.24]	9277.93 [7457.89-11170.37]	119.96	1527.18 [1214.38-1884.1]	1512.62 [1224.04-1819.14]	-0.14 [-0.8 to 0.53]
Micronesia (Federated States of)	Male	4.38 [3.42-5.47]	6.6 [5.08-8.24]	50.68	1305.2 [1018.04-1620.48]	1405.03 [1086.83-1753.56]	0.22 [0.18 to 0.25]
Mongolia	Male	157.84 [123.64-197.43]	356.06 [276.4-450.03]	125.58	2066.45 [1615.82-2542.13]	2245.9 [1767.49-2801.8]	0.37 [0.3 to 0.44]
Montenegro	Male	40.73 [32.17-49.98]	50.57 [40.07-62.91]	24.16	1309.63 [1036.84-1593.25]	1313.73 [1052.98-1613.82]	0.03 [0 to 0.06]
Morocco	Male	1329.22 [1034.99-1661.24]	2767.49 [2122.73-3480.58]	108.2	1384.55 [1069.86-1743.32]	1468.52 [1133.53-1834.46]	0.22 [0.18 to 0.26]
Mozambique	Male	161.13 [125.81-198.77]	350.57 [273.75-429.96]	117.57	394.84 [305.57-492.34]	419.87 [328.17-521.84]	0.22 [0.17 to 0.26]
Myanmar	Male	3557.75 [2886.82-4268.71]	6167.14 [4922.59-7499.4]	73.34	2557.72 [2076.72-3063.82]	2480.76 [2000-2981.5]	-0.3 [-0.4 to -0.2]
Namibia	Male	42.14 [33.35-51.43]	86.52 [67.58-108.58]	105.32	858.62 [676.42-1054.9]	905.94 [714.13-1117.22]	0.2 [0.13 to 0.26]
Nepal	Male	1160.16 [913.62-1462.22]	2391.01 [1874.49-2923.29]	106.09	1649.27 [1307.89-2057.82]	1905.49 [1489.57-2348.09]	0.5 [0.42 to 0.58]
Netherlands	Male	1929.66 [1514.43-2393.4]	2490.41 [1943.24-3137.75]	29.06	2244.46 [1759.84-2777.57]	2110.67 [1641.91-2631.74]	-0.27 [-0.3 to -0.23]
New Zealand	Male	419.4 [329.37-519.73]	527.39 [442.85-628.74]	25.75	2345.12 [1831.02-2918.14]	1861.06 [1558.96-2189.72]	-0.8 [-0.98 to -0.62]
Nicaragua	Male	100.44 [78.97-124.75]	249.59 [193.91-313.04]	148.5	879.75 [691.34-1095.58]	897.23 [699.82-1109.31]	0.1 [0.07 to 0.14]
Niger	Male	174.54 [137.23-212.52]	501.73 [400.66-608.56]	187.46	771.88 [608.6-953.83]	868.36 [680.29-1081.36]	0.49 [0.4 to 0.59]
Nigeria	Male	2735.49 [2156.43-3345.39]	5874.6 [4647.08-7184.31]	114.75	836.41 [662.53-1028.15]	936.76 [738.24-1166.4]	0.47 [0.35 to 0.59]
North Macedonia	Male	131.72 [105.12-162.07]	184.96 [146.45-231.76]	40.42	1301.66 [1043.33-1587.11]	1313.3 [1059.05-1601.11]	0.06 [0.03 to 0.09]
Northern Mariana Islands	Male	3.01 [2.3-3.92]	4.28 [3.14-5.51]	42.19	1317.73 [1013.98-1643.8]	1418.31 [1099.09-1788.36]	0.21 [0.18 to 0.24]
Norway	Male	1006.54 [790.66-1236.96]	1339.04 [1047.11-1672.52]	33.03	3974.31 [3141.45-4893.36]	3767.28 [2956.74-4682.85]	0.71 [0.42 to 0.99]

Oman	Male	129.69 [99.03-170.88]	492.14 [372.55-664.23]	279.47	1384.16 [1073.8-1748.28]	1486.8 [1159.07-1867.6]	0.27 [0.23 to 0.3]
Pakistan	Male	7188.87 [5692.66-8830.67]	17029.08 [13395.98-21214.05]	136.88	1743.99 [1368.28-2151.84]	1945.69 [1520.82-2410.92]	0.37 [0.28 to 0.46]
Palestine	Male	83.73 [65.54-105.81]	290.94 [225.8-367.38]	247.47	1385.49 [1062.11-1744.53]	1473.97 [1147.41-1861.24]	0.25 [0.22 to 0.28]
Panama	Male	86.2 [67.79-106.9]	187.75 [145.27-233.67]	117.81	888.42 [701.16-1095.33]	895.04 [695.57-1116.57]	0.03 [0 to 0.06]
Papua New Guinea	Male	187.67 [144.83-236.17]	540.73 [415.61-688.86]	188.13	1279.75 [983.97-1608.4]	1375.98 [1058.82-1732.76]	0.22 [0.16 to 0.28]
Paraguay	Male	161.91 [127.12-199.99]	372.16 [292.54-459.93]	129.86	1125.57 [889.37-1394.05]	1144.44 [897.65-1413.62]	0.05 [0.03 to 0.08]
Peru	Male	1702.88 [1315.5-2180.99]	3690.89 [2824.03-4696.51]	116.74	2115.83 [1645.42-2703.8]	2179.48 [1668.96-2763.23]	0.12 [0.08 to 0.17]
Philippines	Male	7861.81 [6331.37-9415.16]	21018.55 [16824.13-25312.47]	167.35	4055.71 [3263.37-4878.8]	4404.77 [3570.61-5280.97]	0.23 [0.06 to 0.41]
Poland	Male	4839.95 [3622.33-6260.82]	2083.97 [1802.01-2425.08]	-56.94	2466.04 [1870.54-3142.1]	850.7 [741.93-973.08]	-4.62 [-5.37 to -3.87]
Portugal	Male	979.41 [763.92-1234.73]	1219.3 [1064.63-1455.04]	24.49	1757.87 [1367.57-2208.52]	1679.15 [1463.71-1987.35]	-0.18 [-0.23 to -0.12]
Puerto Rico	Male	230.39 [178.01-286.16]	332.83 [256.66-425.64]	44.46	1369.7 [1058.51-1712.45]	1482.02 [1142.39-1866.75]	0.31 [0.28 to 0.34]
Qatar	Male	45.48 [33.68-62]	397.84 [300.46-527.71]	774.76	1383.72 [1078.08-1737.59]	1485.67 [1157.59-1857.15]	0.27 [0.24 to 0.29]
Republic of Korea	Male	4332.01 [3321.92-5537.72]	8213.44 [6227.93-10423.48]	89.6	2114.39 [1640.29-2669.56]	2117.61 [1630.82-2667.19]	-0.02 [-0.05 to 0]
Republic of Moldova	Male	1101.68 [878.7-1352.09]	1246.04 [973.13-1568.83]	13.1	5235.88 [4199.41-6388.3]	5349.63 [4155.18-6688.99]	0.22 [0.14 to 0.3]
Romania	Male	1638.4 [1297.69-2036.35]	1635.48 [1272.51-2037.52]	-0.18	1306.69 [1036.03-1612.39]	1308.14 [1046.2-1593.82]	2.19 [1.46 to 2.94]
Russian Federation	Male	59911.61 [48215.77-72667.72]	58056.67 [46373.68-70904.7]	-3.1	7914.58 [6420.42-9551.79]	6529.33 [5252.51-7937.54]	-0.81 [-1 to -0.62]
Rwanda	Male	82.9 [65-103.56]	181.56 [142.76-225.47]	119.01	395.74 [308.61-495.62]	418.36 [325.96-522.05]	0.22 [0.17 to 0.27]

Saint Lucia	Male	6.29 [4.95-7.67]	15.48 [11.82-19.6]	146.1	1341.12 [1037.13-1664.22]	1455.87 [1128.89-1821.1]	0.33 [0.29 to 0.37]
Saint Vincent and the Grenadines	Male	5.71 [4.55-6.95]	12.35 [9.7-15.16]	116.29	1519.81 [1208.62-1873.74]	1777.56 [1413.25-2178.41]	0.65 [0.58 to 0.71]
Samoa	Male	7.42 [5.78-9.19]	12.76 [9.79-15.98]	71.97	1319.8 [1023.06-1657.58]	1408.06 [1084.25-1754.76]	0.17 [0.13 to 0.21]
Sao Tome and Principe	Male	2.91 [2.31-3.56]	6.52 [5.13-7.99]	124.05	776.12 [614.6-959.1]	880.24 [691.06-1102.05]	0.48 [0.39 to 0.56]
Saudi Arabia	Male	981.07 [755.86-1266.58]	3702.57 [2815.85-4902.21]	277.4	1387.63 [1075.15-1744.13]	1484.43 [1156.91-1871.42]	0.26 [0.22 to 0.29]
Senegal	Male	171.51 [135.86-209.73]	429.28 [337.72-524.02]	150.29	773.54 [608.27-960.52]	869.39 [682.16-1079.44]	0.5 [0.41 to 0.59]
Serbia	Male	627.58 [493.41-779.77]	677.53 [529.78-839.68]	7.96	1182.37 [942.22-1448.35]	1203.75 [949.57-1469.26]	1.98 [1.34 to 2.62]
Seychelles	Male	6.04 [4.8-7.54]	15.05 [11.81-18.8]	149.17	2125.8 [1683.94-2659.29]	2342.08 [1884.09-2879.28]	0.29 [0.2 to 0.38]
Sierra Leone	Male	93.33 [74.43-113.49]	228.47 [180.77-278.27]	144.8	771.04 [607.04-943.04]	865.8 [688.51-1077.33]	0.47 [0.38 to 0.57]
Singapore	Male	338.97 [263.67-433.64]	884.79 [665.13-1122.57]	161.02	2230.73 [1753.44-2810.5]	2122.55 [1610.47-2673.6]	-0.21 [-0.29 to -0.13]
Slovakia	Male	403.86 [324.13-489.17]	510.79 [394.89-636.41]	26.48	1511.98 [1215.13-1828.56]	1466.77 [1160.67-1795.33]	0.62 [-0.57 to 1.82]
Slovenia	Male	139.08 [108.87-170.58]	184.8 [142.28-232.85]	32.87	1321.49 [1046.41-1609.5]	1298.16 [1026.59-1590.89]	1.1 [0.33 to 1.89]
Solomon Islands	Male	14.33 [11.21-17.96]	34.34 [26.72-43.06]	139.64	1321.11 [1033.2-1641.79]	1404.15 [1097.8-1746.46]	0.14 [0.1 to 0.18]
Somalia	Male	87.21 [67.77-109.08]	248.45 [192.53-310.81]	184.89	394.87 [304.45-490.61]	417.69 [324.14-518.49]	0.2 [0.14 to 0.26]
South Africa	Male	1382.86 [1085.6-1729.07]	2708.77 [2131.84-3354.25]	95.88	965.41 [758.62-1188.23]	1005.51 [793.24-1236.11]	0.1 [0.08 to 0.13]
South Sudan	Male	79.42 [62.02-97.77]	124.12 [97.21-152.16]	56.28	394.46 [302.82-488.17]	417.49 [323.89-514.7]	0.21 [0.16 to 0.25]
Spain	Male	4299.92 [3353.98-5370.5]	6498.69 [5021.33-8218.1]	51.14	1991.33 [1557.8-2501.63]	2031.23 [1584.82-2541.42]	0.08 [0.05 to 0.12]
Sri Lanka	Male	1460.3 [1115.69-1822.52]	2662.9 [2050.73-3350.64]	82.35	2084.73 [1594.74-2609.87]	2208.56 [1718.28-2764.78]	0.21 [0.15 to 0.27]

Sudan	Male	937.42 [737.36-1169.75]	2253.68 [1743.72-2847.11]	140.41	1374.04 [1068.27-1720.69]	1460.33 [1122.94-1823.81]	0.24 [0.2 to 0.28]
Suriname	Male	24.87 [19.9-30.31]	67.75 [53.5-82.64]	172.42	1620.82 [1303.06-1971.52]	2182.59 [1752.48-2630.05]	1.17 [0.99 to 1.35]
Sweden	Male	1244.22 [966.42-1528.38]	1348.93 [1072.73-1676.72]	8.42	2276.77 [1778.95-2797.55]	1912.03 [1513.95-2360]	-0.25 [-0.57 to 0.07]
Switzerland	Male	902.1 [698.19-1128.16]	1333.36 [1016.72-1674.19]	47.81	2176.14 [1685.68-2716.77]	2207.89 [1696.42-2757.52]	0.26 [-0.19 to 0.71]
Syrian Arab Republic	Male	577.23 [453.99-715.51]	1049.96 [813.26-1324.66]	81.9	1383.5 [1073.73-1733.02]	1483.53 [1154.2-1871.3]	0.26 [0.24 to 0.29]
Tajikistan	Male	410.27 [325.82-507.29]	951.16 [757.84-1178.93]	131.84	2188.18 [1733.35-2666.34]	2354.46 [1912.31-2863.01]	0.27 [0.23 to 0.31]
Thailand	Male	6893.39 [5503.27-8286.98]	12581.26 [10099.09-15569.36]	82.51	3195.47 [2569.44-3837.08]	2648.98 [2147.77-3238.21]	-1.07 [-1.3 to -0.84]
Timor-Leste	Male	50.06 [39.26-63.08]	101.82 [80.92-125.06]	103.4	2030.71 [1626.79-2503.4]	2126.77 [1686.59-2624.82]	0.15 [0.08 to 0.21]
Togo	Male	73.85 [58.99-90.01]	217.54 [171.19-266.93]	194.57	777.57 [614.7-956.58]	867.65 [686.21-1074.11]	0.46 [0.37 to 0.56]
Tonga	Male	4.29 [3.37-5.31]	5.98 [4.62-7.49]	39.39	1321.3 [1036.84-1646.91]	1430.03 [1109.51-1792.22]	0.22 [0.19 to 0.24]
Trinidad and Tobago	Male	86.36 [67.1-106.45]	245.89 [181.41-457.17]	184.73	1819.44 [1389.83-2273.14]	2678.05 [1988.37-4846.25]	1.74 [1.47 to 2]
Tunisia	Male	461.53 [361.09-572.06]	974.08 [757.33-1230.66]	111.05	1387.75 [1071.49-1733.82]	1487.67 [1169.11-1863.54]	0.23 [0.2 to 0.26]
Turkey	Male	3419.44 [2664.51-4320.1]	7202.69 [5632.65-9166.99]	110.64	1385.89 [1081.15-1739.01]	1519.8 [1197.1-1908.9]	0.37 [0.35 to 0.38]
Turkmenistan	Male	275.56 [216.2-344.14]	547.42 [427.93-680.43]	98.66	2089.44 [1650.25-2562.53]	2210.3 [1746.41-2741.09]	0.24 [0.15 to 0.34]
Uganda	Male	195.3 [153.85-242.56]	476.17 [369.78-594.36]	143.81	396.81 [312.12-493.19]	420.11 [324.99-520.99]	0.21 [0.16 to 0.26]
Ukraine	Male	21401.1 [17241.5-26093.41]	16463.44 [13046.55-20330.32]	-23.07	7481.98 [6030.25-9035.25]	6037.09 [4775.28-7424.73]	-1.04 [-1.21 to -0.87]
United Arab Emirates	Male	177.96 [132.13-241.82]	1568.47 [1147.17-2130.92]	781.36	1378.71 [1065.5-1728.85]	1472.88 [1150.29-1852.21]	0.25 [0.22 to 0.28]

United Kingdom	Male	8088.72 [6301.53-10087.74]	11295.18 [9151.93-13716.03]	39.64	2417.64 [1886.14-2999.31]	2560.78 [2065.1-3103.02]	0.66 [0.47 to 0.86]
United Republic of Tanzania	Male	305.49 [239.62-376.33]	756.3 [590.4-938.89]	147.57	397.17 [306.19-490.61]	419.57 [326.97-521.1]	0.2 [0.15 to 0.24]
United States of America	Male	29682.22 [23117.99-36932.76]	24473.55 [21082.75-28888.51]	-17.55	2212.61 [1707.08-2764.22]	1130.88 [975.29-1308.77]	-2.47 [-2.85 to -2.09]
United States Virgin	Male	7.41 [5.83-9.21]	15.85 [12.08-19.92]	113.9	1508.27 [1202.09-1844.67]	2205.85 [1701.97-2698.64]	1.71 [1.43 to 1.98]
Uruguay	Male	332.96 [256.39-417.7]	409.77 [316.97-513.93]	23.07	2128.43 [1624.4-2696.2]	2196.3 [1712.6-2765.34]	0.14 [0.11 to 0.17]
Uzbekistan	Male	1591.63 [1250.06-1978.32]	3423.18 [2680.96-4256.47]	115.07	2151.76 [1667.74-2660.53]	2259.97 [1791.54-2798.27]	0.2 [0.13 to 0.26]
Vanuatu	Male	6.52 [5.06-8.23]	15.93 [12.2-19.95]	144.33	1274.55 [985.4-1583.55]	1376.67 [1047.99-1724.45]	0.24 [0.19 to 0.29]
Venezuela (Bolivarian Republic of)	Male	692.04 [551.88-853.71]	1570.93 [1247.31-1927.3]	127	1027.23 [817.92-1251.43]	1066.51 [856.45-1299.98]	0.01 [-0.12 to 0.13]
Viet Nam	Male	4863.48 [3828.16-5966.66]	17066.18 [13048.21-22406.64]	250.9	2286.8 [1806.46-2832.16]	3224.46 [2501.92-4193.65]	1.52 [1.24 to 1.81]
Yemen	Male	568.32 [447.5-710.05]	1694.4 [1321.27-2144.74]	198.14	1372.94 [1063.97-1727.13]	1466.73 [1142.35-1833.02]	0.24 [0.2 to 0.28]
Zambia	Male	91.5 [70.78-113.15]	244.6 [190.67-306.1]	167.32	396.86 [306.33-492.64]	419.31 [326.87-519.59]	0.21 [0.17 to 0.26]
Zimbabwe	Male	308.65 [247.02-381.27]	518.31 [410.99-642.84]	67.93	918.86 [734.95-1122.21]	974.78 [776.57-1189.89]	0.15 [0.09 to 0.2]
Afghanistan	Female	377.72 [292.63-467.36]	1085.25 [849.71-1329.42]	187.32	872.19 [672.48-1088.88]	1184.27 [927.24-1467.36]	0.19 [0.12 to 0.25]
Albania	Female	179.5 [142.34-221.17]	211.31 [167.87-261.83]	17.72	1256.78 [996.09-1536.16]	1284.26 [1027.1-1572.4]	0.07 [0.03 to 0.1]
Algeria	Female	774.26 [608.91-953.94]	1976.14 [1517.69-2472.73]	155.23	891.32 [692.53-1117.28]	1214.32 [938.28-1510.07]	0.2 [0.16 to 0.24]
American Samoa	Female	1.1 [0.86-1.35]	1.78 [1.36-2.25]	61.82	668.13 [520.84-822.29]	1038.32 [802.58-1300.76]	-0.21 [-0.3 to -0.12]
Andorra	Female	2.25 [1.75-2.83]	4.37 [3.3-5.5]	94.22	765.37 [598.66-950.9]	1417.66 [1105.13-1764.58]	0.05 [0.03 to 0.07]
Angola	Female	160.97 [125.2-201.89]	530.84 [411.83-662.33]	229.78	449.48 [348.15-560.73]	574.59 [446.56-710.03]	0.28 [0.21 to 0.34]

Antigua and Barbuda	Female	1.96 [1.55-2.45]	4.45 [3.5-5.56]	127.04	707.62 [549.79-884.92]	1120.5 [878.39-1394.75]	0.5 [0.47 to 0.54]
Argentina	Female	1970.11 [1516.44-2453.97]	3018.8 [2315.64-3833.46]	53.23	1198.35 [923.08-1499.81]	1664.07 [1287.41-2095.93]	0.03 [0 to 0.06]
Armenia	Female	213.14 [172.63-258.5]	331.97 [249.8-478.09]	55.75	1251.8 [1021.09-1508.7]	2540.63 [1939.79-3808.42]	1.06 [0.95 to 1.16]
Australia	Female	689.44 [539.85-863.94]	1098.14 [848.26-1362.79]	59.28	735.82 [574.67-920.44]	1282.28 [986.27-1592.12]	-0.11 [-0.15 to -0.07]
Austria	Female	913.58 [715.83-1063.22]	768.75 [565.1-1066.91]	-15.85	1783.54 [1413.15-2054.89]	2409.47 [1729.79-3299.85]	-1.05 [-1.24 to -0.86]
Azerbaijan	Female	376.28 [296.45-464.81]	695.4 [540.26-867.68]	84.81	1101.33 [869.16-1354.47]	1670.51 [1320.01-2064.77]	0.21 [0.14 to 0.27]
Bahamas	Female	8.04 [6.26-10.18]	17.25 [13.32-21.77]	114.55	702.2 [542.58-874.5]	1110.49 [867.16-1373.13]	0.29 [0.27 to 0.31]
Bahrain	Female	13.77 [10.75-17.36]	60.5 [46.62-77.17]	339.36	891.2 [685.2-1103.77]	1292.07 [1004.2-1626.35]	0.23 [0.19 to 0.27]
Bangladesh	Female	3713.22 [2886.33-4757.03]	8562.65 [6581.35-10894.76]	130.6	960.26 [747.09-1221.31]	1484.37 [1161.51-1837.85]	0.33 [0.27 to 0.39]
Barbados	Female	9.19 [7.26-11.35]	15.25 [11.82-19]	65.94	705.73 [550.43-877.49]	1196 [946.51-1469.55]	0.32 [0.29 to 0.34]
Belarus	Female	1932.75 [1572.89-2343.28]	1946.8 [1538.27-2464.96]	0.73	2801.86 [2289.62-3385.24]	4017.99 [3181.04-4977.84]	-0.21 [-0.34 to -0.07]
Belgium	Female	494.63 [385.02-612.02]	630.2 [492.99-785.02]	27.41	808.93 [629.74-1005.94]	1545.25 [1189.04-1909.61]	1.54 [0.83 to 2.25]
Belize	Female	4.27 [3.36-5.27]	15.07 [11.8-18.88]	252.93	707.37 [554.59-879.06]	1195.09 [938.73-1476.8]	0.47 [0.39 to 0.54]
Benin	Female	80.79 [62.97-101.09]	223.64 [175.38-276.46]	176.82	494 [387.93-611.61]	677.31 [530.21-834.42]	0.16 [0.11 to 0.21]
Bermuda	Female	2.53 [1.96-3.15]	3.57 [2.77-4.48]	41.11	721.66 [559.65-892.76]	1122.78 [873.55-1400.88]	0.25 [0.2 to 0.29]
Bhutan	Female	21.15 [16.53-27.3]	38.42 [30.15-49.13]	81.65	961.19 [753.2-1212.67]	1504.04 [1184.36-1864.77]	0.36 [0.31 to 0.41]
Bolivia (Plurinational State of)	Female	271.63 [208.91-344.6]	653.35 [505.21-829.34]	140.53	1131.48 [870.76-1432.59]	1661.89 [1284.24-2086.54]	0.21 [0.15 to 0.27]
Bosnia and Herzegovina	Female	298.39 [236.21-366.71]	275.56 [218.17-345.11]	-7.65	1221.14 [971.6-1488.44]	1270.68	0.17 [0.13 to 0.21]

						[1012.5-1552.54]	
Botswana	Female	21.81 [17.07-27.16]	52.8 [40.63-66.31]	142.09	445.97 [346.3-552.76]	671.78 [520.67-827.97]	-0.23 [-0.37 to -0.08]
Brazil	Female	5517.05 [4428.41-6805.03]	10317.03 [8342.06-12484.82]	87	854.8 [685.09-1037.74]	969.89 [790.08-1165.75]	-0.29 [-0.37 to -0.21]
Brunei Darussalam	Female	6 [4.77-7.38]	16.87 [13.21-21.24]	181.17	761.48 [600.84-939.02]	1496.08 [1168.31-1849.76]	0.09 [0.04 to 0.15]
Bulgaria	Female	858.86 [675.01-1058.64]	581.28 [456.8-727.67]	-32.32	1548.44 [1233.59-1880.22]	1267.09 [1013.2-1537.38]	-0.82 [-1.04 to -0.59]
Burkina Faso	Female	170.93 [133.4-211.14]	431.01 [334.11-528.87]	152.16	506.51 [395.36-628.26]	686.69 [539.8-844.24]	0.21 [0.18 to 0.24]
Burundi	Female	114.75 [91.31-142.05]	241.36 [188.84-299.54]	110.34	621.5 [490.21-763.86]	525.01 [408.44-646.9]	0.11 [0.07 to 0.14]
Cabo Verde	Female	6.54 [5.19-8.06]	13.91 [10.83-17.3]	112.69	489.73 [379.77-614.12]	686.98 [540-847.34]	0.26 [0.21 to 0.31]
Cambodia	Female	205.34 [162.32-252.74]	494.29 [385.89-614.51]	140.72	567.63 [447.56-694.57]	1496.36 [1193.87-1823.68]	0.19 [0.15 to 0.23]
Cameroon	Female	183.63 [143.44-227.79]	547.24 [425.77-678.29]	198.01	508.8 [399.17-625.25]	685.95 [536.64-844.09]	0.01 [-0.03 to 0.06]
Canada	Female	1360.31 [1072.07-1686.93]	2069.5 [1633.51-2558.84]	52.13	861.11 [681.5-1060.69]	1057.58 [842.25-1298.52]	0.11 [0.08 to 0.15]
Central African Republic	Female	46.14 [35.62-57.59]	96.48 [75.46-120.05]	109.1	448.43 [347.32-561.27]	569.1 [439.49-699.28]	0.24 [0.17 to 0.3]
Chad	Female	102.16 [80.94-127.03]	252.31 [197.57-314.27]	146.98	490 [386.42-607.97]	697.84 [545.71-858.78]	0.12 [0.06 to 0.19]
Chile	Female	770.49 [601.11-983.93]	1317.17 [1009.2-1669.32]	70.95	1199.37 [934.06-1531.81]	1697.13 [1313.63-2162.36]	-0.96 [-1.2 to -0.73]
China	Female	42003.98 [32700.85-51718.89]	54847.16 [43673.79-68030.56]	30.58	833.13 [646.75-1032.79]	889.9 [717.04-1075.44]	-2.02 [-2.24 to -1.8]
Colombia	Female	759.33 [593.36-944.79]	1726.4 [1318.44-2181.09]	127.36	606.93 [467.14-762.6]	767.44 [597.06-949.94]	0.17 [0.12 to 0.21]
Comoros	Female	9.78 [7.73-11.91]	20.99 [16.57-25.82]	114.62	626.06 [491.82-773.48]	541.33 [422.81-665.08]	0.24 [0.21 to 0.28]
Congo	Female	40.22 [31.26-49.33]	108.04 [83.32-135.37]	168.62	451.77 [350.3-561.19]	581.49 [456.32-722.66]	0.28 [0.22 to 0.35]
Costa Rica	Female	69.87 [54.07-87.49]	169.31 [128.1-214.84]	142.32	612.5 [469.78-772.04]	749.51 [583.25-927.12]	0.03 [0 to 0.05]
Cote d'Ivoire	Female	189.89 [149.61-238.03]	478.97 [372.98-607.3]	152.24	498.1 [396.12-613.71]	696.7 [546.19-858.21]	0.12 [0.08 to 0.16]
Croatia	Female	350.16 [278.06-430.29]	353.9 [306.66-402.08]	1.07	1176.23 [938.39-1438.65]	1411.12	-0.18 [-0.39 to

						[1182.58-1629.74]	0.03]
Cuba	Female	398.85 [310.62-493.59]	657.77 [511.42-838.5]	64.92	738.48 [576.7-909.21]	1386.08 [1104.01-1723.71]	0.56 [0.5 to 0.62]
Cyprus	Female	27.72 [21.63-34.44]	59.69 [45.92-74.77]	115.33	667.63 [520.78-829.46]	1210.05 [946.15-1518.88]	-1.02 [-1.66 to -0.38]
Czechia	Female	1008.3 [809.02-1241.25]	877.49 [697.01-1093.43]	-12.97	1572.72 [1256.6-1897.21]	1332.17 [1060.66-1621.44]	-0.31 [-0.46 to -0.16]
Democratic People's Republic of Korea	Female	652.87 [502.76-822.41]	1038.68 [786.58-1318.19]	59.09	598.21 [466.8-747.57]	1015.16 [797.59-1264.19]	0.09 [0.04 to 0.14]
Democratic Republic of the Congo	Female	623.88 [487.98-778.81]	1528.42 [1181.68-1898.52]	144.99	451.15 [353.39-563.15]	575.14 [446.78-708.7]	0.25 [0.19 to 0.31]
Denmark	Female	225.48 [175.88-277.04]	267.8 [209.05-332.29]	18.77	707.06 [548.45-879.85]	1344.58 [1057.84-1665.52]	0.1 [-0.17 to 0.36]
Djibouti	Female	8.77 [6.88-10.86]	33.42 [25.85-42.16]	281.07	643.18 [505.8-792.97]	550.04 [429.48-677.65]	0.36 [0.32 to 0.4]
Dominica	Female	2.2 [1.74-2.72]	2.77 [2.12-3.47]	25.91	693.82 [539.46-865.98]	1103.14 [856.57-1367.7]	0.21 [0.18 to 0.24]
Dominican Republic	Female	191.41 [148.68-239.19]	390.21 [302.71-489.76]	103.86	691 [532.77-869.08]	1089.59 [840.64-1363.63]	0.22 [0.19 to 0.24]
Ecuador	Female	432.12 [348.94-527.96]	1400.9 [1293-1515.34]	224.19	1134.62 [910.06-1378.47]	2023 [1868.57-2177.03]	1.8 [1.58 to 2.01]
Egypt	Female	1855.34 [1451.87-2294.12]	3975.57 [3082.26-4943.37]	114.28	895.89 [697.26-1116.09]	1218.21 [950.84-1508.08]	0.18 [0.14 to 0.22]
El Salvador	Female	117.65 [91.08-146.84]	205.76 [157.86-258.57]	74.89	608.02 [462.03-775.84]	734.69 [576.84-911.72]	0.05 [0.01 to 0.09]
Equatorial Guinea	Female	7.36 [5.8-9.22]	24.08 [18.72-30.39]	227.17	447.78 [351.45-555.32]	577.55 [451.96-717.34]	0.38 [0.32 to 0.44]
Eritrea	Female	60.26 [47.51-74.29]	160.74 [126.57-197.35]	166.74	616.7 [485.01-763.83]	543.6 [427.58-670.23]	0.31 [0.27 to 0.34]
Estonia	Female	303.92 [251.49-361.76]	258.45 [202.05-331.78]	-14.96	2827.34 [2325.94-3408.37]	3939.19 [3090.59-4861.57]	-0.22 [-0.34 to -0.1]
Eswatini	Female	12.73 [9.99-15.85]	22.6 [17.75-28.59]	77.53	446.14 [347.71-554.16]	673.04 [531.21-827.5]	0.14 [0.07 to 0.2]
Ethiopia	Female	1266.68 [999.43-1566.73]	2629.96 [2076.73-3282.74]	107.63	788.21 [616.57-978.6]	601.15 [472.33-737.84]	-0.28 [-0.31 to -0.24]
Fiji	Female	17.72 [13.55-22.5]	29.93 [22.93-37.49]	68.91	618.43 [473.42-777.55]	1019.49 [784.61-1280.35]	0.2 [0.14 to 0.26]
Finland	Female	212.76 [164.31-265.28]	241.09 [185.31-303.81]	13.32	676.43 [524.87-847.73]	1226.94 [949.83-1534.6]	-0.46 [-1.41 to

							0.49]
France	Female	2558.6 [2009.19-3188.59]	3220.4 [2535.28-4009.16]	25.87	754.2 [592.27-938.1]	1371.42 [1053.58-1713.18]	0.07 [0.05 to 0.1]
Gabon	Female	16.94 [13.23-20.97]	38.83 [29.82-49.1]	129.22	455.44 [351.26-567.02]	587.82 [455.3-728.15]	0.27 [0.21 to 0.32]
Gambia	Female	15.41 [12.02-19.43]	41.73 [32.38-52.32]	170.8	499.06 [393.44-619.15]	686.49 [539.84-841.85]	0.1 [0.05 to 0.15]
Germany	Female	3630.94 [2823.65-4553.18]	4034.08 [3123.27-5100.53]	11.1	700.14 [539.97-882.19]	1300.93 [1008.39-1616.51]	1.77 [1.18 to 2.35]
Ghana	Female	262.02 [204.92-324.22]	700.22 [544.54-871.7]	167.24	490.15 [383.78-604.7]	999.72 [761.04-1545.52]	0.21 [0.17 to 0.25]
Greece	Female	476.71 [367.93-594.81]	534.09 [410.45-672.31]	12.04	752.83 [585.35-942.76]	1370.26 [1060.65-1712.81]	0.05 [0.03 to 0.07]
Greenland	Female	2.14 [1.66-2.69]	2.61 [2.04-3.25]	21.96	854.28 [677.85-1048.51]	1013.92 [800.31-1249]	0.01 [-0.03 to 0.05]
Grenada	Female	2.41 [1.92-2.95]	4.6 [3.61-5.67]	90.87	705.17 [553.48-880.61]	1573.6 [1250.21-1902.1]	0.55 [0.52 to 0.58]
Guam	Female	3.87 [3.03-4.83]	5.97 [4.56-7.51]	54.26	739.61 [574.87-922.63]	1048.85 [802.35-1317.85]	-0.61 [-0.77 to -0.45]
Guatemala	Female	162.41 [128.13-199.87]	467.97 [367.69-583.5]	188.14	607.75 [475.63-758.5]	747.78 [589.19-921.95]	0 [-0.03 to 0.03]
Guinea	Female	113.15 [89.62-138.47]	230.11 [179.99-286.89]	103.37	492.59 [388.53-610.29]	686.68 [539.54-843.62]	0.14 [0.08 to 0.2]
Guinea-Bissau	Female	17.97 [14.17-22.36]	36.53 [28.3-46.06]	103.28	512.89 [401.94-628.56]	670.31 [526.92-818.13]	-0.04 [-0.1 to 0.02]
Guyana	Female	20.96 [16.44-26.08]	31.31 [24.73-38.83]	49.38	708.34 [551.67-876.16]	1286.55 [1037.63-1563.12]	0.43 [0.38 to 0.48]
Haiti	Female	165.97 [131.68-206.17]	414.57 [326.16-522.63]	149.79	679.56 [535.99-850.89]	1089.33 [863.28-1337]	0.34 [0.3 to 0.38]
Honduras	Female	95.11 [74.93-117.33]	286.42 [223.73-354.08]	201.15	639.64 [501.77-797.86]	907.3 [716.55-1111.34]	0.32 [0.24 to 0.39]
Hungary	Female	1127.02 [905.18-1414.7]	905.12 [746.65-1101.99]	-19.69	1632.24 [1306.65-2003.73]	1419.87 [1164.68-1700.44]	-0.86 [-1 to -0.73]
Iceland	Female	9.2 [7.16-11.51]	14.15 [11.02-17.64]	53.8	701.75 [545.57-872.45]	1275.79 [995.02-1577.9]	-0.17 [-0.21 to -0.14]
India	Female	36806.62 [28742.55-46948.13]	91123.24 [70451.88-115379]	147.57	1047.13 [820.84-1322.43]	1821.01 [1434.25-2260.91]	1.04 [0.83 to 1.25]
Indonesia	Female	4665.13 [3686.66-5756.91]	6645.37 [5291.9-8070.46]	42.45	640.55 [503.14-789.52]	1221.13 [995.99-1451.71]	-1.35 [-1.63 to -1.08]
Iran (Islamic Republic of)	Female	1877.92	4609.73	145.47	961.34 [750.76-1192.89]	1321.27	0.18 [0.11 to 0.26]

		[1476.39-2295.82]	[3576.05-5792.16]			[1034.54-1647.31]	
Iraq	Female	490.08 [388.12-594.88]	1608.6 [1260.71-1989.19]	228.23	873.15 [672.79-1082.57]	1216.19 [956.79-1515.1]	0.29 [0.24 to 0.34]
Ireland	Female	136.49 [106.21-169.05]	233.91 [180.65-291.87]	71.38	751.04 [584.44-938.22]	1376.24 [1066.09-1717.13]	0.13 [0.11 to 0.16]
Israel	Female	180.55 [141.75-223.79]	380.93 [294.16-472.69]	110.98	752.76 [588.27-936.19]	1416.27 [1100.08-1761.17]	0.11 [0.09 to 0.14]
Italy	Female	3914.12 [3061.92-4852.51]	4202.46 [3407.1-5145.86]	7.37	1086.51 [844.08-1353.02]	1553.49 [1250.12-1877.97]	-0.57 [-0.64 to -0.5]
Jamaica	Female	66.43 [51.9-82.21]	112.84 [86.81-141.57]	69.86	697.34 [539.35-868.12]	1165.08 [920.28-1442.38]	0.25 [0.23 to 0.27]
Japan	Female	7071.58 [5430.26-8986.01]	7788.32 [6446.92-9593.5]	10.14	854.18 [662.1-1070.71]	1487.85 [1195.07-1792.3]	-0.64 [-0.8 to -0.48]
Jordan	Female	99.81 [77.07-123.53]	620.8 [452-913.59]	521.98	886 [684.85-1106.26]	1792.23 [1331.12-2622.06]	2.11 [1.71 to 2.5]
Kazakhstan	Female	1144.51 [930.78-1369.24]	1312.1 [1054.23-1595.84]	14.64	1413.5 [1150.5-1692.29]	1947.07 [1579.25-2360.53]	-0.5 [-0.62 to -0.39]
Kenya	Female	496.88 [396.25-609.06]	1403.37 [1107.05-1741.97]	182.44	721.69 [565.35-883.46]	610.41 [479.37-750.16]	0.18 [0.15 to 0.2]
Kuwait	Female	47.3 [36.65-60.24]	215.45 [163.41-278.44]	355.5	897.07 [695.03-1116.49]	1246.6 [959.4-1548.12]	0.21 [0.17 to 0.24]
Kyrgyzstan	Female	232.92 [183.02-284.51]	404.72 [316.34-499.27]	73.76	1206.45 [944.07-1473.5]	1907.44 [1520.62-2345.03]	0.5 [0.4 to 0.59]
Lao People's Democratic Republic	Female	82.13 [65.17-100.7]	183.92 [144.48-227.84]	123.94	564.32 [445.7-691.92]	1415.83 [1131.06-1730.5]	0.1 [0.06 to 0.15]
Latvia	Female	566.1 [471.08-670.41]	389.26 [318.43-478.85]	-31.24	2974.22 [2445.61-3534.82]	4156.67 [3404.74-5049.01]	-0.83 [-0.96 to -0.7]
Lebanon	Female	124.91 [95.97-154.95]	268.83 [205.46-336.84]	115.22	899.46 [689.68-1114.05]	1202.23 [927.53-1500.36]	0.21 [0.17 to 0.24]
Lesotho	Female	30.94 [24.23-38]	43.33 [33.82-54.16]	40.05	444.94 [344.84-551.29]	669.73 [527.81-819.32]	0.11 [0.04 to 0.17]
Liberia	Female	35.47 [27.81-44.55]	93.66 [72.31-116.6]	164.05	501.56 [392.67-621.14]	694.88 [542.45-859.58]	0.11 [0.07 to 0.16]
Libya	Female	111.91 [88.07-136.64]	328.31 [250.62-419.88]	193.37	893.65 [696.74-1110.79]	1214.85 [946.64-1515.64]	0.17 [0.13 to 0.21]

Lithuania	Female	745.02 [622.18-884.3]	605.89 [491.22-758.67]	-18.67	3118.08 [2590.67-3704.02]	3993.76 [3171.52-4918.5]	-1.11 [-1.33 to -0.88]
Luxembourg	Female	19.99 [15.35-24.67]	33.47 [25.79-41.59]	67.43	839.69 [648.05-1045.31]	1558.26 [1215.2-1963.17]	0.01 [-0.75 to 0.77]
Madagascar	Female	241.56 [189.64-295.95]	620.65 [485.7-773.25]	156.93	630.46 [490.56-777.01]	535.88 [419.71-656.23]	0.12 [0.08 to 0.16]
Malawi	Female	194.54 [153.38-240.77]	405.8 [322.08-499.31]	108.59	625.68 [491.54-778.16]	538.94 [423.5-660.74]	0.17 [0.14 to 0.19]
Malaysia	Female	361.63 [282.7-449.02]	934.23 [717.38-1160.25]	158.34	545.21 [422.63-675.02]	1400.71 [1073.18-1746.84]	0.37 [0.31 to 0.43]
Maldives	Female	3.43 [2.68-4.22]	11.59 [8.9-14.49]	237.9	542.48 [421.33-673.73]	1538.13 [1172.88-1933.53]	0.39 [0.35 to 0.43]
Mali	Female	155.61 [122.65-191.21]	365.4 [284.64-455.59]	134.82	507.86 [399.27-625.52]	694.13 [540.49-852.61]	0 [-0.04 to 0.05]
Malta	Female	17.22 [13.33-21.25]	23.72 [18.58-29.53]	37.75	798.3 [620.94-985.13]	1544.27 [1202.82-1918.41]	1.71 [1.22 to 2.21]
Marshall Islands	Female	0.86 [0.69-1.06]	1.76 [1.38-2.19]	104.65	703.4 [558.15-868.41]	1073.78 [846.91-1337.84]	-0.03 [-0.08 to 0.02]
Mauritania	Female	37.77 [29.85-46.27]	81.38 [63.8-100.26]	115.46	519.92 [411.48-642.58]	701.94 [551.05-861.3]	0 [-0.04 to 0.04]
Mauritius	Female	26.91 [21.12-33.46]	51.54 [39.3-65.48]	91.53	554.27 [432.24-692.36]	1381.01 [1056.36-1735.85]	0.29 [0.24 to 0.34]
Mexico	Female	2535.12 [2004.33-3138.79]	6588.09 [5228.18-7956.43]	159.87	833.85 [651.7-1033.83]	1226.77 [988.55-1468.54]	0.76 [0.14 to 1.39]
Micronesia (Federated States of)	Female	2.12 [1.68-2.63]	3.2 [2.5-3.97]	50.94	662.22 [524.8-822.52]	1043.5 [812.48-1295.87]	0.06 [0.01 to 0.12]
Mongolia	Female	89.8 [72.47-110.53]	202.58 [156.78-255.41]	125.59	1141.52 [906.68-1398.6]	1658.27 [1301.87-2047.56]	0.01 [-0.06 to 0.09]
Montenegro	Female	42.32 [33.48-52.06]	49.44 [38.9-61.47]	16.82	1272.32 [1011.44-1556.01]	1285.18 [1030.46-1572.79]	0.02 [-0.02 to 0.05]
Morocco	Female	853.99 [672.81-1048.02]	1764.38 [1342.7-2187.75]	106.6	888.37 [685.35-1101.47]	1201.07 [926.12-1497.88]	0.19 [0.14 to 0.23]
Mozambique	Female	293.78 [232.67-363.17]	664.09 [526.14-822.1]	126.05	620.41 [489.21-766.6]	562.38 [442.82-693.55]	0.44 [0.41 to 0.48]
Myanmar	Female	971.03 [780.25-1191.91]	1883.79 [1505.17-2333.76]	94	622.42 [500.27-765.02]	1476.21 [1184.5-1788.06]	-0.01 [-0.08 to 0.06]

Namibia	Female	23.5 [18.45-28.89]	49.44 [38.5-61.64]	110.38	446.44 [345.56-552.73]	665.14 [524.36-824.5]	0.15 [0.09 to 0.21]
Nepal	Female	732.39 [571.33-945.12]	1618.02 [1253.08-2047.8]	120.92	955.26 [746.62-1203.45]	1445.54 [1131.2-1789]	0.34 [0.28 to 0.4]
Netherlands	Female	782.48 [619.91-973.27]	921.03 [718.95-1150.19]	17.71	850.68 [671.97-1058.16]	1448.71 [1133.74-1803.4]	-0.33 [-0.38 to -0.28]
New Zealand	Female	189.57 [150.13-236.53]	219.78 [182.57-258.23]	15.94	1015.08 [796.25-1274.41]	1288.37 [1082.49-1508.08]	-0.82 [-1.18 to -0.46]
Nicaragua	Female	71.75 [55.91-89.25]	186.31 [143.94-234.56]	159.67	604.78 [461.84-755.59]	749.44 [582.73-924.68]	0.11 [0.06 to 0.15]
Niger	Female	123.91 [97.65-154.45]	357.68 [281.05-441.54]	188.66	493.55 [386.37-611.2]	683.57 [536.7-838.42]	0.13 [0.07 to 0.19]
Nigeria	Female	1660.77 [1310.31-2049.58]	4525.47 [3544.94-5635.91]	172.49	550.99 [431.46-682.2]	732.17 [579.07-897.2]	0.05 [-0.03 to 0.14]
North Macedonia	Female	131.82 [105.2-162.49]	168.97 [133.77-209.28]	28.18	1267.79 [1015.9-1558.87]	1289.99 [1034.39-1578.54]	0.05 [0.01 to 0.08]
Northern Mariana Islands	Female	1.08 [0.83-1.4]	1.88 [1.36-2.46]	74.07	676.09 [527.71-841.13]	1061.68 [814.6-1327]	-0.09 [-0.14 to -0.04]
Norway	Female	341.01 [267.75-417.3]	428.63 [334.53-530.3]	25.69	1324.21 [1043.14-1647.34]	2537.56 [1985.32-3154.17]	0.04 [-0.01 to 0.09]
Oman	Female	43.65 [34.68-54.08]	131.2 [101.29-166.31]	200.57	887.43 [697.19-1107.87]	1309.32 [1014.21-1643.65]	0.23 [0.18 to 0.27]
Pakistan	Female	4076.9 [3182.13-5137.37]	10180.85 [7919.9-13011.5]	149.72	1027.53 [802.59-1293.67]	1542.81 [1202.33-1922.15]	0.31 [0.2 to 0.42]
Palestine	Female	57.67 [45.58-70.61]	174.86 [136.22-216.44]	203.21	884.93 [692.38-1098.74]	1208.65 [943.36-1513.2]	0.23 [0.19 to 0.28]
Panama	Female	55.82 [42.82-70.1]	130.15 [99.23-163.72]	133.16	608.54 [462.09-771.15]	754.15 [581.71-936.92]	0.03 [0 to 0.07]
Papua New Guinea	Female	87.41 [69.11-108.36]	244.2 [187.73-306.55]	179.37	641.45 [504.68-785.74]	1031.41 [795.86-1294.47]	0.01 [-0.05 to 0.06]
Paraguay	Female	118.64 [92.97-149.07]	263.35 [206.98-331.9]	121.97	768.55 [602.12-947.33]	961.65 [759.04-1188.35]	0.12 [0.09 to 0.16]
Peru	Female	978.46 [756.89-1234.84]	2155.28 [1656.71-2717.31]	120.27	1168.99 [898.82-1462.74]	1684.65 [1297.75-2127.67]	0.18 [0.13 to 0.23]
Philippines	Female	1578.22 [1266.65-1930.23]	5297.75 [4227.22-6455.76]	235.68	738.72 [591.5-903.35]	2696.74 [2177.83-3255.03]	1.34 [1.18 to 1.49]
Poland	Female	4143.17 [3303.27-5071.27]	2274.18 [1971.06-2627.18]	-45.11	1862.67 [1490.23-2261.53]	873.44 [762.46-999.21]	-3.17 [-3.66 to -2.69]

Portugal	Female	406.79 [313.52-505.16]	664.79 [565.59-763.53]	63.42	668.35 [516.23-828.74]	1249.49 [1091.66-1438.16]	1.28 [1.03 to 1.52]
Puerto Rico	Female	132.09 [101.66-167.07]	173.71 [133.53-216.99]	31.51	698.39 [533.15-882.07]	1087.25 [840.82-1357.17]	0.25 [0.22 to 0.27]
Qatar	Female	9.22 [7.14-11.95]	66.39 [50.98-85.4]	620.07	885.88 [685.94-1099.75]	1363.09 [1062.01-1706.32]	0.26 [0.22 to 0.3]
Republic of Korea	Female	1501.96 [1161.91-1885.84]	2980.58 [2277.58-3800.05]	98.45	723.99 [552.95-912.89]	1443.72 [1109.59-1807.66]	0.11 [0.07 to 0.15]
Republic of Moldova	Female	642.05 [499.41-804.16]	726.53 [567-914.22]	13.16	2517.76 [1956.39-3122.67]	3918.49 [3057.48-4877.04]	0.38 [0.27 to 0.49]
Romania	Female	1866.93 [1480.73-2301.01]	1783.93 [1388.78-2232.22]	-4.45	1407.15 [1117.29-1726.73]	1354.04 [1075.96-1656.63]	1.84 [1.23 to 2.45]
Russian Federation	Female	33128.16 [27018.04-40622.01]	32549.91 [26319.82-40160.98]	-1.75	3336.98 [2690.64-4036.37]	4541.88 [3648.94-5522]	-0.47 [-0.64 to -0.31]
Rwanda	Female	148.58 [118.07-182.17]	323.96 [256.29-402.22]	118.04	636.4 [496.91-781.37]	542.34 [427.05-670.24]	0.06 [0 to 0.12]
Saint Lucia	Female	4.21 [3.38-5.12]	10.04 [7.91-12.48]	138.48	798.3 [639.18-986.12]	1187.62 [933.68-1471.61]	0.57 [0.49 to 0.64]
Saint Vincent and the Grenadines	Female	2.89 [2.27-3.56]	4.63 [3.58-5.93]	60.21	698.11 [539.7-876.59]	1276 [1014.06-1571.81]	0.22 [0.19 to 0.26]
Samoa	Female	3.69 [2.92-4.51]	6.02 [4.7-7.41]	63.14	686.24 [540.45-850.65]	1067.93 [827.24-1324.48]	0 [-0.08 to 0.08]
Sao Tome and Principe	Female	2.08 [1.65-2.54]	4.36 [3.39-5.4]	109.62	501.12 [394.26-621.66]	696.66 [549.24-858.51]	0.18 [0.15 to 0.2]
Saudi Arabia	Female	386.81 [301.33-477.47]	1419.13 [1078.31-1821.48]	266.88	895.76 [683.01-1114.53]	1273.28 [989.02-1597.24]	0.2 [0.16 to 0.24]
Senegal	Female	126.84 [99.18-156.21]	295.34 [231.72-364.08]	132.84	495.62 [388.21-612.56]	683.92 [535.09-841.96]	0.12 [0.06 to 0.17]
Serbia	Female	690.49 [549.57-850.33]	715.12 [563.98-890.94]	3.57	1274.36 [1011.19-1548.73]	1245.04 [993.33-1518.06]	1.29 [0.88 to 1.71]
Seychelles	Female	1.65 [1.3-2.04]	3.56 [2.73-4.47]	115.76	542.51 [424.59-677.62]	1509.98 [1197.97-1850.35]	0.47 [0.44 to 0.49]
Sierra Leone	Female	66.48 [52.1-82.58]	154.13 [121.03-193.3]	131.84	492.26 [387.06-607.48]	689.39 [545.14-853.67]	0.17 [0.12 to 0.22]
Singapore	Female	112.29 [86.54-139.82]	282.03 [213.09-359.53]	151.16	752.96 [585.21-945.64]	1460 [1121.81-1834.12]	-0.06 [-0.11 to

							-0.01]
Slovakia	Female	410.13 [330.35-500.68]	483.63 [382.59-599.97]	17.92	1403.67 [1125.18-1704.9]	1416.67 [1127.33-1728.94]	-0.45 [-0.78 to -0.11]
Slovenia	Female	132.67 [106.97-161.93]	149.81 [118.86-186.4]	12.92	1134.32 [909.71-1385.15]	1212.3 [964.1-1480.7]	0.01 [-0.15 to 0.17]
Solomon Islands	Female	6.79 [5.31-8.4]	16.9 [13.11-21.45]	148.9	681.39 [537.41-846.53]	1050.73 [819.23-1312.54]	-0.08 [-0.15 to -0.01]
Somalia	Female	152.06 [118.31-189.58]	415.01 [326.49-509.61]	172.93	651.23 [508.18-799.63]	542.22 [422.76-665.05]	0.07 [0.04 to 0.09]
South Africa	Female	793.16 [621.01-982.83]	1477.55 [1152.46-1841.32]	86.29	496.78 [390.05-615.34]	740.36 [584.86-910.43]	0.08 [0 to 0.16]
South Sudan	Female	105.21 [82.82-130.88]	206.99 [161.73-258.19]	96.74	619.74 [483.9-763.52]	533.43 [416.22-657.46]	0.18 [0.15 to 0.21]
Spain	Female	1728.91 [1355.89-2149.69]	2420.63 [1852.35-3026.3]	40.01	759.85 [591.76-950.73]	1388.66 [1084.79-1732.65]	0.06 [0.05 to 0.08]
Sri Lanka	Female	398.11 [309.28-497.44]	807.46 [618.5-1011.91]	102.82	552.95 [429.51-690.29]	1354.93 [1049.65-1685.03]	0.28 [0.23 to 0.34]
Sudan	Female	586.74 [459.91-728.46]	1371.17 [1072.73-1698.51]	133.69	883.17 [675.91-1111.92]	1193.14 [927.08-1475.74]	0.19 [0.13 to 0.24]
Suriname	Female	11.33 [8.8-14.02]	23.63 [18.24-29.51]	108.56	688.14 [533.22-854.62]	1429.08 [1150.25-1721.64]	0.26 [0.22 to 0.3]
Sweden	Female	457.96 [358.86-559.32]	517.8 [402.24-648.41]	13.07	853.17 [666.95-1050.32]	1348.1 [1057-1661.9]	-0.04 [-0.26 to 0.18]
Switzerland	Female	346.98 [269.78-429.65]	460.02 [356.08-575.2]	32.58	812.23 [631.83-1009.75]	1503.18 [1160.4-1867.68]	0.04 [-0.28 to 0.37]
Syrian Arab Republic	Female	346.8 [274.18-423.8]	722.87 [555.68-907.67]	108.44	883.78 [688.71-1097.54]	1194.85 [934.41-1495.66]	0.26 [0.22 to 0.29]
Tajikistan	Female	214.92 [169.21-266.48]	483.71 [377.89-605.29]	125.07	1081.69 [845.25-1336.55]	1742.18 [1401.67-2129.84]	0.26 [0.19 to 0.33]
Thailand	Female	1424.3 [1116.95-1764.28]	3375.02 [2632.96-4225.51]	136.96	571.57 [447.66-704.11]	1608.4 [1301.43-1968.53]	0.58 [0.55 to 0.61]
Timor-Leste	Female	13.6 [10.7-17.16]	29 [23.18-35.43]	113.24	543.61 [432.85-670.16]	1370.39 [1085.12-1697.64]	0.31 [0.27 to 0.34]

Togo	Female	60.92 [47.66-77.6]	167.47 [129.21-210.93]	174.9	496.71 [390.29-616.69]	665.35 [525.65-817.76]	0.16 [0.1 to 0.23]
Tonga	Female	2.17 [1.68-2.7]	2.94 [2.27-3.64]	35.48	613.47 [472.45-772.38]	1029.01 [801.66-1287.89]	0.24 [0.18 to 0.29]
Trinidad and Tobago	Female	38.53 [29.96-48.08]	74.46 [58.66-92.36]	93.25	745.3 [580.38-926.3]	1768.54 [1338.69-2882.31]	0.84 [0.69 to 0.98]
Tunisia	Female	291.36 [225.05-355.19]	641.31 [492.64-807.38]	120.11	899.03 [691.02-1119.03]	1212.34 [946.37-1517.71]	0.2 [0.16 to 0.23]
Turkey	Female	2105.72 [1676.94-2614.23]	4427.65 [3413.62-5595.31]	110.27	875.31 [689.27-1087.28]	1232.54 [971.68-1540.71]	0.3 [0.25 to 0.35]
Turkmenistan	Female	158.23 [125.95-196.49]	290.05 [226.9-355.66]	83.31	1083.96 [863.05-1337.83]	1668.62 [1315.54-2051.08]	0.28 [0.2 to 0.35]
Uganda	Female	319.29 [251.12-396.86]	835.9 [657.86-1031.88]	161.8	612.39 [478.15-754.38]	536.57 [417.29-660.79]	0.18 [0.14 to 0.23]
Ukraine	Female	12683.98 [10133.44-15755.99]	9565.35 [7580.15-11925.61]	-24.59	3418.16 [2741.63-4158.16]	4282.6 [3377.63-5271.76]	-0.89 [-1.05 to -0.72]
United Arab Emirates	Female	36.76 [28.36-47.22]	264.69 [198.79-354.02]	620.05	889.82 [681.02-1111.55]	1337.56 [1047.6-1680.12]	0.16 [0.11 to 0.21]
United Kingdom	Female	3302.86 [2589.44-4055.05]	4538.15 [3654.5-5476.76]	37.4	924.76 [721.31-1149.86]	1792.3 [1447.42-2165.2]	1.14 [0.8 to 1.47]
United Republic of Tanzania	Female	518.17 [410.37-633.72]	1306.47 [1023.32-1617.6]	152.13	613.52 [483.06-749.49]	540.14 [422.76-658.6]	0.24 [0.19 to 0.29]
United States of America	Female	17788.56 [14051.26-21985.47]	17602.33 [15262.99-20365.21]	-1.05	1227.97 [969.62-1516.81]	974.46 [844.52-1120.12]	-1.94 [-2.29 to -1.59]
United States Virgin	Female	5.2 [4.11-6.47]	7.77 [5.93-9.77]	49.42	942.68 [749.14-1163.34]	1571.62 [1217.85-1928.31]	0.4 [0.14 to 0.66]
Uruguay	Female	198.85 [154.05-247.47]	241.86 [187.78-303.39]	21.63	1193.29 [915.07-1510.38]	1676.55 [1305.44-2117.06]	0.07 [0.03 to 0.12]
Uzbekistan Female	Female	904.46 [709.41-1131.12]	1936.5 [1517.76-2390.24]	114.11	1111.89 [864.5-1375.55]	1679.65 [1334.46-2080.39]	0.19 [0.14 to 0.24]
Vanuatu	Female	2.87 [2.22-3.62]	7.22 [5.59-9.01]	151.57	607.11 [469.75-762.27]	1018.25 [774.9-1274.89]	0.19 [0.12 to 0.25]
Venezuela (Bolivarian Republic	Female	450.62 [353.9-556.4]	1069.95 [829.79-1332.51]	137.44	648.43 [504.65-802.93]	860.12 [684.06-1054.01]	0.05 [0 to 0.1]

Female of)

Viet Nam	Female	1634.28 [1292.44-2012.66]	5896.93 [4240.8-8752.33]	260.83	618.43 [489.05-765.18]	2074.64 [1579.98-2770.63]	2.37 [1.95 to 2.79]
Yemen	Female	350.83 [277.85-434.69]	1033.6 [808.41-1286.12]	194.62	883.77 [690.36-1102.36]	1198.72 [930.36-1489.5]	0.18 [0.13 to 0.23]
Zambia	Female	158.65 [124.44-195.4]	401.53 [317.62-501.71]	153.09	667.13 [522.65-826.26]	542.89 [426.97-666.88]	-0.02 [-0.08 to 0.03]
Zimbabwe	Female	158.48 [123.13-199.42]	283.56 [220.91-353.95]	78.92	450.77 [350.46-562.15]	687.19 [547.16-846.74]	0.06 [0 to 0.13]

ASIR: age-standardized incidence; EAPCs: estimated annual percentage change, CI: confidence interval; UI: Uncertainty interval.

Supplementary Table S4. Age distribution of incidence (per 100,000) for Urolithiasis in different countries in 2019.

Supplementary Table S4. Age distribution of incidence (per 100,000) for Urolithiasis in different countries in 2019.

Country	sex	<5	5 to 9	10 to 14	15 to 19	20 to 24	25 to 29	30 to 34	35 to 39	40 to 44	45 to 49	50 to 54	55 to 59	60 to 64	65 to 69	70 to 74	75 to 79	80 to 84	85 to 89
Afghanistan	Male	52.04	99.54	192.66	364.96	677.88	1183.47	1908.56	2560.89	2988.55	3320.33	3496.97	3353.92	2846.85	2316.66	1736.25	1077.29	589.93	266.58
Afghanistan	Female	53.03	101.34	176.01	268.01	381.35	554.97	890.35	1289.93	1661.86	2108.77	2535.22	2657.73	2308.45	1774.55	1220.32	678.62	314.3	118.99
Albania	Male	112.43	171.2	264.06	465.6	777.56	1095.36	1418.39	1652.77	1871.32	2239.11	2605.99	2920.66	3137.37	3359.31	3246.2	2670.72	2025.44	1395.81
Albania	Female	121.05	216.08	408.44	845.81	1288.19	1427.25	1488.92	1541.71	1632.04	1898.46	2211.61	2494.37	2638	2713.38	2521.61	1946	1343.4	841.11
Algeria	Male	52.16	99.44	191.73	364.5	679.32	1189.83	1931.78	2602.15	3053.05	3392.41	3594.84	3488.54	2959.34	2442.55	1899.59	1227.32	695.82	324.36
Algeria	Female	52.81	100.83	176.63	270.71	383.96	560.43	896.39	1294.76	1686.92	2166.71	2639.72	2785.54	2415.44	1879.05	1315.04	738.07	342.46	128.76
American Samoa	Male	33.56	48.45	85.12	184.37	425.07	869.8	1524.06	2140.32	2675.83	3295.6	3853.76	4078.84	3675.23	3018.79	2381.85	1950.45	1405.46	751.23
American Samoa	Female	21.44	32.4	56.11	109.78	211.94	383.87	675.93	999.65	1252.74	1497.03	1738.64	1873.36	1751.55	1472.85	1070.08	611.43	304.64	130.91
Andorra	Male	18.43	25.56	56.15	193.34	626.24	1387.89	2357.87	3138.27	3740.82	4465.81	5064.26	5300.88	5058.17	4699.23	4044.44	2958.52	2083.65	1504.96
Andorra	Female	11.57	18.44	45.41	155.53	408.12	717.1	1036.54	1261.69	1420.05	1625.92	1788.97	1821.32	1668.81	1500.91	1265.21	874.33	550.06	350.29
Angola	Male	29.4	46.54	85.85	177.08	341.21	559.87	810.5	997.29	1155.21	1392.66	1629.51	1778.29	1744.77	1555.62	1197.18	753.65	431.31	243.79
Angola	Female	29	53.48	99.16	172.2	275.62	414.74	629.67	825.7	967.53	1107.78	1136.22	1025.51	846.34	692.56	524.64	311.49	148.5	59.69
Antigua and Barbuda	Male	46.67	72.76	128.45	254.45	507.48	897.48	1419.67	1902.28	2391.06	3108.42	3868.6	4398.98	4318.17	3827.35	2976.61	1842.24	1005.03	521.61
Antigua and Barbuda	Female	34.65	58.33	105.18	193.43	344.95	579.26	920.09	1233.89	1516.46	1869.12	2133.67	2143.89	1853.11	1426.19	957.27	552.75	324.12	182.01
Argentina	Male	26.04	48.94	112.76	313.6	848.56	1809.77	3090.03	4059.12	4659.01	5155.36	5239.96	4894.3	4189.45	3483.54	2748.11	1871.98	1178.49	688.59
Argentina	Female	14.75	34.79	101.67	292.96	672.81	1220.81	1900.34	2372.52	2629.99	2860.87	2811.97	2453.47	1928.69	1475.76	1093.98	704.73	419.27	230.89
Armenia	Male	96.73	161.14	288.64	627.14	1458	2376.11	2830.66	3057.52	3886.37	5541.1	7088.41	8821.71	12854.87	15595.05	13985.93	12020.17	9406.03	6509.39
Armenia	Female	61.94	113.68	250.92	534.05	856.29	1098.74	1378.07	1652.01	1992.33	2526.1	3059.67	3741.81	4867.14	5463.56	4990.63	4269.38	3365.19	2400.66
Australia	Male	19.33	26.95	56.93	178.02	524.77	1132.16	1918.44	2599.64	3236.24	4049.28	4764.59	5136.59	5055.04	4869.57	4349.74	3354.28	2454.22	1848.51
Australia	Female	10.33	17.43	45.13	148.86	401.34	744.76	1054.53	1210.13	1304.57	1460.44	1580.09	1609.23	1501.69	1345.53	1118	797.68	553.89	377.75
Austria	Male	24.19	31.81	74.77	278.64	1007.29	2237.49	3897.46	5644.12	7064.33	8209.65	9149.42	9553.29	8990.82	8118.79	7040.51	5342.68	3419.44	1985.73
Austria	Female	14.25	21.04	60.62	246.92	690.2	1178.48	1608.55	1999.44	2345.08	2723.62	2986.33	2997.79	2878.45	2602.44	2146.02	1596.9	996.03	537.48

Azerbaijan	Male	97.3	162.16	292.35	632.34	1459.5	2389.78	2836.69	2945.93	3283.29	3955.09	4562.98	5076.54	5439.67	5514.21	4739.9	3505.35	2445.55	1435.61
Azerbaijan	Female	61.4	113.1	249.53	527.46	850.8	1090.02	1346.21	1588.83	1860.6	2263.16	2628.97	2792.64	2576.62	2150.85	1595.98	1138	861.24	538.3
Bahamas	Male	46.87	73.21	128.87	254.23	506.87	901.59	1438.32	1932.85	2463.77	3259.97	4074.38	4617.83	4505.45	3908.03	2999.37	1859.54	982.96	497.29
Bahamas	Female	35.26	58.71	104.61	191.92	342.72	572.81	914.46	1227.97	1497.92	1814.25	1984.98	1899.52	1581.56	1193.41	803.89	460.67	255.18	129.36
Bahrain	Male	51.92	99.89	193.67	365.1	678.43	1189.34	1929.16	2592.78	3041.08	3417.58	3606.89	3460.55	2949.16	2432.08	1899.59	1268.46	806.14	507.41
Bahrain	Female	52.6	101.04	176.37	268.88	382.76	562.01	900.29	1288.9	1682.16	2172.1	2634.25	2765.95	2392.84	1850.9	1282.3	730.84	374.76	185.19
Bangladesh	Male	48.68	89.48	189.79	457.5	978.2	1619.39	2303.95	2839.55	3259.98	3801.18	4337.61	4743.24	4782.14	4434.93	3465.92	2475.64	1809.25	1038.13
Bangladesh	Female	31.25	59.6	146.88	416.82	903.33	1355.13	1714.5	1895.88	1954.16	2115.72	2194.35	2030.79	1641.82	1240.58	870.93	510.67	262.3	122.44
Barbados	Male	46.89	73.08	128.22	254.15	511.5	913.05	1460.4	1977.67	2566.95	3489.36	4524.3	5325.61	5334.9	4781.77	3822.59	2478.54	1345.49	810.48
Barbados	Female	34.71	58.29	104.89	192.03	345.03	578.95	913.26	1225.12	1505.21	1825.94	2026.18	1965.64	1640.22	1243	842.34	489.58	281.61	152.89
Belarus	Male	71.05	103.8	202.29	568.61	1573.64	3188.27	5452.28	7796.93	9840.8	12091.8	14238.02	15505.19	15926.59	15822.59	13230.67	10176.49	7198.83	3755.79
Belarus	Female	48.36	82.03	205.94	615.01	1311.68	1990.06	2627.16	3184.18	3949.84	5319.76	6900.77	7996.01	7868.46	7101.54	5588.39	3591.95	2121.33	1035.53
Belgium	Male	20.77	28.63	61.49	212	697.57	1538.87	2611.48	3472.07	4160.83	4977.15	5620.67	5899.22	5657.7	5319.7	4622.03	3366.93	2515.59	2011.02
Belgium	Female	12.32	19.5	48.34	167.55	441.91	783.42	1125.68	1362.29	1531.04	1745.98	1922.75	1964.99	1818.47	1680.12	1481.19	1089.21	776.85	674.75
Belize	Male	46.84	73.02	129.58	255.85	510.47	917.27	1465.52	1970.32	2515.55	3343.02	4331.1	5140.73	4964.63	4107.48	3065.92	1877.06	996.65	515.64
Belize	Female	35.11	59.14	105.26	192.68	344.7	584.82	937.93	1255.86	1547.32	1927.3	2187.09	2137.85	1762.67	1291.98	849.54	477.04	263.15	138.35
Benin	Male	28.71	44.24	83.07	179.2	356.71	576.88	808.55	980.22	1143.01	1411.43	1744.22	2253.8	2880.64	3184.94	2653.27	1718.19	1061.45	647.87
Benin	Female	27.99	50.04	95.09	170.92	278.81	420.22	623.67	828.46	1017.99	1189.82	1192.78	1051.32	897.39	851.59	810.18	542.38	225.5	78.2
Bermuda	Male	47.32	74	129.87	254.37	506.21	898.12	1417.73	1890.95	2398.49	3125.79	3899.3	4450.38	4402.41	3980.99	3176.37	2010.3	1099.36	581.42
Bermuda	Female	34.92	58.94	105.53	192.22	342.34	579.17	919.12	1229.65	1514.24	1851.41	2061.03	2007.96	1706.11	1337.63	953.2	599.72	400.65	273.44
Bhutan	Male	49.17	88.81	190.39	462.89	988.52	1623.65	2304.31	2847.41	3269.6	3803.67	4344.76	4761.21	4834.01	4546.87	3578.18	2371.06	1543.88	912.53
Bhutan	Female	31.5	60.02	146.15	416.45	902.8	1354.76	1717.2	1902.43	1957.73	2110.59	2199.05	2042.61	1641.23	1255.27	927.1	568.89	288.59	135.13
Bolivia (Plurinational State of)	Male	38.63	56.76	105.07	273.86	801.12	1843.73	3219.41	4182.74	4694.95	5150.37	5237.29	4788.19	3955.09	3265.1	2553.05	1706.99	1116.96	801.77
Bolivia (Plurinational State of)	Female	24.38	40.29	87.03	232.25	556.58	1041.52	1668.99	2172.95	2541.37	2948.51	3066.91	2747.67	2163.36	1624.18	1132.06	679.65	420.14	278.99
Bosnia and Herzegovina	Male	112.57	170.22	262.24	466.81	777.86	1097.07	1405.37	1628.37	1849.6	2234.41	2625.06	2933.65	3110.9	3272.25	3101.33	2549.54	2010.41	1462.36
Bosnia and	Female	121.04	215.48	406.41	836.55	1284.34	1436.99	1489.68	1529.5	1621.72	1887.62	2213.23	2489.15	2597.01	2650.25	2418.31	1799.12	1165.98	665.86

Herzegovina																			
Botswana	Male	38.45	66.54	142.5	347.88	674.98	945.89	1175.04	1364.22	1486.12	1625.31	1779.33	1969.38	2101.11	2087	1651.11	965.15	536.78	346.3
Botswana	Female	28.05	56.79	111.51	176.53	246.4	365.33	589.77	806.65	942.02	1053.59	1066.24	963.85	820.7	706.72	567.65	367.99	193.36	87.66
Brazil	Male	28.52	44.38	84.16	188.82	423.15	777.04	1242.95	1650.19	1957.95	2334.77	2722.61	2958.2	3005.57	2968.44	2552.99	1896.66	1443.23	1152.99
Brazil	Female	21.82	37.19	94.2	264.35	542	822.8	1136.38	1383.26	1525.6	1695.15	1824.69	1804.5	1583.83	1293.42	988.77	718.88	547.84	403.11
Brunei Darussalam	Male	32.87	62.6	121.33	274.74	677.78	1360.66	2387.23	3507.19	4503.16	5309.54	5580.99	5248.85	4491.43	4022.06	3915.52	3488.84	2958.13	2550.1
Brunei Darussalam	Female	23.67	47.77	94.35	181.01	283.65	362.24	486.99	722.28	1121.35	1676.45	2244.69	2483.4	2301.41	2167.13	1983.74	1627.8	1238.99	892.91
Bulgaria	Male	112.35	170.88	262.27	461.4	775.61	1097.32	1415.24	1647.4	1867.54	2235.2	2620.41	2944.38	3129.63	3287.68	3115.29	2526.65	1897.57	1285.58
Bulgaria	Female	122.15	216.63	406.97	838.59	1281.24	1433.31	1493.58	1526.36	1616.21	1890.4	2208.35	2487.82	2571.43	2579.7	2332.89	1731.81	1167.84	721.12
Burkina Faso	Male	28.43	44.29	85.01	184.64	369.04	583.31	804.29	978	1145.07	1409.64	1738.19	2275.63	2933.47	3252.03	2757.05	1837.48	1163.74	681.77
Burkina Faso	Female	27.61	49.58	95.06	172.19	279.27	421.41	631.19	831.89	1048.28	1255.71	1238.76	1071.23	913.64	886.23	1027.78	790.67	287.77	87.91
Burundi	Male	19.09	25.62	44.66	98.01	199.19	326.67	475.52	597.31	690.3	807.94	936.21	1056.68	1113.7	1096.17	892.46	555.86	310.09	162.03
Burundi	Female	28.89	52.45	99.42	178.99	285.87	427.29	652.29	944.18	1370.69	1717.21	1568.41	1207.07	1013.19	1148.27	1503.86	1206.14	464.67	152.12
Cabo Verde	Male	28.46	43.52	82.25	179.8	358.36	576.67	808.07	978.28	1143.47	1422.15	1765.33	2308.89	3022.64	3392.76	2908.68	1994.92	1316.86	829.29
Cabo Verde	Female	27.86	50.15	95.49	170.32	279.21	429.19	638.55	839.25	1024.01	1201.03	1215.7	1083.81	939.93	915.89	872.71	587.92	260.11	96.07
Cambodia	Male	35.87	52.64	91.17	191.69	472.43	1023.9	1898.74	2869.72	4045.57	5725.04	7169.55	7844.77	8804.71	8559.33	6361.81	4794.69	3224.72	1619.99
Cambodia	Female	21.69	33.33	56.15	106.44	215.59	399.02	636.19	798.7	971.93	1337.63	1658.08	1767.66	1732.32	1556.35	1135.83	665.42	387.31	232.54
Cameroon	Male	28.39	43.95	83.29	178.94	356.02	577.11	809.02	981.55	1145.84	1402.18	1730.65	2272.59	2920.42	3212.65	2671.57	1730.4	1073.14	664.6
Cameroon	Female	27.74	49.62	95.33	171.85	280.04	424.06	629.79	835.6	1030.45	1200.05	1199.22	1060.08	908.74	862.25	830.18	564.54	232.45	80.4
Canada	Male	21.66	40.58	91.21	230.48	490.01	810.79	1159.72	1418.02	1638.72	1984.23	2422.02	2958.64	3464.23	4079.87	4371.88	4276.04	3832.8	3281.5
Canada	Female	20.24	40.74	115.34	373.79	757.39	1046.63	1269.34	1358.82	1358.76	1432.12	1529.1	1635.92	1678.81	1752.17	1717.7	1466.64	1116.41	818.34
Central African Republic	Male	29.3	46.14	84.8	175.67	340.37	552.44	799.15	991.25	1155.4	1398.08	1632.63	1779	1734.02	1512.62	1126.69	687.07	388.5	214.38
Central African Republic	Female	29.15	53.38	99.03	173.76	276.52	414.26	624.57	814.64	955.08	1092.41	1114.09	1001.98	824.98	670.99	503.6	292.92	137.09	53.53
Chad	Male	28.37	43.67	82.45	179.21	357.6	574.32	803.77	977.17	1144.18	1405.4	1726.13	2257.38	2880.69	3172.91	2662.09	1732.07	1072.55	630.46
Chad	Female	27.69	49.93	95.1	170.67	277.83	420.23	622.96	822.03	1011.77	1185.28	1187.22	1051.88	906.55	847.71	759.93	487.6	209.41	75.98

Chile	Male	25.74	48.81	113.15	317.68	857.21	1833.51	3154.67	4131.93	4728.21	5249.29	5379.76	5073.73	4366.96	3647.13	2882.03	2046.03	1381.39	958.08
Chile	Female	14.6	34.58	100.88	293.61	675.3	1222.47	1897.59	2371.04	2638.88	2875.47	2842.24	2483.62	1939.64	1483.87	1097.56	705.3	420.05	228.4
China	Male	24.52	26.4	43.45	120.33	344.43	733.28	1258.88	1735.52	2121.77	2697.18	3199.92	3320.65	3197.91	3096.98	2733.95	2193.7	1780.85	1521.11
China	Female	12.65	14.73	27.99	76.55	167.43	278.88	442.09	634.68	846.37	1207.92	1595.23	1743.47	1628.41	1466.77	1193.44	845.07	553.69	335.48
Colombia	Male	48.73	68.24	103.1	187.41	377.16	702.34	1128.35	1477.82	1752.05	1952.04	2015.72	2043.01	1982.91	1937.81	1786.54	1441.57	1070.57	736.98
Colombia	Female	42.58	69.96	87.71	100.74	156.62	295.06	561.41	900.97	1293.41	1715.82	1929.28	1812.48	1456.83	1095.92	790.89	536.69	385.87	276.93
Comoros	Male	19.15	25.66	44.84	97.2	197.38	323.26	469.42	589.86	680.32	805.44	940.04	1059.2	1118.51	1107.77	907.98	569.75	321.77	168.08
Comoros	Female	29.08	53.09	100.63	181.11	286.25	424.83	651.9	955.32	1434.88	1829.32	1632.85	1207.63	1009.62	1156.82	1691.63	1438.96	515.74	150.35
Congo	Male	29.24	46.19	85.12	175.14	339.53	552.91	799.86	993.01	1159.06	1395.06	1629.1	1786.91	1753.81	1554.93	1188.69	746.83	429.18	242.47
Congo	Female	29	53.48	99.14	173.52	278.02	419.5	629.61	823.73	969.41	1113.95	1142.52	1025.49	845.72	697.18	530.94	313.12	148.96	59.4
Costa Rica	Male	48.76	68.39	103.83	188.98	378.78	694.9	1106.22	1451.76	1717.34	1901.97	1949.67	1957.08	1896.89	1870.7	1765.43	1469.09	1134.48	828.63
Costa Rica	Female	42.59	70.13	87.75	100.2	155.63	290.67	543.87	868.35	1256.51	1670.69	1872.72	1754.39	1410.72	1077.38	794.89	551.01	416.06	324.74
Côte d'Ivoire	Male	28.46	43.96	83.83	182.47	361.07	574.56	802.02	972.12	1139.45	1403.35	1740.76	2277.21	2915.15	3199.28	2651.79	1733.78	1085.36	652.17
Côte d'Ivoire	Female	27.79	49.49	94.62	171.01	279.38	425.94	635.46	833.47	1019.52	1196.39	1199.14	1054.21	893.92	842.44	797.2	531.54	221.88	78.4
Croatia	Male	69.3	113.92	198.39	332.65	553.82	919.36	1486.93	2227.85	2924.78	3534.25	3979.03	4163.48	4231.27	4162.87	3569.4	2988.99	2166.55	1247.26
Croatia	Female	73.29	107.73	223.46	573.11	901.01	1089.24	1262.56	1455.86	1988.58	2539.01	2795.44	2833.95	2700.8	2557.22	2104.95	1488.64	950.41	494.18
Cuba	Male	46.52	72.79	129.52	255.07	507.04	905.52	1462.92	2007.7	2668.44	3738.43	5010.08	6333.88	7000.34	6524.31	5605.27	3854.24	1798.72	945.6
Cuba	Female	34.82	58.55	104.9	192.67	347.85	589.77	933.34	1252.91	1581.74	1987.71	2282.57	2345.92	2117.21	1659.73	1085.24	604.54	334.16	177.94
Cyprus	Male	16.08	23.08	50.47	169.22	549.72	1220.97	2062.65	2750.8	3306.55	3942.05	4422.82	4611.74	4405.82	4105.93	3534.16	2546.2	1710.06	1097.62
Cyprus	Female	10.13	16.45	40.76	137.22	360.45	640.79	920.76	1118.88	1258.46	1436.16	1581.89	1615.75	1485.54	1328.7	1121.44	855.68	716.99	483.65
Czechia	Male	118.75	180.05	275.75	490.56	824.89	1164.26	1498.94	1741.2	1972.13	2368.19	2796.06	3146.56	3348.53	3552.17	3344.45	2652.18	1912.56	1294.92
Czechia	Female	127.37	224.78	421.16	874.94	1342.6	1495.05	1553.89	1600.78	1692.02	1964.06	2299.84	2591.37	2670.09	2664.53	2347.57	1674.34	1083.11	644.27
Democratic People's Republic of Korea	Male	23.26	28.87	49.67	131.82	362.26	781.3	1394.02	1942.83	2424.38	3142.52	3927.14	4252.8	4081.27	3779.61	3133.67	2266.83	1632.28	1262.93
Democratic People's Republic of Korea	Female	13.69	17.92	33.84	83.78	175.63	305.07	503.35	712.72	946.11	1338.63	1778.29	1971.47	1845.25	1654.91	1350.45	926.63	586.36	333.26

Democratic Republic of the Congo	Male	29.38	46.87	85.47	176.8	340.03	548.79	798.59	994.51	1155.94	1388.77	1615.73	1766.57	1736.82	1540.82	1177.67	736.81	418.01	230.16
Democratic Republic of the Congo	Female	28.92	52.92	98.46	173.48	275.12	416.08	630.69	821.42	965.9	1111.38	1136.81	1017.7	842.25	698.63	533.55	316.39	149	58.29
Denmark	Male	16.13	22.85	49.98	170.59	555.57	1228.02	2083.2	2789.29	3352.11	4050.03	4648.65	4964.71	4967.35	5118	5291.22	4831.43	4644.12	4619.41
Denmark	Female	10.32	16.7	41	139.98	366.03	652.37	938.64	1129.09	1262.44	1446.99	1605.35	1659.89	1543.21	1431.99	1305.48	982.58	765.69	605.97
Djibouti	Male	19.1	25.62	44.65	98.38	200.06	328.58	475.27	591.06	684.88	810.18	945.5	1068.74	1125.51	1112.76	910.69	570.98	322.69	174.17
Djibouti	Female	29.22	53.42	100.88	180.76	286.45	426.66	658.82	974.19	1538.92	2031.94	1759.58	1226.39	1014.08	1171.19	1830.71	1585.88	537.24	150.15
Dominica	Male	46.78	72.66	128.94	254.19	504.25	900.89	1419.46	1891.57	2377.36	3080.49	3844.34	4338.85	4196	3697.06	2854.32	1740.6	935.94	474
Dominica	Female	34.93	58.37	104.75	190.54	340.55	569.78	895.15	1194.9	1470.11	1777.97	1930.63	1843.36	1526.86	1151.79	788.22	455.59	251.62	128.26
Dominican	Male	46.76	73.29	129.45	252.23	504.67	895.83	1414.41	1884.45	2364.15	3062.81	3803.76	4324.09	4254.35	3781.59	2947.35	1843.06	1019.55	533.85
Dominican	Female	34.83	58.23	103.94	189.61	337.34	561.8	886.1	1181.64	1448.17	1763.88	1936.05	1849.08	1540.37	1183.67	819.13	482.12	273.87	142.34
Ecuador	Male	33.8	55.58	90.94	211.38	697.18	1909.39	3558.35	4842.86	5297	5545.47	5917.59	5507.61	4934.77	4307.22	3313.23	2501.78	1938.61	1567
Ecuador	Female	19.91	36.94	83.8	287.05	702.59	1328.6	2086.1	2945.28	3465.65	3853.63	4257.48	3959.64	3207.45	2554.47	1809.65	1109.64	753.18	590.35
Egypt	Male	51.85	99.71	193.22	364.87	677.24	1186.93	1930.1	2590.17	3023.28	3392	3596.54	3458.67	2923.38	2400.77	1866.51	1204.46	682.88	318.06
Egypt	Female	52.74	101.13	176.01	270.89	385.4	564.13	904.7	1293.31	1674.17	2154.41	2611.05	2744.86	2402.44	1888.65	1328.42	751.58	352.18	135.75
El Salvador	Male	49.36	69.16	103.95	188.31	374.77	687.74	1109.98	1455.47	1715.3	1895.47	1936.11	1948.21	1882.62	1830.99	1698.08	1375.17	1002.01	686.45
El Salvador	Female	42.42	69.82	87.69	100.41	154.81	287.23	542.09	863.85	1250.76	1663.38	1854.74	1734.91	1401.75	1075.07	792.17	547.91	407.77	310.5
Equatorial Guinea	Male	29.33	46.61	85.81	176.59	339.25	552.58	806.14	1000.56	1160.83	1398.08	1633.84	1806.56	1794.69	1610.32	1261.57	822.46	490.43	288.93
Equatorial Guinea	Female	28.81	53.31	99.66	173.81	276.71	418.76	633.01	826.04	969.96	1113.51	1149.57	1041.97	865.65	717.3	549.31	331.25	161.97	70.89
Eritrea	Male	18.92	25.5	44.72	97.59	198.37	326.74	472.96	589.57	677.71	799.42	934.14	1060.59	1123.78	1101.46	893.3	558.02	310.93	159.32
Eritrea	Female	28.73	52.19	99.22	179.47	285.64	422.44	649.91	956.74	1460.46	1887.47	1670.43	1211.8	1004.81	1142.19	1596.35	1330.29	492.04	149.63
Estonia	Male	71.13	103.25	199.54	567.45	1564.05	3163.47	5403.55	7741.29	9747.42	11926.25	13865.57	14873.18	14398.59	13003.21	10461.13	7827.01	6109.43	4098.85
Estonia	Female	48.26	81.66	204.6	612.28	1311.48	1981.39	2610.42	3176.46	3979.07	5361.94	6946.66	8013.99	7780.41	6947.22	5487.97	3606.8	2183.44	1127.13
Eswatini	Male	38.56	66.88	144.36	354.04	681.54	954.05	1187.28	1394.38	1544.93	1707.6	1889.54	2075.19	2191.3	2141.22	1650.68	947.68	511.58	328.64
Eswatini	Female	27.89	55.91	110.57	175.13	243.94	363.59	589.57	807.62	939.75	1046.03	1057.37	952.32	805.5	689.54	554.32	360.29	186.27	78.19
Ethiopia	Male	26.39	37.94	64.28	126.02	234.77	375.58	541.22	656.57	734.66	881.9	1054.01	1174.23	1184.15	1119.52	897.24	549.21	296.39	150.16

Ethiopia	Female	37.9	66.48	119.88	213.05	342.85	510.59	770.81	1087.54	1546.16	1960.28	1837.68	1444.68	1236.39	1378.98	1707.02	1311.88	503.08	186.29
Fiji	Male	33.84	48.57	85.21	183.37	421.12	858.16	1505.26	2100.7	2615.26	3226.19	3754.58	3911.47	3499.11	2917.17	2259.36	1609.77	1043.59	551.09
Fiji	Female	21.22	32.3	56.33	110.44	212.27	384.62	676.37	985.77	1239.14	1496.22	1745.2	1880.66	1749.51	1466.59	1062.37	608.69	308.55	134.33
Finland	Male	15.8	22.89	50.53	169.96	550.51	1210.11	2057.56	2761.01	3324.54	3958.31	4447.26	4641.19	4437.58	4165.04	3608.83	2628.4	1896.41	1341.41
Finland	Female	10.04	16.54	40.51	137.6	359.94	636.55	914.25	1110	1252.69	1438.53	1575.65	1600.91	1468.04	1307.92	1085.78	724.53	420.91	229.64
France	Male	18.59	25.83	56.24	191.86	624.59	1388.09	2357.23	3150.04	3760.96	4481.56	5095.42	5336	5062.3	4655.37	3919.06	2793.42	1872.31	1254.95
France	Female	11.3	18.23	45.38	155.16	408.25	719.01	1034.79	1266.85	1423.97	1620.25	1776.32	1801.46	1646.77	1460.16	1192.67	790.59	490.09	307.82
Gabon	Male	29.44	46.73	85.27	175.77	338.21	549.79	804.59	1001.98	1163.3	1404.61	1654.5	1823.15	1810.06	1636.3	1279.78	831.97	490.58	284.2
Gabon	Female	28.93	53.18	98.7	172.72	276.39	419.2	631.74	822.36	969.49	1118.24	1150.21	1039.3	863.06	710.66	540.99	325.78	159.46	68.1
Gambia	Male	28.36	43.47	83.18	181.72	359.81	574.54	802.94	973.87	1142.04	1404.29	1731.02	2255.59	2898.67	3211.61	2678.31	1738.81	1081.01	657.5
Gambia	Female	27.78	49.81	94.95	169.94	278.18	424.15	634.98	834.46	1024.46	1198.98	1200.53	1065.46	912.8	859.95	813.68	547.92	228.8	79.48
Germany	Male	17.14	24.21	52.87	179.73	585.72	1296.68	2198.47	2933.67	3510.63	4204.16	4776.59	4986.97	4750.4	4444.98	3848.3	2778.19	1806.36	1150.23
Germany	Female	10.38	17	42.38	142.61	371.65	655.05	945.72	1149.95	1296.19	1491.21	1651.99	1687.19	1547.03	1383.05	1147.18	795.09	511.54	309.97
Ghana	Male	28.6	44.13	86.86	200.59	406.93	626.63	842.57	1038.11	1329.49	2030.54	3255.36	4828.53	7133.76	8191.04	6724.7	4956	3612.72	2726.51
Ghana	Female	27.69	49.68	94.43	170.3	279.09	420.79	625.36	827.66	1019.58	1195.76	1200.74	1059.37	908.08	870.58	866.47	608.66	249.6	84.08
Greece	Male	18.29	25.49	55.62	188.94	621.39	1386.41	2345.44	3110.47	3741.9	4470.36	5040.59	5281.57	5052.64	4728.63	4099.17	2992.88	1993.87	1244.41
Greece	Female	11.68	18.68	46.06	157.03	408.31	715.31	1031.82	1251.39	1406.08	1610.47	1763.18	1797.02	1651.41	1467.81	1198.96	791.57	459.86	248.39
Greenland	Male	21.52	40.22	90.93	228.54	479.77	783.32	1124.82	1375.01	1577.58	1900.28	2305.15	2765.42	3146.36	3608.24	3753.78	3279.24	2621.85	1860.84
Greenland	Female	20.23	40.88	115.55	367.38	743.29	1039.05	1260.36	1333.83	1323.43	1393	1481.65	1559.6	1576.9	1643.57	1626.18	1392.93	1128.07	853.77
Grenada	Male	47.09	73.83	130.11	256.52	513.05	921.9	1522.21	2144.84	2934.54	4249.58	5719.41	7141.85	8269.02	9084.91	8792.41	6119.33	2894.66	1370.32
Grenada	Female	34.62	58.03	104.86	193.37	344.24	578	936.31	1274.48	1564.91	1916.53	2160.96	2143.73	1859.41	1434.35	936.75	514.23	289.84	160.86
Guam	Male	33.84	48.61	85.3	184.69	424	857.85	1512.88	2116.25	2636.01	3275.68	3822.07	3985.44	3616.72	3084.06	2454.61	1713.53	1059.65	545.93
Guam	Female	21.26	31.91	56.05	109.56	212.28	386.01	683.97	1006.99	1256.62	1509.03	1749.05	1886.08	1787.76	1531.69	1128.79	651.08	327.16	143.4
Guatemala	Male	49.16	68.99	103.85	188.64	376.59	695.07	1119.83	1466.93	1737.65	1923.69	1980.82	2029.16	1957.67	1860.22	1689.62	1348.43	978.73	681.6
Guatemala	Female	42.37	69.99	87.89	101.54	158.58	296.67	553.91	879.71	1265.43	1667.57	1857.84	1749.72	1427.88	1076.67	751.49	484	325.67	244.4
Guinea	Male	28.38	43.55	82.55	180.34	358.42	574.21	803.1	983.88	1154.21	1404.56	1726.33	2262.68	2896.44	3170.06	2632.04	1722.21	1079.35	647.45
Guinea	Female	27.64	49.63	94.75	172.96	280.32	419.99	626.02	824.46	1010.52	1183.57	1187.27	1047.81	892.28	837.89	779.7	514.87	215.52	75.85
Guinea-Bissau	Male	28.31	43.92	83.05	179.9	360.57	576.69	803.48	971.89	1136.68	1409.4	1740.49	2250.63	2874.57	3143.62	2569.35	1636.8	1011.09	620.75
Guinea-Bissau	Female	27.85	50.01	95.31	173.09	280.05	420.72	629.2	833.69	1035.2	1224.1	1210.18	1045.98	884.82	831.36	789.3	527.53	216	73.3
Guyana	Male	46.51	72.62	127.84	254.02	511.92	918.58	1481.12	2032.6	2709.38	3833.11	5172.91	6213.37	6037.75	4995.83	3711.73	2202.41	1062.91	539.28
Guyana	Female	34.76	58.47	105.83	193.3	345.07	580.45	929.6	1263.15	1573.3	1938.57	2200.98	2160.16	1776.38	1294.16	822.78	445.27	240.91	122.2

Haiti	Male	47	73.36	129.47	253.22	501.93	891.46	1416.15	1902.24	2414.52	3173.22	4049.28	4655.72	4377.81	3631.42	2776.32	1739.6	940.96	507.93
Haiti	Female	35.48	59.52	105.6	190.52	339.96	573.06	918.81	1240.1	1493.22	1788.44	1959.36	1864.62	1521.94	1103.44	709.49	388.41	207.56	100.65
Honduras	Male	48.98	69.02	104.57	189.93	384.31	717.15	1175.91	1599.75	1933.15	2132.95	2282.08	2577.55	3108.81	3623.6	3324.16	2556.93	1870.68	1232.89
Honduras	Female	42.63	70.64	88.99	102.91	159.95	300.04	573.7	930.7	1359.6	1885.28	2278.61	2167.64	1664.77	1253.63	922.32	596.54	371.66	244.51
Hungary	Male	111.95	171.18	264.4	467.79	785.99	1110.77	1434.61	1671.95	1906.13	2325.52	2869.56	3466.74	4093.76	4925.34	5343.17	5133.6	3976.91	2648.51
Hungary	Female	121.42	214.76	405.15	843.39	1287.58	1438.49	1508.93	1549.76	1635.94	1943.31	2343.97	2704.62	2980.36	3172.28	2892.54	2298.75	1569.19	922.53
Iceland	Male	15.87	22.73	49.97	169.72	552.88	1229.35	2092.16	2778.25	3320.7	3978.07	4529.38	4783.47	4632.34	4511.91	4199.17	3338.62	2583.5	1980.54
Iceland	Female	10.16	16.34	40.57	138.67	363.4	640.37	921.34	1116.59	1253.26	1437.59	1587.78	1632.62	1511.22	1359.96	1158.4	813.79	518.48	332.24
India	Male	66.32	115.68	241.11	598.83	1314.91	2221.54	3152.69	3689.38	3967.72	4535.89	5183.3	5595.75	5383.64	4791.41	3847.67	2659.86	1704.08	941.79
India	Female	39.7	72.83	181.95	560.18	1235	1823.95	2255.17	2388.54	2326.46	2500.96	2646.18	2452.96	1948.22	1450.79	1046.41	646.68	332.78	134.76
Indonesia	Male	27.47	38.23	64.67	132.85	345.31	778.14	1459.04	2227.96	3180.99	4517.98	5591.99	5948.86	6245.75	6133.96	5002.88	3797.49	2648.12	1607.59
Indonesia	Female	18.4	25.18	40.38	77.19	164.66	320.35	519.86	635.85	752.45	1074.75	1357.83	1397.03	1284.78	1142.6	920.09	615.27	387.25	244.77
Iran (Islamic Republic of)	Male	65.09	119.93	229.22	440.42	815.43	1383.2	2170.52	2804.7	3216.42	3674.31	3944.15	3778.66	3197.39	2615.59	1994.87	1266.06	719.42	351.81
Iran (Islamic Republic of)	Female	65.31	117.8	202.44	321.32	478.67	695.1	1042.36	1410.14	1788.39	2308.56	2773.31	2857.51	2439.09	1865.96	1267.34	681.48	323.72	139.25
Iraq	Male	51.98	99.77	193.3	365.37	679.37	1187.36	1935.67	2622.61	3067.35	3411.84	3608.53	3461.22	2931	2407.05	1839.97	1181.38	674.85	318.65
Iraq	Female	52.99	100.95	176.37	270.5	385.18	565.4	904.07	1296.25	1690.44	2173.01	2622.87	2744.04	2375.99	1855.53	1295.06	721.27	337.41	129.39
Ireland	Male	18.71	25.92	56.18	191.63	628.46	1391.22	2339.42	3127.62	3754.25	4468.81	5032.19	5235.03	4968.49	4607.41	3952.82	2827.02	1853.19	1247.45
Ireland	Female	11.38	18.45	45.59	155.59	406.65	717.88	1038.73	1264.86	1420.7	1621.77	1787.42	1819.45	1662.85	1521.4	1300.68	883.38	555.29	354.88
Israel	Male	18.66	25.9	55.9	191.32	621.88	1374.51	2325.17	3105.65	3762.08	4519.68	5083.75	5347.78	5205.2	4971.98	4662.79	4082.85	3392.26	2521.62
Israel	Female	11.46	18.58	45.94	156.38	409.39	723.49	1043.7	1260.01	1400.5	1599.23	1769.52	1810.52	1668.55	1509.49	1346.61	1025.7	733.48	540.82
Italy	Male	37.27	52.51	87.52	207.62	537	1086.48	1972.95	3022.7	3928.27	4953.03	5996.37	6449.13	6069.14	5421.55	4443.84	3060.99	1970.59	1225.1
Italy	Female	21.96	34.69	74.59	215.34	501.58	816.87	1117.89	1368.68	1652.27	2073.35	2437.85	2594.16	2411.76	2075.39	1669.69	1121.49	658.65	359.77
Jamaica	Male	47.06	73.33	129.56	256.31	510.71	906.43	1429.71	1904.61	2371.72	3064.7	3978.75	4836.17	5111.61	4830.39	3913.95	2822.43	1679.37	645.03
Jamaica	Female	34.84	58.76	105.29	191.23	339.42	568.35	901.62	1213.45	1480.19	1772.67	1937.97	1863.48	1575.56	1220.9	824.55	477.03	285.37	157.03
Japan	Male	37.53	67.51	120.3	255.19	658.05	1361.56	2407.38	3606.12	4706.4	5637.03	5980.39	5595.88	4753.04	4026.71	3405.67	2641.98	1989.57	1451.12
Japan	Female	24.24	47.79	96.17	183.21	285.66	358.28	459.75	674.18	1089.94	1700.3	2303.58	2529.57	2256.84	1928.39	1670.84	1401.17	1181.62	946.96
Jordan	Male	66.51	146.79	299.89	488.53	846.96	1618.21	2862.67	4065.64	4765.56	5027.11	5051.41	4667.24	3882.22	3258.75	2580.84	1847.29	1218.67	519.73
Jordan	Female	82.45	177.14	317.83	377.31	370.06	481.67	979.04	1590.62	2146.92	3013.32	4152.07	4705.93	4245.9	3326.05	2281.61	1457.1	709.59	167.97
Kazakhstan	Male	97.09	161.68	289.1	624.55	1444.17	2362.67	2821.22	2973.24	3455.6	4366.42	5090.06	5892.98	8470.17	10690.12	9444.97	7995.19	5698.46	2396.2

Kazakhstan	Female	60.93	112.27	251.12	533.17	855.85	1087.77	1339.61	1588.61	1873.2	2318.33	2791.06	3072.09	3172.16	3137.57	2540.41	1995.45	1314.07	480.68
Kenya	Male	26.03	37.45	63.67	124.44	232.58	372.71	536.63	653.35	739.23	892	1066.21	1187.36	1201.39	1146.45	926.22	572.85	312.88	159.61
Kenya	Female	38.13	66.58	119.75	211.66	341.87	510.32	769.53	1088.31	1556.4	1975.65	1846.51	1447.15	1249.54	1424.88	1763.37	1363.12	551.31	221.05
Kuwait	Male	51.77	99.24	192.49	366.08	682.19	1201.32	1951.3	2606.32	3050.61	3415.94	3611.82	3485.71	2976.27	2465.38	1912.71	1246.07	729.06	382.81
Kuwait	Female	53.22	101.48	176.33	271.72	385.44	563.42	905.19	1304.4	1699.2	2181.96	2644.16	2787.01	2427.36	1898.61	1330.38	744.53	349.31	135.59
Kyrgyzstan	Male	111.9	187.09	336.82	724.97	1675.89	2736.33	3264.43	3476.18	4055.26	5024.38	5698.28	6114.71	6395.94	6180.36	4917.87	3384.62	2167.61	1162.14
Kyrgyzstan	Female	69.89	127.36	279.64	594.05	950.35	1204.67	1487.75	1776.17	2084.37	2512.29	2913.61	3063.24	2768.31	2296.29	1739.51	1156.47	748.24	448.57
Lao People's Democratic Republic	Male	36.15	52.71	91.38	191.92	473.43	1034.86	1892.02	2804.8	3869.05	5323.35	6374.36	6573.43	6686.32	6341.54	4972.94	3805.49	2607.13	1376.64
Lao People's Democratic	Female	21.77	33.73	56.86	106.87	214.45	396.67	633.97	796.05	965.09	1325.15	1631.03	1703.45	1624.5	1443.33	1060.55	626.91	358.84	208.5
Latvia	Male	69.42	101.24	197.46	557.02	1523.45	3078.55	5258.47	7517.8	9496.19	11685.95	13780.11	15269.98	18222.21	21671.18	19595.71	18716.29	16184.04	9955.11
Latvia	Female	44.61	76.12	192.02	571.38	1207.67	1823.09	2413.65	2941.34	3686.29	4985.41	6471.51	7528.74	7448.16	6859.42	5551.68	4381.23	3132.21	1692.69
Lebanon	Male	52.19	99.76	191.82	364.48	679.11	1191.29	1929.71	2598.84	3051.59	3413.72	3609.51	3476.62	2973.49	2466.7	1909.89	1238.76	712.07	338.71
Lebanon	Female	53.06	101.01	176.03	269.69	383.1	562.7	901.7	1295.49	1690.36	2179.44	2641.48	2792.57	2445.85	1912.91	1340.12	755.44	356.08	136.14
Lesotho	Male	38.74	67.11	143.76	351.75	682.28	957.7	1189.17	1382.53	1513.9	1668.36	1839.4	2021.85	2098.33	1998.97	1531.81	869.95	466.8	290.02
Lesotho	Female	27.93	55.82	109.92	174.66	245.04	366.21	588.6	799.94	932.45	1037.54	1045.46	942.86	798.71	685.28	554.33	359.75	183.75	75.59
Liberia	Male	28.38	43.78	83.69	181.83	359.3	573.23	801.31	976.48	1141.25	1412.42	1748.81	2261.89	2899.5	3201.77	2675.73	1753.54	1090.86	662.01
Liberia	Female	27.68	49.42	94.86	171.5	279.15	421.54	629.81	831.76	1024.86	1209.35	1214.06	1063.23	897.21	850.27	836.86	578.94	236.86	79.35
Libya	Male	52.03	99.6	194.46	368.62	677.01	1175.02	1912.12	2586.94	3045.6	3400.59	3589.65	3435.98	2908.7	2408.45	1863.99	1203.48	682.8	320.6
Libya	Female	53.06	101.17	175.93	270.35	385.3	563.89	900.25	1292.4	1676.59	2150.3	2611.5	2752.64	2385.07	1846.12	1282.8	713.94	333.77	128.86
Lithuania	Male	73.52	106.96	208.5	581.23	1594.18	3221.49	5482.76	7833.34	9799.63	11944.31	13867.21	14806.89	14727.55	14129.9	11171.21	8115.09	5795.97	2810.06
Lithuania	Female	52.56	88.62	223.92	658.3	1387.66	2088.21	2750.33	3358.3	4181.24	5573.85	7137.4	8113.98	7914.93	7216.03	5537.52	3919.16	2774.47	1318.47
Luxembourg	Male	21.36	29.2	62.17	213	693.81	1548.88	2641.55	3542.37	4250.2	5025.69	5642.56	5888.2	5598.34	5187.8	4427.66	3157.33	2061.03	1334.25
Luxembourg	Female	12.81	20.31	50.86	174.45	458.61	807.05	1162.02	1406.47	1581.6	1806.59	1969.06	2006.94	1844.74	1626.36	1311.38	853.52	490.07	259.51
Madagascar	Male	18.97	25.43	44.73	98.19	198.24	324.41	470.96	591.12	687.53	809.95	935.12	1052.54	1117.7	1106.8	906.69	568.92	316.01	161.86
Madagascar	Female	29.16	53.25	100.8	178.25	283	422.89	648.55	944.78	1412.56	1793.01	1603.09	1201.83	1006.3	1141.13	1550.73	1277.33	484.49	152.93
Malawi	Male	19.2	25.64	44.64	98.66	200.23	328.24	477.82	595.73	683.19	806.51	939.19	1064.18	1127.02	1102.4	896.17	565.23	319	169.48
Malawi	Female	28.95	53.12	100.52	179.84	285.96	424.18	650.54	948.37	1382.98	1737.3	1587.86	1210.58	1007.27	1155.65	1653.67	1384.66	501.54	150.17
Malaysia	Male	35.91	52.34	90.34	189.57	465.46	1005.34	1849.67	2736.54	3682.79	4945.42	5979.63	6414.44	6391.43	6089.16	5012.95	3506.42	2273.58	1312.32

Malaysia	Female	22.03	33.49	55.87	105.99	212.13	394.12	632.13	794.27	961.49	1312.96	1645.45	1733.62	1621.97	1488.53	1201.35	779.37	468.17	289.31
Maldives	Male	36.01	52.95	90.89	190.28	468.73	1009.11	1858.79	2763.61	3754.46	5027.48	6018.23	6437.24	6462.01	6276.68	5322.29	3805.7	2440.34	1381.49
Maldives	Female	21.69	33.43	56	104.23	209.2	391.56	631.37	793.07	953.37	1310.96	1635.66	1711.49	1606.17	1470.56	1201.99	807.97	507.38	306.3
Mali	Male	28.57	44.09	83.89	181.74	357.18	573.44	804.01	974.16	1141.85	1412.75	1738.13	2268.81	2918.38	3217.51	2703.33	1783.54	1114.94	655.73
Mali	Female	27.64	49.68	94.87	172.62	279.36	420.88	626.79	827.87	1021.88	1199.59	1199.57	1054.61	902.47	849.27	811.48	552.16	227.1	78.12
Malta	Male	20.77	28.59	61	210.1	691.36	1525.84	2584.26	3456.57	4134.37	4917.35	5571.78	5872.58	5699.66	5499.59	5040.06	3918.6	2851.73	2058.55
Malta	Female	11.91	19.01	46.96	161.83	426.21	753.66	1075.13	1297.36	1472.39	1698.04	1864.35	1912.72	1774.62	1618.28	1407.5	1004.77	627.64	363.7
Marshall Islands	Male	34.04	49.01	85.94	184.87	422.8	861.07	1521.09	2131.79	2674.03	3314.65	3870.66	4120.91	3692.03	2916.18	2196.49	1763.01	1237.86	590.62
Marshall Islands	Female	21.41	32.46	56.68	109.39	211.19	389.42	713.92	1100.55	1372.5	1572.67	1869.68	2075.47	1934.89	1662.95	1253.04	700.45	298.63	111.64
Mauritania	Male	28.64	44.17	83.82	181.45	359.05	575.27	806.8	981.78	1145.77	1411.53	1733.37	2273.72	2958.51	3308.87	2796.49	1842.29	1163.24	684.34
Mauritania	Female	27.88	50.14	96.04	174.38	281.99	423.6	633.3	837.58	1041.21	1228.32	1223.29	1074.89	914.81	879.56	990.44	758.7	293.03	93.23
Mauritius	Male	35.9	52.18	89.58	187.89	464.63	1005.48	1845.61	2742.89	3738.03	5005.49	6026.62	6470.29	6450.3	6298.5	5412.01	3858.11	2504.47	1460.75
Mauritius	Female	21.73	33.27	55.95	106.06	212.24	394.44	637.05	799.34	960.72	1312.98	1632.18	1708.32	1599.09	1475.37	1209.15	807.02	510.64	311.85
Mexico	Male	55.08	75.21	124.39	253.07	536.45	1021.62	1715.49	2379.45	2889.53	3253.02	3548.16	3713.94	3642.48	3449.93	2966.48	2292.5	1613.89	1094.13
Mexico	Female	44.06	78.02	111.89	139.76	219.41	407.41	754.88	1225.18	1857.51	2652.23	3259.32	3169.4	2450.72	1742.22	1183.01	730.8	476.66	350.84
Micronesia (Federated States of)	Male	33.62	47.81	84.19	184.54	421.58	856.32	1507.17	2118.87	2668.33	3291.21	3826.75	4016.57	3576.24	2907.62	2225.61	1699.93	1168.79	583.38
Micronesia (Federated States of)	Female	21.23	32.36	56.88	110.55	212.37	387.68	700.69	1055.94	1319.21	1540.65	1801.4	1970.76	1855.68	1581.87	1163.42	658.28	299.1	116.14
Mongolia	Male	97.3	161.23	289.74	629.77	1458.8	2365.57	2792.59	2910.62	3274.03	3962.31	4558.51	5097.38	5487.17	5611.79	4997.37	3788.16	2482.01	1276.31
Mongolia	Female	61.13	113.27	252.32	532.9	854.74	1085.63	1334.59	1587.79	1859.43	2251.53	2619.34	2749.2	2510.32	2133.99	1659.75	1127.2	708.53	380.97
Montenegro	Male	112.04	170.08	260.7	460.13	776.58	1097.4	1418.1	1651.1	1868.67	2226.89	2593.8	2908.89	3108.95	3359.3	3328.47	2819.76	2236.21	1657.34
Montenegro	Female	121.27	214.68	406.56	840.98	1287.34	1433.82	1491.27	1530.08	1612.45	1883.52	2210.72	2510.6	2652.19	2729.8	2538.07	1961.64	1386.61	916.76
Morocco	Male	52	99.46	191.34	363.08	673.97	1184.41	1937.27	2599.48	3018.76	3368.12	3556.51	3413.9	2920.54	2413.89	1857.64	1201.4	681.7	315.34
Morocco	Female	52.99	101.31	176.79	267.94	380.73	563.74	905.38	1299.05	1681.19	2158.89	2608.13	2726	2368.12	1842.21	1284.13	723.75	339.7	130.37
Mozambique	Male	18.9	25.45	44.65	98.67	201.19	325.99	470.32	595.65	688.49	808.89	941.17	1062.12	1126.26	1105.37	897.49	563.61	315.8	167.5
Mozambique	Female	28.97	53.1	101.1	181.19	286.43	424.75	652.41	962.63	1527.69	2001.54	1729.97	1223.62	1011.79	1160.82	1716.11	1451.84	508.5	147.39

Myanmar	Male	35.8	52.19	90.81	193.22	474.3	1030.51	1901.21	2879.23	4076.97	5715.71	6984.7	7423.58	7936.47	7600.59	5781.26	4374.66	2971.02	1527.55
Myanmar	Female	21.6	33.29	56.67	106.89	215.44	397.84	637.34	805.71	986.58	1366.8	1690.47	1828.59	1889.26	1773.73	1295.57	764.11	466.46	292.21
Namibia	Male	39.08	67.38	143.34	349.74	675.23	937.4	1164.33	1355.33	1484.86	1625.13	1778.34	1972.8	2094.03	2061.85	1607.69	925.04	512.53	323.79
Namibia	Female	27.89	55.99	110.86	176.58	245.57	366.28	596.35	811.2	935.84	1036.89	1046.44	949.62	817.78	714.19	580.58	377.86	195.26	82.02
Nepal	Male	48.84	89.27	191.38	457.91	979.73	1628.75	2315.52	2831.8	3222.09	3755.97	4289.71	4717.12	4822.94	4488.56	3504.57	2351.05	1541.34	911.2
Nepal	Female	31.74	60.63	147.86	415.49	904.35	1348.22	1700.43	1891.86	1947.73	2092.19	2163.31	2010.34	1621.91	1218.28	882.59	538.78	270.67	125.63
Netherlands	Male	18.49	25.81	56.34	191.96	623.61	1377.66	2332.26	3121.64	3763.63	4531.03	5171.78	5413.48	5225.46	5084.83	4629.88	3841.72	3567.41	3176.48
Netherlands	Female	11.44	18.18	45.11	154.67	405.43	720.43	1041.63	1265.57	1422.6	1629.95	1818.26	1887.88	1764.77	1646.82	1510.14	1206.12	948.18	704.71
New Zealand	Male	18.76	24.48	50.31	169.48	523.18	1125.99	1883.59	2513	3080.02	3843	4494.66	5029.51	5149.1	4996.76	4733.87	3982.43	3100.64	2182.45
New Zealand	Female	8.38	12.46	39.5	145.16	422.61	789.2	1074.8	1227.57	1375.86	1585.6	1705.42	1736.21	1679.99	1546.81	1296.09	951.81	679.42	446.33
Nicaragua	Male	49.12	69.11	104.14	189.72	376.01	686.56	1105.74	1459.42	1723.91	1912.82	1959.68	1960.71	1900.97	1873.93	1745.24	1420.12	1059.71	732.11
Nicaragua	Female	42.68	70.38	87.78	99.82	154.72	289.97	542.56	864.72	1259.63	1676.14	1871.57	1760.46	1416.77	1070.02	787	545.77	414.93	337.21
Niger	Male	28.81	44.56	83.92	179.02	355.82	573.8	805.65	983.46	1152.62	1418.01	1744.15	2263.15	2892.7	3195.43	2686.1	1765.82	1102.49	650.4
Niger	Female	27.89	49.85	95.75	174.59	280.99	421.63	628.19	824.92	1012.54	1188.68	1193.45	1056.23	905.4	850.52	782.11	510.75	213.5	75.29
Nigeria	Male	37.94	60.02	108.68	217.27	408.04	646.66	908.97	1091.55	1256.99	1579.33	2028.53	2564.45	2960.7	3106.13	2608.97	1665.31	961.33	513.13
Nigeria	Female	36.94	63.49	113.42	202.02	332.27	496.03	713.73	900.79	1053.09	1225.17	1285.69	1190.49	1032.78	934.86	762.19	452.37	214.37	94.01
North Macedonia	Male	111.92	169.7	261.29	463.77	777.79	1095.25	1409.42	1634.4	1856.54	2231.51	2619.95	2920.79	3101.76	3363.29	3319.52	2816.32	2254.44	1662.22
North Macedonia	Female	121.63	215.66	406.79	845.52	1290.59	1433.01	1489.55	1526.57	1608.93	1882.79	2218.16	2526.05	2666.44	2737	2551.9	1992.34	1411.7	923.58
Northern Mariana Islands	Male	33.72	48.28	84.14	182.65	421.83	866.03	1526.55	2126.6	2660.82	3296.18	3821.04	4001.69	3634.01	3081.16	2453.43	1724.1	1080.65	565.31
Northern Mariana Islands	Female	21.1	32.04	56.36	109.31	211.81	388.92	684.5	999.36	1252.52	1514.56	1783.25	1928.51	1804.13	1537.82	1156.85	681.97	328.67	138.73
Micronesia (Federated States of)	Male	33.62	47.81	84.19	184.54	421.58	856.32	1507.17	2118.87	2668.33	3291.21	3826.75	4016.57	3576.24	2907.62	2225.61	1699.93	1168.79	583.38
Micronesia (Federated	Female	21.1	32.04	56.36	109.31	211.81	388.92	684.50	999.36	1252.52	1514.56	1783.25	1928.51	1804.13	1537.82	1156.85	681.97	328.67	138.73

States of)

Norway	Male	48.09	71.02	145.08	406.68	1137.9	2462.12	4238.5	5635.93	6685.22	8085.26	9342.03	9756.72	9219.77	8784.5	8175.17	6877.1	5648.4	4457.68
Norway	Female	27.78	46.1	100.87	270.71	645.17	1155.16	1686.93	2016.25	2230.98	2599.51	2952.16	3042.57	2803.88	2619.6	2403.9	1822.1	1203.56	777.31
Oman	Male	51.95	99.63	192.94	366.12	679.11	1190.47	1942.49	2612.98	3050.34	3396.5	3585.48	3473.08	2970.89	2455.76	1931.96	1307.97	788.41	384.12
Oman	Female	53.06	101.02	176.4	271.5	384.43	562.57	905.09	1300.71	1694.1	2173.3	2632.28	2771.43	2394.89	1844.85	1283.27	732.22	352.2	136.03
Pakistan	Male	62.77	114.93	239.12	536.92	1090.06	1789.8	2542.38	3044.28	3398.1	4006.19	4612.49	4861.46	4476.98	3727	2679.91	1635.34	1052.56	659.58
Pakistan	Female	42.57	82.25	189.37	464.03	949.89	1459.36	1873.89	2037.06	2063.43	2223.72	2315.81	2137	1701.95	1266.35	854.03	460.78	223.17	93.45
Palestine	Male	52.11	100.67	194.46	366.51	674.79	1178.36	1934.14	2610.02	3039.4	3376.8	3550.16	3433.23	2943.83	2433.9	1873.06	1197.88	695.31	370.96
Palestine	Female	53.26	101.84	176.75	271.01	384.23	562.99	901.09	1297.19	1688.31	2165.05	2613.79	2755.01	2390.45	1836.62	1266.81	702.07	329.02	127.1
Panama	Male	48.94	68.79	104.08	190.6	378.18	691.14	1106.56	1446.73	1711.28	1907.3	1955.13	1952.43	1883.92	1854.53	1744.54	1439.38	1090.62	776.21
Panama	Female	42.66	69.94	87.16	100.24	155.62	288.51	539.56	868.32	1264.75	1668.35	1856.21	1744.72	1411.83	1081.69	795.77	551.6	416.42	317.57
Papua New Guinea	Male	34.05	49.2	86.15	183.76	421.83	862.77	1513.98	2110.68	2631.22	3249.1	3746.49	3878.72	3438.87	2828.73	2179.09	1496.9	932.7	462.8
Papua New Guinea	Female	21.65	32.72	57.22	110.58	212.71	387.76	693.78	1046.52	1306.27	1514.14	1741.81	1859.72	1714.27	1412.21	987.01	551.42	274.19	116.39
Paraguay	Male	31.47	50.88	96.99	217.81	493.27	900.47	1370.61	1721.56	1969.47	2272.97	2551.65	2712.09	2710.13	2674.72	2406.16	1959.11	1640.06	1337.25
Paraguay	Female	22.91	40.92	101.93	279.88	567.52	866.02	1176.02	1369.67	1456.78	1585.72	1639.68	1559.05	1374.03	1204.67	1009.5	759.07	569.4	434.08
Peru	Male	38.68	56.3	104.88	273.71	797.07	1836.41	3230.05	4239.16	4772.42	5216.15	5276.8	4814.94	4032.24	3355.04	2661.61	1888.99	1322.95	910.94
Peru	Female	24.42	40.4	87.24	235.91	566.52	1056.83	1681.12	2192.63	2583.14	3003.81	3120.99	2814.49	2258.14	1736.35	1249.42	774.8	486.78	300.67
Philippines	Male	40	63.97	129.36	317.39	788.45	1686.1	3154.41	4885.47	7090.07	10289.01	12913.64	13669.05	13999.08	13475.74	10813.23	7917.46	5234.79	2896.14
Philippines	Female	23.8	43.51	89.57	183.02	342.21	584.05	967.93	1373.89	1869.09	2470.74	2840.72	3048.59	3038.75	2782.81	2096.71	1250.12	774.69	499.13
Poland	Male	104.5	130.97	183.17	325.72	542.85	722.23	883.49	1026.16	1157.77	1370.97	1603.72	1835.81	2026.74	2214.69	2201.34	1913.32	1552.42	1187.32
Poland	Female	114.36	168.87	296.21	673.21	1010.9	1028.47	1016.03	1052.49	1108.89	1273.42	1496.65	1726.69	1860.17	1924.55	1820.73	1453.1	1050.66	694.63
Portugal	Male	16.45	27.56	70.03	191.09	621.59	1027.96	1286.39	2551.34	3352.51	3482.37	4740.27	5125.56	4255.49	3591.19	3054.56	2436.42	1827.55	1422.21
Portugal	Female	10.05	16.28	40.84	151.69	528.28	830.47	841.41	1187.68	1893.86	2256.39	2048.78	1858.29	2003.44	1716.04	1194.14	929.96	655.23	416.16
PuertoRico	Male	46.9	73.39	130.12	256.13	506.62	900.55	1424.41	1903.2	2407.64	3101.68	3850.76	4404.84	4337.96	3911.81	3121.01	1979.23	1119.06	600.16
PuertoRico	Female	34.43	57.94	104.36	192.22	340.47	570.55	902.31	1200.53	1474.59	1787.99	1951.82	1881.22	1581.95	1203.29	824.34	485.41	278.15	150.43
Qatar	Male	51.93	99.73	193.59	366.63	677.91	1187.03	1942.64	2602.08	3034.66	3412.31	3608.6	3480.92	2979.85	2465.16	1922.51	1252.88	715.08	345.18
Qatar	Female	52.59	100.47	174.95	269.08	386.63	569.07	912.08	1312.16	1705.46	2176.72	2626.96	2769.6	2401.85	1857.46	1292.5	726.86	348.25	142.2

Republic of Korea	Male	32.92	62.76	120.72	273.35	680.11	1357.11	2381.43	3513.61	4541.15	5391.29	5648.63	5245.24	4400.66	3713.57	3147.02	2471.99	2050.6	1866.09
Republic of Korea	Female	23.95	48.04	94.16	180.91	284.66	362.78	489.59	723.43	1115.29	1660.19	2182.26	2384.54	2126.89	1820.23	1569.58	1239.87	934.63	732.55
Republic of Moldova	Male	71.25	103.79	201.89	569.24	1573.39	3168.79	5376.47	7670.78	9754.09	11942.1	13868.68	14909.94	14643.38	13844.24	11251.96	7478.78	4627.26	2607.08
Republic of Moldova	Female	48.68	82.99	206	615.23	1304.17	1969.78	2605.35	3164.18	3951.08	5339.87	6851.65	7873.68	7737.46	7061.25	5675.1	3588.12	2047.6	1019.82
Romania	Male	112.53	170.29	261.3	463.3	782.25	1101.65	1417.62	1650.55	1858.15	2207.2	2587.57	2924.53	3128.14	3334.37	3264.31	2739.43	2117.34	1511.85
Romania	Female	137.75	241.18	451.26	933.15	1429.97	1600.18	1660.84	1704.26	1804.49	2099.73	2467.55	2794.37	2929.79	3005.63	2777.91	2132.36	1498.61	983.48
Russian Federation	Male	89.72	139.9	277.75	704.75	1761.74	3544.65	6108.79	8560.65	10654.36	13392.88	16341.79	18213.8	18413.3	18691.24	17475.71	13628	9045.16	5231.32
Russian Federation	Female	65.06	115.05	264.04	670.6	1395	2190.57	2987.21	3609.96	4328.76	5726.91	7338.55	8425.54	8418.81	7979.07	6763.61	4625.34	2764.45	1395.13
Rwanda	Male	18.89	25.33	44.38	96.52	196.75	323.26	469.5	589.41	682.15	807.15	935.83	1056.57	1124.61	1113.43	910.66	573.82	324.33	169.17
Rwanda	Female	28.95	52.99	100.04	178.8	285.58	427.06	652.19	948.12	1386.6	1740.03	1587.67	1218.15	1021.46	1162.04	1575.62	1297.2	493.18	152.54
Saint Lucia	Male	46.45	72.97	129.71	255.69	505.16	899.09	1429.2	1900.08	2367.51	3040.15	3780.85	4303.57	4239.49	3811.12	2999.68	1889.95	1044.84	528.06
Saint Lucia	Female	35.24	59.58	106.67	194.75	348.78	595.34	967.71	1324.88	1693.9	2174.78	2543.46	2675.94	2451.59	1916.44	1235.56	680.37	435.2	294.72
Saint Vincent and the Grenadines	Male	46.97	73.35	129.83	256.14	509.74	907.29	1466.29	2016.48	2684.7	3762.03	5018.84	5869.2	5660.97	4846.9	3912.5	2613	1355.91	844.09
Saint Vincent and the Grenadines	Female	34.9	58.53	104.16	191.05	338.6	565.96	889.32	1187.51	1461.75	1769.24	1941.76	1859.98	1546.97	1189.28	824.17	481.25	268.12	138.79
Samoa	Male	33.53	48.32	84.57	184.92	420.32	863.33	1529.09	2131.2	2649.06	3265.46	3787.69	3959.12	3558.1	2953.38	2327.65	1842.68	1283.71	642.37
Samoa	Female	21.26	32.32	56.96	110.31	211.7	387.73	703.75	1065.21	1333.13	1555.18	1817.87	2003.36	1957.5	1781.25	1416.78	828.24	342.83	129.61
Sao Tome and Principe	Male	28.55	44.03	83.67	181.56	360.58	576.43	806.41	980.1	1146.06	1416.36	1762.57	2309.17	2978.03	3279.8	2744.86	1830.87	1157.12	689.48
Sao Tome and Principe	Female	28.05	50.13	95.65	174.06	282.97	426.47	635.58	835.52	1042.46	1237.98	1228.83	1075.55	917.87	876.3	896.69	641.74	261.56	88.8
Saudi Arabia	Male	52.16	99.66	192.14	364.25	678.68	1195.38	1954.41	2622.31	3058.07	3408.38	3591.12	3468.99	2953.3	2424.64	1883.5	1229.97	705.08	331.34

Saudi Arabia	Female	53.02	101.84	177.27	269.34	383.25	566.98	908.69	1302.23	1697.59	2181.85	2639.91	2772.46	2410.88	1883.66	1306.32	723.58	339.58	131.91
Senegal	Male	28.8	44.54	83.75	179.44	356.84	574.6	802.77	974.4	1141.94	1414.45	1742.36	2262.5	2910.77	3224.86	2706.25	1787	1125.75	661.7
Senegal	Female	27.75	49.68	95.24	171.06	277.94	419.34	623.81	830.24	1026.35	1197.11	1200.2	1063.71	910.05	855.29	799.88	533.45	224.43	79.96
Serbia	Male	102.4	156.52	241.61	428.19	716.16	1008.3	1310.39	1534.19	1745.84	2087.91	2423.89	2725.28	2901.41	3052.47	2898.34	2323.28	1700.74	1145.25
Serbia	Female	126.96	224.07	421.93	871.91	1330.33	1482.71	1545.14	1591.01	1687.29	1972.79	2311.2	2592.37	2668.9	2657.68	2372.06	1740.62	1149.75	699.55
Seychelles	Male	35.97	52.67	90.66	191.02	473.19	1020.92	1879.23	2809.59	3859.02	5252.32	6306.27	6731.11	7275.57	7147.58	5678.19	4565.67	3377.07	1936.63
Seychelles	Female	21.86	33.32	55.75	105.02	213.92	398.73	639.42	804.78	973.23	1332.84	1646.36	1749.3	1679.29	1521.58	1213.93	816.89	534.84	354.64
Sierra Leone	Male	28.5	43.82	83.5	181.51	360.22	577.3	806.8	981.56	1138.98	1388.09	1710.33	2236.65	2915.08	3238.05	2690.89	1757.91	1100.79	654.27
Sierra Leone	Female	27.78	49.65	94.88	171.54	277.77	419.66	625.89	831.04	1027.7	1199.1	1200.03	1060.25	898.79	844.35	791.34	526.78	221.86	76.81
Singapore	Male	33.26	63.04	121.65	274.1	680.9	1361.42	2396.27	3529.06	4526.76	5383.16	5695.28	5335.47	4545.91	3886.3	3238.13	2349.32	1584.56	1028.29
Singapore	Female	23.94	47.67	94.1	179.78	285.09	368.27	492.54	724.05	1117.34	1662.25	2187.52	2387.92	2138.73	1838.61	1577.54	1230.92	904.53	607.23
Slovakia	Male	130.21	195.73	296.11	523.06	878.86	1241	1607.41	1874.55	2118.98	2521.51	2937.07	3314.67	3547.93	3727.59	3506.05	2805.96	2069.8	1373.31
Slovakia	Female	137.12	242.84	455.33	941.67	1443.79	1600.98	1659.08	1715.33	1815.64	2108.6	2471.95	2774.33	2849.09	2814.73	2462.55	1756.72	1127.53	656.39
Slovenia	Male	110.76	169.48	260.66	462.2	775.19	1094.88	1415.14	1641.73	1862.67	2235.03	2607.18	2919.18	3110.57	3303.18	3166.85	2570.52	1898.24	1365.76
Slovenia	Female	108.62	192.73	361.45	756.86	1164.33	1300.53	1352.11	1388.02	1466.78	1702.82	1995.25	2265.66	2366.31	2379.58	2137.4	1560.06	1023.06	611.93
Solomon Islands	Male	34.17	49.32	86.16	185.1	426.28	863.6	1509.54	2130.46	2683.64	3293.09	3830.03	4041.47	3579.89	2868.4	2170.71	1607.1	1064.34	507.56
Solomon Islands	Female	21.54	32.47	56.77	110.4	212.97	392.91	740.7	1170.89	1435.98	1576.1	1766.36	1897.62	1767.9	1465.41	1028.95	574.56	285.65	120.25
Somalia	Male	19.26	25.97	45.13	99.27	201.44	327.19	471.14	588.71	682.04	807.22	944.98	1064.65	1114.44	1089.94	880.4	542.96	299.81	158.38
Somalia	Female	29.01	52.85	100.25	179.87	284.94	423.83	649.43	948.61	1453.78	1878.4	1660.86	1208.88	999.28	1129.04	1593.25	1319.11	466.37	135.22
South Africa	Male	50.5	88.38	180.71	394.27	735.42	1069.93	1360.2	1522.57	1616.09	1824.34	2069.25	2250.05	2232.65	2053.01	1569.32	915.6	510.32	302.08
South Africa	Female	37.29	68.7	123.69	203.98	307.32	450.23	672.69	862.37	981.31	1106.16	1150.36	1060.43	892.11	746.04	572.16	346.89	178.38	83.6
South Sudan	Male	19.14	25.69	44.75	98.22	198.08	326.56	476.08	594.58	682.15	802.76	934.18	1053.75	1116.53	1096.43	889.6	555.16	307.87	158.91
South Sudan	Female	28.8	52.36	100.19	178.36	283.85	426.05	654.42	946.16	1379.39	1743.55	1588.28	1205.39	1008.11	1139.48	1574.28	1301.43	477.35	144.62
Spain	Male	18.29	25.53	55.77	191.28	622.48	1380.88	2343.16	3131.9	3763.8	4498.08	5109.18	5341.95	5091.82	4749.56	4084.58	2952.37	2069.89	1466.48
Spain	Female	11.57	18.56	45.63	155	405.21	720.24	1039.38	1259.7	1417.95	1630.48	1801.03	1834.42	1668.5	1467.71	1214.45	830.85	533.57	348.62
Sri Lanka	Male	36.12	52.86	90.76	188.7	462.54	1005.34	1857.55	2743.52	3722.53	5002.84	6015.5	6468.91	6504.66	6352.22	5482.16	4019.71	2643.34	1514.08
Sri Lanka	Female	21.89	33.11	55.5	104.98	211.63	395.89	636.13	796.8	960.77	1314.63	1630.69	1702.88	1599.3	1468.85	1196.16	805	511.92	308.13
Sudan	Male	52.09	100.22	193.17	366.59	677.68	1185.81	1928.12	2577.94	3004.64	3361.07	3544.07	3401.93	2892.16	2372.5	1816.54	1152.39	641.47	296.44
Sudan	Female	53.12	101.38	177.74	269.23	381.71	560.78	894.49	1284.45	1680.15	2151.67	2578.14	2703.36	2355.14	1817.18	1255.84	703.51	324.19	121.9

Suriname	Male	47.15	73.14	129.09	256.38	508.05	912.76	1490.26	2071.74	2783.36	4094.71	6174.6	8108.9	8256.45	7080.75	5529.84	3582.65	1843.77	938.66
Suriname	Female	35.26	58.94	104.84	191.74	341.89	572.04	903.14	1205.08	1472.71	1765.43	1921.9	1849.87	1530.91	1142.34	769.17	440	241.11	120.79
Sweden	Male	36.23	63.82	121.42	267.81	594.66	1101.97	1758.8	2382.04	3056.91	3954.27	4654.89	4908.01	4905.16	5091.33	5028.67	4579.28	4295.04	3816.5
Sweden	Female	23.9	45.2	98.37	221.61	394.04	541.44	719.62	961.41	1316.66	1772.69	2102.15	2159.27	1937.53	1715.75	1478.14	1098.34	788.46	569.36
Switzerland	Male	20.53	28.07	60.13	208.03	684.2	1528.72	2583.28	3409.86	4098.05	4891.94	5512.06	5748.19	5501.91	5145.66	4389.06	3224.72	2410.16	1618.45
Switzerland	Female	12.08	19.14	47.54	162.4	428.59	760.86	1085.07	1309.2	1482.24	1705.15	1864.13	1889.75	1732.77	1533.86	1257	840.54	500.14	279.84
Syrian Arab Republic	Male	52.01	99.64	191.85	364.48	677.82	1189.07	1950.52	2630.43	3047.42	3391.57	3600.45	3477.23	2957.75	2426.73	1887.87	1229.19	695.25	326.24
Syrian Arab Republic	Female	52.79	100.63	175.95	270.53	385.91	565.24	907.07	1290.73	1674.87	2164.11	2632.98	2776.8	2413.08	1882.68	1315.86	735.76	342.16	129.75
Tajikistan	Male	97.04	162.69	293.47	641.71	1542.52	2624.51	3139.4	3127.2	3337.43	4043.97	4764.8	5549.05	5869.1	5273.3	4733.68	4005.24	2507.63	1300.29
Tajikistan	Female	61.4	113.34	250.25	532.68	856.52	1097.91	1355.56	1587.46	1849.95	2246.31	2631.46	2791.62	2548.63	2099.14	1562.77	1092.55	718.34	366.57
Thailand	Male	36.03	52.5	89.2	186.4	462.89	1013.91	1871.43	2798.93	3898.78	5356.28	6721.99	7627.34	8668.35	9302.82	8333.39	7108.74	5009.14	2770.9
Thailand	Female	21.53	32.86	55.33	104.15	210.73	394.5	639.66	804.75	966.63	1325.39	1653.76	1761.81	1800.04	1889.38	1797.87	1588.42	1212.91	807.48
Timor-Leste	Male	36.12	52.6	90.74	191.71	475.3	1026.16	1867.58	2763.74	3744.82	5016.12	5921.88	6173.47	6185.21	5636.89	4381.25	3444.41	2424.09	1293.67
Timor-Leste	Female	21.85	33.76	56.75	107.21	213.73	393.78	627.33	790.2	959.33	1309.02	1619.66	1701.21	1621.88	1445.35	1073.15	643.17	382.56	243.93
Togo	Male	28.76	44.35	84.25	182.02	359.92	573.78	797.35	973.18	1146.5	1406.87	1737.99	2266.79	2912.8	3219.48	2684.61	1751.9	1092.75	657.41
Togo	Female	27.68	49.39	94.66	172.29	279.71	420.56	629.43	830.52	1011.4	1189.03	1200.76	1062.52	912.11	865.18	810.79	537.28	226.22	80.77
Tonga	Male	33.81	48.77	86.12	185.54	424.37	861.59	1509.94	2125.97	2675.77	3312.79	3863.77	4084.81	3676.06	2982.46	2332.38	1968.94	1446.77	736.84
Tonga	Female	21.51	32.65	56.62	109.2	211.87	386.78	678.93	990.61	1242.93	1497.5	1742.97	1880.21	1759.75	1480.7	1075.77	618.88	313.33	137.14
Trinidad and Tobago	Male	46.82	73.05	129.47	254.47	503.8	895.34	1426.22	1946.96	2823.7	4700.64	7430.41	10266.46	11680.88	10629.43	7942.63	4858.63	2481.15	1528.66
Trinidad and Tobago	Female	34.74	58.63	104.64	190.54	340.39	574.37	930.54	1290.83	1703.85	2245.22	2612.82	2446	1869.16	1381.2	1016.53	714.39	478.48	242.27
Tunisia	Male	52.16	99.66	192.11	364.65	678.71	1191.39	1937.79	2615.68	3068.96	3421.53	3631.85	3497.19	2954.68	2429.42	1891.6	1240.41	707.46	331.43
Tunisia	Female	53.25	100.9	175.12	268.18	382.23	561.89	903.07	1301.12	1693.89	2176.14	2635.85	2783.59	2443.81	1909.87	1332.19	748.51	347.45	133.77
Turkey	Male	51.9	99.75	193.22	367.64	685.57	1203.62	1948.98	2608.58	3067.11	3452.34	3664.64	3557.76	3066.77	2573.09	2080.81	1589.11	1088.62	538.49
Turkey	Female	53.37	100.92	175.31	268.92	383.05	562.83	909.6	1303.89	1682.01	2172.78	2634.09	2757.68	2378.53	1831.13	1290.51	758.38	389.34	166.97
Turkmenistan	Male	97.67	162.34	291.04	629.93	1443.97	2353.57	2807.14	2936.66	3324.7	4087.41	4697.48	5123.52	5415.04	5246.45	4222.45	2916.13	1823.75	975.02
Turkmenistan	Female	60.75	113.09	252.09	533.79	855.17	1095.67	1361.64	1602.24	1872.18	2268.01	2624.04	2797.01	2570.22	2114.13	1562.28	993.65	610.88	357.64
Uganda	Male	18.97	25.37	44.33	97.85	198.07	323.82	472.19	595.5	685.89	808.75	942.96	1062.88	1123.25	1112.85	914.07	571.78	320.64	168.07

Uganda	Female	28.68	52.38	99.97	177.87	284	425.38	653.37	950.92	1361.38	1697.53	1572.9	1218.28	1014.48	1149.79	1519.84	1230.79	476.71	155.91
Ukraine	Male	89.79	139.29	274.78	696.35	1757.75	3561.35	6255.55	8938.66	11192.75	13869.71	16506.1	17380.16	16139.41	14624.01	11231.53	6407.84	3311.79	1657.88
Ukraine	Female	65.2	114.66	260.36	663.36	1379.81	2168.16	2947.42	3551.76	4328.7	5876.77	7609.14	8508.31	7959.36	6740.18	4939.52	2827.43	1551.24	878.29
United Arab Emirates	Male	51.72	99.48	192.7	366.41	677.63	1186.9	1934.3	2588.27	3026.19	3386.22	3568.2	3450.03	2950.01	2407.34	1837.96	1197.56	700.84	340.65
United Arab Emirates	Female	52.79	101.02	176.1	270	384.2	561.85	897.51	1284.03	1669.03	2157.72	2613.94	2740.2	2350.26	1783.26	1223.22	678.37	317.77	122.53
United Kingdom	Male	16.81	22.4	55.16	213.54	735.67	1613.43	2730.53	3726.55	4577.2	5538.8	6348.59	6744.59	6625.6	6437.67	5925.41	4743.71	3557.35	2595.99
United Kingdom	Female	8.7	15.24	45.61	204.58	580.84	1008.83	1382.38	1655.98	1885.19	2154.39	2380.84	2454.06	2290.28	2147.11	1960.97	1472.13	929.6	532.48
United Republic of Tanzania	Male	18.97	25.23	44.18	97.05	197.02	324.48	472.66	589.92	681.95	808.96	941.11	1060.11	1125.03	1114.23	916.39	581.07	327.05	168.45
United Republic of Tanzania	Female	29.11	52.81	99.99	178.99	284.78	426.51	654.89	952.71	1377.32	1730.2	1589.18	1218.43	1019.21	1162.88	1650.61	1382.74	508.29	154.87
United States of America	Male	21.23	35.46	76.56	211.95	448.74	688.5	968.59	1242.59	1484.53	1803.83	2166.1	2645.18	3183.98	3820.99	4143.39	3805.94	3249.13	2686.03
United States of America	Female	21.78	35.51	98.91	366.49	709.49	901.58	1085.66	1231.06	1299.47	1394.86	1487.41	1588.43	1670.71	1812.03	1851.56	1644.35	1397.28	1145.76
United States Virgin	Male	46.78	73.07	129.08	255.57	514	931.23	1512.92	2093.47	2801.2	4039.51	5974.54	7990.2	8185.22	7153.81	6252.76	4387.02	2219.23	1199.94
United States Virgin	Female	34.93	58.92	105.54	193.54	347.96	593.18	976.13	1358.25	1748.75	2282.39	2819.43	3121.39	2954.84	2458.55	1749.48	1075.71	662.19	381.94
Uruguay	Male	25.99	48.68	111.9	311.45	852.49	1812.35	3086.53	4075.23	4687.59	5168.03	5288.31	5022.47	4352.98	3672.56	2931.12	2154.98	1473.17	820.63
Uruguay	Female	14.8	34.85	102.19	294.09	673.18	1219.52	1900.08	2380.34	2652.82	2879.16	2836.36	2480.95	1914.92	1448.86	1089.82	714.2	426	227.69
Uzbekistan	Male	97.09	161.72	288.6	626.33	1456.04	2361.71	2784.8	2902.22	3265.92	3950.27	4582.25	5154.7	5585.14	5750.83	5133.46	3871.63	2534.58	1372.33
Uzbekistan	Female	60.71	113.3	251.47	532.08	853.83	1089.16	1341.13	1591.99	1868.31	2266.36	2641.05	2818.73	2632.31	2247.79	1740.04	1184.38	754	425.38
Vanuatu	Male	34.04	49.13	86.12	184.92	421.41	862	1515.05	2106.27	2624.2	3234.73	3709.53	3848.03	3467.32	2893.63	2235.36	1535.63	950.76	489.48
Vanuatu	Female	21.29	32.34	56.93	110.36	211.03	384.59	679.19	991.19	1232.95	1480.38	1710.8	1834.79	1693.14	1381.9	982.45	556.5	279.82	119.17

Venezuela (Bolivarian Republic of)	Male	49.19	69.33	104.14	189.88	387.51	719.54	1166.47	1562.24	1912.25	2218.4	2449.47	2575.96	2492.93	2394.95	2287.34	2215.26	1822.24	1224.89
Venezuela (Bolivarian Republic of)	Female	42.83	71.08	90.33	107.17	172.01	331.28	617.08	958.77	1364.51	1804.13	2027.27	1933.96	1578.87	1178.02	849.83	598.29	424.68	287.94
Viet Nam	Male	48.47	76	136.61	299.42	766.84	1652.33	3019.4	4482.02	5931.73	7650.5	8856.45	9101.25	8849.03	8201.65	6576.04	4761.85	3197.04	1813.76
Viet Nam	Female	32.58	60.4	111.01	207.02	387.13	670.07	1088.77	1486.55	1842.63	2425.3	2829.48	2801.01	2575.62	2232.62	1704.28	1114.95	709.56	474.06
Viet Nam	Male	52.12	99.91	192.1	364.59	677.12	1188.24	1942.69	2603.07	3025.18	3386.07	3569.28	3411.97	2897.51	2363.12	1801.3	1150.02	638.27	290.45
Yemen	Female	52.85	101.13	175.81	268.77	383.7	559.98	896.47	1289.51	1676.44	2153.17	2604.3	2729.72	2361.85	1821.18	1258.85	703.46	325.38	121.54
Zambia	Male	19.04	25.54	44.72	98.05	199.64	328.23	474.16	592.19	680.43	800.75	935.32	1061.43	1124.33	1112.63	910.85	569.59	320.41	171.11
Zambia	Female	28.88	52.64	100.38	178.72	282.09	418.39	646.1	945.23	1450.11	1881.71	1667.26	1220.2	1017.21	1167.13	1738.71	1483.25	523.31	152.17
Zimbabwe	Male	38.85	68.8	159.13	450.13	867.9	1091.89	1213.73	1354.05	1467.1	1600.53	1752.07	1969.23	2323.77	2553.59	1917.3	981.8	552.41	402.13
Zimbabwe	Female	27.82	55.85	110.38	175.08	243.64	362.56	590.67	809.38	938.32	1033.26	1040.5	948.06	810.24	696.14	551.89	350.08	180.05	75.02

Supplementary Table S5. The death cases and age-standardized death rate of Urolithiasis in 1990 and 2019, and its temporal trends from 1990 to 2019.

Supplementary Table S5. The death cases and age-standardized death rate of Urolithiasis in 1990 and 2019, and its temporal trends from 1990 to 2019.

Nation	Sex	Deaths cases No. (95% UI)		change absolute number (%)	ASDR per 100,000 No. (95% UI)		1990-2019 EAPC No. (95% CI)
		1990	2019		1990	2019	
Afghanistan	Both	0.02 [0.01-0.07]	0.08 [0.03-0.19]	300	0.03 [0.01-0.11]	0.06 [0.02-0.17]	2.43 [2.11 to 2.74]
Albania	Both	0.02 [0.01-0.04]	0 [0-0.02]	−100.00	0.12 [0.04-0.17]	0.01 [0-0.04]	-7.3 [-8.79 to -5.79]
Algeria	Both	0.01 [0-0.04]	0.04 [0.02-0.08]	300	0.01 [0-0.04]	0.02 [0.01-0.03]	1.46 [1.05 to 1.88]
American Samoa	Both	0 [0-0]	0 [0-0]	NaN	0.23 [0.08-0.38]	0.1 [0.05-0.16]	-3.87 [-5.29 to -2.43]
Andorra	Both	0 [0-0]	0 [0-0]	NaN	0.18 [0.07-0.3]	0.13 [0.06-0.19]	-1.02 [-1.37 to -0.67]
Angola	Both	0.07 [0.03-0.14]	0.11 [0.06-0.22]	57.14	0.18 [0.08-0.34]	0.11 [0.06-0.2]	-2.06 [-2.28 to -1.85]
Antigua and Barbuda	Both	0 [0-0]	0 [0-0]	NaN	0.06 [0.04-0.08]	0.12 [0.09-0.16]	2.46 [2.16 to 2.75]
Argentina	Both	0.03 [0.02-0.05]	0.14 [0.1-0.2]	366.67	0.01 [0.01-0.02]	0.03 [0.02-0.04]	3.57 [2.92 to 4.24]
Armenia	Both	0.17 [0.13-0.24]	0.7 [0.36-1.57]	311.76	0.76 [0.55-1.06]	1.82 [0.94-4.03]	4.11 [3.51 to 4.72]
Australia	Both	0.36 [0.27-0.42]	0.4 [0.32-0.55]	11.11	0.19 [0.14-0.22]	0.09 [0.07-0.12]	-2.56 [-3.16 to -1.95]
Austria	Both	0.49 [0.38-0.6]	0.18 [0.11-0.23]	−63.27	0.39 [0.3-0.48]	0.09 [0.06-0.11]	-3.47 [-4.84 to -2.07]
Azerbaijan	Both	0.02 [0.01-0.03]	0.06 [0.01-0.12]	200	0.04 [0.01-0.08]	0.13 [0.02-0.29]	4.41 [3.33 to 5.5]
Bahamas	Both	0 [0-0]	0 [0-0.01]	NaN	0.07 [0.05-0.1]	0.11 [0.08-0.15]	1.51 [1.2 to 1.83]
Bahrain	Both	0 [0-0]	0 [0-0]	NaN	0.02 [0.01-0.06]	0.05 [0.02-0.16]	2.63 [1.28 to 4]
Bangladesh	Both	0.62 [0.29-0.99]	0.74 [0.34-1.4]	19.35	0.12 [0.06-0.21]	0.06 [0.03-0.12]	-2.07 [-2.36 to -1.77]
Barbados	Both	0 [0-0.01]	0.01 [0.01-0.01]	Inf	0.12 [0.09-0.18]	0.18 [0.11-0.24]	1.47 [1.21 to 1.74]
Belarus	Both	0.65 [0.51-0.82]	0.68 [0.42-0.98]	4.62	0.51 [0.4-0.64]	0.42 [0.26-0.61]	-1.13 [-1.34 to -0.93]
Belgium	Both	0.1 [0.07-0.13]	0.34 [0.22-0.67]	240	0.07 [0.05-0.09]	0.12 [0.08-0.23]	2.34 [1.84 to 2.85]
Belize	Both	0 [0-0]	0.01 [0-0.01]	Inf	0.08 [0.06-0.12]	0.19 [0.13-0.23]	2.7 [1.84 to 3.56]
Benin	Both	0.02 [0.01-0.03]	0.03 [0.02-0.04]	50	0.1 [0.04-0.19]	0.06 [0.04-0.09]	-1.96 [-2.09 to -1.83]
Bermuda	Both	0 [0-0]	0 [0-0]	NaN	0.07 [0.05-0.1]	0.08 [0.06-0.11]	0.71 [0.28 to 1.13]
Bhutan	Both	0 [0-0.01]	0.01 [0-0.01]	Inf	0.22 [0.09-0.53]	0.14 [0.06-0.29]	-1.47 [-1.57 to -1.38]
Bolivia (Plurinational State of)	Both	0.06 [0.02-0.09]	0.12 [0.05-0.18]	100	0.18 [0.07-0.3]	0.14 [0.06-0.21]	-0.57 [-0.72 to -0.43]

Bosnia and Herzegovina	Both	0.04 [0.02-0.05]	0.02 [0.01-0.03]	−50.00	0.1 [0.06-0.15]	0.03 [0.02-0.05]	-5.9 [-7.13 to -4.66]
Botswana	Both	0 [0-0.01]	0.01 [0-0.01]	Inf	0.05 [0.03-0.08]	0.06 [0.03-0.1]	-0.15 [-0.55 to 0.25]
Brazil	Both	0.88 [0.66-1.06]	5.47 [4.29-9.27]	521.59	0.1 [0.07-0.12]	0.24 [0.18-0.4]	4 [3.75 to 4.25]
Brunei Darussalam	Both	0 [0-0]	0 [0-0.01]	NaN	0.39 [0.24-0.67]	0.3 [0.19-0.45]	-0.28 [-0.58 to 0.01]
Bulgaria	Both	0.99 [0.77-1.67]	0.07 [0.03-0.11]	−92.93	0.85 [0.67-1.4]	0.05 [0.02-0.08]	-10.59 [-12.16 to -8.99]
Burkina Faso	Both	0.04 [0.02-0.07]	0.08 [0.03-0.14]	100	0.12 [0.05-0.23]	0.11 [0.05-0.2]	-0.56 [-0.7 to -0.42]
Burundi	Both	0.04 [0.01-0.08]	0.05 [0.01-0.1]	25	0.19 [0.06-0.38]	0.16 [0.05-0.32]	-0.81 [-0.96 to -0.67]
Cabo Verde	Both	0 [0-0]	0 [0-0]	NaN	0.02 [0.01-0.03]	0.02 [0.01-0.03]	-0.71 [-1.2 to -0.22]
Cambodia	Both	0.26 [0.03-0.46]	0.47 [0.06-0.79]	80.77	0.57 [0.07-1.11]	0.43 [0.06-0.74]	-1.18 [-1.31 to -1.05]
Cameroon	Both	0.04 [0.02-0.08]	0.07 [0.03-0.12]	75	0.12 [0.04-0.22]	0.07 [0.03-0.12]	-2.45 [-2.66 to -2.23]
Canada	Both	0.26 [0.2-0.31]	0.83 [0.63-1.25]	219.23	0.08 [0.06-0.1]	0.11 [0.09-0.16]	2.03 [1.52 to 2.53]
Central African Republic	Both	0.02 [0.01-0.05]	0.05 [0.02-0.09]	150	0.2 [0.08-0.35]	0.21 [0.08-0.39]	0.17 [0 to 0.35]
Chad	Both	0.03 [0.01-0.05]	0.03 [0.02-0.05]	0	0.1 [0.04-0.2]	0.06 [0.04-0.1]	-1.94 [-2.1 to -1.77]
Chile	Both	0.07 [0.06-0.1]	0.15 [0.12-0.19]	114.29	0.07 [0.06-0.1]	0.06 [0.05-0.08]	-1.3 [-1.76 to -0.83]
China	Both	40.07 [18.12-50.57]	25.58 [17.34-35.48]	−36.16	0.56 [0.26-0.71]	0.15 [0.1-0.2]	-5.1 [-5.42 to -4.78]
Colombia	Both	0.09 [0.07-0.11]	0.33 [0.24-0.47]	266.67	0.05 [0.03-0.06]	0.06 [0.04-0.09]	2.2 [1.28 to 3.12]
Comoros	Both	0 [0-0.01]	0.01 [0-0.02]	Inf	0.19 [0.06-0.36]	0.19 [0.07-0.41]	-0.15 [-0.27 to -0.04]
Congo	Both	0.02 [0.01-0.03]	0.03 [0.01-0.05]	50	0.17 [0.09-0.3]	0.12 [0.06-0.2]	-1.49 [-1.71 to -1.27]
Costa Rica	Both	0 [0-0]	0.01 [0.01-0.02]	Inf	0 [0-0.01]	0.02 [0.01-0.04]	7.18 [6.26 to 8.1]
Cote d'Ivoire	Both	0.03 [0.01-0.05]	0.05 [0.03-0.09]	66.67	0.1 [0.04-0.18]	0.06 [0.03-0.1]	-1.72 [-1.9 to -1.55]
Croatia	Both	0.05 [0.04-0.07]	0.09 [0.05-0.18]	80	0.08 [0.07-0.12]	0.09 [0.06-0.18]	2.4 [0.93 to 3.88]
Cuba	Both	0.16 [0.13-0.21]	0.41 [0.3-0.6]	156.25	0.15 [0.12-0.21]	0.22 [0.16-0.32]	1.6 [1.26 to 1.94]
Cyprus	Both	0.01 [0-0.02]	0.01 [0-0.03]	0	0.13 [0.06-0.28]	0.06 [0.03-0.2]	-2.87 [-3.17 to -2.57]
Czechia Democratic	Both	1.18 [0.91-2.26]	0.21 [0.15-0.29]	−82.20	0.86 [0.66-1.61]	0.09 [0.07-0.13]	-7.19 [-8.27 to -6.1]
People's Republic of Korea	Both	0.47 [0.17-0.71]	0.72 [0.3-1.14]	53.19	0.3 [0.12-0.47]	0.23 [0.1-0.37]	-1.02 [-1.28 to -0.77]
Democratic Republic of the Congo	Both	0.18 [0.08-0.31]	0.36 [0.16-0.66]	100	0.12 [0.05-0.21]	0.11 [0.04-0.2]	-0.37 [-0.53 to -0.21]
Denmark	Both	0.13 [0.11-0.17]	0.22 [0.17-0.34]	69.23	0.16 [0.13-0.2]	0.17 [0.13-0.26]	0.7 [0.24 to 1.17]
Djibouti	Both	0 [0-0]	0.01 [0-0.02]	Inf	0.19 [0.09-0.33]	0.21 [0.08-0.46]	0.43 [0.24 to 0.62]
Dominica	Both	0 [0-0]	0 [0-0]	NaN	0.03 [0.02-0.06]	0.06 [0.04-0.09]	2.83 [2.59 to 3.07]
Dominican Republic	Both	0.01 [0.01-0.02]	0.02 [0.01-0.04]	100	0.03 [0.01-0.05]	0.03 [0.02-0.04]	-0.1 [-0.9 to 0.7]

Ecuador	Both	0.03 [0.02-0.05]	0.12 [0.07-0.19]	300	0.07 [0.04-0.1]	0.08 [0.05-0.14]	1.72 [1.2 to 2.24]
Egypt	Both	0.02 [0.01-0.04]	0.06 [0.02-0.11]	200	0.01 [0-0.02]	0.01 [0-0.02]	1.49 [1.22 to 1.77]
El Salvador	Both	0.01 [0-0.01]	0.02 [0.01-0.03]	100	0.03 [0.01-0.05]	0.03 [0.01-0.04]	-0.87 [-1.4 to -0.34]
Equatorial Guinea	Both	0 [0-0.01]	0 [0-0.01]	NaN	0.17 [0.06-0.34]	0.1 [0.05-0.18]	-2.03 [-2.51 to -1.54]
Eritrea	Both	0.02 [0.01-0.03]	0.04 [0.02-0.09]	100	0.2 [0.07-0.4]	0.23 [0.1-0.44]	0.51 [0.36 to 0.66]
Estonia	Both	0.15 [0.1-0.28]	0.06 [0.04-0.08]	−60.00	0.73 [0.51-1.38]	0.19 [0.13-0.26]	-6.47 [-7.28 to -5.66]
Eswatini	Both	0 [0-0]	0 [0-0.01]	NaN	0.07 [0.04-0.11]	0.07 [0.04-0.11]	0.3 [-0.51 to 1.12]
Ethiopia	Both	0.62 [0.35-1.11]	0.6 [0.3-1]	−3.23	0.36 [0.19-0.59]	0.2 [0.11-0.32]	-2.34 [-2.5 to -2.18]
Fiji	Both	0 [0-0]	0 [0-0.01]	NaN	0.03 [0.01-0.06]	0.05 [0.03-0.08]	3.88 [3.08 to 4.69]
Finland	Both	0.05 [0.03-0.1]	0.06 [0.04-0.08]	20	0.06 [0.04-0.13]	0.04 [0.03-0.06]	-1.2 [-1.66 to -0.74]
France	Both	0.82 [0.65-0.98]	0.98 [0.73-1.3]	19.51	0.1 [0.08-0.11]	0.06 [0.04-0.08]	-1.64 [-1.8 to -1.48]
Gabon	Both	0.01 [0-0.02]	0.01 [0.01-0.03]	0	0.16 [0.05-0.35]	0.14 [0.06-0.28]	-0.64 [-0.84 to -0.43]
Gambia	Both	0 [0-0]	0.01 [0-0.01]	Inf	0.09 [0.04-0.2]	0.07 [0.04-0.12]	-1.21 [-1.37 to -1.05]
Germany	Both	1.16 [0.82-1.42]	1.4 [1.09-2.02]	20.69	0.09 [0.06-0.11]	0.06 [0.05-0.09]	-0.39 [-1.17 to 0.4]
Ghana	Both	0.16 [0.02-0.37]	0.38 [0.04-0.69]	137.5	0.32 [0.04-0.76]	0.3 [0.03-0.53]	0.31 [0.12 to 0.51]
Greece	Both	0.02 [0.01-0.03]	0.03 [0.02-0.05]	50	0.01 [0.01-0.02]	0.01 [0.01-0.02]	0.46 [-0.6 to 1.53]
Greenland	Both	0 [0-0]	0 [0-0]	NaN	0.04 [0.01-0.09]	0.02 [0.01-0.02]	-3.9 [-4.69 to -3.11]
Grenada	Both	0 [0-0]	0 [0-0]	NaN	0.16 [0.13-0.23]	0.33 [0.23-0.44]	2.92 [2.47 to 3.37]
Guam	Both	0 [0-0]	0 [0-0]	NaN	0.2 [0.03-0.31]	0.02 [0.01-0.04]	-8.97 [-10.54 to -7.37]
Guatemala	Both	0.06 [0.04-0.08]	0.13 [0.09-0.17]	116.67	0.16 [0.1-0.2]	0.11 [0.08-0.16]	-1.44 [-1.85 to -1.03]
Guinea	Both	0.03 [0.01-0.06]	0.04 [0.02-0.06]	33.33	0.1 [0.04-0.2]	0.07 [0.04-0.11]	-1.25 [-1.36 to -1.13]
Guinea-Bissau	Both	0.01 [0-0.01]	0.01 [0-0.01]	0	0.15 [0.07-0.26]	0.09 [0.05-0.14]	-2.05 [-2.17 to -1.93]
Guyana	Both	0.01 [0.01-0.01]	0.02 [0.01-0.03]	100	0.18 [0.14-0.27]	0.28 [0.18-0.39]	1.58 [1.15 to 2.01]
Haiti	Both	0.11 [0.04-0.2]	0.22 [0.09-0.45]	100	0.29 [0.12-0.6]	0.31 [0.12-0.65]	0.57 [0.42 to 0.71]
Honduras	Both	0.05 [0.01-0.08]	0.11 [0.04-0.2]	120	0.23 [0.06-0.39]	0.2 [0.08-0.36]	-0.25 [-0.49 to -0.02]
Hungary	Both	1.7 [1.43-2.28]	0.6 [0.38-0.8]	−64.71	1.21 [1-1.62]	0.29 [0.18-0.39]	-4.42 [-5.25 to -3.58]
Iceland	Both	0 [0-0.01]	0.01 [0-0.01]	Inf	0.15 [0.12-0.18]	0.1 [0.07-0.12]	-1.15 [-1.4 to -0.9]
India	Both	14.02 [6.16-22.44]	17.5 [10.23-26.35]	24.82	0.37 [0.17-0.64]	0.17 [0.1-0.26]	-3.02 [-3.25 to -2.8]
Indonesia	Both	4.82 [0.47-8]	8.78 [0.92-14.19]	82.16	0.5 [0.06-0.84]	0.44 [0.05-0.7]	-0.26 [-0.34 to -0.19]
Iran (Islamic Republic of)	Both	0.05 [0.02-0.1]	0.23 [0.05-0.38]	360	0.02 [0.01-0.05]	0.04 [0.01-0.06]	1.87 [0.74 to 3]
Iraq	Both	0.08 [0.05-0.16]	0.07 [0.04-0.1]	−12.50	0.09 [0.05-0.16]	0.03 [0.02-0.06]	-4.8 [-5.47 to -4.13]

Ireland	Both	0.04 [0.03-0.05]	0.05 [0.04-0.07]	25	0.09 [0.07-0.12]	0.07 [0.05-0.09]	-1.2 [-1.8 to -0.6]
Israel	Both	0.03 [0.02-0.05]	0.17 [0.11-0.27]	466.67	0.06 [0.04-0.12]	0.13 [0.09-0.2]	1.28 [0.15 to 2.42]
Italy	Both	1.41 [1.02-1.61]	0.88 [0.62-1.18]	−37.59	0.16 [0.12-0.19]	0.05 [0.04-0.07]	-3.53 [-4.28 to -2.77]
Jamaica	Both	0 [0-0.01]	0.03 [0.02-0.05]	Inf	0.02 [0.02-0.05]	0.11 [0.08-0.17]	6.74 [6 to 7.49]
Japan	Both	1.01 [0.69-1.26]	6.39 [4.47-8.84]	532.67	0.07 [0.05-0.08]	0.12 [0.09-0.17]	3.23 [2.9 to 3.56]
Jordan	Both	0 [0-0]	0.01 [0-0.01]	Inf	0.02 [0.01-0.03]	0.01 [0.01-0.02]	-2.06 [-2.4 to -1.73]
Kazakhstan	Both	0.83 [0.51-1.4]	1.37 [1.04-1.76]	65.06	0.71 [0.44-1.19]	0.88 [0.68-1.16]	0.28 [-0.11 to 0.66]
Kenya	Both	0.09 [0.05-0.16]	0.31 [0.16-0.6]	244.44	0.16 [0.09-0.26]	0.21 [0.11-0.38]	1.18 [1.07 to 1.29]
Kuwait	Both	0 [0-0]	0 [0-0.01]	NaN	0.01 [0.01-0.01]	0.01 [0.01-0.03]	4.53 [3.71 to 5.34]
Kyrgyzstan	Both	0.08 [0.06-0.12]	0.06 [0.05-0.09]	−25.00	0.24 [0.19-0.34]	0.13 [0.09-0.17]	-2.79 [-3.49 to -2.08]
Lao People's Democratic Republic	Both	0.14 [0.01-0.29]	0.15 [0.02-0.28]	7.14	0.67 [0.07-1.46]	0.38 [0.05-0.69]	-2.33 [-2.58 to -2.09]
Latvia	Both	0.31 [0.23-0.49]	0.24 [0.18-0.33]	−22.58	0.86 [0.64-1.37]	0.53 [0.41-0.71]	-2.65 [-3.11 to -2.2]
Lebanon	Both	0 [0-0.01]	0.01 [0-0.01]	Inf	0.01 [0-0.03]	0.01 [0-0.02]	2.16 [1.69 to 2.64]
Lesotho	Both	0.01 [0-0.01]	0.01 [0.01-0.02]	0	0.06 [0.03-0.11]	0.09 [0.04-0.15]	2.57 [2.11 to 3.04]
Liberia	Both	0.01 [0-0.02]	0.01 [0.01-0.02]	0	0.11 [0.04-0.19]	0.06 [0.04-0.11]	-2.15 [-2.43 to -1.87]
Libya	Both	0 [0-0.01]	0.01 [0-0.02]	Inf	0.01 [0-0.03]	0.02 [0.01-0.03]	3.6 [3.09 to 4.11]
Lithuania	Both	0.36 [0.31-0.49]	0.22 [0.16-0.3]	−38.89	0.82 [0.69-1.1]	0.34 [0.26-0.47]	-4.01 [-4.44 to -3.58]
Luxembourg	Both	0 [0-0.01]	0 [0-0.01]	NaN	0.08 [0.06-0.1]	0.04 [0.03-0.06]	-1.88 [-2.11 to -1.65]
Madagascar	Both	0.07 [0.03-0.13]	0.12 [0.05-0.26]	71.43	0.16 [0.07-0.28]	0.15 [0.07-0.3]	-0.15 [-0.24 to -0.07]
Malawi	Both	0.07 [0.03-0.13]	0.11 [0.05-0.21]	57.14	0.22 [0.1-0.41]	0.22 [0.09-0.41]	-0.02 [-0.08 to 0.04]
Malaysia	Both	0.08 [0.03-0.12]	0.24 [0.07-0.65]	200	0.1 [0.04-0.16]	0.1 [0.03-0.29]	0.29 [0.06 to 0.51]
Maldives	Both	0 [0-0]	0 [0-0]	NaN	0.08 [0.02-0.17]	0.03 [0.01-0.05]	-4.42 [-4.69 to -4.15]
Mali	Both	0.05 [0.02-0.08]	0.06 [0.03-0.09]	20	0.13 [0.05-0.23]	0.07 [0.04-0.12]	-2.33 [-2.54 to -2.13]
Malta	Both	0.01 [0.01-0.01]	0.01 [0.01-0.01]	0	0.19 [0.14-0.23]	0.1 [0.08-0.14]	-1.85 [-2.2 to -1.5]
Marshall Islands	Both	0 [0-0]	0 [0-0]	NaN	0.24 [0.09-0.48]	0.18 [0.07-0.34]	-1.02 [-1.1 to -0.93]
Mauritania	Both	0.01 [0.01-0.02]	0.01 [0.01-0.02]	0	0.16 [0.06-0.29]	0.07 [0.03-0.12]	-3.42 [-3.59 to -3.25]
Mauritius	Both	0 [0-0]	0 [0-0.01]	NaN	0.03 [0.02-0.04]	0.02 [0.01-0.03]	-1.51 [-2.65 to -0.36]
Mexico	Both	1.48 [1.2-1.74]	3.67 [2.88-5.45]	147.97	0.35 [0.28-0.41]	0.31 [0.25-0.47]	-0.27 [-0.58 to 0.04]
Micronesia (Federated States of)	Both	0 [0-0]	0 [0-0]	NaN	0.28 [0.1-0.51]	0.19 [0.08-0.34]	-1.53 [-1.64 to -1.41]

Mongolia	Both	0.01 [0.01-0.03]	0.01 [0-0.02]	0	0.14 [0.05-0.3]	0.04 [0.02-0.09]	-5.97 [-6.74 to -5.2]
Montenegro	Both	0 [0-0]	0 [0-0]	NaN	0 [0-0.01]	0 [0-0.01]	0.4 [0.26 to 0.54]
Morocco	Both	0.01 [0-0.04]	0.06 [0.02-0.1]	500	0.01 [0-0.04]	0.02 [0.01-0.04]	3.07 [2.66 to 3.47]
Mozambique	Both	0.11 [0.04-0.2]	0.21 [0.09-0.42]	90.91	0.25 [0.1-0.47]	0.27 [0.11-0.52]	0.39 [0.27 to 0.5]
Myanmar	Both	1.38 [0.16-2.68]	1.66 [0.22-2.99]	20.29	0.6 [0.08-1.23]	0.39 [0.05-0.7]	-1.76 [-2.06 to -1.46]
Namibia	Both	0[0-0.01]	0.01[0-0.01]	Inf	0.06[0.03-0.1]	0.05[0.02-0.08]	-1.34 [-1.84 to -0.84]
Nepal	Both	0.18[0.08-0.34]	0.31[0.15-0.6]	72.22	0.21[0.09-0.44]	0.16[0.08-0.31]	-0.74 [-1.16 to -0.33]
Netherlands	Both	0.65[0.53-0.91]	0.54[0.4-0.83]	−16.92	0.32[0.26-0.44]	0.14[0.11-0.22]	-3.08 [-3.46 to -2.69]
New Zealand	Both	0.11[0.09-0.15]	0.15[0.12-0.2]	36.36	0.27[0.23-0.38]	0.18[0.15-0.25]	-0.9[-1.46 to -0.33]
Nicaragua	Both	0.01[0-0.01]	0.03[0.01-0.04]	200	0.04[0.02-0.07]	0.07[0.02-0.1]	1.22[0.55 to 1.9]
Niger	Both	0.03[0.01-0.05]	0.04[0.02-0.07]	33.33	0.12[0.05-0.24]	0.06[0.03-0.1]	-2.61[-2.81to -2.4]
Nigeria	Both	0.29[0.13-0.49]	0.35[0.19-0.51]	20.69	0.08[0.03-0.14]	0.05[0.03-0.07]	-2.35[-2.55 to -2.14]
North Macedonia	Both	0[0-0]	0[0-0]	NaN	0.01[0.01-0.02]	0[0-0.01]	-2.85[-3.76 to -1.93]
Northern Mariana Islands	Both	0[0-0]	0[0-0]	NaN	0.1 [0.06-0.17]	0.04[0.03-0.07]	-2.78 [-3.64 to -1.92]
Norway	Both	0.21[0.17-0.3]	0.14[0.1-0.24]	−33.33	0.27[0.22-0.38]	0.12[0.09-0.21]	-2.36[-2.85 to -1.86]
Oman	Both	0[0-0]	0[0-0.01]	NaN	0.02[0.01-0.05]	0.03[0.01-0.07]	4.16[3.41 to 4.92]
Pakistan	Both	1.36[0.65-2.46]	2.27[1.21-3.9]	66.91	0.24[0.11-0.48]	0.23[0.12-0.39]	-0.48[-0.81to -0.15]
Palestine	Both	0[0-0.01]	0.01[0-0.01]	Inf	0.04[0.02-0.09]	0.03[0.02-0.07]	-1.53[-2.26 to -0.8]
Panama	Both	0[0-0]	0.01[0.01-0.01]	Inf	0.01[0.01-0.02]	0.02[0.01-0.03]	2.16[0.98 to 3.36]
Papua New Guinea	Both	0.04[0.01-0.06]	0.07[0.02-0.12]	75	0.19[0.05-0.32]	0.13[0.04-0.23]	-1.25[-1.33 to -1.18]
Paraguay	Both	0.01[0-0.01]	0.04[0.01-0.08]	300	0.04[0.01-0.06]	0.08[0.02-0.14]	3[2.25 to 3.76]
Peru	Both	0.06[0.03-0.08]	0.13[0.05-0.19]	116.67	0.05[0.02-0.07]	0.04[0.02-0.06]	-0.22[-0.82 to 0.38]
Philippines	Both	2.44[1.67-3.26]	4.75[3.52-7.47]	94.67	0.86[0.62-1.29]	0.65[0.48-1.12]	-1.04[-1.18 to -0.9]
Poland	Both	1.69[1.47-2.32]	0.26[0.19-0.37]	−84.62	0.39[0.34-0.54]	0.04[0.03-0.05]	-8.98[-10.31 to -7.63]
Portugal	Both	0.1[0.05-0.13]	0.23[0.15-0.48]	130	0.08[0.03-0.1]	0.08[0.05-0.17]	1.12[-0.02 to 2.28]
Puerto Rico	Both	0[0-0]	0.02[0.02-0.03]	Inf	0.01[0.01-0.01]	0.03[0.02-0.04]	3.67[2.74 to 4.61]
Qatar	Both	0[0-0]	0[0-0]	NaN	0.02[0.01-0.04]	0.02[0.01-0.03]	0.42[-0.02 to 0.86]
Republic of Korea	Both	0.18[0.12-0.3]	0.64[0.33-1.07]	255.56	0.09[0.06-0.15]	0.08[0.04-0.13]	-1.11[-1.74 to -0.48]
Republic of Moldova	Both	0.13 [0.11-0.17]	0.16 [0.1-0.21]	23.08	0.32 [0.25-0.41]	0.28 [0.18-0.36	-0.24 [-0.97 to 0.49
Romania	Both	0.02[0.02-0.03]	0.03[0.02-0.04]	50	0.01[0.01-0.01]	0.01[0-0.01]	-0.3 [-0.97 to 0.37]
Russian Federation	Both	12.81 [10.69-17.15]	14.4 [11.78-18.15]	12.41	0.72 [0.59-0.97]	0.62 [0.5-0.78]	-0.59 [-1.17 to 0]

Rwanda	Both	0.05 [0-0.11]	0.07 [0-0.17]	40	0.21 [0.01-0.44]	0.18 [0.01-0.47]	-1.02 [-1.23 to -0.8]
Saint Lucia	Both	0 [0-0]	0 [0-0]	NaN	0.12 [0.09-0.15]	0.18 [0.13-0.23]	0.96 [0.67 to 1.25]
Saint Vincent and the Grenadines	Both	0 [0-0]	0 [0-0]	NaN	0.13 [0.09-0.2]	0.17 [0.1-0.23]	1.25 [0.81 to 1.69]
Samoa	Both	0 [0-0]	0 [0-0]	NaN	0.21 [0.08-0.34]	0.12 [0.05-0.19]	-2.19 [-2.34 to -2.04]
Sao Tome and Principe	Both	0 [0-0]	0 [0-0]	NaN	0.11 [0.04-0.18]	0.08 [0.03-0.18]	-1.41 [-1.67 to -1.14]
Saudi Arabia	Both	0 [0-0.01]	0.01 [0.01-0.02]	Inf	0.01 [0-0.02]	0.01 [0-0.03]	1.35 [0.97 to 1.72]
Senegal	Both	0.03 [0.01-0.05]	0.04 [0.02-0.07]	33.33	0.12 [0.05-0.21]	0.06 [0.04-0.1]	-2.33 [-2.47 to -2.19]
Serbia	Both	0.07 [0.05-0.1]	0.08 [0.05-0.12]	14.29	0.07 [0.05-0.1]	0.05 [0.03-0.07]	-1.05 [-1.23 to -0.87]
Seychelles	Both	0 [0-0]	0 [0-0.01]	NaN	0.14 [0.03-0.24]	0.27 [0.03-0.5]	1.93 [1.48 to 2.38]
Sierra Leone	Both	0.01 [0.01-0.03]	0.02 [0.01-0.03]	100	0.09 [0.04-0.16]	0.06 [0.03-0.09]	-1.31 [-1.44 to -1.18]
Singapore	Both	0.03 [0.02-0.04]	0.02 [0.01-0.02]	−33.33	0.14 [0.11-0.21]	0.02 [0.02-0.03]	-5.19 [-6.3 to -4.07]
Slovakia	Both	0.13 [0.06-0.18]	0.06 [0.04-0.11]	−53.85	0.23 [0.11-0.3]	0.07 [0.05-0.12]	-4.39 [-4.76 to -4.02]
Slovenia	Both	0.04 [0.02-0.08]	0.02 [0.01-0.04]	−50.00	0.15 [0.09-0.31]	0.03 [0.02-0.07]	-5.53 [-5.95 to -5.11]
Solomon Islands	Both	0 [0-0.01]	0.01 [0-0.01]	Inf	0.26 [0.1-0.47]	0.2 [0.09-0.34]	-0.86 [-0.98 to -0.74]
Somalia	Both	0.06 [0.02-0.13]	0.16 [0.05-0.34]	166.67	0.27 [0.1-0.53]	0.32 [0.11-0.66]	0.79 [0.68 to 0.91]
South Africa	Both	0.13 [0.1-0.19]	0.21 [0.16-0.3]	61.54	0.06 [0.04-0.08]	0.05 [0.04-0.07]	-0.49 [-1.23 to 0.25]
South Sudan	Both	0.03 [0.01-0.06]	0.04 [0.02-0.09]	33.33	0.16 [0.06-0.3]	0.15 [0.06-0.28]	-0.26 [-0.34 to -0.18]
Spain	Both	0.54 [0.35-0.63]	0.74 [0.54-1.2]	37.04	0.1 [0.07-0.12]	0.06 [0.05-0.1]	-1.39 [-1.72 to -1.06]
Sri Lanka	Both	0.01 [0.01-0.02]	0.05 [0.02-0.08]	400	0.02 [0.01-0.03]	0.02 [0.01-0.04]	0.59 [-0.16 to 1.34]
Sudan	Both	0.01 [0-0.04]	0.04 [0.01-0.08]	300	0.01 [0-0.04]	0.02 [0.01-0.05]	2.55 [2.12 to 2.99]
Suriname	Both	0 [0-0.01]	0.02 [0.01-0.03]	Inf	0.16 [0.09-0.3]	0.3 [0.16-0.47]	2.19 [1.66 to 2.72]
Sweden	Both	0.31 [0.24-0.43]	0.31 [0.23-0.39]	0	0.19 [0.15-0.26]	0.12 [0.09-0.15]	-0.57 [-1.33 to 0.19]
Switzerland	Both	0.1 [0.07-0.14]	0.08 [0.06-0.13]	−20.00	0.09 [0.06-0.12]	0.04 [0.03-0.06]	-2.65 [-2.96 to -2.34]
Syrian Arab Republic	Both	0.02 [0-0.03]	0.02 [0-0.03]	0	0.03 [0.01-0.06]	0.01 [0-0.02]	-3.76 [-4.31 to -3.21]
Tajikistan	Both	0.05 [0.02-0.08]	0.13 [0.06-0.2]	160	0.17 [0.08-0.27]	0.29 [0.15-0.48]	2.07 [1.83 to 2.31]
Thailand	Both	2.21 [0.64-3.59]	4.56 [1.23-8.58]	106.33	0.74 [0.21-1.23]	0.47 [0.13-0.88]	-2.16 [-2.83 to -1.49]
Timor-Leste	Both	0.01 [0-0.02]	0.03 [0-0.05]	200	0.41 [0.04-0.98]	0.38 [0.05-0.72]	-0.46 [-0.64 to -0.28]
Togo	Both	0.01 [0-0.02]	0.02 [0.01-0.03]	100	0.11 [0.04-0.19]	0.07 [0.04-0.1]	-1.96 [-2.13 to -1.79]
Tonga	Both	0 [0-0]	0 [0-0]	NaN	0.15 [0.06-0.26]	0.12 [0.05-0.21]	-0.99 [-1.21 to -0.78]
Trinidad and Tobago	Both	0.02 [0.02-0.04]	0.1 [0.07-0.15]	400	0.3 [0.23-0.46]	0.53 [0.37-0.83]	3.58 [2.81 to 4.35]

Tunisia	Both	0 [0-0.01]	0.01 [0-0.02]	Inf	0.01 [0-0.03]	0.01 [0-0.02]	2 [1.64 to 2.35]
Turkey	Both	0.21 [0.11-0.42]	0.47 [0.31-0.66]	123.81	0.06 [0.03-0.13]	0.06 [0.04-0.08]	1.24 [0.45 to 2.03]
Turkmenistan	Both	0.01 [0.01-0.01]	0.07 [0.05-0.09]	600	0.05 [0.02-0.07]	0.18 [0.13-0.24]	4.77 [4.3 to 5.25]
Uganda	Both	0.07 [0.01-0.13]	0.16 [0.04-0.34]	128.57	0.14 [0.02-0.28]	0.17 [0.04-0.35]	0.35 [0.15 to 0.56]
Ukraine	Both	3.57 [2.75-4.49]	3.18 [2.03-4.11]	−10.92	0.49 [0.38-0.62]	0.42 [0.28-0.54]	-1.27 [-1.63 to -0.91]
United Arab Emirates	Both	0 [0-0]	0.01 [0-0.02]	Inf	0.02 [0.01-0.06]	0.03 [0.01-0.07]	2.86 [1.95 to 3.77]
United Kingdom	Both	1.5 [0.8-1.74]	2.44 [2.03-3.49]	62.67	0.16 [0.09-0.19]	0.18 [0.15-0.25]	0.69 [0.36 to 1.03]
United Republic of Tanzania	Both	0.13 [0.06-0.25]	0.3 [0.12-0.64]	130.77	0.17 [0.08-0.32]	0.17 [0.06-0.36]	0.04 [-0.06 to 0.14]
United States of America	Both	3.09 [2.46-3.84]	5.41 [4.46-7.27]	75.08	0.09 [0.07-0.12]	0.09 [0.08-0.12]	0.65 [0.29 to 1.02]
United States Virgin Islands	Both	0 [0-0]	0.01 [0.01-0.01]	Inf	0.36 [0.24-0.59]	0.49 [0.33-0.7]	1.67 [1.42 to 1.93]
Uruguay	Both	0.01 [0.01-0.02]	0.04 [0.03-0.05]	300	0.03 [0.02-0.05]	0.07 [0.05-0.09]	2.84 [2.4 to 3.29]
Uzbekistan	Both	0 [0-0.01]	0.01 [0.01-0.02]	Inf	0 [0-0.01]	0.01 [0.01-0.02]	4.27 [3.8 to 4.74]
Vanuatu	Both	0 [0-0]	0 [0-0.01]	NaN	0.19 [0.07-0.37]	0.16 [0.07-0.32]	-0.84 [-1.02 to -0.67]
Venezuela (Bolivarian Republic of)	Both	0.14 [0.12-0.24]	0.61 [0.41-0.84]	335.71	0.14 [0.11-0.23]	0.21 [0.14-0.29]	0.48 [-0.41 to 1.39]
Viet Nam	Both	0.69 [0.06-1.27]	1.13 [0.09-1.84]	63.77	0.18 [0.02-0.33]	0.13 [0.01-0.22]	-1.23 [-1.77 to -0.69]
Yemen	Both	0.01 [0-0.02]	0.03 [0.01-0.08]	200	0.01 [0-0.04]	0.02 [0.01-0.07]	2.78 [2.38 to 3.18]
Zambia	Both	0.05 [0.03-0.11]	0.11 [0.04-0.23]	120	0.23 [0.11-0.42]	0.22 [0.09-0.46]	-0.29 [-0.37 to -0.2]
Zimbabwe	Both	0.02 [0.01-0.04]	0.05 [0.02-0.08]	150	0.05 [0.02-0.07]	0.06 [0.02-0.09]	0.98 [0.38 to 1.58]
Afghanistan	Male	0.01 [0-0.03]	0.02 [0.01-0.06]	100	0.02 [0-0.09]	0.04 [0.01-0.11]	2.26 [1.93 to 2.6]
Albania	Male	0.02 [0.01-0.03]	0 [0-0.02]	−100.00	0.28 [0.09-0.44]	0.02 [0.01-0.08]	-8.74 [-10.47 to -6.99]
Algeria	Male	0.01 [0-0.03]	0.01 [0-0.03]	0	0.01 [0-0.05]	0.01 [0-0.02]	0.94 [0.54 to 1.34]
American Samoa	Male	0 [0-0]	0 [0-0]	NaN	0.29 [0.08-0.56]	0.17 [0.06-0.29]	-2.62 [-4.31 to -0.9]
Andorra	Male	0 [0-0]	0 [0-0]	NaN	0.22 [0.06-0.42]	0.13 [0.05-0.21]	-1.61 [-1.93 to -1.28]
Angola	Male	0.05 [0.02-0.12]	0.08 [0.03-0.15]	60	0.28 [0.09-0.55]	0.18 [0.07-0.34]	-1.96 [-2.16 to -1.76]
Antigua and Barbuda	Male	0 [0-0]	0 [0-0]	NaN	0.05 [0.03-0.07]	0.07 [0.04-0.1]	1.38 [1.15 to 1.61]
Argentina	Male	0.02 [0.01-0.04]	0.07 [0.03-0.11]	250	0.02 [0.01-0.03]	0.03 [0.01-0.05]	2.67 [1.96 to 3.4]
Armenia	Male	0.09 [0.06-0.16]	0.24 [0.11-0.64]	166.67	1.02 [0.71-1.72]	1.56 [0.72-4.12]	1.55 [1.22 to 1.89]
Australia	Male	0.19 [0.13-0.24]	0.19 [0.13-0.28]	0	0.26 [0.18-0.33]	0.1 [0.07-0.14]	-3.31 [-4.01 to -2.6]
Austria	Male	0.13 [0.1-0.2]	0.06 [0.04-0.09]	−53.85	0.33 [0.23-0.47]	0.08 [0.04-0.11]	-3.15 [-4.39 to -1.9]
Azerbaijan	Male	0 [0-0.01]	0.01 [0-0.02]	Inf	0.02 [0.01-0.04]	0.03 [0.01-0.06]	2.05 [1.12 to 3]

Bahamas	Male	0 [0-0]	0 [0-0]	NaN	0.09 [0.06-0.13]	0.13 [0.07-0.18]	1.19 [0.88 to 1.49]
Bahrain	Male	0 [0-0]	0 [0-0]	NaN	0.02 [0.01-0.05]	0.06 [0.01-0.19]	4.38 [3.27 to 5.51]
Bangladesh	Male	0.35 [0.12-0.65]	0.41 [0.18-0.77]	17.14	0.13 [0.04-0.24]	0.07 [0.03-0.12]	-1.64 [-2.07 to -1.21]
Barbados	Male	0 [0-0]	0.01 [0-0.01]	Inf	0.2 [0.14-0.35]	0.26 [0.12-0.37]	0.76 [0.43 to 1.09]
Belarus	Male	0.28 [0.17-0.39]	0.29 [0.13-0.48]	3.57	0.66 [0.4-0.91]	0.55 [0.24-0.89]	-1.03 [-1.27 to -0.78]
Belgium	Male	0.04 [0.02-0.06]	0.1 [0.06-0.19]	150	0.07 [0.04-0.11]	0.1 [0.06-0.18]	0.91 [0.36 to 1.46]
Belize	Male	0 [0-0]	0 [0-0]	NaN	0.07 [0.04-0.12]	0.19 [0.1-0.26]	3.03 [2 to 4.07]
Benin	Male	0 [0-0.01]	0.01 [0-0.01]	Inf	0.04 [0.02-0.07]	0.04 [0.02-0.07]	0.62 [0.46 to 0.78]
Bermuda	Male	0 [0-0]	0 [0-0]	NaN	0.05 [0.03-0.08]	0.05 [0.03-0.07]	0.54 [0.36 to 0.73]
Bhutan	Male	0 [0-0.01]	0 [0-0.01]	NaN	0.25 [0.05-0.66]	0.15 [0.05-0.3]	-1.78 [-1.92 to -1.63]
Bolivia (Plurinational State of)	Male	0.03 [0.01-0.07]	0.06 [0.02-0.11]	100	0.23 [0.06-0.5]	0.16 [0.06-0.28]	-1.04 [-1.17 to -0.91]
Bosnia and Herzegovina	Male	0.02 [0.01-0.03]	0.01 [0-0.02]	−50.00	0.12 [0.05-0.19]	0.03 [0.01-0.06]	-5.69 [-6.84 to -4.54]
Botswana	Male	0 [0-0]	0 [0-0.01]	NaN	0.08 [0.04-0.16]	0.08 [0.04-0.17]	-0.33 [-0.97 to 0.31]
Brazil	Male	0.47 [0.34-0.66]	2.32 [1.67-4.36]	393.62	0.12 [0.08-0.16]	0.23 [0.16-0.43]	3.07 [2.86 to 3.28]
Brunei Darussalam	Male	0 [0-0]	0 [0-0]	NaN	0.72 [0.28-1.69]	0.47 [0.2-0.9]	-0.63 [-1.4 to 0.15]
Bulgaria	Male	0.48 [0.3-0.92]	0.04 [0.01-0.06]	−91.67	0.93 [0.6-1.66]	0.06 [0.02-0.1]	-10.33 [-11.78 to -8.84]
Burkina Faso	Male	0.01 [0-0.01]	0.01 [0.01-0.02]	0	0.03 [0.01-0.06]	0.02 [0.01-0.06]	-0.57 [-0.83 to -0.31]
Burundi	Male	0 [0-0.01]	0.01 [0-0.01]	Inf	0.03 [0.01-0.06]	0.02 [0.01-0.05]	-0.99 [-1.12 to -0.86]
Cabo Verde	Male	0 [0-0]	0 [0-0]	NaN	0.01 [0-0.02]	0.02 [0.01-0.04]	-0.27 [-1.04 to 0.51]
Cambodia	Male	0.15 [0.01-0.31]	0.28 [0.03-0.48]	86.67	0.83 [0.08-1.78]	0.65 [0.08-1.08]	-0.98 [-1.08 to -0.88]
Cameroon	Male	0.01 [0-0.01]	0.02 [0.01-0.04]	100	0.04 [0.02-0.07]	0.05 [0.02-0.09]	0.83 [0.46 to 1.2]
Canada	Male	0.13 [0.1-0.2]	0.37 [0.27-0.61]	184.62	0.11 [0.08-0.17]	0.12 [0.09-0.2]	1 [0.64 to 1.36]
Democratic Republic	Male	0.01 [0.01-0.03]	0.03 [0.01-0.06]	200	0.28 [0.09-0.54]	0.29 [0.1-0.63]	0.09 [-0.03 to 0.2]
Chad	Male	0 [0-0.01]	0.01 [0-0.02]	Inf	0.03 [0.01-0.06]	0.04 [0.01-0.08]	0.99 [0.5 to 1.47]
Chile	Male	0.03 [0.02-0.04]	0.07 [0.04-0.1]	133.33	0.07 [0.05-0.1]	0.07 [0.04-0.1]	-0.3 [-0.76 to 0.17]
China	Male	24.44 [9.37-32.03]	17.24 [9.89-26.48]	−29.46	0.83 [0.31-1.11]	0.24 [0.13-0.37]	-4.45 [-4.79 to -4.12]
Colombia	Male	0.05 [0.03-0.07]	0.12 [0.07-0.18]	140	0.05 [0.03-0.07]	0.05 [0.03-0.08]	1.22 [0.19 to 2.25]
Comoros	Male	0 [0-0]	0 [0-0]	NaN	0.02 [0.01-0.04]	0.02 [0.01-0.04]	-0.8 [-0.99 to -0.61]
Congo	Male	0.01 [0-0.02]	0.02 [0.01-0.03]	100	0.24 [0.1-0.48]	0.15 [0.06-0.26]	-2.23 [-2.55 to -1.91]
Costa Rica	Male	0 [0-0]	0 [0-0.01]	NaN	0.01 [0-0.01]	0.02 [0.01-0.04]	5.12 [4.63 to 5.61]

Cote d'Ivoire	Male	0.01 [0-0.01]	0.02 [0.01-0.03]	100	0.04 [0.02-0.07]	0.05 [0.02-0.09]	0.32 [-0.02 to 0.67]
Croatia	Male	0.02 [0.02-0.04]	0.03 [0.02-0.05]	50	0.11 [0.08-0.17]	0.08 [0.05-0.15]	-0.61 [-1.64 to 0.44]
Cuba	Male	0.09 [0.06-0.15]	0.25 [0.15-0.38]	177.78	0.17 [0.12-0.29]	0.28 [0.17-0.42]	1.66 [1.19 to 2.14]
Cyprus	Male	0.01 [0-0.02]	0 [0-0.01]	−100.00	0.18 [0.02-0.49]	0.04 [0.01-0.13]	-5.5 [-5.96 to -5.04]
Czechia Democratic	Male	0.54 [0.34-1.12]	0.08 [0.05-0.11]	−85.19	1.09 [0.68-2.25]	0.09 [0.06-0.13]	-8.09 [-9.14 to -7.02]
People's Republic of Korea Democratic	Male	0.27 [0.08-0.42]	0.44 [0.14-0.72]	62.96	0.5 [0.16-0.84]	0.39 [0.13-0.65]	-0.96 [-1.26 to -0.66]
Republic of the Congo	Male	0.11 [0.04-0.21]	0.21 [0.08-0.46]	90.91	0.17 [0.06-0.31]	0.15 [0.06-0.33]	-0.36 [-0.51 to -0.21]
Denmark	Male	0.07 [0.05-0.1]	0.12 [0.08-0.19]	71.43	0.2 [0.16-0.32]	0.24 [0.16-0.38]	0.82 [0.22 to 1.43]
Djibouti	Male	0 [0-0]	0 [0-0]	NaN	0.02 [0.01-0.04]	0.02 [0.01-0.05]	0.32 [-0.11 to 0.75]
Dominica	Male	0 [0-0]	0 [0-0]	NaN	0.04 [0.02-0.08]	0.08 [0.04-0.14]	2.63 [2.35 to 2.92]
Dominican Republic	Male	0.01 [0-0.02]	0.02 [0.01-0.03]	100	0.06 [0.02-0.09]	0.04 [0.02-0.08]	-0.15 [-1.07 to 0.78]
Ecuador	Male	0.02 [0.01-0.04]	0.07 [0.03-0.13]	250	0.09 [0.04-0.15]	0.11 [0.04-0.21]	2.22 [1.74 to 2.71]
Egypt	Male	0.01 [0-0.03]	0.03 [0.01-0.07]	200	0.01 [0-0.02]	0.01 [0-0.02]	1.09 [0.8 to 1.38]
El Salvador	Male	0.01 [0-0.01]	0.01 [0-0.02]	0	0.06 [0.02-0.1]	0.04 [0.01-0.07]	-1.43 [-2.24 to -0.62]
Equatorial Guinea	Male	0 [0-0]	0 [0-0]	NaN	0.26 [0.08-0.53]	0.13 [0.05-0.26]	-2.73 [-3.3 to -2.16]
Eritrea	Male	0 [0-0.01]	0 [0-0.01]	NaN	0.03 [0.01-0.06]	0.03 [0.01-0.06]	-0.48 [-0.73 to -0.24]
Estonia	Male	0.06 [0.04-0.11]	0.02 [0.01-0.04]	−66.67	0.95 [0.68-1.85]	0.25 [0.16-0.4]	-6.55 [-7.45 to -5.64]
Eswatini	Male	0 [0-0]	0 [0-0.01]	NaN	0.1 [0.05-0.19]	0.13 [0.07-0.23]	1.1 [0.31 to 1.91]
Ethiopia	Male	0.07 [0.02-0.19]	0.05 [0.02-0.09]	−28.57	0.06 [0.02-0.13]	0.02 [0.01-0.04]	-3.58 [-3.71 to -3.46]
Fiji	Male	0 [0-0]	0 [0-0]	NaN	0.04 [0.02-0.09]	0.08 [0.03-0.15]	4.52 [3.73 to 5.31]
Finland	Male	0.01 [0-0.01]	0.03 [0.01-0.05]	200	0.02 [0.02-0.05]	0.06 [0.03-0.08]	4.68 [3.5 to 5.88]
France	Male	0.38 [0.28-0.52]	0.34 [0.22-0.48]	−10.53	0.13 [0.09-0.18]	0.05 [0.03-0.08]	-2.82 [-3.2 to -2.43]
Gabon	Male	0 [0-0.01]	0.01 [0-0.02]	Inf	0.21 [0.08-0.48]	0.18 [0.08-0.38]	-0.55 [-0.71 to -0.38]
Gambia	Male	0 [0-0]	0 [0-0]	NaN	0.03 [0.01-0.05]	0.05 [0.02-0.09]	2 [1.78 to 2.21]
Germany	Male	0.38 [0.26-0.52]	0.51 [0.32-0.74]	34.21	0.09 [0.06-0.12]	0.06 [0.04-0.08]	-0.84 [-1.65 to -0.03]
Ghana	Male	0.12 [0-0.29]	0.3 [0.01-0.58]	150	0.56 [0.01-1.42]	0.6 [0.01-1.13]	1.11 [0.76 to 1.45]
Greece	Male	0.01 [0-0.01]	0.01 [0.01-0.03]	0	0.01 [0.01-0.02]	0.01 [0.01-0.02]	1.26 [0.34 to 2.19]
Greenland	Male	0 [0-0]	0 [0-0]	NaN	0.08 [0.01-0.21]	0.02 [0.01-0.04]	-6 [-7.17 to -4.81]
Grenada	Male	0 [0-0]	0 [0-0]	NaN	0.28 [0.2-0.44]	0.55 [0.32-0.8]	2.83 [2.02 to 3.63]
Guam	Male	0 [0-0]	0 [0-0]	NaN	0.09 [0.02-0.17]	0.02 [0.01-0.04]	-4.91 [-6.74 to -3.05]

Guatemala	Male	0.03 [0.02-0.04]	0.05 [0.02-0.07]	66.67	0.13 [0.08-0.18]	0.09 [0.05-0.13]	-1.53 [-2.05 to -1.01]
Guinea	Male	0 [0-0.01]	0.01 [0-0.02]	Inf	0.03 [0.01-0.07]	0.05 [0.02-0.09]	1.74 [1.52 to 1.97]
Guinea-Bissau	Male	0 [0-0]	0 [0-0]	NaN	0.06 [0.03-0.12]	0.06 [0.02-0.11]	-0.05 [-0.27 to 0.17]
Guyana	Male	0 [0-0.01]	0.01 [0.01-0.02]	Inf	0.25 [0.18-0.41]	0.36 [0.19-0.51]	1.44 [1.09 to 1.8]
Haiti	Male	0.07 [0.02-0.15]	0.13 [0.04-0.32]	85.71	0.41 [0.11-0.99]	0.41 [0.11-1.04]	0.37 [0.21 to 0.52]
Honduras	Male	0.04 [0.01-0.07]	0.08 [0.02-0.17]	100	0.36 [0.07-0.65]	0.32 [0.08-0.64]	-0.19 [-0.54 to 0.16]
Hungary	Male	0.67 [0.43-0.97]	0.21 [0.14-0.3]	−68.66	1.29 [0.85-1.85]	0.3 [0.19-0.42]	-4.7 [-5.39 to -4]
Iceland	Male	0 [0-0]	0 [0-0.01]	NaN	0.16 [0.12-0.23]	0.14 [0.1-0.19]	-0.27 [-0.86 to 0.33]
India	Male	7.92 [2.39-14.27]	9.92 [4.93-17.51]	25.25	0.45 [0.14-0.84]	0.21 [0.1-0.38]	-2.77 [-2.96 to -2.58]
Indonesia	Male	3.85 [0.31-6.51]	7.17 [0.67-11.59]	86.23	0.86 [0.08-1.46]	0.79 [0.09-1.25]	-0.11 [-0.18 to -0.03]
Iran (Islamic Republic of)	Male	0.02 [0.01-0.06]	0.12 [0.02-0.22]	500	0.02 [0.01-0.05]	0.04 [0.01-0.07]	2.57 [1.49 to 3.67]
Iraq	Male	0.07 [0.03-0.14]	0.05 [0.03-0.08]	−28.57	0.16 [0.07-0.28]	0.05 [0.02-0.1]	-5.11 [-5.83 to -4.39]
Ireland	Male	0.02 [0.02-0.04]	0.02 [0.01-0.02]	0	0.16 [0.11-0.22]	0.06 [0.03-0.08]	-3.85 [-5.15 to -2.53]
Israel	Male	0.02 [0.01-0.04]	0.08 [0.04-0.12]	300	0.08 [0.05-0.19]	0.15 [0.07-0.23]	1.42 [0.52 to 2.32]
Italy	Male	0.77 [0.54-0.96]	0.43 [0.29-0.61]	−44.16	0.24 [0.17-0.3]	0.06 [0.04-0.09]	-4.18 [-4.89 to -3.47]
Jamaica	Male	0 [0-0.01]	0.02 [0.01-0.04]	Inf	0.04 [0.03-0.09]	0.16 [0.09-0.25]	6.08 [5.14 to 7.03]
Japan	Male	0.47 [0.29-0.68]	1.72 [1.21-2.59]	265.96	0.08 [0.05-0.11]	0.1 [0.07-0.14]	1.5 [1.26 to 1.74]
Jordan	Male	0 [0-0]	0 [0-0.01]	NaN	0.02 [0.01-0.03]	0.01 [0.01-0.02]	-1 [-1.15 to -0.85]
Kazakhstan	Male	0.38 [0.23-0.81]	0.62 [0.42-0.9]	63.16	0.99 [0.58-2.06]	1.09 [0.76-1.69]	-0.14 [-0.49 to 0.21]
Kenya	Male	0.01 [0-0.01]	0.02 [0.01-0.03]	100	0.02 [0.01-0.03]	0.02 [0.01-0.04]	0.78 [0.39 to 1.18]
Kuwait	Male	0 [0-0]	0 [0-0]	NaN	0.01 [0.01-0.01]	0.02 [0.01-0.04]	5.75 [4.85 to 6.66]
Kyrgyzstan	Male	0.06 [0.04-0.09]	0.05 [0.03-0.07]	−16.67	0.39 [0.28-0.6]	0.21 [0.13-0.3]	-2.64 [-3.22 to -2.05]
Lao People's Democratic Republic	Male	0.09 [0.01-0.21]	0.1 [0.01-0.2]	11.11	0.94 [0.07-2.34]	0.53 [0.06-1.1]	-2.32 [-2.56 to -2.08]
Latvia	Male	0.12 [0.09-0.18]	0.1 [0.06-0.15]	−16.67	1.1 [0.81-1.63]	0.75 [0.44-1.06]	-2.6 [-3.2 to -1.99]
Lebanon	Male	0 [0-0]	0 [0-0]	NaN	0.01 [0-0.03]	0.01 [0-0.02]	2.04 [1.42 to 2.66]
Lesotho	Male	0 [0-0.01]	0.01 [0-0.01]	Inf	0.09 [0.04-0.2]	0.15 [0.08-0.26]	2.3 [1.79 to 2.8]
Liberia	Male	0 [0-0]	0 [0-0.01]	NaN	0.04 [0.01-0.08]	0.04 [0.01-0.08]	-0.06 [-0.28 to 0.15]
Libya	Male	0 [0-0]	0 [0-0.01]	NaN	0.01 [0-0.03]	0.01 [0-0.02]	3.3 [2.89 to 3.71]
Lithuania	Male	0.16 [0.12-0.22]	0.07 [0.05-0.1]	−56.25	0.99 [0.72-1.38]	0.32 [0.22-0.47]	-5.17 [-5.72 to -4.63]
Luxembourg	Male	0 [0-0]	0 [0-0]	NaN	0.12 [0.09-0.2]	0.05 [0.04-0.08]	-2.48 [-2.75 to -2.2]

Madagascar	Male	0.01 [0-0.02]	0.01 [0-0.02]	0	0.02 [0.01-0.04]	0.02 [0.01-0.03]	-0.54 [-0.64 to -0.45]
Malawi	Male	0.01 [0-0.02]	0.01 [0-0.02]	0	0.03 [0.01-0.05]	0.03 [0.01-0.05]	-0.27 [-0.53 to -0.02]
Malaysia	Male	0.04 [0.01-0.07]	0.1 [0.03-0.26]	150	0.11 [0.03-0.19]	0.09 [0.03-0.24]	-0.65 [-1.18 to -0.13]
Maldives	Male	0 [0-0]	0 [0-0]	NaN	0.11 [0.03-0.24]	0.04 [0.02-0.07]	-4.19 [-4.45 to -3.92]
Mali	Male	0.01 [0-0.01]	0.01 [0-0.02]	0	0.03 [0.01-0.07]	0.03 [0.01-0.07]	0.39 [0.15 to 0.64]
Malta	Male	0 [0-0.01]	0 [0-0.01]	NaN	0.25 [0.17-0.33]	0.12 [0.08-0.17]	-2.04 [-2.45 to -1.63]
Marshall Islands	Male	0 [0-0]	0 [0-0]	NaN	0.24 [0.06-0.57]	0.15 [0.05-0.33]	-1.39 [-1.59 to -1.19]
Mauritania	Male	0 [0-0]	0 [0-0]	NaN	0.04 [0.02-0.06]	0.03 [0.01-0.04]	-0.88 [-1.01 to -0.74]
Mauritius	Male	0 [0-0]	0 [0-0]	NaN	0.05 [0.03-0.07]	0.02 [0.01-0.05]	-1.98 [-3.23 to -0.72]
Mexico	Male	0.85 [0.6-1.07]	1.81 [1.33-2.78]	112.94	0.42 [0.29-0.54]	0.34 [0.25-0.52]	-0.8 [-1.09 to -0.5]
Micronesia (Federated States of)	Male	0 [0-0]	0 [0-0]	NaN	0.25 [0.06-0.55]	0.15 [0.06-0.3]	-1.79 [-1.91 to -1.66]
Mongolia	Male	0 [0-0.01]	0 [0-0.01]	NaN	0.06 [0.02-0.15]	0.03 [0.01-0.06]	-4.04 [-4.7 to -3.37]
Montenegro	Male	0 [0-0]	0 [0-0]	NaN	0.01 [0-0.01]	0.01 [0-0.01]	0.29 [0.09 to 0.48]
Morocco	Male	0.01 [0-0.03]	0.02 [0.01-0.04]	100	0.01 [0-0.04]	0.02 [0-0.03]	2.48 [2.01 to 2.96]
Mozambique	Male	0.01 [0-0.02]	0.02 [0.01-0.04]	100	0.03 [0.01-0.06]	0.03 [0.01-0.08]	1.05 [0.87 to 1.24]
Myanmar	Male	0.87 [0.07-1.92]	1.05 [0.12-1.92]	20.69	0.87 [0.08-1.99]	0.59 [0.07-1.07]	-1.53 [-1.81 to -1.26]
Namibia	Male	0[0-0.01]	0[0-0.01]	NaN	0.09[0.04-0.18]	0.08[0.04-0.15]	-0.8[-1.33to-0.27]
Nepal	Male	0.09[0.02-0.2]	0.14[0.05-0.32]	55.56	0.22[0.05-0.55]	0.16[0.06-0.37]	-0.76[-1.12to-0.39]
Netherlands	Male	0.24[0.15-0.32]	0.21[0.13-0.33]	−12.50	0.33[0.2-0.44]	0.15[0.09-0.23]	-2.79[-3.17to-2.4]
New Zealand	Male	0.04[0.03-0.06]	0.06[0.04-0.09]	50	0.25[0.18-0.39]	0.17[0.12-0.24]	-1.09[-1.71to-0.46]
Nicaragua	Male	0.01[0-0.01]	0.01[0.01-0.02]	0	0.09[0.03-0.14]	0.09[0.03-0.14]	-0.76[-1.55to0.03]
Niger	Male	0[0-0.01]	0.01[0-0.02]	Inf	0.04[0.01-0.08]	0.03[0.01-0.07]	-0.22[-0.3to-0.14]
Nigeria	Male	0.05[0.02-0.09]	0.11[0.04-0.19]	120	0.03[0.01-0.05]	0.03[0.01-0.06]	0.5[0.36to0.64]
North Macedonia	Male	0[0-0]	0[0-0]	NaN	0.01[0.01-0.02]	0.01[0-0.01]	-2.85[-3.75to-1.95]
Northern Mariana Islands	Male	0[0-0]	0[0-0]	NaN	0.06 [0.02-0.13]	0.04 [0.01-0.07]	-1.92 [-2.66 to -1.17]
Norway	Male	0.11[0.08-0.16]	0.06[0.04-0.12]	−45.45	0.37[0.27-0.58]	0.14[0.1-0.28]	-3.15[-3.73to-2.57]
Oman	Male	0[0-0]	0[0-0]	NaN	0.02[0.01-0.06]	0.03[0.01-0.09]	4.54[3.7to5.39]
Pakistan	Male	0.78[0.21-1.73]	1.16[0.46-2.29]	48.72	0.26[0.06-0.63]	0.25[0.1-0.49]	-0.49[-0.88to-0.09]
Palestine	Male	0[0-0]	0[0-0.01]	NaN	0.05[0.02-0.1]	0.04[0.02-0.11]	-0.76[-1.44to-0.08]
Panama	Male	0[0-0]	0[0-0.01]	NaN	0.01[0.01-0.02]	0.02[0.01-0.03]	3.63[2.56to4.7]

Papua New Guinea	Male	0.01[0-0.03]	0.02[0.01-0.05]	100	0.15[0.04-0.36]	0.1[0.03-0.24]	-1.2[-1.33to-1.06]
Paraguay	Male	0.01[0-0.01]	0.02[0-0.05]	100	0.06[0.01-0.11]	0.08[0.02-0.19]	0.9[0.12to1.68]
Peru	Male	0.03[0.01-0.05]	0.06[0.02-0.11]	100	0.05[0.02-0.09]	0.04[0.02-0.07]	-0.1[-0.78to0.59]
Philippines	Male	1.66[1.03-2.58]	3.13[2.05-5.85]	88.55	1.2[0.78-2.2]	0.96[0.62-2]	-0.84[-1to-0.69]
Poland	Male	0.83[0.53-1.21]	0.13[0.08-0.18]	−84.34	0.49[0.32-0.72]	0.05[0.03-0.07]	-8.91[-10.24to-7.56]
Portugal	Male	0.06[0.03-0.08]	0.08[0.04-0.15]	33.33	0.12[0.05-0.15]	0.07[0.04-0.14]	-1.35[-2.87to0.19]
Puerto Rico	Male	0[0-0]	0.01[0.01-0.02]	Inf	0.01[0-0.01]	0.03[0.02-0.05]	7.04[6.03to8.06]
Qatar	Male	0[0-0]	0[0-0]	NaN	0.02[0.01-0.04]	0.01[0.01-0.02]	-1.4[-1.96to-0.84]
Republic of Korea	Male	0.11[0.06-0.2]	0.23[0.11-0.4]	109.09	0.19[0.08-0.42]	0.09[0.04-0.17]	-4.07[-4.89to-3.24]
Republic of Moldova	Male	0.06[0.03-0.09]	0.07[0.04-0.09]	16.67	0.42 [0.23-0.58]	0.3 [0.17-0.4]	-1.26 [-2.06 to -0.46
Romania	Male	0.01[0-0.01]	0.02[0.01-0.02]	100	0.01[0-0.01]	0.01[0.01-0.01]	2.41[2.05to2.77]
Russian Federation	Male	5.42 [3.15-8.89]	5.37 [3.25-7.11]	−0.92	1.1 [0.61-1.93]	0.7 [0.42-0.92]	-1.69 [-2.26 to -1.11]
Rwanda	Male	0.01 [0-0.01]	0 [0-0.01]	−100.00	0.03 [0.01-0.07]	0.02 [0.01-0.04]	-2.38 [-2.74 to -2.01]
Saint Lucia	Male	0 [0-0]	0 [0-0]	NaN	0.03 [0.02-0.04]	0.04 [0.02-0.05]	0.74 [0.39 to 1.09]
Saint Vincent and the Grenadines	Male	0 [0-0]	0 [0-0]	NaN	0.29 [0.2-0.45]	0.32 [0.18-0.44]	0.69 [0.24 to 1.15]
Samoa	Male	0 [0-0]	0 [0-0]	NaN	0.18 [0.06-0.42]	0.09 [0.03-0.18]	-2.62 [-2.88 to -2.36]
Sao Tome and Principe	Male	0 [0-0]	0 [0-0]	NaN	0.02 [0.01-0.03]	0.03 [0.01-0.06]	1.96 [1.71 to 2.22]
Saudi Arabia	Male	0 [0-0.01]	0.01 [0-0.02]	Inf	0.01 [0-0.03]	0.01 [0-0.03]	1.55 [1.13 to 1.98]
Senegal	Male	0.01 [0-0.01]	0.01 [0-0.02]	0	0.04 [0.02-0.08]	0.04 [0.02-0.08]	0.62 [0.4 to 0.85]
Serbia	Male	0.04 [0.02-0.07]	0.04 [0.02-0.07]	0	0.09 [0.04-0.15]	0.06 [0.03-0.09]	-1.71 [-1.95 to -1.47]
Seychelles	Male	0 [0-0]	0 [0-0]	NaN	0.26 [0.04-0.48]	0.42 [0.04-0.9]	1.21 [0.73 to 1.69]
Sierra Leone	Male	0 [0-0.01]	0.01 [0-0.01]	Inf	0.03 [0.01-0.06]	0.04 [0.01-0.08]	0.76 [0.48 to 1.04]
Singapore	Male	0.01 [0.01-0.02]	0.01 [0.01-0.01]	0	0.16 [0.1-0.23]	0.03 [0.02-0.05]	-4.13 [-6.07 to -2.16]
Slovakia	Male	0.05 [0.01-0.08]	0.02 [0.01-0.03]	−60.00	0.21 [0.06-0.34]	0.05 [0.03-0.09]	-5.09 [-5.48 to -4.7]
Slovenia	Male	0.01 [0.01-0.02]	0.01 [0-0.01]	0	0.12 [0.08-0.26]	0.03 [0.02-0.06]	-4.61 [-5.63 to -3.57]
Solomon Islands	Male	0 [0-0]	0 [0-0]	NaN	0.21 [0.06-0.47]	0.15 [0.05-0.3]	-1.11 [-1.21 to -1.01]
Somalia	Male	0 [0-0.02]	0.01 [0-0.05]	Inf	0.03 [0.01-0.07]	0.03 [0.01-0.11]	0.49 [0.3 to 0.69]
South Africa	Male	0.07 [0.04-0.11]	0.11 [0.07-0.18]	57.14	0.07 [0.04-0.12]	0.07 [0.04-0.11]	-0.19 [-1.05 to 0.68]
South Sudan	Male	0 [0-0.01]	0 [0-0.01]	NaN	0.02 [0.01-0.05]	0.02 [0.01-0.04]	-0.34 [-0.46 to -0.23]
Spain	Male	0.24 [0.15-0.3]	0.27 [0.17-0.41]	12.5	0.12 [0.08-0.15]	0.06 [0.04-0.09]	-2.14 [-2.51 to -1.77]

Sri Lanka	Male	0.01 [0-0.02]	0.03 [0.01-0.05]	200	0.03 [0.01-0.05]	0.03 [0.01-0.06]	-0.68 [-1.24 to -0.12]
Sudan	Male	0.01 [0-0.02]	0.02 [0-0.03]	100	0.01 [0-0.05]	0.02 [0-0.04]	2.4 [1.92 to 2.89]
Suriname	Male	0 [0-0.01]	0.02 [0.01-0.03]	Inf	0.29 [0.15-0.54]	0.57 [0.27-0.92]	2.34 [1.78 to 2.9]
Sweden	Male	0.16 [0.11-0.27]	0.18 [0.13-0.26]	12.5	0.26 [0.18-0.44]	0.17 [0.12-0.24]	-0.45 [-1.24 to 0.34]
Switzerland	Male	0.03 [0.02-0.07]	0.04 [0.02-0.06]	33.33	0.08 [0.04-0.16]	0.04 [0.03-0.07]	-2.01 [-2.32 to -1.7]
Syrian Arab Republic	Male	0.01 [0-0.03]	0.01 [0-0.03]	0	0.05 [0.01-0.1]	0.02 [0-0.04]	-4.1 [-4.63 to -3.56]
Tajikistan	Male	0.04 [0.01-0.05]	0.09 [0.04-0.15]	125	0.26 [0.11-0.43]	0.44 [0.24-0.82]	1.9 [1.6 to 2.19]
Thailand	Male	1.57 [0.42-2.62]	2.43 [0.74-4.09]	54.78	1.13 [0.32-1.88]	0.57 [0.17-0.95]	-3.23 [-4.12 to -2.33]
Timor-Leste	Male	0.01 [0-0.02]	0.02 [0-0.03]	100	0.5 [0.04-1.38]	0.49 [0.06-0.96]	-0.13 [-0.32 to 0.07]
Togo	Male	0 [0-0]	0 [0-0.01]	NaN	0.03 [0.01-0.05]	0.04 [0.01-0.08]	1.15 [0.75 to 1.54]
Tonga	Male	0 [0-0]	0 [0-0]	NaN	0.27 [0.08-0.5]	0.23 [0.08-0.42]	-0.66 [-0.9 to -0.43]
Trinidad and Tobago	Male	0.02 [0.01-0.03]	0.07 [0.04-0.13]	250	0.43 [0.32-0.8]	0.82 [0.5-1.43]	3.56 [2.92 to 4.21]
Tunisia	Male	0 [0-0.01]	0 [0-0.01]	NaN	0.01 [0-0.03]	0.01 [0-0.02]	1.93 [1.55 to 2.31]
Turkey	Male	0.09 [0.03-0.23]	0.23 [0.11-0.35]	155.56	0.05 [0.01-0.15]	0.06 [0.03-0.09]	2.88 [1.86 to 3.91]
Turkmenistan	Male	0.01 [0-0.01]	0.03 [0.01-0.05]	200	0.07 [0.03-0.1]	0.2 [0.09-0.3]	4.04 [3.52 to 4.56]
Uganda	Male	0.01 [0-0.01]	0.01 [0-0.03]	0	0.02 [0.01-0.03]	0.02 [0.01-0.04]	-0.01 [-0.39 to 0.36]
Ukraine	Male	1.44 [1.02-2.48]	1.57 [0.97-2.21]	9.03	0.61 [0.43-1.08]	0.57 [0.35-0.8]	-1.09 [-1.47 to -0.72]
United Arab Emirates	Male	0 [0-0]	0.01 [0-0.01]	Inf	0.02 [0-0.06]	0.03 [0.01-0.06]	2.27 [1.86 to 2.68]
United Kingdom	Male	0.62 [0.34-0.77]	1.02 [0.68-1.58]	64.52	0.19 [0.1-0.23]	0.18 [0.12-0.27]	0.26 [-0.27 to 0.79]
United Republic of Tanzania	Male	0.01 [0-0.01]	0.01 [0-0.02]	0	0.01 [0-0.02]	0.01 [0-0.02]	0.01 [-0.16 to 0.18]
United States of America	Male	1.2 [0.82-1.71]	2.11 [1.53-3.06]	75.83	0.1 [0.07-0.13]	0.09 [0.06-0.12]	0.19 [-0.15 to 0.53]
United States Virgin Islands	Male	0 [0-0]	0 [0-0.01]	NaN	0.23 [0.1-0.61]	0.56 [0.29-0.89]	4.35 [3.75 to 4.94]
Uruguay	Male	0 [0-0.01]	0.01 [0.01-0.02]	Inf	0.03 [0.02-0.06]	0.06 [0.03-0.09]	3.52 [2.56 to 4.49]
Uzbekistan	Male	0 [0-0]	0.01 [0-0.01]	Inf	0 [0-0.01]	0.01 [0.01-0.02]	4.56 [3.91 to 5.22]
Vanuatu	Male	0 [0-0]	0 [0-0]	NaN	0.18 [0.04-0.42]	0.14 [0.04-0.33]	-0.75 [-0.94 to -0.57]
Venezuela (Bolivarian Republic of)	Male	0.07 [0.05-0.15]	0.32 [0.17-0.47]	357.14	0.16 [0.11-0.33]	0.25 [0.13-0.37]	0.39 [-0.66 to 1.46]
Viet Nam	Male	0.28 [0.02-0.59]	0.46 [0.04-0.95]	64.29	0.18 [0.01-0.37]	0.13 [0.01-0.26]	-1.5 [-2.02 to -0.98]
Yemen	Male	0 [0-0.01]	0.01 [0-0.02]	Inf	0.01 [0-0.05]	0.02 [0-0.04]	2.61 [2.19 to 3.04]
Zambia	Male	0 [0-0.01]	0.01 [0-0.02]	Inf	0.02 [0.01-0.05]	0.02 [0.01-0.05]	-0.28 [-0.75 to 0.19]
Zimbabwe	Male	0.02 [0.01-0.03]	0.04 [0.01-0.08]	100	0.08 [0.04-0.13]	0.12 [0.05-0.19]	1.23 [0.53 to 1.93]

Afghanistan	Female	0.01 [0-0.04]	0.05 [0.02-0.13]	400	0.04 [0.01-0.14]	0.06 [0.02-0.17]	2.5 [2.19 to 2.81]
Albania	Female	0 [0-0]	0 [0-0]	NaN	0.02 [0.01-0.02]	0.01 [0-0.04]	-3.27 [-3.85 to -2.68]
Algeria	Female	0.01 [0-0.02]	0.03 [0.01-0.06]	200	0.02 [0-0.05]	0.02 [0.01-0.03]	1.88 [1.43 to 2.33]
American Samoa	Female	0 [0-0]	0 [0-0]	NaN	0.18 [0.05-0.31]	0.1 [0.05-0.16]	-5.99 [-7.06 to -4.9]
Andorra	Female	0 [0-0]	0 [0-0]	NaN	0.15 [0.06-0.26]	0.13 [0.06-0.19]	-0.35 [-0.72 to 0.02]
Angola	Female	0.02 [0.01-0.05]	0.04 [0.01-0.07]	100	0.09 [0.04-0.2]	0.11 [0.06-0.2]	-1.65 [-1.94 to -1.35]
Antigua and Barbuda	Female	0 [0-0]	0 [0-0]	NaN	0.07 [0.05-0.09]	0.12 [0.09-0.16]	3 [2.62 to 3.37]
Argentina	Female	0.01 [0.01-0.02]	0.07 [0.05-0.11]	600	0.01 [0-0.01]	0.03 [0.02-0.04]	4.8 [4.05 to 5.55]
Armenia	Female	0.08 [0.05-0.12]	0.46 [0.2-1.25]	475	0.61 [0.39-0.84]	1.82 [0.94-4.03]	5.92 [5.1 to 6.75]
Australia	Female	0.17 [0.12-0.21]	0.21 [0.16-0.32]	23.53	0.16 [0.11-0.19]	0.09 [0.07-0.12]	-2.02 [-2.54 to -1.48]
Austria	Female	0.36 [0.26-0.44]	0.12 [0.06-0.16]	−66.67	0.43 [0.32-0.54]	0.09 [0.06-0.11]	-3.54 [-4.99 to -2.07]
Azerbaijan	Female	0.01 [0-0.03]	0.05 [0.01-0.11]	400	0.05 [0.02-0.1]	0.13 [0.02-0.29]	5.35 [4.3 to 6.42]
Bahamas	Female	0 [0-0]	0 [0-0]	NaN	0.06 [0.04-0.08]	0.11 [0.08-0.15]	1.76 [1.38 to 2.14]
Bahrain	Female	0 [0-0]	0 [0-0]	NaN	0.03 [0.01-0.07]	0.05 [0.02-0.16]	1.43 [-0.26 to 3.15]
Bangladesh	Female	0.27 [0.1-0.46]	0.33 [0.12-0.86]	22.22	0.11 [0.05-0.22]	0.06 [0.03-0.12]	-2.6 [-2.86 to -2.33]
Barbados	Female	0 [0-0]	0 [0-0]	NaN	0.07 [0.05-0.09]	0.18 [0.11-0.24]	2.59 [2.25 to 2.94]
Belarus	Female	0.38 [0.29-0.53]	0.39 [0.19-0.6]	2.63	0.46 [0.36-0.66]	0.42 [0.26-0.61]	-1.2 [-1.46 to -0.95]
Belgium	Female	0.06 [0.04-0.08]	0.23 [0.13-0.55]	283.33	0.07 [0.04-0.08]	0.12 [0.08-0.23]	3.06 [2.52 to 3.59]
Belize	Female	0 [0-0]	0 [0-0]	NaN	0.1 [0.06-0.13]	0.19 [0.13-0.23]	2.36 [1.6 to 3.12]
Benin	Female	0.01 [0.01-0.03]	0.02 [0.01-0.03]	100	0.15 [0.06-0.29]	0.06 [0.04-0.09]	-2.73 [-2.91 to -2.55]
Bermuda	Female	0 [0-0]	0 [0-0]	NaN	0.09 [0.06-0.13]	0.08 [0.06-0.11]	0.78 [0.26 to 1.29]
Bhutan	Female	0 [0-0.01]	0 [0-0.01]	NaN	0.21 [0.09-0.52]	0.14 [0.06-0.29]	-1.38 [-1.44 to -1.31]
Bolivia (Plurinational State of)	Female	0.02 [0.01-0.04]	0.06 [0.02-0.09]	200	0.13 [0.05-0.22]	0.14 [0.06-0.21]	-0.07 [-0.27 to 0.13]
Bosnia and Herzegovina	Female	0.02 [0.01-0.04]	0.01 [0-0.02]	− 50.00	0.1 [0.05-0.17]	0.03 [0.02-0.05]	-6.27 [-7.55 to -4.97]
Botswana	Female	0[0-0]	0[0-0.01]	NaN	0.04[0.01-0.07]	0.06[0.03-0.1]	0.64[0.43to0.85]
Brazil	Female	0.41[0.28-0.52]	3.16[2.27-5.95]	670.73	0.08[0.06-0.1]	0.24[0.18-0.4]	4.86[4.54to5.17]
Brunei Darussalam	Female	0[0-0]	0[0-0]	NaN	0.29[0.14-0.51]	0.3[0.19-0.45]	-0.07[-0.3to0.17]
Bulgaria	Female	0.51[0.36-0.9]	0.04[0.01-0.06]	−92.16	0.8[0.57-1.38]	0.05[0.02-0.08]	-10.75[-12.48to-8.98]
Burkina Faso	Female	0.04[0.01-0.07]	0.07[0.02-0.13]	75	0.2[0.06-0.37]	0.11[0.05-0.2]	-0.81[-1.01to-0.61]
Burundi	Female	0.03[0.01-0.08]	0.04[0.01-0.1]	33.33	0.3[0.08-0.63]	0.16[0.05-0.32]	-0.8[-0.97to-0.62]

CaboVerde	Female	0[0-0]	0[0-0]	NaN	0.02[0.01-0.04]	0.02[0.01-0.03]	-0.92[-1.34to-0.5]
Cambodia	Female	0.11[0.01-0.18]	0.19[0.02-0.4]	72.73	0.39[0.04-0.71]	0.43[0.06-0.74]	-1.31[-1.5to-1.12]
Cameroon	Female	0.04[0.01-0.07]	0.05[0.02-0.1]	25	0.19[0.05-0.35]	0.07[0.03-0.12]	-3.37[-3.62to-3.12]
Canada	Female	0.13[0.08-0.16]	0.46[0.32-0.77]	253.85	0.07[0.04-0.08]	0.11[0.09-0.16]	2.82[2.15to3.49]
Central African Republic	Female	0.02[0.01-0.05]	0.02 [0.01-0.05]	100	0.14 [0.05-0.29]	0.21 [0.08-0.39]	0.43 [0.15 to 0.72]
Chad	Female	0.02[0.01-0.04]	0.02[0.01-0.04]	0	0.16[0.06-0.33]	0.06[0.04-0.1]	-2.54[-2.72to-2.35]
Chile	Female	0.04[0.03-0.07]	0.08[0.06-0.11]	100	0.08[0.06-0.13]	0.06[0.05-0.08]	-1.98[-2.53to-1.44]
China	Female	15.63[6.64-20.44]	8.35[5.12-11.11]	−46.58	0.4[0.17-0.52]	0.15[0.1-0.2]	-6.03[-6.36to-5.69]
Colombia	Female	0.05[0.03-0.06]	0.21[0.14-0.32]	320	0.04[0.03-0.06]	0.06[0.04-0.09]	2.95[2.12to3.79]
Comoros	Female	0[0-0.01]	0.01[0-0.02]	Inf	0.31[0.09-0.61]	0.19[0.07-0.41]	-0.11[-0.22to0]
Congo	Female	0.01[0-0.01]	0.01[0.01-0.03]	0	0.11[0.05-0.21]	0.12[0.06-0.2]	-0.51[-0.68to-0.34]
Costa Rica	Female	0[0-0]	0.01[0-0.01]	Inf	0[0-0.01]	0.02[0.01-0.04]	8.97[7.56to10.4]
Coted' Ivoire	Female	0.02[0.01-0.04]	0.03[0.02-0.07]	50	0.15[0.04-0.29]	0.06[0.03-0.1]	-2.42[-2.58to-2.26]
Croatia	Female	0.03[0.02-0.04]	0.06[0.03-0.15]	100	0.07[0.05-0.11]	0.09[0.06-0.18]	4.68[2.74to6.65]
Cuba	Female	0.07[0.05-0.09]	0.16[0.11-0.27]	128.57	0.13[0.1-0.17]	0.22[0.16-0.32]	1.58[1.27to1.89]
Cyprus	Female	0[0-0.01]	0.01[0-0.02]	Inf	0.09[0.04-0.24]	0.06[0.03-0.2]	-0.33[-1to0.35]
Czechia	Female	0.64 [0.44-1.31]	0.13 [0.09-0.2]	−79.69	0.75 [0.52-1.53]	0.09 [0.07-0.13]	-6.65 [-7.74 to -5.54]
Democratic People's Republic of Korea	Female	0.2 [0.07-0.34]	0.28 [0.11-0.53]	40	0.21 [0.07-0.36]	0.23 [0.1-0.37]	-1.23 [-1.46 to -1]
Democratic Republic of the Congo	Female	0.07 [0.02-0.14]	0.14 [0.05-0.3]	100	0.08 [0.03-0.17]	0.11 [0.04-0.2]	0.09 [-0.19 to 0.37]
Denmark	Female	0.07 [0.05-0.09]	0.1 [0.07-0.17]	42.86	0.13 [0.1-0.17]	0.17 [0.13-0.26]	0.3 [-0.19 to 0.8]
Djibouti	Female	0 [0-0]	0.01 [0-0.02]	Inf	0.31 [0.14-0.55]	0.21 [0.08-0.46]	0.52 [0.35 to 0.68]
Dominica	Female	0 [0-0]	0 [0-0]	NaN	0.02 [0.01-0.05]	0.06 [0.04-0.09]	2.5 [2.29 to 2.71]
Dominican Republic	Female	0 [0-0]	0.01 [0-0.01]	Inf	0.01 [0.01-0.02]	0.03 [0.02-0.04]	0.4 [-0.02 to 0.83]
Ecuador	Female	0.01 [0.01-0.02]	0.04 [0.02-0.09]	300	0.05 [0.03-0.08]	0.08 [0.05-0.14]	1.17 [0.14 to 2.2]
Egypt	Female	0.01 [0-0.02]	0.02 [0.01-0.05]	100	0.01 [0-0.01]	0.01 [0-0.02]	2.01 [1.73 to 2.29]
El Salvador	Female	0 [0-0]	0.01 [0-0.01]	Inf	0.01 [0.01-0.02]	0.03 [0.01-0.04]	1.18 [0.89 to 1.46]
Equatorial Guinea	Female	0 [0-0]	0 [0-0]	NaN	0.1 [0.04-0.24]	0.1 [0.05-0.18]	-0.92 [-1.21 to -0.63]
Eritrea	Female	0.01 [0-0.03]	0.04 [0.01-0.08]	300	0.29 [0.1-0.57]	0.23 [0.1-0.44]	0.55 [0.43 to 0.68]
Estonia	Female	0.09 [0.05-0.19]	0.03 [0.02-0.05]	−66.67	0.64 [0.4-1.31]	0.19 [0.13-0.26]	-6.39 [-7.2 to -5.57]

Eswatini	Female	0 [0-0]	0 [0-0]	NaN	0.05 [0.01-0.09]	0.07 [0.04-0.11]	-0.7 [-1.48 to 0.09]
Ethiopia	Female	0.54 [0.29-1.03]	0.55 [0.27-0.95]	1.85	0.61 [0.32-1.04]	0.2 [0.11-0.32]	-2.2 [-2.38 to -2.03]
Fiji	Female	0 [0-0]	0 [0-0]	NaN	0.02 [0.01-0.05]	0.05 [0.03-0.08]	3.4 [2.48 to 4.33]
Finland	Female	0.04 [0.02-0.09]	0.03 [0.02-0.04]	−25.00	0.08 [0.05-0.18]	0.04 [0.03-0.06]	-3.19 [-3.7 to -2.68]
France	Female	0.45 [0.3-0.55]	0.63 [0.42-0.93]	40	0.08 [0.05-0.1]	0.06 [0.04-0.08]	-0.86 [-1.11 to -0.61]
Gabon	Female	0 [0-0.01]	0.01 [0-0.01]	Inf	0.13 [0.01-0.31]	0.14 [0.06-0.28]	-0.83 [-1.12 to -0.54]
Gambia	Female	0 [0-0]	0 [0-0.01]	NaN	0.14 [0.05-0.32]	0.07 [0.04-0.12]	-2 [-2.23 to -1.77]
Germany	Female	0.78 [0.5-1.01]	0.88 [0.62-1.47]	12.82	0.1 [0.07-0.13]	0.06 [0.05-0.09]	-0.26 [-1.06 to 0.55]
Ghana	Female	0.04 [0.01-0.08]	0.08 [0.03-0.14]	100	0.13 [0.04-0.31]	0.3 [0.03-0.53]	-1.15 [-1.4 to -0.9]
Greece	Female	0.01 [0.01-0.02]	0.01 [0.01-0.03]	0	0.01 [0.01-0.03]	0.01 [0.01-0.02]	-0.22 [-1.39 to 0.96]
Greenland	Female	0 [0-0]	0 [0-0]	NaN	0.01 [0.01-0.02]	0.02 [0.01-0.02]	0.27 [-0.04 to 0.58]
Grenada	Female	0 [0-0]	0 [0-0]	NaN	0.08 [0.06-0.1]	0.33 [0.23-0.44]	3.32 [3.05 to 3.59]
Guam	Female	0 [0-0]	0 [0-0]	NaN	0.31 [0.04-0.51]	0.02 [0.01-0.04]	-10.89 [-12.32 to -9.44]
Guatemala	Female	0.04 [0.02-0.05]	0.08 [0.05-0.12]	100	0.19 [0.11-0.26]	0.11 [0.08-0.16]	-1.86 [-2.31 to -1.41]
Guinea	Female	0.02 [0.01-0.05]	0.02 [0.01-0.05]	0	0.15 [0.05-0.34]	0.07 [0.04-0.11]	-2.2 [-2.37 to -2.03]
Guinea-Bissau	Female	0 [0-0.01]	0 [0-0.01]	NaN	0.22 [0.08-0.4]	0.09 [0.05-0.14]	-2.66 [-2.82 to -2.51]
Guyana	Female	0 [0-0]	0.01 [0.01-0.01]	Inf	0.12 [0.08-0.18]	0.28 [0.18-0.39]	1.88 [1.29 to 2.47]
Haiti	Female	0.04 [0.02-0.06]	0.09 [0.03-0.16]	125	0.19 [0.08-0.34]	0.31 [0.12-0.65]	0.82 [0.62 to 1.02]
Honduras	Female	0.01 [0-0.02]	0.03 [0.01-0.06]	200	0.11 [0.04-0.17]	0.2 [0.08-0.36]	-0.5 [-0.7 to -0.29]
Hungary	Female	1.04 [0.84-1.43]	0.38 [0.2-0.53]	−63.46	1.19 [0.95-1.64]	0.29 [0.18-0.39]	-4.25 [-5.19 to -3.3]
Iceland	Female	0 [0-0]	0 [0-0]	NaN	0.14 [0.1-0.17]	0.1 [0.07-0.12]	-2.12 [-2.39 to -1.84]
India	Female	6.11 [2.45-9.42]	7.58 [3.59-13.57]	24.06	0.3 [0.12-0.48]	0.17 [0.1-0.26]	-3.19 [-3.51 to -2.88]
Indonesia	Female	0.97 [0.1-1.79]	1.61 [0.18-3.29]	65.98	0.18 [0.02-0.35]	0.44 [0.05-0.7]	-0.53 [-0.7 to -0.35]
Iran (Islamic Republic of)	Female	0.03 [0.01-0.06]	0.12 [0.02-0.25]	300	0.03 [0.01-0.06]	0.04 [0.01-0.06]	1.2 [0.05 to 2.35]
Iraq	Female	0.01 [0.01-0.03]	0.02 [0.01-0.03]	100	0.04 [0.01-0.07]	0.03 [0.02-0.06]	-3.29 [-3.71 to -2.87]
Ireland	Female	0.01 [0.01-0.02]	0.03 [0.02-0.05]	200	0.05 [0.04-0.07]	0.07 [0.05-0.09]	1.28 [0.98 to 1.59]
Israel	Female	0.01 [0.01-0.02]	0.09 [0.05-0.16]	800	0.05 [0.04-0.09]	0.13 [0.09-0.2]	1.26 [-0.19 to 2.73]
Italy	Female	0.65 [0.41-0.75]	0.45 [0.28-0.55]	−30.77	0.12 [0.08-0.14]	0.05 [0.04-0.07]	-2.87 [-3.68 to -2.05]
Jamaica	Female	0 [0-0]	0.01 [0.01-0.02]	Inf	0.01 [0.01-0.02]	0.11 [0.08-0.17]	8.33 [7.06 to 9.62]
Japan	Female	0.55 [0.33-0.67]	4.67 [2.97-6.82]	749.09	0.06 [0.04-0.07]	0.12 [0.09-0.17]	4.15 [3.79 to 4.52]
Jordan	Female	0 [0-0]	0 [0-0.01]	NaN	0.03 [0.01-0.04]	0.01 [0.01-0.02]	-2.84 [-3.49 to -2.17]

Kazakhstan	Female	0.45 [0.24-0.76]	0.74 [0.5-1]	64.44	0.59 [0.32-0.99]	0.88 [0.68-1.16]	0.47 [-0.02 to 0.96]
Kenya	Female	0.08 [0.05-0.15]	0.29 [0.14-0.58]	262.5	0.27 [0.16-0.46]	0.21 [0.11-0.38]	0.7 [0.58 to 0.81]
Kuwait	Female	0 [0-0]	0 [0-0]	NaN	0.01 [0-0.01]	0.01 [0.01-0.03]	2.42 [1.43 to 3.42]
Kyrgyzstan	Female	0.03 [0.02-0.04]	0.02 [0.01-0.02]	−33.33	0.14 [0.1-0.2]	0.13 [0.09-0.17]	-3.3 [-4.47 to -2.12]
Lao People's Democratic Republic	Female	0.05 [0-0.1]	0.06 [0-0.1]	20.00	0.46 [0.04-0.86]	0.38 [0.05-0.69]	-2.44 [-2.69 to -2.2]
Latvia	Female	0.19[0.12-0.36]	0.14[0.09-0.22]	−26.32	0.78[0.52-1.47]	0.53[0.41-0.71]	-2.7[-3.11to-2.29]
Lebanon	Female	0[0-0]	0[0-0.01]	NaN	0.01[0-0.03]	0.01[0-0.02]	2.21[1.83to2.6]
Lesotho	Female	0[0-0]	0[0-0.01]	NaN	0.04[0.01-0.08]	0.09[0.04-0.15]	3.18[2.61to3.75]
Liberia	Female	0.01[0-0.01]	0.01[0-0.02]	0	0.19[0.06-0.32]	0.06[0.04-0.11]	-2.76[-3.05to-2.47]
Libya	Female	0[0-0]	0.01[0-0.01]	Inf	0.01[0-0.04]	0.02[0.01-0.03]	3.78[3.18to4.38]
Lithuania	Female	0.21[0.16-0.3]	0.15[0.11-0.22]	−28.57	0.73[0.57-1.07]	0.34[0.26-0.47]	-3.21[-3.63to-2.79]
Luxembourg	Female	0[0-0]	0[0-0]	NaN	0.06[0.04-0.07]	0.04[0.03-0.06]	-1.32[-1.52to-1.12]
Madagascar	Female	0.06[0.02-0.12]	0.12[0.04-0.25]	100	0.28[0.11-0.52]	0.15[0.07-0.3]	-0.35[-0.45to-0.25]
Malawi	Female	0.06[0.02-0.12]	0.1[0.04-0.2]	66.67	0.36[0.16-0.69]	0.22[0.09-0.41]	-0.58[-0.68to-0.48]
Malaysia	Female	0.04[0.01-0.07]	0.14[0.03-0.47]	250	0.09[0.03-0.16]	0.1[0.03-0.29]	1.12[0.7to1.54]
Maldives	Female	0[0-0]	0[0-0]	NaN	0.04[0.01-0.09]	0.03[0.01-0.05]	-3.87[-4.24to-3.5]
Mali	Female	0.04[0.01-0.07]	0.04[0.02-0.09]	0	0.22[0.07-0.37]	0.07[0.04-0.12]	-2.61[-2.78to-2.43]
Malta	Female	0[0-0]	0.01[0-0.01]	Inf	0.16[0.11-0.2]	0.1[0.08-0.14]	-1.69[-2.01to-1.37]
Marshall Islands	Female	0[0-0]	0[0-0]	NaN	0.25[0.07-0.53]	0.18[0.07-0.34]	-0.87[-1.12to-0.62]
Mauritania	Female	0.01[0-0.02]	0.01[0-0.02]	0	0.26[0.08-0.48]	0.07[0.03-0.12]	-3.43[-3.61to-3.26]
Mauritius	Female	0[0-0]	0[0-0]	NaN	0.02[0.01-0.02]	0.02[0.01-0.03]	-0.72[-1.86to0.44]
Mexico	Female	0.63[0.49-0.8]	1.86[1.29-3.22]	195.24	0.27[0.21-0.35]	0.31[0.25-0.47]	0.41[0.09to0.74]
Micronesia (Federated States of)	Female	0 [0-0]	0 [0-0]	NaN	0.33 [0.1-0.61]	0.19 [0.08-0.34]	-1.46 [-1.65 to -1.28]
Mongolia	Female	0.01[0-0.03]	0.01[0-0.02]	0	0.21[0.06-0.5]	0.04[0.02-0.09]	-6.82[-7.75to-5.87]
Montenegro	Female	0[0-0]	0[0-0]	NaN	0[0-0.01]	0[0-0.01]	0.4[0.18to0.62]
Morocco	Female	0.01[0-0.03]	0.04[0.01-0.07]	300	0.01[0-0.04]	0.02[0.01-0.04]	3.4[2.92to3.88]
Mozambique	Female	0.1[0.04-0.19]	0.19[0.07-0.4]	90	0.39[0.15-0.75]	0.27[0.11-0.52]	-0.04[-0.15to0.07]
Myanmar	Female	0.51[0.05-0.95]	0.61[0.07-1.44]	19.61	0.4[0.05-0.76]	0.39[0.05-0.7]	-1.94[-2.29to-1.58]
Namibia	Female	0[0-0]	0[0-0]	NaN	0.04[0.01-0.09]	0.05[0.02-0.08]	-2.05[-2.47to-1.62]

Nepal	Female	0.09[0.04-0.17]	0.17[0.07-0.35]	88.89	0.2[0.09-0.41]	0.16[0.08-0.31]	-0.77[-1.23to-0.3]
Netherlands	Female	0.41[0.31-0.61]	0.32[0.21-0.59]	−21.95	0.31[0.24-0.46]	0.14[0.11-0.22]	-3.15[-3.57to-2.73]
NewZealand	Female	0.07[0.05-0.1]	0.08[0.07-0.12]	14.29	0.3[0.24-0.45]	0.18[0.15-0.25]	-0.88[-1.44to-0.32]
Nicaragua	Female	0[0-0]	0.01[0-0.03]	Inf	0.02[0.01-0.02]	0.07[0.02-0.1]	5.11[4.51to5.72]
Niger	Female	0.02[0.01-0.05]	0.03[0.02-0.06]	50	0.19[0.07-0.37]	0.06[0.03-0.1]	-3.06[-3.29to-2.83]
Nigeria	Female	0.24[0.08-0.42]	0.24[0.12-0.42]	0	0.12[0.04-0.21]	0.05[0.03-0.07]	-3.07[-3.28to-2.86]
North Mac edonia	Female	0[0-0]	0 [0-0]	NaN	0.01[0.01-0.01]	0[0-0.01]	-2.63[-3.59to-1.65]
Northern Mariana Islands	Female	0 [0-0]	0 [0-0]	NaN	0.17[0.08-0.3]	0.04[0.03-0.07]	-3.59 [-4.84 to -2.32]
Norway	Female	0.1[0.08-0.16]	0.08[0.05-0.15]	−20.00	0.22[0.18-0.33]	0.12[0.09-0.21]	-1.69[-2.13to-1.26]
Oman	Female	0[0-0]	0[0-0]	NaN	0.02[0.01-0.06]	0.03[0.01-0.07]	3.72[3.08to4.37]
Pakistan	Female	0.58[0.26-0.95]	1.1[0.48-1.93]	89.66	0.22[0.1-0.38]	0.23[0.12-0.39]	-0.34[-0.59to-0.1]
Palestine	Female	0[0-0.01]	0[0-0.01]	NaN	0.04[0.02-0.1]	0.03[0.02-0.07]	-1.99[-2.84to-1.13]
Panama	Female	0[0-0]	0[0-0.01]	NaN	0.01[0.01-0.02]	0.02[0.01-0.03]	1.03[-0.4to2.47]
Papua New Guinea	Female	0.02[0-0.05]	0.04[0-0.09]	100	0.23[0.01-0.48]	0.13[0.04-0.23]	-1.23[-1.3to-1.16]
Paraguay	Female	0[0-0]	0.02[0-0.04]	Inf	0.02[0.01-0.04]	0.08[0.02-0.14]	6.32[5.34to7.31]
Peru	Female	0.02[0.01-0.04]	0.06[0.02-0.11]	200	0.04[0.02-0.06]	0.04[0.02-0.06]	-0.32[-0.85to0.23]
Philippines	Female	0.78[0.41-1.04]	1.62[1.03-2.35]	107.69	0.55[0.29-0.76]	0.65[0.48-1.12]	-0.96[-1.07to-0.85]
Poland	Female	0.86[0.72-1.29]	0.13[0.09-0.22]	−84.88	0.34[0.28-0.51]	0.04[0.03-0.05]	-9.11[-10.44to-7.76]
Portugal	Female	0.04[0.01-0.06]	0.15[0.08-0.39]	275	0.05[0.02-0.07]	0.08[0.05-0.17]	3.08[2.24to3.93]
PuertoRico	Female	0[0-0]	0.01[0.01-0.02]	Inf	0.01[0.01-0.02]	0.03[0.02-0.04]	1.48[-0.02to3.01]
Qatar	Female	0[0-0]	0[0-0]	NaN	0.03[0.01-0.06]	0.02[0.01-0.03]	2.89[2.11to3.67]
Republic of Korea	Female	0.07[0.05-0.17]	0.4[0.16-0.82]	471.43	0.06[0.04-0.14]	0.08[0.04-0.13]	0.88[0.3to1.46]
Republic of Moldova	Female	0.07[0.05-0.1]	0.09[0.05-0.12]	28.57	0.27 [0.19-0.37]	0.28 [0.18-0.36]	0.62 [-0.14 to 1.38]
Romania	Female	0.02[0.01-0.02]	0.01[0.01-0.02]	−50.00	0.01[0.01-0.02]	0.01[0-0.01]	-2.05[-2.97to-1.12]
Russian Federation	Female	7.39 [6.23-9.52]	9.03 [6.83-12.39]	22.19	0.63 [0.53-0.82]	0.62 [0.5-0.78]	-0.26 [-0.87 to 0.35]
Rwanda	Female	0.05 [0-0.1]	0.06 [0-0.16]	20	0.34 [0-0.73]	0.18 [0.01-0.47]	-1.38 [-1.61 to -1.16]
Saint Lucia	Female	0 [0-0]	0 [0-0]	NaN	0.19 [0.15-0.24]	0.18 [0.13-0.23]	1.19 [0.9 to 1.49]
Saint Vincent and the Grenadines	Female	0 [0-0]	0 [0-0]	NaN	0.01 [0.01-0.01]	0.17 [0.1-0.23]	1.7 [1.43 to 1.96]
Samoa	Female	0 [0-0]	0 [0-0]	NaN	0.25 [0.06-0.46]	0.12 [0.05-0.19]	-2.07 [-2.26 to -1.88]
Sao Tome and Principe	Female	0 [0-0]	0 [0-0]	NaN	0.16 [0.06-0.28]	0.08 [0.03-0.18]	-1.44 [-1.73 to -1.16]

Saudi Arabia	Female	0 [0-0]	0 [0-0.01]	NaN	0.01 [0-0.02]	0.01 [0-0.03]	1.1 [0.82 to 1.39]
Senegal	Female	0.03 [0.01-0.05]	0.03 [0.01-0.05]	0	0.18 [0.06-0.34]	0.06 [0.04-0.1]	-3.19 [-3.4 to -2.97]
Serbia	Female	0.03 [0.02-0.05]	0.04 [0.02-0.06]	33.33	0.05 [0.03-0.09]	0.05 [0.03-0.07]	-0.2 [-0.5 to 0.11]
Seychelles	Female	0 [0-0]	0 [0-0]	NaN	0.06 [0.01-0.11]	0.27 [0.03-0.5]	3.3 [2.77 to 3.83]
Sierra Leone	Female	0.01 [0-0.02]	0.01 [0.01-0.03]	0	0.14 [0.05-0.26]	0.06 [0.03-0.09]	-1.95 [-2.08 to -1.83]
Singapore	Female	0.01 [0.01-0.02]	0.01 [0-0.01]	0	0.13 [0.1-0.21]	0.02 [0.02-0.03]	-6.13 [-6.79 to -5.47]
Slovakia	Female	0.08 [0.04-0.12]	0.04 [0.03-0.09]	−50.00	0.24 [0.12-0.36]	0.07 [0.05-0.12]	-4.05 [-4.44 to -3.67]
Slovenia	Female	0.03 [0.01-0.06]	0.01 [0.01-0.03]	−66.67	0.16 [0.08-0.37]	0.03 [0.02-0.07]	-5.54 [-6.16 to -4.91]
Solomon Islands	Female	0 [0-0]	0 [0-0.01]	NaN	0.32 [0.08-0.62]	0.2 [0.09-0.34]	-0.82 [-1.02 to -0.62]
Somalia	Female	0.06 [0.02-0.13]	0.15 [0.05-0.32]	150	0.46 [0.16-0.94]	0.32 [0.11-0.66]	0.35 [0.26 to 0.44]
South Africa	Female	0.07 [0.04-0.1]	0.09 [0.07-0.14]	28.57	0.05 [0.03-0.07]	0.05 [0.04-0.07]	-0.8 [-1.42 to -0.18]
South Sudan	Female	0.03 [0.01-0.05]	0.04 [0.02-0.08]	33.33	0.27 [0.1-0.54]	0.15 [0.06-0.28]	-0.17 [-0.21 to -0.13]
Spain	Female	0.3 [0.17-0.38]	0.47 [0.31-0.92]	56.67	0.09 [0.05-0.12]	0.06 [0.05-0.1]	-0.91 [-1.24 to -0.59]
Sri Lanka	Female	0 [0-0]	0.02 [0-0.04]	Inf	0 [0-0.01]	0.02 [0.01-0.04]	6.16 [4.09 to 8.28]
Sudan	Female	0.01 [0-0.02]	0.02 [0.01-0.06]	100	0.02 [0-0.05]	0.02 [0.01-0.05]	2.74 [2.34 to 3.14]
Suriname	Female	0 [0-0]	0 [0-0]	NaN	0.05 [0.02-0.09]	0.3 [0.16-0.47]	1.92 [1.55 to 2.29]
Sweden	Female	0.14 [0.11-0.19]	0.12 [0.08-0.17]	−14.29	0.15 [0.11-0.2]	0.12 [0.09-0.15]	-1 [-1.72 to -0.28]
Switzerland	Female	0.07 [0.04-0.09]	0.05 [0.03-0.08]	−28.57	0.1 [0.06-0.12]	0.04 [0.03-0.06]	-3.13 [-3.53 to -2.73]
Syrian Arab Republic	Female	0 [0-0.01]	0 [0-0.01]	NaN	0.01[0.01-0.03]	0.01[0-0.02]	-2.8 [-3.43 to -2.17]
Tajikistan	Female	0.02[0-0.03]	0.04[0.01-0.07]	100	0.1[0.03-0.19]	0.29[0.15-0.48]	2.26[2.04to2.48]
Thailand	Female	0.65[0.15-1.39]	2.13[0.31-5.25]	227.69	0.43[0.09-0.94]	0.47[0.13-0.88]	-0.55[-0.92to-0.17]
Timor-Leste	Female	0[0-0.01]	0.01[0-0.02]	Inf	0.32[0.03-0.69]	0.38[0.05-0.72]	-1.06[-1.28to-0.84]
Togo	Female	0.01[0-0.02]	0.01[0.01-0.03]	0	0.16[0.05-0.31]	0.07[0.04-0.1]	-2.84[-3.06to-2.61]
Tonga	Female	0[0-0]	0[0-0]	NaN	0.06[0.03-0.09]	0.12[0.05-0.21]	-1.52[-1.76to-1.27]
Trinidad and Tobago	Female	0.01[0.01-0.01]	0.03[0.02-0.04]	200	0.18[0.14-0.28]	0.53[0.37-0.83]	3.53 [2.25 to 4.83]
Tunisia	Female	0[0-0.01]	0.01[0-0.02]	Inf	0.01[0-0.03]	0.01[0-0.02]	1.99[1.64to2.35]
Turkey	Female	0.12[0.06-0.25]	0.24[0.14-0.36]	100	0.07[0.03-0.14]	0.06[0.04-0.08]	0.02[-0.64to0.67]
Turkmenistan	Female	0.01[0-0.01]	0.04[0.02-0.06]	300	0.05[0.02-0.07]	0.18[0.13-0.24]	5.47[4.97to5.96]
Uganda	Female	0.06[0.01-0.13]	0.15[0.03-0.32]	150	0.23[0.03-0.47]	0.17[0.04-0.35]	0[-0.17to0.18]
Ukraine	Female	2.14[1.41-2.87]	1.61[0.8-2.29]	−24.77	0.46[0.31-0.62]	0.42[0.28-0.54]	-1.67[-2.02to-1.33]
United Arab Emirates	Female	0.02[0-0.06]	0.03[0.01-0.07]	NaN	0.02 [0-0.06]	0.03 [0.01-0.07]	3.76 [2.22 to 5.32]

United Kingdom	Female	0.88[0.38-1.08]	1.43[1.12-2.28]	62.5	0.16[0.07-0.19]	0.18[0.15-0.25]	0.9[0.66to1.14]
United Republic of Tanzania	Female	0.12[0.05-0.24]	0.29[0.11-0.63]	141.67	0.28[0.12-0.52]	0.17[0.06-0.36]	-0.07 [-0.2 to 0.06
United States of America	Female	1.88[1.42-2.43]	3.29[2.57-4.7]	75	0.09 [0.07-0.12	0.09 [0.08-0.12]	1.02 [0.63 to 1.41
United States Virgin Islands	Female	0 [0-0]	0 [0-0.01	NaN	0.47 [0.3-0.74]	0.49 [0.33-0.7]	-0.12 [-0.22 to -0.02]
Uruguay	Female	0.01[0-0.01]	0.03[0.02-0.04]	200	0.04[0.02-0.05]	0.07[0.05-0.09]	2.45[1.98to2.93]
Uzbekistan	Female	0[0-0]	0.01[0-0.01]	Inf	0[0-0.01]	0.01[0.01-0.02]	3.92[3.48to4.35]
Vanuatu	Female	0[0-0]	0[0-0]	NaN	0.21[0.06-0.41]	0.16[0.07-0.32]	-0.93[-1.15to-0.71]
Venezuela (Bolivarian Republic of)	Female	0.07[0.06-0.1]	0.29[0.18-0.42]	314.29	0.12[0.09-0.17]	0.21[0.14-0.29]	0.62[-0.27to1.51]
Viet Nam	Female	0.41[0.03-0.77]	0.68[0.04-1.14]	65.85	0.18[0.01-0.34]	0.13[0.01-0.22]	-1.11[-1.65to-0.57]
Yemen	Female	0[0-0.01]	0.02 [0.01-0.07]	Inf	0.01 [0-0.05]	0.02[0.01-0.07]	3 [2.61 to 3.39]
Zambia	Female	0.05[0.02-0.11]	0.1 [0.04-0.22]	100	0.42 [0.2-0.81]	0.22[0.09-0.46]	-0.76 [-0.88 to -0.65]
Zimbabwe	Female	0[0-0.01]	0.01 [0-0.01]	Inf	0.02 [0-0.04]	0.06[0.02-0.09]	0.96 [0.61 to 1.32]

Supplementary Table S6. The DALY and age-standardized DALY rate of Urolithiasis in 1990 and 2019, and its temporal trends from 1990 to 2019.

Supplementary Table S6. The DALYs cases and age-standardized DALYs rate of Urolithiasis in 1990 and 2019, and its temporal trends from 1990 to 2019.

Nation	Sex	DALYs cases No.(95% UI)		change absolute number(%)	DALYs per 100,000 No.(95% UI)		1990-2019 EAPC No. (95% CI)
		1990	2019		1990	2019	
Afghanistan	Both	3.3 [2.15-5.02]	10.95 [7.25-15.56]	231.82	3.97 [2.57-6.06]	4.67 [3.06-6.94]	0.76 [0.67 to 0.85]
Albania	Both	1.84 [1.03-2.75]	1.28 [0.83-1.83]	-30.43	6.54 [3.86-9.14]	3.82 [2.47-5.43]	-1.48 [-1.95 to -1.02]
Algeria	Both	6.09 [3.89-8.92]	15.51 [10.01-23.09]	154.68	3.49 [2.22-5.12]	3.69 [2.39-5.44]	0.26 [0.21 to 0.3]
American Samoa	Both	0.02 [0.01-0.03]	0.03 [0.02-0.03]	50	7.83 [4.41-11.16]	4.93 [3.51-6.6]	-2.32 [-3.05 to -1.58]
Andorra	Both	0.05 [0.03-0.06]	0.08 [0.05-0.11]	60	7.74 [4.67-10.7]	6.36 [4.18-8.73]	-0.59 [-0.75 to -0.43]
Angola	Both	3.8 [2.16-6.62]	6.99 [4.64-10.66]	83.95	6.33 [3.59-10.92]	4.24 [2.79-6.6]	-1.65 [-1.79 to -1.51]
Antigua and Barbuda	Both	0.02 [0.02-0.03]	0.06 [0.05-0.08]	200	4.47 [3.27-5.94]	6.04 [4.61-7.76]	1.14 [1.04 to 1.24]
Argentina	Both	15.5 [10.23-22.4]	25.64 [16.9-37.11]	65.42	4.85 [3.19-7.02]	5.22 [3.42-7.58]	0.31 [0.26 to 0.36]
Armenia	Both	5.53 [4.25-7.21]	13.31 [8.61-24.74]	140.69	19.88 [15.29-25.86]	33.33 [21.71-61.27]	2.46 [2.07 to 2.85]
Australia	Both	13.89 [11.07-17.35]	17.08 [12.79-22.22]	22.97	7.29 [5.77-9.08]	5.01 [3.64-6.62]	-1.18 [-1.45 to -0.9]
Austria	Both	15.96 [12.87-19.87]	10.53 [7.02-15.8]	-34.02	14.9 [11.86-18.7]	7.92 [5.03-12.18]	-1.18 [-1.53 to -0.82]
Azerbaijan	Both	3.13 [2.01-4.58]	6.28 [4.09-8.91]	100.64	4.99 [3.28-7.29]	6.15 [4-8.69]	0.89 [0.7 to 1.09]
Bahamas	Both	0.1 [0.08-0.13]	0.27 [0.2-0.35]	170	5.01 [3.83-6.45]	6.22 [4.7-8.11]	0.84 [0.71 to 0.98]
Bahrain	Both	0.16 [0.1-0.23]	0.74 [0.45-1.13]	362.5	3.72 [2.39-5.37]	4.2 [2.65-6.3]	0.5 [0.3 to 0.7]
Bangladesh	Both	50.92 [33.28-71.25]	82.52 [55.74-116.66]	62.06	6.92 [4.64-9.68]	5.49 [3.73-7.72]	-0.7 [-0.8 to -0.59]
Barbados	Both	0.16 [0.12-0.21]	0.34 [0.25-0.43]	112.5	6.25 [4.85-8.35]	7.79 [5.75-9.91]	0.89 [0.74 to 1.03]
Belarus	Both	30.38 [24.08-38.07]	28.39 [20.49-37.35]	-6.55	24.26 [19.17-30.5]	20.03 [14.5-26.28]	-1.01 [-1.14 to -0.89]
Belgium	Both	6.76 [4.82-9.26]	10.56 [7.41-14.82]	56.21	5.4 [3.82-7.46]	6.03 [4.14-8.27]	1.77 [1.04 to 2.5]
Belize	Both	0.06 [0.04-0.08]	0.29 [0.23-0.37]	383.33	5.14 [3.82-6.9]	8.56 [6.71-10.6]	1.75 [1.31 to 2.2]
Benin	Both	0.99 [0.66-1.41]	2.24 [1.58-2.98]	126.26	3.77 [2.52-5.58]	3.15 [2.25-4.25]	-0.67 [-0.72 to -0.62]
Bermuda	Both	0.03 [0.02-0.04]	0.05 [0.04-0.07]	66.67	4.57 [3.41-5.97]	5 [3.72-6.59]	0.33 [0.19 to 0.48]
Bhutan	Both	0.32 [0.2-0.49]	0.46 [0.31-0.7]	43.75	8.71 [5.27-15.25]	6.91 [4.63-10.56]	-0.86 [-0.95 to -0.76]

Bolivia (Plurinational State of)	Both	3.76 [2.4-5.27]	8 [5.46-10.88]	112.77	8.93 [5.55-12.76]	7.8 [5.25-10.64]	-0.36 [-0.46 to -0.26]
Bosnia and Herzegovina	Both	2.51 [1.74-3.37]	1.81 [1.21-2.54]	-27.89	5.57 [3.94-7.45]	4.03 [2.67-5.6]	-1.44 [-1.75 to -1.12]
Botswana	Both	0.28 [0.19-0.38]	0.7 [0.46-0.97]	150	3.26 [2.21-4.47]	3.45 [2.29-4.79]	0 [-0.2 to 0.2]
Brazil	Both	63.6 [50.35-79.42]	208.86 [166.58-296.44]	228.4	5.48 [4.4-6.81]	8.65 [6.91-12.33]	2.08 [1.94 to 2.22]
Brunei Darussalam	Both	0.15 [0.1-0.2]	0.3 [0.21-0.4]	100	10.8 [7.57-15.13]	8.48 [6.47-11]	-0.56 [-0.79 to -0.32]
Bulgaria	Both	28.55 [21.84-46.98]	4.74 [3.26-6.53]	-83.4	23.82 [18.25-38.74]	4.64 [3.27-6.4]	-5.78 [-6.86 to -4.7]
Burkina Faso	Both	2.21 [1.48-3.19]	4.99 [3.31-7.18]	125.79	4.1 [2.69-6.06]	3.95 [2.61-5.76]	-0.22 [-0.3 to -0.14]
Burundi	Both	1.81 [0.86-3.56]	2.75 [1.54-4.49]	51.93	5.66 [2.59-10.91]	4.34 [2.24-7.26]	-1.25 [-1.39 to -1.11]
Cabo Verde	Both	0.05 [0.03-0.07]	0.12 [0.08-0.16]	140	2.12 [1.46-2.98]	2.28 [1.5-3.22]	0.14 [0.06 to 0.22]
Cambodia	Both	10.66 [2.84-16.6]	18.26 [6.55-27.06]	71.29	18.24 [4.95-29.47]	13.79 [4.77-20.66]	-1.19 [-1.3 to -1.07]
Cameroon	Both	2.42 [1.5-3.71]	5.72 [3.88-8.15]	136.36	4.24 [2.51-6.51]	3.34 [2.26-4.7]	-1.01 [-1.1 to -0.91]
Canada	Both	13.56 [10.25-17.8]	26.9 [20.81-34.18]	98.38	4.33 [3.26-5.7]	4.81 [3.68-6.16]	0.76 [0.58 to 0.94]
Central African Republic	Both	1.13 [0.64-2.07]	2.24 [1.27-4.08]	98.23	6.92 [3.8-12.01]	7.11 [3.88-12.64]	0.15 [0 to 0.3]
Chad	Both	1.37 [0.89-2.02]	2.77 [1.93-3.84]	102.19	3.95 [2.54-5.96]	3.36 [2.36-4.64]	-0.66 [-0.73 to -0.6]
Chile	Both	7.53 [5.38-10.18]	13.35 [9.49-18.1]	77.29	6.36 [4.56-8.53]	6.11 [4.31-8.37]	-1.09 [-1.35 to -0.82]
China	Both	1590.42 [940.18-1966.34]	1022.09 [789.78-1293.12]	-35.73	16.92 [9.96-20.73]	5.27 [4.09-6.66]	-4.57 [-4.85 to -4.3]
Colombia	Both	8.55 [6.45-11.15]	20.6 [15.41-27.47]	140.94	3.53 [2.68-4.56]	3.97 [2.97-5.28]	0.9 [0.53 to 1.28]
Comoros	Both	0.14 [0.06-0.25]	0.27 [0.14-0.49]	92.86	5.02 [2.4-9.11]	4.78 [2.52-8.55]	-0.4 [-0.56 to -0.25]
Congo	Both	0.81 [0.51-1.43]	1.6 [1.06-2.41]	97.53	5.83 [3.62-10.16]	4.47 [2.92-6.73]	-1.22 [-1.39 to -1.04]
Costa Rica	Both	0.52 [0.33-0.74]	1.36 [0.94-1.89]	161.54	2.24 [1.41-3.22]	2.62 [1.81-3.62]	0.83 [0.69 to 0.97]
Cote d'Ivoire	Both	2.26 [1.49-3.23]	5.06 [3.47-6.96]	123.89	3.66 [2.4-5.39]	3.2 [2.22-4.41]	-0.52 [-0.57 to -0.46]
Croatia	Both	3.02 [2.28-3.93]	3.66 [2.59-5.16]	21.19	5.13 [3.86-6.68]	5.45 [3.85-7.46]	0.57 [0.38 to 0.75]
Cuba	Both	7.36 [5.92-9.24]	15.92 [12.05-21.2]	116.3	6.99 [5.64-8.81]	9.36 [7.09-12.33]	1.3 [1.12 to 1.49]
Cyprus	Both	0.43 [0.29-0.62]	0.74 [0.5-1.05]	72.09	5.34 [3.58-7.58]	4.26 [2.86-6.06]	-1.89 [-2.42 to -1.35]
Czechia	Both	29.44 [22.82-50.16]	8.54 [6.43-11.26]	-70.99	21.97 [17.1-36.79]	5.39 [3.97-7.13]	-3.43 [-4.19 to -2.67]
Democratic People's Republic of Korea	Both	20.59 [10.29-29.47]	28.52 [15.82-40.82]	38.51	10.89 [5.56-15.28]	8.82 [4.88-12.65]	-0.82 [-0.99 to -0.66]
Democratic Republic of the Congo	Both	10.05 [6.53-15.78]	21.16 [13.74-31.46]	110.55	4.46 [2.78-6.78]	4.17 [2.57-6.4]	-0.18 [-0.28 to -0.08]

Denmark	Both	4.44 [3.5-5.73]	5.85 [4.59-7.49]	31.76	6.29 [4.85-8.2]	6.16 [4.71-7.94]	0.09 [-0.07 to 0.25]
Djibouti	Both	0.11 [0.06-0.17]	0.38 [0.22-0.65]	245.45	4.72 [2.73-7.55]	4.88 [2.78-8.67]	0.02 [-0.11 to 0.16]
Dominica	Both	0.02 [0.02-0.03]	0.04 [0.03-0.05]	100	3.58 [2.47-5.01]	4.61 [3.34-6.18]	0.96 [0.9 to 1.02]
Dominican Republic	Both	1.9 [1.3-2.6]	3.8 [2.58-5.33]	100	3.72 [2.6-5.11]	3.67 [2.49-5.13]	0.11 [-0.07 to 0.29]
Ecuador	Both	4.29 [3.02-5.78]	12.27 [8.9-16.07]	186.01	5.97 [4.28-8.03]	7.39 [5.36-9.68]	1.39 [1.1 to 1.69]
Egypt	Both	14.5 [9.42-21.12]	32 [20.18-47.28]	120.69	3.4 [2.21-4.94]	3.63 [2.32-5.26]	0.28 [0.24 to 0.31]
El Salvador	Both	0.98 [0.66-1.39]	1.54 [1.05-2.14]	57.14	2.72 [1.83-3.86]	2.57 [1.75-3.56]	-0.22 [-0.34 to -0.1]
Equatorial Guinea	Both	0.16 [0.09-0.28]	0.28 [0.18-0.42]	75	6.02 [3.3-10.65]	3.74 [2.41-5.7]	-1.71 [-2.1 to -1.33]
Eritrea	Both	0.9 [0.51-1.64]	2.04 [1.14-3.54]	126.67	5.71 [3.07-10.09]	5.57 [3.13-9.54]	-0.02 [-0.12 to 0.08]
Estonia	Both	4.91 [3.56-7.36]	2.75 [1.99-3.66]	-43.99	24.99 [18.28-36.92]	13.79 [9.78-18.63]	-2.95 [-3.36 to -2.54]
Eswatini	Both	0.18 [0.12-0.25]	0.33 [0.22-0.46]	83.33	3.75 [2.56-5.11]	3.9 [2.61-5.33]	0.36 [-0.15 to 0.88]
Ethiopia	Both	28.82 [18.16-50.43]	27.38 [16.9-39.49]	-5	9.94 [6.32-16.29]	4.81 [2.97-7.06]	-2.84 [-2.97 to -2.7]
Fiji	Both	0.18 [0.11-0.25]	0.35 [0.24-0.49]	94.44	3.22 [2.1-4.63]	3.97 [2.78-5.46]	1.09 [0.88 to 1.3]
Finland	Both	2.76 [1.83-3.95]	3.34 [2.37-4.57]	21.01	4.31 [2.83-6.23]	4.06 [2.78-5.71]	-0.71 [-1.31 to -0.11]
France	Both	38.74 [28.84-50.83]	45.41 [33.28-60.46]	17.22	5.48 [4-7.33]	4.8 [3.32-6.66]	-0.43 [-0.48 to -0.37]
Gabon	Both	0.36 [0.19-0.67]	0.61 [0.37-1]	69.44	5.58 [2.8-10.22]	4.78 [2.78-7.79]	-0.56 [-0.71 to -0.41]
Gambia	Both	0.17 [0.12-0.25]	0.44 [0.3-0.62]	158.82	3.49 [2.3-5.26]	3.4 [2.31-4.71]	-0.17 [-0.26 to -0.08]
Germany	Both	58.1 [44.02-75.4]	62.82 [45.21-84.25]	8.12	5.39 [4.05-7.07]	4.7 [3.27-6.48]	1.22 [0.75 to 1.7]
Ghana	Both	6.52 [2.44-11.63]	15.43 [6.03-23.89]	136.66	8.61 [2.84-16.92]	8.35 [2.85-13.29]	0.32 [0.16 to 0.48]
Greece	Both	5.05 [3.25-7.36]	5.85 [3.74-8.38]	15.84	3.98 [2.55-5.78]	3.99 [2.55-5.78]	0.09 [0.03 to 0.14]
Greenland	Both	0.02 [0.01-0.02]	0.02 [0.01-0.03]	0	3.32 [2.18-4.56]	3.08 [2.01-4.43]	-0.3 [-0.41 to -0.2]
Grenada	Both	0.05 [0.04-0.06]	0.15 [0.11-0.19]	200	7.58 [5.95-9.7]	12.71 [9.73-16]	2.19 [1.96 to 2.41]
Guam	Both	0.07 [0.03-0.1]	0.06 [0.04-0.09]	-14.29	7.31 [3.26-10.2]	3.43 [2.22-4.89]	-3.21 [-3.93 to -2.49]
Guatemala	Both	3.58 [2.65-4.66]	6.69 [5.03-8.86]	86.87	6.52 [4.9-8.23]	4.97 [3.75-6.57]	-1.07 [-1.37 to -0.77]
Guinea	Both	1.55 [0.96-2.4]	2.53 [1.68-3.63]	63.23	3.88 [2.42-6.19]	3.44 [2.31-4.94]	-0.37 [-0.44 to -0.3]
Guinea-Bissau	Both	0.29 [0.17-0.47]	0.41 [0.28-0.59]	41.38	5.37 [3.15-8.37]	3.93 [2.6-5.5]	-1.17 [-1.23 to -1.12]
Guyana	Both	0.39 [0.3-0.52]	0.84 [0.62-1.1]	115.38	7.88 [6.13-10.64]	11.6 [8.57-15.11]	1.41 [1.07 to 1.75]
Haiti	Both	5.38 [2.72-9.22]	10.31 [5.75-15.9]	91.64	11.62 [6.04-18.77]	11.3 [6.17-19.01]	0.28 [0.14 to 0.41]
Honduras	Both	2.4 [1.1-3.48]	4.84 [2.84-7.17]	101.67	8.63 [3.92-12.59]	6.93 [3.98-10.48]	-0.68 [-0.78 to -0.58]

Hungary	Both	39.61 [34.14-51.77]	15.04 [11.1-19.33]	-62.03	28.1 [24.34-36.87]	9.07 [6.76-11.68]	-3.55 [-4.18 to -2.92]
Iceland	Both	0.17 [0.13-0.21]	0.24 [0.18-0.31]	41.18	6.08 [4.72-7.77]	5.14 [3.68-6.85]	-0.88 [-1.09 to -0.66]
India	Both	711.04 [445.5-968.23]	1112.46 [796.52-1486.64]	56.46	12.39 [7.51-17.97]	8.58 [6.17-11.43]	-1.37 [-1.57 to -1.16]
Indonesia	Both	229.45 [81.92-337.65]	323.06 [103.97-474.17]	40.8	18.65 [6.5-27.9]	13.35 [4.15-19.56]	-1.27 [-1.41 to -1.13]
Iran (Islamic Republic of)	Both	15.83 [10.57-22.08]	39.55 [26.49-55.2]	149.84	3.94 [2.61-5.51]	4.38 [2.97-6.03]	0.44 [0.26 to 0.62]
Iraq	Both	7.1 [4.73-11.65]	14 [9.53-19.4]	97.18	5.88 [4.01-8.73]	4.07 [2.82-5.62]	-1.73 [-1.95 to -1.51]
Ireland	Both	2.12 [1.56-2.77]	3.12 [2.19-4.24]	47.17	5.69 [4.13-7.49]	4.91 [3.41-6.65]	-0.49 [-0.67 to -0.32]
Israel	Both	2.17 [1.48-3.06]	6.05 [4.44-7.91]	178.8	4.7 [3.18-6.6]	5.78 [4.15-7.7]	0.4 [0.08 to 0.72]
Italy	Both	59.4 [46.4-74.75]	49.72 [36.4-66.76]	-16.3	7.84 [6.06-9.94]	5.18 [3.64-7]	-1.41 [-1.66 to -1.15]
Jamaica	Both	0.62 [0.42-0.86]	1.63 [1.2-2.2]	162.9	3.34 [2.24-4.75]	5.49 [4.02-7.43]	2.27 [2.04 to 2.5]
Japan	Both	87.03 [61-118.99]	145.68 [113.91-182.75]	67.39	5.51 [3.85-7.53]	5.97 [4.46-7.79]	0.39 [0.3 to 0.48]
Jordan	Both	0.91 [0.61-1.29]	5.3 [3.14-8.57]	482.42	3.75 [2.5-5.27]	5.19 [3.12-8.36]	1.54 [1.25 to 1.83]
Kazakhstan	Both	27.6 [18.74-41.79]	39.72 [31.29-49.52]	43.91	20.74 [14.08-31.55]	22.57 [17.82-28.08]	-0.34 [-0.65 to -0.02]
Kenya	Both	4.9 [3.34-7.2]	14.48 [9.43-22.78]	195.51	4.34 [2.88-6.43]	5 [3.18-8.12]	0.79 [0.67 to 0.9]
Kuwait	Both	0.54 [0.34-0.81]	1.9 [1.19-2.92]	251.85	3.6 [2.29-5.27]	3.7 [2.42-5.4]	0.22 [0.18 to 0.25]
Kyrgyzstan	Both	5.25 [4.05-6.99]	5.44 [4.08-7.18]	3.62	14.1 [10.96-18.42]	9.15 [6.89-11.96]	-1.37 [-1.82 to -0.93]
Lao People's Democratic Republic)	Both	5.65 [1.31-9.45]	6.53 [2.4-10.26]	15.58	21.44 [5.12-38.43]	12.56 [4.37-20.15]	-2.2 [-2.39 to -2]
Latvia	Both	10.15 [7.74-13.81]	6.92 [5.47-8.59]	-31.82	29.24 [22.33-39.45]	20.47 [15.73-25.88]	-2.13 [-2.46 to -1.8]
Lebanon	Both	0.9 [0.57-1.31]	1.9 [1.19-2.75]	111.11	3.32 [2.11-4.83]	3.54 [2.24-5.12]	0.27 [0.23 to 0.31]
Lesotho	Both	0.43 [0.29-0.64]	0.78 [0.51-1.09]	81.4	3.35 [2.21-5.04]	4.56 [2.93-6.37]	1.64 [1.36 to 1.91]
Liberia	Both	0.53 [0.33-0.77]	1.01 [0.69-1.4]	90.57	4.09 [2.56-5.92]	3.28 [2.29-4.53]	-0.88 [-1.05 to -0.71]
Libya	Both	0.98 [0.62-1.46]	2.74 [1.75-4.02]	179.59	3.48 [2.18-5.15]	3.76 [2.46-5.32]	0.35 [0.28 to 0.41]
Lithuania	Both	13.24 [10.84-16.39]	7.8 [6.07-10.03]	-41.09	30.39 [24.78-37.64]	17.2 [12.95-22.47]	-2.87 [-3.19 to -2.55]
Luxembourg	Both	0.27 [0.2-0.37]	0.42 [0.29-0.58]	55.56	5.65 [4.05-7.62]	5.01 [3.45-7.01]	-0.32 [-0.87 to 0.23]
Madagascar	Both	3.63 [1.97-6.66]	6.95 [4.01-12.04]	91.46	5.02 [2.8-8.51]	4.46 [2.57-7.56]	-0.41 [-0.49 to -0.33]
Malawi	Both	3.07 [1.69-5.93]	4.56 [2.78-7.56]	48.53	5.8 [3.24-10.19]	4.83 [2.84-8.01]	-0.73 [-0.81 to -0.64]
Malaysia	Both	6.19 [4.15-8.6]	16.86 [10.6-26.43]	172.37	5.41 [3.54-7.43]	5.7 [3.54-9.28]	0.25 [0.16 to 0.34]
Maldives	Both	0.06 [0.04-0.08]	0.22 [0.14-0.34]	266.67	5.21 [3.38-7.39]	4.69 [3.1-6.74]	-0.49 [-0.69 to -0.29]

Mali	Both	2.49 [1.49-3.88]	4.25 [2.94-6.04]	70.68	4.74 [2.78-7.08]	3.52 [2.46-4.77]	-1.09 [-1.22 to -0.95]
Malta	Both	0.31 [0.24-0.4]	0.41 [0.31-0.54]	32.26	7.49 [5.72-9.51]	6.02 [4.34-8.03]	0.72 [0.3 to 1.14]
Marshall Islands	Both	0.02 [0.01-0.03]	0.04 [0.02-0.05]	100	9.06 [4.75-14.91]	7.83 [4.57-11.91]	-0.62 [-0.72 to -0.52]
Mauritania	Both	0.6 [0.36-0.89]	0.87 [0.6-1.23]	45	5.15 [2.98-7.74]	3.35 [2.3-4.77]	-1.59 [-1.7 to -1.49]
Mauritius	Both	0.41 [0.29-0.57]	0.7 [0.45-1]	70.73	4.4 [3.09-6.18]	4.12 [2.7-5.85]	-0.19 [-0.36 to -0.02]
Mexico	Both	62.99 [53.46-74.65]	143.01 [113.57-193.52]	127.04	11.73 [9.83-13.82]	11.41 [9.09-15.51]	0 [-0.37 to 0.38]
Micronesia (Federated States of)	Both	0.06 [0.03-0.09]	0.07 [0.04-0.1]	16.67	10.38 [5.39-16.04]	7.98 [4.61-12.33]	-1.09 [-1.19 to -1]
Mongolia	Both	1.15 [0.71-1.7]	1.87 [1.24-2.65]	62.61	8.19 [5.01-12.74]	5.66 [3.79-7.87]	-2.01 [-2.34 to -1.67]
Montenegro	Both	0.24 [0.15-0.34]	0.28 [0.18-0.41]	16.67	3.64 [2.35-5.27]	3.63 [2.36-5.24]	0.01 [0 to 0.03]
Morocco	Both	6.55 [4.17-9.45]	14.02 [9.03-20.23]	114.05	3.42 [2.19-4.94]	3.79 [2.47-5.41]	0.43 [0.38 to 0.47]
Mozambique	Both	4.78 [2.65-8.25]	8.61 [4.83-15.17]	80.13	6.15 [3.26-10.24]	5.93 [3.21-10.56]	0.05 [-0.12 to 0.21]
Myanmar	Both	56.62 [15.49-92.27]	64.94 [24.25-102.59]	14.69	19.97 [5.54-34.38]	12.89 [4.63-20.46]	-1.79 [-2.06 to -1.52]
Namibia	Both	0.33 [0.22-0.47]	0.59 [0.41-0.8]	78.79	3.47 [2.33-4.84]	3.09 [2.17-4.16]	-0.58 [-0.82 to -0.34]
Nepal	Both	10.99 [7.23-15.48]	18.19 [12.36-26.71]	65.51	8.53 [5.44-13.11]	7.16 [4.85-10.59]	-0.51 [-0.77 to -0.25]
Netherlands	Both	17.7 [14.35-21.73]	16.84 [12.74-21.4]	-4.86	9.29 [7.44-11.47]	6.21 [4.59-8.04]	-1.51 [-1.7 to -1.32]
New Zealand	Both	3.88 [3.09-4.93]	4.73 [3.86-5.85]	21.91	10.29 [8.19-13.07]	7.3 [5.82-9.03]	-0.9 [-1.3 to -0.51]
Nicaragua	Both	0.68 [0.47-0.94]	1.97 [1.28-2.68]	189.71	3.11 [2.16-4.26]	3.68 [2.34-5.01]	0.51 [0.2 to 0.81]
Niger	Both	1.97 [1.15-3.38]	3.81 [2.62-5.11]	93.4	4.37 [2.75-6.63]	3.3 [2.22-4.49]	-1.2 [-1.32 to -1.08]
Nigeria	Both	20.41 [13.54-28.63]	39.13 [27.45-53]	91.72	3.53 [2.39-4.93]	2.98 [2.13-4]	-0.69 [-0.77 to -0.62]
North Macedonia	Both	0.78 [0.51-1.11]	1 [0.63-1.44]	28.21	3.83 [2.52-5.38]	3.67 [2.35-5.27]	-0.12 [-0.19 to -0.05]
Northern Mariana Islands	Both	0.02 [0.01-0.03]	0.02 [0.01-0.03]	0	5.45 [3.81-7.63]	3.78 [2.56-5.44]	-1.14 [-1.47 to -0.81]
Norway	Both	6.72 [5.31-8.37]	6.55 [4.73-8.62]	-2.53	11.39 [8.7-14.43]	8.64 [6.1-11.64]	-0.21 [-0.44 to 0.01]
Oman	Both	0.52 [0.33-0.76]	1.82 [1.13-2.75]	250	3.73 [2.42-5.43]	4.18 [2.72-6.04]	0.64 [0.54 to 0.75]
Pakistan	Both	69.51 [46.62-98.49]	141.4 [100.05-191.52]	103.42	9.43 [6.2-13.89]	9.34 [6.61-13.24]	-0.23 [-0.41 to -0.05]
Palestine	Both	0.53 [0.35-0.74]	1.43 [0.95-2.06]	169.81	4.08 [2.72-5.75]	3.88 [2.55-5.55]	-0.19 [-0.3 to -0.07]
Panama	Both	0.45 [0.3-0.64]	1.05 [0.71-1.47]	133.33	2.38 [1.59-3.39]	2.49 [1.69-3.48]	0.25 [0.06 to 0.45]
Papua New Guinea	Both	2.02 [0.99-3.01]	4.51 [2.52-6.67]	123.27	7.7 [3.72-11.66]	6.42 [3.61-9.62]	-0.65 [-0.7 to -0.6]
Paraguay	Both	1.03 [0.69-1.42]	3.01 [1.78-4.2]	192.23	3.65 [2.46-4.97]	4.76 [2.78-6.69]	1.25 [0.94 to 1.57]

Peru	Both	9.2 [6.11-13.03]	19.06 [12.69-26.74]	107.17	5.76 [3.86-8.09]	5.53 [3.7-7.76]	-0.07 [-0.17 to 0.03]
Philippines	Both	103.03 [70.36-130.32]	203.73 [159.65-257.45]	97.74	26.9 [19.7-34.07]	22.66 [18.14-29.72]	-0.66 [-0.76 to -0.56]
Poland	Both	65.99 [54.34-83.95]	17.61 [13.45-23.09]	-73.31	15.34 [12.61-19.47]	3.33 [2.49-4.41]	-5.84 [-6.33 to -5.35]
Portugal	Both	5.73 [4.04-7.77]	8.48 [5.9-12.31]	47.99	4.73 [3.34-6.42]	4.87 [3.37-6.84]	0.38 [0.08 to 0.67]
Puerto Rico	Both	1.09 [0.7-1.57]	1.81 [1.26-2.52]	66.06	3.04 [1.93-4.41]	3.65 [2.51-5.17]	0.65 [0.54 to 0.76]
Qatar	Both	0.16 [0.09-0.25]	1.3 [0.77-2.04]	712.5	3.86 [2.45-5.64]	4 [2.58-5.95]	0.27 [0.21 to 0.34]
Republic of Korea	Both	20.52 [13.95-28.39]	39.36 [26.7-54.31]	91.81	5.37 [3.7-7.39]	4.99 [3.41-7.02]	-0.4 [-0.55 to -0.25]
Republic of Moldova	Both	8.17 [6.19-10.5]	8.93 [6.57-11.66]	9.3	17.84 [13.67-22.8]	17.04 [12.47-22.23]	0.01 [-0.34 to 0.36]
Romania	Both	10.2 [6.71-14.5]	9.94 [6.51-14.17]	-2.55	3.95 [2.59-5.6]	3.92 [2.55-5.52]	1.86 [1.24 to 2.49]
Russian Federation	Both	562.41 [460.32-683.3]	525.88 [424.34-646.79]	-6.5	31.36 [25.72-38.41]	24.65 [19.66-30.59]	-1.06 [-1.39 to -0.73]
Rwanda	Both	2.44 [0.79-4.81]	3.26 [1.51-5.89]	33.61	6.37 [1.67-12.37]	4.48 [1.69-9.06]	-1.87 [-2.14 to -1.6]
Saint Lucia	Both	0.06 [0.05-0.07]	0.17 [0.13-0.22]	183.33	6.11 [4.83-7.73]	7.97 [6.14-10.18]	0.84 [0.69 to 0.99]
Saint Vincent and the Grenadines	Both	0.05 [0.04-0.06]	0.1 [0.08-0.13]	100	6.33 [4.8-8.41]	7.75 [5.76-9.87]	0.93 [0.72 to 1.13]
Samoa	Both	0.08 [0.05-0.12]	0.1 [0.06-0.15]	25	8.06 [4.38-11.57]	6.14 [3.82-8.74]	-1.2 [-1.29 to -1.1]
Sao Tome and Principe	Both	0.03 [0.02-0.04]	0.05 [0.03-0.08]	66.67	3.79 [2.46-5.39]	3.63 [2.25-5.69]	-0.43 [-0.57 to -0.29]
Saudi Arabia	Both	3.95 [2.43-5.86]	14.42 [8.84-21.39]	265.06	3.52 [2.21-5.15]	3.69 [2.36-5.35]	0.23 [0.21 to 0.26]
Senegal	Both	1.71 [1.1-2.51]	3.11 [2.15-4.35]	81.87	4.14 [2.6-6.13]	3.24 [2.28-4.5]	-0.91 [-1.02 to -0.81]
Serbia	Both	5.19 [3.68-7.07]	5.42 [3.84-7.25]	4.43	4.81 [3.44-6.53]	4.48 [3.14-6.03]	0.87 [0.49 to 1.25]
Seychelles	Both	0.04 [0.02-0.06]	0.11 [0.05-0.17]	175	6.85 [3.48-9.66]	9.73 [4.17-14.71]	1.04 [0.79 to 1.28]
Sierra Leone	Both	0.83 [0.55-1.17]	1.63 [1.12-2.27]	96.39	3.51 [2.32-4.99]	3.2 [2.22-4.46]	-0.26 [-0.3 to -0.21]
Singapore	Both	1.9 [1.39-2.51]	3.57 [2.3-5.16]	87.89	6.91 [5.19-9.05]	4.48 [2.9-6.47]	-1.09 [-1.44 to -0.73]
Slovakia	Both	5.13 [3.39-6.71]	3.86 [2.71-5.35]	-24.76	8.86 [5.91-11.52]	5.2 [3.67-7.14]	-1.43 [-1.85 to -1]
Slovenia	Both	1.38 [0.94-2.1]	1.16 [0.78-1.63]	-15.94	5.9 [4.04-8.9]	3.92 [2.62-5.45]	-0.91 [-1.24 to -0.59]
Solomon Islands	Both	0.2 [0.11-0.31]	0.42 [0.23-0.7]	110	10.7 [5.64-16.26]	9.04 [5.06-14.12]	-0.56 [-0.66 to -0.45]
Somalia	Both	3.03 [1.43-6.07]	7.42 [3.46-13.63]	144.88	7.45 [3.51-13.82]	7.56 [3.37-14.06]	0.21 [0.09 to 0.32]
South Africa	Both	11.56 [8.56-14.88]	18.1 [13.34-23.51]	56.57	3.84 [2.91-4.92]	3.33 [2.48-4.27]	-0.49 [-0.89 to -0.09]
South Sudan	Both	1.43 [0.79-2.49]	2.15 [1.24-3.57]	50.35	4.29 [2.39-7.28]	3.96 [2.33-6.54]	-0.35 [-0.46 to -0.24]
Spain	Both	26.21 [19.39-33.99]	34.66 [24.34-46.64]	32.24	5.57 [4.08-7.36]	4.88 [3.37-6.74]	-0.37 [-0.47 to -0.27]

Sri Lanka	Both	5.4 [3.5-7.97]	10.37 [6.73-14.9]	92.04	3.92 [2.58-5.68]	4.07 [2.67-5.82]	0.17 [0.11 to 0.23]
Sudan	Both	4.76 [3.08-6.8]	11.41 [7.52-16.39]	139.71	3.5 [2.26-5.14]	3.83 [2.53-5.47]	0.43 [0.37 to 0.49]
Suriname	Both	0.22 [0.16-0.31]	0.75 [0.49-1.02]	240.91	7.4 [5.41-10.42]	11.95 [7.71-16.16]	1.65 [1.32 to 1.98]
Sweden	Both	9.2 [7.16-11.77]	8.9 [6.84-11.3]	-3.26	7.27 [5.52-9.25]	5.43 [4.02-7.14]	-0.47 [-0.91 to -0.04]
Switzerland	Both	5.09 [3.69-6.8]	6.11 [4.12-8.48]	20.04	5.67 [4.04-7.63]	4.81 [3.19-6.79]	-0.34 [-0.6 to -0.07]
Syrian Arab	Both	3.19 [2.1-4.5]	5.4 [3.5-7.94]	69.28	4.04 [2.65-5.84]	3.67 [2.39-5.34]	-0.47 [-0.56 to -0.39]
Tajikistan	Both	3.35 [2.12-4.49]	8.19 [5.18-11.25]	144.48	9.01 [5.77-12.04]	11.56 [7.72-15.43]	0.74 [0.55 to 0.92]
Thailand	Both	76.1 [37.7-111.16]	120.63 [63.91-181.97]	58.52	19.59 [9.28-29]	12.1 [6.37-18.34]	-2.36 [-2.92 to -1.8]
Timor-Leste	Both	0.56 [0.18-0.95]	1.04 [0.38-1.7]	85.71	13.31 [4.09-25.49]	11.72 [4.11-19.36]	-0.59 [-0.77 to -0.4]
Togo	Both	0.71 [0.47-1.03]	1.65 [1.13-2.32]	132.39	3.87 [2.46-5.87]	3.21 [2.22-4.47]	-0.78 [-0.86 to -0.69]
Tonga	Both	0.04 [0.02-0.05]	0.05 [0.03-0.06]	25	5.85 [3.57-8.57]	5.37 [3.56-7.5]	-0.41 [-0.51 to -0.3]
Trinidad and Tobago	Both	1 [0.79-1.38]	3.32 [2.37-4.89]	232	11 [8.63-15.45]	18.05 [12.95-26.14]	2.84 [2.28 to 3.4]
Tunisia	Both	2.22 [1.4-3.2]	4.75 [3.04-6.9]	113.96	3.37 [2.11-4.83]	3.59 [2.33-5.18]	0.24 [0.21 to 0.27]
Turkey	Both	22.56 [15.62-31.14]	42.16 [29.39-58.16]	86.88	4.77 [3.3-6.63]	4.55 [3.22-6.24]	0.07 [-0.1 to 0.23]
Turkmenistan	Both	1.59 [1.12-2.18]	4.13 [3.08-5.4]	159.75	5.84 [4.1-8.06]	8.95 [6.79-11.48]	1.79 [1.64 to 1.94]
Uganda	Both	3.37 [1.77-5.83]	7.92 [4.48-12.95]	135.01	3.85 [1.79-6.56]	4.04 [2.04-6.82]	-0.1 [-0.3 to 0.11]
Ukraine	Both	180.03 [142.93-224.99]	149.02 [113.97-188.1]	-17.22	26.2 [20.66-32.85]	22.63 [17.36-28.82]	-1.07 [-1.32 to -0.81]
United Arab Emirates	Both	0.63 [0.37-0.98]	5.48 [3.21-8.61]	769.84	3.87 [2.49-5.71]	4.28 [2.71-6.17]	0.55 [0.46 to 0.65]
United Kingdom	Both	58.45 [42.46-74.36]	81.31 [64.38-101.75]	39.11	7.76 [5.74-9.98]	8.21 [6.29-10.27]	0.65 [0.4 to 0.91]
United Republic of Tanzania	Both	6.17 [3.67-10.3]	13.49 [7.92-23.15]	118.64	4.41 [2.55-7.29]	4.19 [2.45-7.31]	-0.1 [-0.23 to 0.02]
United States of America	Both	183.9 [136.29-238.99]	207.13 [166.06-257.85]	12.63	6.36 [4.68-8.35]	4.49 [3.55-5.58]	-1.19 [-1.49 to -0.88]
United States Virgin Islands	Both	0.12 [0.09-0.18]	0.27 [0.2-0.35]	125	12.53 [9.29-18.71]	16.28 [12.06-21.55]	1.39 [1.16 to 1.63]
Uruguay	Both	1.75 [1.19-2.46]	2.5 [1.81-3.37]	42.86	5.29 [3.56-7.48]	6.03 [4.23-8.25]	0.54 [0.47 to 0.62]
Uzbekistan	Both	7.06 [4.4-10.44]	15.17 [9.54-22.24]	114.87	4.51 [2.85-6.57]	4.84 [3.1-6.96]	0.29 [0.25 to 0.34]
Vanuatu	Both	0.07 [0.04-0.1]	0.15 [0.09-0.23]	114.29	7.55 [4.07-11.54]	7.03 [4.21-11.22]	-0.43 [-0.56 to -0.31]
Venezuela (Bolivarian Republic of)	Both	8.14 [6.42-10.86]	24.81 [18.42-32.87]	204.79	6.09 [4.82-8.49]	8.27 [6.17-10.95]	0.39 [-0.22 to 1]
Viet Nam	Both	35.46 [15.63-52.38]	89.87 [49.36-131.89]	153.44	7.85 [3.39-11.76]	8.52 [4.68-12.4]	0.51 [0.16 to 0.86]
Yemen	Both	2.81 [1.8-4.13]	8.57 [5.67-12.39]	204.98	3.45 [2.15-5.13]	3.86 [2.53-5.54]	0.47 [0.41 to 0.52]

Zambia	Both	2.77 [1.6-5.08]	5.06 [2.97-9.04]	82.67	6.34 [3.66-11.15]	5.26 [2.98-9.66]	-0.86 [-0.98 to -0.74]
Zimbabwe	Both	2.38 [1.39-3.51]	4.52 [2.58-7.1]	89.92	3.21 [2.11-4.38]	3.7 [2.35-5.27]	0.52 [0.18 to 0.86]
Afghanistan	Male	1.72 [1.07-2.68]	5.92 [3.78-8.82]	244.19	4.35 [2.64-6.72]	4.81 [3.03-7.24]	0.49 [0.43 to 0.56]
Albania	Male	1.28 [0.59-2.05]	0.67 [0.41-0.98]	-47.66	9.58 [4.77-13.98]	3.96 [2.47-5.75]	-2.55 [-3.28 to -1.8]
Algeria	Male	3.64 [2.21-5.56]	9.3 [5.66-14.46]	155.49	4.14 [2.52-6.38]	4.33 [2.69-6.71]	0.22 [0.18 to 0.26]
American Samoa	Male	0.01 [0.01-0.02]	0.02 [0.01-0.02]	100	9.31 [5.13-14.47]	7.07 [4.7-9.88]	-1.51 [-2.3 to -0.71]
Andorra	Male	0.03 [0.02-0.04]	0.05 [0.03-0.07]	66.67	9.9 [5.77-14.37]	7.88 [5.06-11.03]	-0.68 [-0.83 to -0.53]
Angola	Male	2.44 [1.2-5.16]	4.26 [2.52-7]	74.59	8.84 [4.18-16.68]	5.92 [3.28-9.81]	-1.65 [-1.78 to -1.52]
Antigua and Barbuda	Male	0.01 [0.01-0.02]	0.03 [0.02-0.04]	200	4.87 [3.34-6.87]	5.54 [3.83-7.71]	0.52 [0.48 to 0.56]
Argentina	Male	9.64 [6.21-14.3]	15.52 [9.89-23.09]	61	6.26 [4.03-9.33]	6.6 [4.2-9.82]	0.21 [0.16 to 0.27]
Armenia	Male	3.16 [2.25-4.86]	5.86 [3.78-12.18]	85.44	26.43 [19.12-41.06]	34.62 [22.15-72.14]	1.01 [0.79 to 1.24]
Australia	Male	8.62 [6.51-11.19]	10.62 [7.52-14.45]	23.2	9.77 [7.45-12.54]	6.52 [4.56-9.03]	-1.27 [-1.55 to -0.99]
Austria	Male	7.89 [5.99-10.18]	6.81 [4.19-10.87]	-13.69	17.78 [13.64-22.94]	10.87 [6.62-17.39]	-0.69 [-0.95 to -0.43]
Azerbaijan	Male	1.87 [1.16-2.85]	3.67 [2.26-5.42]	96.26	6.31 [3.99-9.51]	6.76 [4.25-9.8]	0.33 [0.26 to 0.4]
Bahamas	Male	0.06 [0.04-0.08]	0.15 [0.11-0.21]	150	6.26 [4.55-8.34]	7.59 [5.5-10.17]	0.71 [0.6 to 0.82]
Bahrain	Male	0.11 [0.07-0.18]	0.56 [0.33-0.88]	409.09	4.17 [2.61-6.09]	4.77 [3-7.08]	0.55 [0.44 to 0.65]
Bangladesh	Male	29.57 [18.65-43.77]	49.57 [32.8-71.11]	67.64	7.84 [4.82-11.47]	6.68 [4.46-9.53]	-0.33 [-0.46 to -0.19]
Barbados	Male	0.1 [0.08-0.15]	0.22 [0.15-0.29]	120	9.02 [6.67-12.68]	10.65 [7.14-14.11]	0.53 [0.34 to 0.71]
Belarus	Male	16.05 [11.82-21.36]	15.26 [9.94-20.95]	-4.92	31.57 [23.32-41.57]	26.06 [17.09-35.64]	-0.98 [-1.1 to -0.86]
Belgium	Male	4.29 [2.88-6.12]	6.02 [4.08-8.43]	40.33	7.25 [4.86-10.3]	7.58 [5.1-10.77]	1.71 [0.89 to 2.53]
Belize	Male	0.03 [0.02-0.05]	0.16 [0.11-0.21]	433.33	5.56 [3.85-7.83]	9.42 [6.75-12.25]	1.71 [1.29 to 2.12]
Benin	Male	0.37 [0.25-0.51]	1.04 [0.7-1.47]	181.08	2.87 [1.91-4.1]	3.16 [2.13-4.53]	0.4 [0.36 to 0.44]
Bermuda	Male	0.02 [0.01-0.02]	0.02 [0.02-0.03]	0	4.9 [3.39-6.84]	5.15 [3.49-7.23]	0.29 [0.25 to 0.33]
Bhutan	Male	0.17 [0.1-0.29]	0.27 [0.18-0.39]	58.82	9.64 [4.93-17.79]	7.83 [5.18-11.46]	-0.73 [-0.86 to -0.6]
Bolivia (Plurinational State of)	Male	2.23 [1.32-3.45]	4.65 [3.12-6.59]	108.52	11.18 [6.41-17.77]	9.3 [6.18-13.37]	-0.53 [-0.62 to -0.43]
Bosnia and Herzegovina	Male	1.19 [0.79-1.64]	0.92 [0.6-1.3]	-22.69	5.79 [3.77-8]	4.15 [2.76-5.86]	-1.45 [-1.77 to -1.14]
Botswana	Male	0.17 [0.12-0.24]	0.42 [0.28-0.58]	147.06	4.44 [3.01-6.48]	4.51 [3.01-6.44]	-0.2 [-0.58 to 0.17]
Brazil	Male	34.03 [26.32-43.4]	95.23 [75.67-143.38]	179.84	6.27 [4.92-7.93]	8.46 [6.74-12.91]	1.38 [1.28 to 1.48]

Brunei Darussalam	Male	0.1 [0.07-0.15]	0.19 [0.13-0.27]	90	16.09 [9.67-26.64]	11.53 [7.82-15.77]	-0.77 [-1.17 to -0.38]
Bulgaria	Male	13.8 [9.53-25.21]	2.38 [1.62-3.31]	-82.75	24.54 [17.16-43.88]	4.82 [3.25-6.7]	-5.98 [-7 to -4.94]
Burkina Faso	Male	0.82 [0.55-1.15]	1.99 [1.32-2.91]	142.68	2.87 [1.95-4.03]	3.05 [2.03-4.34]	0.25 [0.16 to 0.33]
Burundi	Male	0.39 [0.22-0.8]	0.72 [0.45-1.21]	84.62	2.03 [1.27-3.59]	1.8 [1.14-2.7]	-0.56 [-0.6 to -0.52]
Cabo Verde	Male	0.03 [0.02-0.04]	0.07 [0.04-0.1]	133.33	2.51 [1.65-3.64]	2.87 [1.88-4.1]	0.34 [0.26 to 0.41]
Cambodia	Male	6.39 [1.86-10.66]	11.79 [4.59-17.6]	84.51	26.07 [7.6-46.93]	20.65 [7.62-31.01]	-0.93 [-1.01 to -0.85]
Cameroon	Male	0.85 [0.58-1.17]	2.7 [1.79-3.78]	217.65	2.86 [1.94-3.97]	3.27 [2.13-4.52]	0.52 [0.45 to 0.59]
Canada	Male	7.31 [5.31-9.75]	14.36 [10.7-18.95]	96.44	5.05 [3.71-6.72]	5.33 [3.93-7.04]	0.51 [0.37 to 0.65]
Central African Republic	Male	0.66 [0.33-1.37]	1.31 [0.65-2.62]	98.48	9.03 [4.35-17.51]	9.26 [4.38-18.16]	0.05 [-0.05 to 0.14]
Chad	Male	0.49 [0.32-0.7]	1.3 [0.87-1.85]	165.31	2.81 [1.85-4.07]	3.18 [2.08-4.6]	0.48 [0.39 to 0.57]
Chile	Male	4.18 [2.82-6]	7.85 [5.35-10.98]	87.8	7.4 [5.1-10.47]	7.51 [5.13-10.55]	-0.86 [-1.11 to -0.61]
China	Male	1020.58 [574.63-1293.63]	695.25 [510.85-902.51]	-31.88	22.79 [12.17-28.83]	7.54 [5.52-9.74]	-4.25 [-4.53 to -3.96]
Colombia	Male	4.78 [3.47-6.34]	9.48 [6.69-13.06]	98.33	3.99 [2.92-5.26]	3.89 [2.74-5.34]	0.32 [-0.01 to 0.65]
Comoros	Male	0.03 [0.02-0.04]	0.05 [0.03-0.07]	66.67	1.67 [1.07-2.43]	1.62 [1.09-2.3]	-0.26 [-0.38 to -0.14]
Congo	Male	0.49 [0.27-0.98]	0.89 [0.56-1.31]	81.63	7.94 [4.22-15.67]	5.3 [3.2-7.79]	-1.88 [-2.15 to -1.61]
Costa Rica	Male	0.31 [0.19-0.45]	0.71 [0.47-1.02]	129.03	2.64 [1.64-3.85]	2.92 [1.94-4.16]	0.43 [0.37 to 0.49]
Cote d'Ivoire	Male	1.03 [0.69-1.47]	2.62 [1.74-3.7]	154.37	2.95 [1.95-4.22]	3.24 [2.13-4.6]	0.26 [0.2 to 0.31]
Croatia	Male	1.51 [1.1-2.02]	1.72 [1.19-2.45]	13.91	5.76 [4.3-7.57]	5.65 [3.89-8.05]	0.05 [-0.15 to 0.26]
Cuba	Male	4.15 [3.05-5.78]	9.93 [7.16-13.36]	139.28	8.01 [5.9-11.21]	11.85 [8.61-15.84]	1.49 [1.26 to 1.73]
Cyprus	Male	0.3 [0.17-0.45]	0.46 [0.29-0.66]	53.33	7.53 [4.34-11.81]	5.46 [3.5-7.89]	-2.62 [-3.15 to -2.1]
Czechia	Male	13.9 [9.83-25.83]	3.99 [2.85-5.53]	-71.29	25.32 [17.9-47.16]	5.32 [3.81-7.34]	-3.33 [-4.15 to -2.5]
Democratic People's Republic of Korea	Male	12.57 [5.67-18.37]	18.41 [9.67-27.09]	46.46	15.94 [7.11-23.06]	12.77 [6.62-18.7]	-0.86 [-1.07 to -0.65]
Democratic Republic of the Congo	Male	5.69 [3.33-9.64]	12.34 [7.48-20.13]	116.87	5.73 [3.08-9.16]	5.34 [3.03-9.35]	-0.23 [-0.32 to -0.15]
Denmark	Male	2.69 [1.99-3.71]	3.84 [2.93-5.01]	42.75	8.35 [6.1-11.61]	8.6 [6.44-11.36]	0.23 [0 to 0.46]
Djibouti	Male	0.03 [0.02-0.05]	0.09 [0.06-0.13]	200	1.66 [1.07-2.49]	1.76 [1.13-2.54]	0.12 [-0.02 to 0.25]
Dominica	Male	0.01 [0.01-0.02]	0.03 [0.02-0.03]	200	4.7 [3.16-6.81]	5.91 [4.11-8.09]	0.85 [0.8 to 0.9]
Dominican Republic	Male	1.24 [0.83-1.7]	2.52 [1.68-3.59]	103.23	5.06 [3.42-7.04]	4.95 [3.35-7.01]	0.13 [-0.1 to 0.36]

Ecuador	Male	2.62 [1.79-3.66]	7.16 [5.04-9.51]	173.28	7.45 [5.12-10.34]	8.92 [6.32-11.94]	1.34 [1.07 to 1.6]
Egypt	Male	8.95 [5.58-13.46]	20.28 [12.37-30.78]	126.59	4.11 [2.58-6.13]	4.38 [2.69-6.58]	0.28 [0.25 to 0.3]
El Salvador	Male	0.6 [0.4-0.86]	0.84 [0.56-1.17]	40	3.57 [2.36-5.12]	3.22 [2.12-4.44]	-0.43 [-0.64 to -0.23]
Equatorial Guinea	Male	0.09 [0.04-0.19]	0.16 [0.1-0.23]	77.78	8.48 [3.71-16.87]	4.66 [2.77-7.29]	-2.29 [-2.78 to -1.8]
Eritrea	Male	0.21 [0.11-0.45]	0.43 [0.27-0.75]	104.76	2.04 [1.24-3.52]	1.87 [1.23-2.91]	-0.34 [-0.43 to -0.25]
Estonia	Male	2.53 [1.89-3.68]	1.57 [1.12-2.14]	-37.94	34.12 [25.7-50.94]	18.33 [12.93-25.06]	-3.16 [-3.61 to -2.71]
Eswatini	Male	0.1 [0.07-0.15]	0.22 [0.15-0.31]	120	4.95 [3.29-7.3]	5.8 [3.89-8.18]	0.82 [0.27 to 1.37]
Ethiopia	Male	5.64 [2.99-12.23]	6.43 [4.24-9.19]	14.01	2.99 [1.76-5.87]	1.82 [1.2-2.5]	-1.9 [-2.02 to -1.79]
Fiji	Male	0.11 [0.07-0.17]	0.22 [0.15-0.32]	100	4.15 [2.61-6.12]	5.25 [3.56-7.24]	1.14 [0.94 to 1.34]
Finland	Male	1.53 [0.94-2.27]	2.21 [1.52-3.13]	44.44	5.24 [3.24-7.73]	5.66 [3.75-8.11]	-0.1 [-0.65 to 0.46]
France	Male	24.62 [17.38-33.75]	28.03 [19.04-39.43]	13.85	7.65 [5.41-10.54]	6.43 [4.24-9.22]	-0.55 [-0.66 to -0.44]
Gabon	Male	0.21 [0.11-0.43]	0.36 [0.21-0.58]	71.43	6.89 [3.64-14.14]	5.98 [3.43-10.07]	-0.52 [-0.63 to -0.41]
Gambia	Male	0.08 [0.05-0.11]	0.21 [0.14-0.29]	162.5	2.74 [1.86-3.81]	3.29 [2.2-4.65]	0.75 [0.67 to 0.83]
Germany	Male	32.21 [22.57-44.06]	38.59 [26.14-54.29]	19.81	6.64 [4.67-9.04]	6.13 [4.07-8.69]	1.51 [0.98 to 2.05]
Ghana	Male	4.5 [1.2-8.59]	11.32 [3.19-18.35]	151.56	13.51 [2.97-27.53]	14.75 [3.49-24.53]	0.96 [0.69 to 1.23]
Greece	Male	3.51 [2.22-5.19]	4.1 [2.57-6.08]	16.81	5.69 [3.61-8.49]	5.73 [3.53-8.49]	0.09 [0.03 to 0.15]
Greenland	Male	0.01 [0.01-0.01]	0.01 [0.01-0.02]	0	4.12 [2.57-5.98]	3.42 [2.19-5.01]	-0.75 [-0.96 to -0.55]
Grenada	Male	0.04 [0.03-0.05]	0.11 [0.08-0.14]	175	11.64 [8.83-15.7]	18.88 [13.34-24.43]	2.1 [1.69 to 2.52]
Guam	Male	0.03 [0.02-0.04]	0.04 [0.03-0.06]	33.33	5.27 [3.19-7.64]	4.35 [2.77-6.42]	-0.75 [-1.07 to -0.43]
Guatemala	Male	1.6 [1.14-2.12]	2.97 [2.14-4.08]	85.62	6.01 [4.44-7.83]	4.71 [3.35-6.47]	-0.89 [-1.18 to -0.59]
Guinea	Male	0.56 [0.37-0.82]	1.15 [0.78-1.65]	105.36	2.82 [1.84-4.12]	3.3 [2.16-4.8]	0.7 [0.65 to 0.76]
Guinea-Bissau	Male	0.1 [0.06-0.14]	0.17 [0.12-0.24]	70	3.54 [2.34-5.13]	3.56 [2.33-5.03]	0.08 [0.02 to 0.13]
Guyana	Male	0.24 [0.18-0.34]	0.5 [0.33-0.67]	108.33	10.43 [7.85-14.71]	14.48 [9.59-19.24]	1.34 [1.06 to 1.62]
Haiti	Male	3.38 [1.37-6.91]	5.77 [2.91-10.62]	70.71	14.97 [6.59-27.77]	14.07 [6.71-28.54]	0.16 [0 to 0.32]
Honduras	Male	1.56 [0.59-2.48]	3.03 [1.51-4.91]	94.23	11.94 [4.39-19.07]	9.53 [4.49-15.81]	-0.67 [-0.82 to -0.51]
Hungary	Male	16.52 [12.19-23.43]	6.46 [4.77-8.68]	-60.9	28.57 [21.07-40.79]	9.24 [6.87-12.29]	-3.65 [-4.16 to -3.14]
Iceland	Male	0.1 [0.07-0.13]	0.17 [0.12-0.22]	70	7.75 [5.8-10.32]	7.25 [5.15-9.79]	-0.75 [-1.11 to -0.38]
India	Male	409.54 [243.8-593.05]	666.58 [470.52-922.26]	62.76	14.4 [7.96-22.06]	10.44 [7.32-14.55]	-1.13 [-1.31 to -0.95]
Indonesia	Male	181.76 [65.1-268.07]	258.99 [81.2-386.22]	42.49	31.24 [10.97-46.89]	22.39 [6.66-33.21]	-1.28 [-1.41 to -1.15]

Iran (Islamic Republic of)	Male	9.64 [6.27-13.72]	24.09 [16.01-34.07]	149.9	4.64 [2.99-6.62]	5.2 [3.44-7.26]	0.5 [0.35 to 0.65]
Iraq	Male	5.26 [3.23-9.7]	9.09 [6.08-12.79]	72.81	8.42 [5.3-13.56]	5.18 [3.52-7.25]	-2.25 [-2.54 to -1.97]
Ireland	Male	1.51 [1.09-1.99]	1.95 [1.29-2.74]	29.14	8.53 [6.13-11.24]	6.38 [4.18-9.07]	-1 [-1.3 to -0.7]
Israel	Male	1.45 [0.94-2.1]	3.85 [2.71-5.23]	165.52	6.64 [4.29-9.58]	7.86 [5.44-10.74]	0.43 [0.21 to 0.65]
Italy	Male	36.95 [28.04-47.61]	31.78 [22.42-42.97]	-13.99	10.71 [8.15-13.84]	6.93 [4.78-9.41]	-1.53 [-1.79 to -1.26]
Jamaica	Male	0.4 [0.27-0.57]	1.1 [0.78-1.52]	175	4.54 [3.02-6.57]	7.54 [5.38-10.39]	2.28 [1.98 to 2.57]
Japan	Male	58.76 [39.98-83]	77.13 [57.39-101.75]	31.26	7.82 [5.4-10.82]	7.72 [5.49-10.34]	-0.02 [-0.08 to 0.04]
Jordan	Male	0.55 [0.36-0.8]	3.49 [2.02-5.71]	534.55	4.26 [2.74-6.18]	6.11 [3.64-9.73]	1.73 [1.39 to 2.07]
Kazakhstan	Male	14.67 [9.66-25.36]	21 [15.1-28.04]	43.15	28.17 [18.8-50.69]	28.83 [21.03-38.72]	-0.55 [-0.89 to -0.21]
Kenya	Male	1.17 [0.79-1.62]	3.21 [2.18-4.43]	174.36	1.68 [1.14-2.33]	1.81 [1.23-2.49]	1.08 [0.76 to 1.4]
Kuwait	Male	0.4 [0.24-0.61]	1.28 [0.76-2]	220	4.13 [2.58-6.17]	4.42 [2.83-6.55]	0.36 [0.3 to 0.42]
Kyrgyzstan	Male	3.72 [2.76-5.23]	3.83 [2.85-5.16]	2.96	21.28 [16.07-29.54]	13.58 [10.12-18.04]	-1.34 [-1.77 to -0.92]
Lao People's Democratic Republic)	Male	3.53 [0.89-6.78]	4.39 [1.73-7.19]	24.36	29.12 [7.35-59.98]	17.49 [6.33-29.88]	-2.06 [-2.25 to -1.87]
Latvia	Male	4.86 [3.72-6.43]	3.75 [2.73-4.85]	-22.84	37.98 [29.18-49.91]	28.38 [20.66-36.73]	-2.12 [-2.53 to -1.71]
Lebanon	Male	0.53 [0.33-0.81]	1.07 [0.65-1.62]	101.89	4.01 [2.45-6.12]	4.24 [2.55-6.32]	0.26 [0.23 to 0.29]
Lesotho	Male	0.27 [0.18-0.43]	0.49 [0.34-0.71]	81.48	4.59 [2.95-7.48]	6.36 [4.32-9.47]	1.56 [1.25 to 1.87]
Liberia	Male	0.2 [0.13-0.28]	0.46 [0.3-0.66]	130	2.89 [1.88-4.1]	3.01 [2-4.48]	0.22 [0.13 to 0.32]
Libya	Male	0.64 [0.39-0.96]	1.68 [1.01-2.59]	162.5	4.05 [2.46-6.11]	4.36 [2.73-6.49]	0.31 [0.27 to 0.34]
Lithuania	Male	7.01 [5.4-9.03]	3.79 [2.79-5.06]	-45.93	39.75 [30.84-51]	20.53 [15.01-27.73]	-3.31 [-3.67 to -2.95]
Luxembourg	Male	0.18 [0.13-0.25]	0.3 [0.2-0.42]	66.67	8.18 [5.75-11.1]	7.01 [4.63-9.93]	-0.43 [-0.96 to 0.11]
Madagascar	Male	0.8 [0.47-1.59]	1.46 [0.94-2.17]	82.5	1.78 [1.13-2.94]	1.59 [1.06-2.26]	-0.31 [-0.38 to -0.24]
Malawi	Male	0.62 [0.36-1.25]	1.09 [0.68-1.84]	75.81	1.9 [1.21-3.05]	1.85 [1.22-2.73]	-0.16 [-0.23 to -0.09]
Malaysia	Male	4.36 [2.86-6.35]	11.5 [7.35-16.52]	163.76	7.51 [4.84-10.79]	7.49 [4.79-10.95]	0.03 [-0.1 to 0.15]
Maldives	Male	0.05 [0.03-0.07]	0.19 [0.11-0.29]	280	7.38 [4.73-10.72]	6.61 [4.28-9.63]	-0.46 [-0.58 to -0.35]
Mali	Male	0.77 [0.52-1.09]	1.81 [1.22-2.54]	135.06	2.86 [1.89-4.12]	3.05 [2.05-4.33]	0.34 [0.23 to 0.46]
Malta	Male	0.2 [0.14-0.26]	0.27 [0.19-0.36]	35	10.19 [7.63-13.34]	8.13 [5.72-11.25]	0.84 [0.34 to 1.34]
Marshall Islands	Male	0.01 [0.01-0.02]	0.02 [0.01-0.03]	100	8.98 [4.72-15.77]	7.36 [4.33-11.59]	-0.61 [-0.71 to -0.51]
Mauritania	Male	0.18 [0.12-0.25]	0.37 [0.25-0.53]	105.56	2.89 [1.95-4.01]	2.89 [1.96-4.12]	0.12 [0.01 to 0.22]

Mauritius	Male	0.3 [0.2-0.43]	0.52 [0.33-0.76]	73.33	6.79 [4.6-9.69]	6.31 [4-9.16]	-0.19 [-0.35 to -0.03]
Mexico	Male	35.76 [28.25-43.72]	72.61 [56.52-98.46]	103.05	13.99 [10.88-17.07]	12.35 [9.62-16.83]	-0.46 [-0.83 to -0.08]
Micronesia (Federated States of)	Male	0.03 [0.01-0.04]	0.03 [0.02-0.05]	0	9.33 [4.68-15.35]	7.37 [4.46-10.77]	-0.87 [-0.96 to -0.79]
Mongolia	Male	0.53 [0.34-0.79]	1.08 [0.7-1.57]	103.77	7.09 [4.65-10.16]	6.86 [4.5-9.94]	-0.43 [-0.55 to -0.3]
Montenegro	Male	0.12 [0.07-0.17]	0.14 [0.09-0.21]	16.67	3.72 [2.34-5.49]	3.69 [2.28-5.43]	0.01 [-0.01 to 0.03]
Morocco	Male	3.9 [2.39-5.85]	8.21 [5.01-12.31]	110.51	4.08 [2.47-6.17]	4.42 [2.74-6.63]	0.3 [0.26 to 0.34]
Mozambique	Male	0.89 [0.51-1.76]	1.8 [1.09-3.36]	102.25	1.94 [1.17-3.23]	2.05 [1.28-3.33]	0.45 [0.36 to 0.55]
Myanmar	Male	35.79 [10.66-63.28]	43.69 [16.98-67.75]	22.07	27.65 [8.07-52.18]	19.51 [7.33-30.2]	-1.44 [-1.68 to -1.21]
Namibia	Male	0.21 [0.14-0.3]	0.38 [0.26-0.52]	80.95	4.64 [3.05-6.8]	4.42 [3.09-6.16]	-0.37 [-0.68 to -0.07]
Nepal	Male	5.73 [3.37-9.06]	9.49 [6.16-14.36]	65.62	9.07 [4.99-15.7]	8.1 [5.16-12.65]	-0.26 [-0.49 to -0.04]
Netherlands	Male	9.44 [7.06-12.2]	9.91 [7.05-13.28]	4.98	11.33 [8.53-14.54]	7.89 [5.48-10.62]	-1.23 [-1.38 to -1.08]
New Zealand	Male	1.92 [1.44-2.49]	2.46 [1.87-3.2]	28.13	10.89 [8.25-14.13]	8.05 [6.07-10.65]	-0.95 [-1.28 to -0.61]
Nicaragua	Male	0.43 [0.29-0.62]	1.03 [0.69-1.43]	139.53	4.23 [2.84-6.03]	4.16 [2.72-5.78]	-0.36 [-0.69 to -0.02]
Niger	Male	0.67 [0.44-0.97]	1.66 [1.09-2.34]	147.76	2.97 [1.93-4.26]	3.03 [1.93-4.38]	0.14 [0.07 to 0.21]
Nigeria	Male	9.24 [6.37-12.86]	18.92 [12.61-26.42]	104.76	2.92 [2.02-4.04]	3.18 [2.19-4.4]	0.33 [0.26 to 0.39]
North Macedonia	Male	0.4 [0.26-0.58]	0.52 [0.33-0.76]	30	3.97 [2.6-5.7]	3.73 [2.35-5.38]	-0.19 [-0.28 to -0.1]
Northern Mariana Islands	Male	0.01 [0.01-0.01]	0.01 [0.01-0.02]	0	4.77 [3.07-7.02]	4.52 [2.96-6.57]	-0.23 [-0.38 to -0.08]
Norway	Male	4.31 [3.27-5.55]	4.43 [3.06-6]	2.78	16.26 [12.12-20.8]	12.06 [8.23-16.5]	-0.16 [-0.42 to 0.09]
Oman	Male	0.38 [0.23-0.58]	1.41 [0.83-2.24]	271.05	4.2 [2.65-6.19]	4.64 [2.98-6.75]	0.57 [0.49 to 0.66]
Pakistan	Male	39.97 [23.49-62.71]	78.81 [52.04-115.22]	97.17	10.34 [5.66-17.34]	10.39 [6.63-15.84]	-0.18 [-0.37 to 0.02]
Palestine	Male	0.28 [0.18-0.41]	0.87 [0.56-1.29]	210.71	4.73 [3-6.87]	4.69 [3-6.86]	-0.04 [-0.14 to 0.06]
Panama	Male	0.26 [0.16-0.38]	0.6 [0.39-0.86]	130.77	2.68 [1.72-3.94]	2.87 [1.9-4.13]	0.33 [0.22 to 0.44]
Papua New Guinea	Male	0.9 [0.52-1.44]	2.17 [1.38-3.27]	141.11	6.89 [3.81-12.03]	6.08 [3.71-9.53]	-0.37 [-0.46 to -0.28]
Paraguay	Male	0.61 [0.38-0.86]	1.53 [0.92-2.24]	150.82	4.56 [2.74-6.41]	4.95 [2.9-7.33]	0.31 [-0.02 to 0.64]
Peru	Male	5.73 [3.73-8.29]	11.66 [7.54-16.95]	103.49	7.25 [4.71-10.38]	6.94 [4.5-10.04]	-0.05 [-0.15 to 0.06]
Philippines	Male	73.88 [46.03-99.62]	144.54 [105.63-203.22]	95.64	39.65 [27.54-54.19]	33.81 [24.87-51.02]	-0.64 [-0.75 to -0.53]
Poland	Male	33.41 [25.25-43.97]	8.51 [6.32-11.01]	-74.53	17.71 [13.34-23.29]	3.36 [2.51-4.39]	-6.36 [-6.89 to -5.84]
Portugal	Male	3.83 [2.7-5.24]	4.53 [3.17-6.3]	18.28	6.9 [4.85-9.38]	5.8 [4.01-7.95]	-0.51 [-0.77 to -0.25]

Puerto Rico	Male	0.66 [0.4-0.98]	1.1 [0.74-1.61]	66.67	3.93 [2.38-5.82]	4.74 [3.12-7.05]	0.85 [0.78 to 0.92]
Qatar	Male	0.13 [0.07-0.21]	1.11 [0.64-1.76]	753.85	4.19 [2.62-6.26]	4.24 [2.64-6.42]	0.08 [0.04 to 0.12]
Republic of Korea	Male	14.63 [9.52-20.89]	26.05 [16.95-36.91]	78.06	8.42 [5.59-11.95]	6.89 [4.56-9.73]	-1.01 [-1.21 to -0.8]
Republic of Moldova	Male	4.65 [3.31-6.23]	4.89 [3.4-6.57]	5.16	23.37 [16.81-31.01]	21.04 [14.73-28.21]	-0.38 [-0.74 to -0.02]
Romania	Male	4.63 [2.92-6.71]	4.74 [3.04-7.03]	2.38	3.71 [2.35-5.37]	3.79 [2.46-5.53]	2.16 [1.46 to 2.86]
Russian Federation	Male	290.85 [219.8-378.45]	269.83 [204.96-348.76]	-7.23	42.74 [31.52-58.05]	31.01 [23.54-40.13]	-1.35 [-1.67 to -1.03]
Rwanda	Male	0.51 [0.29-1.04]	0.73 [0.47-1.17]	43.14	2.08 [1.3-3.65]	1.68 [1.13-2.45]	-1.24 [-1.43 to -1.06]
Saint Lucia	Male	0.02 [0.01-0.03]	0.05 [0.03-0.07]	150	4.38 [2.97-6.2]	4.91 [3.32-6.86]	0.39 [0.33 to 0.45]
Saint Vincent and the Grenadines	Male	0.04 [0.03-0.05]	0.09 [0.06-0.11]	125	11.13 [8.5-15.09]	12.74 [9.22-16.41]	0.74 [0.48 to 0.99]
Samoa	Male	0.04 [0.02-0.06]	0.05 [0.03-0.07]	25	7.31 [4.26-12.81]	5.64 [3.69-8.16]	-0.96 [-1.1 to -0.81]
Sao Tome and Principe	Male	0.01 [0.01-0.01]	0.02 [0.01-0.03]	100	2.57 [1.7-3.65]	3 [2.02-4.23]	0.66 [0.57 to 0.74]
Saudi Arabia	Male	2.8 [1.66-4.23]	10.35 [5.98-15.73]	269.64	4.04 [2.5-6.08]	4.27 [2.62-6.29]	0.27 [0.24 to 0.3]
Senegal	Male	0.64 [0.42-0.91]	1.48 [0.99-2.12]	131.25	2.95 [1.96-4.23]	3.19 [2.11-4.54]	0.42 [0.34 to 0.51]
Serbia	Male	2.59 [1.75-3.68]	2.6 [1.75-3.53]	0.39	5 [3.38-7.01]	4.37 [3-6.03]	0.86 [0.44 to 1.29]
Seychelles	Male	0.03 [0.01-0.04]	0.09 [0.04-0.13]	200	11.35 [5.38-16.65]	14.74 [6.18-23.61]	0.61 [0.33 to 0.9]
Sierra Leone	Male	0.34 [0.23-0.49]	0.76 [0.51-1.1]	123.53	2.82 [1.88-4.03]	3.07 [2.03-4.53]	0.43 [0.36 to 0.49]
Singapore	Male	1.28 [0.89-1.8]	2.66 [1.65-3.98]	107.81	9.35 [6.72-12.76]	6.45 [4.1-9.44]	-1.01 [-1.4 to -0.61]
Slovakia	Male	2.21 [1.21-3.18]	1.73 [1.17-2.4]	-21.72	8.49 [4.62-12.15]	4.92 [3.32-6.87]	-0.93 [-1.72 to -0.13]
Slovenia	Male	0.56 [0.39-0.79]	0.58 [0.38-0.84]	3.57	5.62 [3.97-7.93]	4.03 [2.64-5.81]	-0.16 [-0.64 to 0.32]
Solomon Islands	Male	0.09 [0.05-0.15]	0.17 [0.1-0.27]	88.89	9.22 [4.79-15.47]	7.78 [4.59-11.71]	-0.55 [-0.61 to -0.49]
Somalia	Male	0.51 [0.27-1.18]	1.46 [0.75-3.67]	186.27	2.04 [1.19-3.72]	2.19 [1.22-4.39]	0.2 [0.12 to 0.28]
South Africa	Male	6.28 [4.6-8.43]	10.99 [7.92-14.53]	75	4.64 [3.45-6.18]	4.36 [3.17-5.7]	-0.26 [-0.71 to 0.19]
South Sudan	Male	0.36 [0.22-0.64]	0.51 [0.33-0.8]	41.67	1.68 [1.06-2.6]	1.66 [1.09-2.4]	-0.11 [-0.16 to -0.06]
Spain	Male	16.02 [11.41-21.75]	21.68 [14.6-30.67]	35.33	7.41 [5.31-10.11]	6.51 [4.3-9.33]	-0.38 [-0.48 to -0.28]
Sri Lanka	Male	4.21 [2.72-6.27]	7.65 [4.93-11.24]	81.71	6.14 [4.03-8.97]	6.42 [4.18-9.32]	0.14 [0.1 to 0.18]
Sudan	Male	2.83 [1.77-4.15]	6.79 [4.31-10.05]	139.93	4.13 [2.56-6.15]	4.45 [2.79-6.56]	0.36 [0.31 to 0.42]
Suriname	Male	0.17 [0.12-0.24]	0.6 [0.36-0.85]	252.94	11.62 [7.93-17.06]	19.95 [12.09-28.44]	1.89 [1.51 to 2.27]
Sweden	Male	5.82 [4.34-7.9]	5.94 [4.42-7.8]	2.06	10.05 [7.47-13.55]	7.5 [5.47-10.01]	-0.46 [-0.91 to 0]

Switzerland	Male	2.94 [1.95-4.16]	4.13 [2.69-5.93]	40.48	7.07 [4.7-10.03]	6.65 [4.27-9.59]	0.02 [-0.36 to 0.39]
Syrian Arab	Male	2.04 [1.29-3.03]	3.29 [2.05-4.94]	61.27	5.18 [3.17-7.76]	4.61 [2.89-6.87]	-0.59 [-0.7 to -0.48]
Tajikistan	Male	2.33 [1.41-3.21]	5.88 [3.49-8.45]	152.36	13.2 [8.18-17.8]	16.67 [11.09-22.89]	0.62 [0.38 to 0.85]
Thailand	Male	59.47 [28.42-90.18]	80.87 [45.23-114.89]	35.98	31.96 [14.6-48.91]	17.42 [9.7-24.77]	-3 [-3.69 to -2.32]
Timor-Leste	Male	0.34 [0.13-0.66]	0.69 [0.27-1.14]	102.94	16.62 [5.54-35.49]	15.75 [5.79-26.45]	-0.24 [-0.42 to -0.06]
Togo	Male	0.26 [0.17-0.36]	0.73 [0.49-1.04]	180.77	2.79 [1.88-3.93]	3.22 [2.15-4.61]	0.54 [0.47 to 0.61]
Tonga	Male	0.03 [0.01-0.04]	0.03 [0.02-0.05]	0	8.61 [4.58-13.37]	8.11 [4.87-11.78]	-0.27 [-0.39 to -0.15]
Trinidad and Tobago	Male	0.65 [0.5-0.96]	2.4 [1.6-3.69]	269.23	15.09 [11.43-22.89]	26.22 [17.55-40]	2.9 [2.42 to 3.38]
Tunisia	Male	1.34 [0.82-1.97]	2.81 [1.72-4.18]	109.7	4.03 [2.46-6.03]	4.31 [2.66-6.39]	0.24 [0.22 to 0.26]
Turkey	Male	12.73 [8.16-19.05]	25.22 [16.72-35.78]	98.11	5.25 [3.33-7.97]	5.46 [3.69-7.64]	0.42 [0.26 to 0.59]
Turkmenistan	Male	0.97 [0.66-1.36]	2.29 [1.58-3.16]	136.08	7.52 [5.2-10.68]	10.32 [7.07-13.86]	1.27 [1.13 to 1.41]
Uganda	Male	0.86 [0.53-1.42]	1.96 [1.25-3.09]	127.91	1.59 [1.04-2.38]	1.65 [1.09-2.35]	-0.02 [-0.14 to 0.09]
Ukraine	Male	92.18 [67.46-125.27]	83.98 [63.55-110.17]	-8.9	33.32 [24.52-45.46]	30.27 [22.87-39.49]	-0.93 [-1.19 to -0.66]
United Arab Emirates	Male	0.52 [0.3-0.83]	4.63 [2.59-7.43]	790.38	4.24 [2.65-6.43]	4.6 [2.88-6.81]	0.41 [0.36 to 0.45]
United Kingdom	Male	33.79 [25.13-43.95]	46.45 [35.14-59.88]	37.47	10.01 [7.42-13.05]	9.95 [7.41-12.88]	0.46 [0.2 to 0.73]
United Republic of Tanzania	Male	1.2 [0.8-1.78]	2.7 [1.78-3.95]	125	1.43 [0.95-2.01]	1.45 [0.98-2.06]	0.09 [0.06 to 0.13]
United States of America	Male	103.28 [74.2-137.9]	101.77 [79.52-128.67]	-1.46	7.74 [5.56-10.38]	4.61 [3.56-5.8]	-1.73 [-2.08 to -1.39]
United States Virgin Islands	Male	0.04 [0.03-0.08]	0.15 [0.1-0.21]	275	9.5 [6.03-18.51]	19.35 [12.9-27.34]	3.29 [2.87 to 3.72]
Uruguay	Male	1.02 [0.67-1.47]	1.37 [0.92-1.94]	34.31	6.45 [4.2-9.42]	7.12 [4.75-10.19]	0.49 [0.38 to 0.6]
Uzbekistan	Male	4.46 [2.66-6.77]	9.6 [5.9-14.36]	115.25	6.03 [3.7-8.96]	6.43 [4.07-9.43]	0.28 [0.23 to 0.33]
Vanuatu	Male	0.03 [0.02-0.05]	0.08 [0.05-0.12]	166.67	7.44 [3.98-12.6]	6.99 [4.09-11.55]	-0.22 [-0.33 to -0.11]
Venezuela (Bolivarian Republic of)	Male	4.07 [3.01-6.22]	12.3 [8.41-16.58]	202.21	6.65 [4.9-10.56]	8.63 [5.9-11.51]	0.24 [-0.45 to 0.93]
Viet Nam	Male	20.91 [10.96-31.23]	58.8 [34.99-86.26]	181.21	10.36 [5.23-15.72]	11.61 [6.76-16.91]	0.59 [0.28 to 0.91]
Yemen	Male	1.69 [1.04-2.49]	5.05 [3.19-7.5]	198.82	4.07 [2.48-6.06]	4.45 [2.83-6.48]	0.39 [0.34 to 0.44]
Zambia	Male	0.5 [0.3-0.99]	1.07 [0.69-1.71]	114	1.86 [1.18-3.03]	1.81 [1.2-2.74]	-0.19 [-0.4 to 0.01]
Zimbabwe	Male	1.87 [1-2.9]	3.57 [1.8-6.08]	90.91	4.97 [3.17-7]	6.19 [3.75-9.2]	0.79 [0.33 to 1.24]
Afghanistan	Female	1.57 [0.98-2.59]	5.03 [3.19-7.64]	220.38	3.66 [2.27-6.13]	4.67 [3.06-6.94]	0.97 [0.85 to 1.09]
Albania	Female	0.57 [0.36-0.82]	0.61 [0.38-0.88]	7.02	3.95 [2.54-5.64]	3.82 [2.47-5.43]	-0.09 [-0.16 to -0.02]

Algeria	Female	2.46 [1.56-3.56]	6.22 [3.98-9.14]	152.85	2.85 [1.77-4.24]	3.69 [2.39-5.44]	0.31 [0.25 to 0.37]
American Samoa	Female	0.01 [0-0.01]	0.01 [0.01-0.01]	0	6.36 [3.03-9.62]	4.93 [3.51-6.6]	-3.52 [-4.22 to -2.82]
Andorra	Female	0.01 [0.01-0.02]	0.03 [0.02-0.04]	200	5.4 [3.07-7.94]	6.36 [4.18-8.73]	-0.33 [-0.53 to -0.13]
Angola	Female	1.36 [0.67-3.06]	2.73 [1.72-4.22]	100.74	3.86 [2.18-7.19]	4.24 [2.79-6.6]	-1.28 [-1.46 to -1.1]
Antigua and Barbuda	Female	0.01 [0.01-0.02]	0.04 [0.03-0.05]	300	4.13 [3.04-5.43]	6.04 [4.61-7.76]	1.68 [1.49 to 1.86]
Argentina	Female	5.86 [3.71-8.8]	10.12 [6.57-14.62]	72.7	3.56 [2.24-5.37]	5.22 [3.42-7.58]	0.46 [0.4 to 0.53]
Armenia	Female	2.37 [1.68-3.17]	7.45 [3.92-17.12]	214.35	15.16 [10.79-20.28]	33.33 [21.71-61.27]	3.81 [3.23 to 4.39]
Australia	Female	5.27 [4.14-6.43]	6.46 [4.95-8.3]	22.58	5.2 [4.1-6.37]	5.01 [3.64-6.62]	-1.15 [-1.44 to -0.86]
Austria	Female	8.07 [6.39-10.27]	3.72 [2.45-5.29]	-53.9	12.19 [9.54-15.9]	7.92 [5.03-12.18]	-2.16 [-2.68 to -1.64]
Azerbaijan	Female	1.27 [0.84-1.81]	2.61 [1.66-3.77]	105.51	3.8 [2.49-5.47]	6.15 [4-8.69]	1.46 [1.18 to 1.74]
Bahamas	Female	0.04 [0.03-0.06]	0.12 [0.08-0.15]	200	3.97 [2.98-5.22]	6.22 [4.7-8.11]	0.98 [0.79 to 1.17]
Bahrain	Female	0.04 [0.03-0.06]	0.18 [0.11-0.28]	350	2.9 [1.88-4.24]	4.2 [2.65-6.3]	0.33 [-0.04 to 0.71]
Bangladesh	Female	21.35 [12.54-31.14]	32.95 [20.89-52.08]	54.33	5.83 [3.57-8.57]	5.49 [3.73-7.72]	-1.15 [-1.25 to -1.04]
Barbados	Female	0.06 [0.04-0.07]	0.12 [0.08-0.15]	100	4.08 [3-5.34]	7.79 [5.75-9.91]	1.38 [1.21 to 1.55]
Belarus	Female	14.33 [11.28-19.2]	13.14 [8.36-18.87]	-8.3	19.34 [15.12-25.7]	20.03 [14.5-26.28]	-1.08 [-1.28 to -0.88]
Belgium	Female	2.47 [1.81-3.32]	4.54 [3.09-7.88]	83.81	3.67 [2.68-4.99]	6.03 [4.14-8.27]	1.71 [1.17 to 2.25]
Belize	Female	0.03 [0.02-0.03]	0.14 [0.1-0.18]	366.67	4.69 [3.38-6.27]	8.56 [6.71-10.6]	1.79 [1.27 to 2.31]
Benin	Female	0.62 [0.36-0.97]	1.2 [0.81-1.72]	93.55	4.55 [2.51-7.43]	3.15 [2.25-4.25]	-1.42 [-1.51 to -1.33]
Bermuda	Female	0.01 [0.01-0.02]	0.03 [0.02-0.03]	200	4.25 [3.21-5.55]	5 [3.72-6.59]	0.38 [0.12 to 0.64]
Bhutan	Female	0.14 [0.09-0.23]	0.19 [0.12-0.34]	35.71	7.92 [4.59-14.37]	6.91 [4.63-10.56]	-1.12 [-1.2 to -1.04]
Bolivia (Plurinational State of)	Female	1.54 [0.94-2.25]	3.34 [2.06-4.71]	116.88	6.93 [4.21-10.17]	7.8 [5.25-10.64]	-0.18 [-0.31 to -0.05]
Bosnia and Herzegovina	Female	1.32 [0.92-1.84]	0.89 [0.56-1.34]	-32.58	5.53 [3.84-7.66]	4.03 [2.67-5.6]	-1.48 [-1.8 to -1.16]
Botswana	Female	0.11 [0.06-0.16]	0.28 [0.15-0.44]	154.55	2.3 [1.24-3.45]	3.45 [2.29-4.79]	0.43 [0.27 to 0.59]
Brazil	Female	29.57 [22.9-37.89]	113.63 [87.57-175.75]	284.27	4.78 [3.71-6.04]	8.65 [6.91-12.33]	2.79 [2.61 to 2.98]
Brunei Darussalam	Female	0.04 [0.03-0.06]	0.1 [0.08-0.14]	150	7.22 [4.28-10.63]	8.48 [6.47-11]	-0.18 [-0.41 to 0.05]
Bulgaria	Female	14.75 [10.37-24.94]	2.36 [1.55-3.4]	-84	23.5 [16.45-39.49]	4.64 [3.27-6.4]	-5.58 [-6.71 to -4.43]
Burkina Faso	Female	1.39 [0.8-2.23]	3 [1.72-4.94]	115.83	5.09 [2.7-8.35]	3.95 [2.61-5.76]	-0.5 [-0.63 to -0.36]
Burundi	Female	1.42 [0.53-2.97]	2.03 [0.95-3.78]	42.96	8.71 [3.15-17.77]	4.34 [2.24-7.26]	-1.22 [-1.4 to -1.04]

Cabo Verde	Female	0.02 [0.02-0.03]	0.05 [0.03-0.07]	150	1.83 [1.24-2.59]	2.28 [1.5-3.22]	-0.08 [-0.16 to -0.01]
Cambodia	Female	4.28 [0.85-7.08]	6.46 [1.7-11.64]	50.93	12.37 [2.53-20.35]	13.79 [4.77-20.66]	-1.59 [-1.77 to -1.4]
Cameroon	Female	1.56 [0.78-2.74]	3.02 [1.89-4.9]	93.59	5.5 [2.54-9.58]	3.34 [2.26-4.7]	-1.99 [-2.15 to -1.83]
Canada	Female	6.25 [4.53-8.32]	12.54 [9.67-16.25]	100.64	3.8 [2.75-5.07]	4.81 [3.68-6.16]	0.95 [0.71 to 1.18]
Central African Republic	Female	0.47 [0.21-1.07]	0.93 [0.44-2.22]	97.87	5.12 [2.39-10.35]	7.11 [3.88-12.64]	0.34 [0.1 to 0.58]
Chad	Female	0.89 [0.51-1.45]	1.48 [0.97-2.2]	66.29	4.93 [2.71-8.25]	3.36 [2.36-4.64]	-1.4 [-1.5 to -1.29]
Chile	Female	3.36 [2.41-4.56]	5.5 [3.87-7.51]	63.69	5.41 [3.89-7.38]	6.11 [4.31-8.37]	-1.36 [-1.66 to -1.06]
China	Female	569.84 [292.65-746.84]	326.84 [246.13-415.03]	-42.64	11.94 [6.16-15.51]	5.27 [4.09-6.66]	-5.17 [-5.48 to -4.86]
Colombia	Female	3.77 [2.7-5.07]	11.12 [8.02-14.88]	194.96	3.09 [2.21-4.14]	3.97 [2.97-5.28]	1.5 [1.08 to 1.92]
Comoros	Female	0.11 [0.04-0.22]	0.22 [0.1-0.43]	100	7.94 [3.14-15.7]	4.78 [2.52-8.55]	-0.38 [-0.54 to -0.22]
Congo	Female	0.32 [0.18-0.58]	0.71 [0.43-1.25]	121.87	4.15 [2.34-7.22]	4.47 [2.92-6.73]	-0.43 [-0.58 to -0.28]
Costa Rica	Female	0.21 [0.13-0.3]	0.65 [0.44-0.91]	209.52	1.86 [1.16-2.71]	2.62 [1.81-3.62]	1.34 [1.05 to 1.63]
Cote d'Ivoire	Female	1.22 [0.72-1.94]	2.44 [1.57-3.74]	100	4.37 [2.26-7.18]	3.2 [2.22-4.41]	-1.16 [-1.25 to -1.08]
Croatia	Female	1.52 [1.11-1.99]	1.94 [1.33-3.03]	27.63	4.76 [3.41-6.37]	5.45 [3.85-7.46]	0.91 [0.62 to 1.21]
Cuba	Female	3.21 [2.55-3.98]	5.99 [4.31-8.96]	86.6	6.01 [4.76-7.45]	9.36 [7.09-12.33]	1.08 [0.89 to 1.28]
Cyprus	Female	0.14 [0.09-0.21]	0.28 [0.19-0.46]	100	3.35 [2.23-5.37]	4.26 [2.86-6.06]	-0.73 [-1.27 to -0.19]
Czechia	Female	15.54 [11.12-28.93]	4.55 [3.35-5.98]	-70.72	20.1 [14.42-36.14]	5.39 [3.97-7.13]	-3.79 [-4.54 to -3.03]
Democratic People's Republic of Korea	Female	8.02 [3.71-12.62]	10.12 [5.3-16.3]	26.18	7.59 [3.48-11.97]	8.82 [4.88-12.65]	-1.02 [-1.15 to -0.88]
Democratic Republic of the Congo	Female	4.36 [2.34-7.91]	8.82 [5.49-13.99]	102.29	3.33 [1.88-5.56]	4.17 [2.57-6.4]	-0.02 [-0.19 to 0.15]
Denmark	Female	1.75 [1.41-2.2]	2.01 [1.52-2.8]	14.86	4.56 [3.61-5.69]	6.16 [4.71-7.94]	-0.29 [-0.5 to -0.09]
Djibouti	Female	0.08 [0.04-0.14]	0.29 [0.15-0.56]	262.5	7.63 [4.02-13.25]	4.88 [2.78-8.67]	0.09 [-0.06 to 0.25]
Dominica	Female	0.01 [0.01-0.01]	0.01 [0.01-0.02]	0	2.69 [1.85-3.83]	4.61 [3.34-6.18]	0.73 [0.68 to 0.77]
Dominican Republic	Female	0.66 [0.45-0.93]	1.28 [0.84-1.84]	93.94	2.42 [1.61-3.42]	3.67 [2.49-5.13]	0.04 [-0.04 to 0.13]
Ecuador	Female	1.67 [1.16-2.31]	5.12 [3.62-7.13]	206.59	4.53 [3.15-6.3]	7.39 [5.36-9.68]	1.53 [1.15 to 1.91]
Egypt	Female	5.55 [3.49-8]	11.72 [7.35-17.26]	111.17	2.67 [1.66-3.87]	3.63 [2.32-5.26]	0.23 [0.19 to 0.27]
El Salvador	Female	0.38 [0.25-0.55]	0.7 [0.47-1.03]	84.21	1.99 [1.31-2.9]	2.57 [1.75-3.56]	0.19 [0.15 to 0.23]
Equatorial Guinea	Female	0.06 [0.03-0.13]	0.12 [0.07-0.21]	100	4.09 [2.19-7.73]	3.74 [2.41-5.7]	-0.94 [-1.16 to -0.71]

Eritrea	Female	0.7 [0.34-1.37]	1.61 [0.8-3.07]	130	8.34 [3.92-16.09]	5.57 [3.13-9.54]	0.11 [0.03 to 0.2]
Estonia	Female	2.38 [1.62-4.07]	1.18 [0.83-1.57]	-50.42	18.96 [13.06-31.48]	13.79 [9.78-18.63]	-3 [-3.41 to -2.59]
Eswatini	Female	0.08 [0.04-0.12]	0.12 [0.06-0.17]	50	2.82 [1.36-4.2]	3.9 [2.61-5.33]	-0.24 [-0.72 to 0.24]
Ethiopia	Female	23.18 [13.33-43.6]	20.94 [12.24-32.23]	-9.66	16.92 [10.2-30.01]	4.81 [2.97-7.06]	-3.06 [-3.23 to -2.9]
Fiji	Female	0.06 [0.04-0.09]	0.13 [0.09-0.17]	116.67	2.31 [1.5-3.43]	3.97 [2.78-5.46]	1.15 [0.86 to 1.44]
Finland	Female	1.23 [0.83-2.01]	1.12 [0.76-1.5]	-8.94	3.26 [2.23-4.97]	4.06 [2.78-5.71]	-1.48 [-2.13 to -0.84]
France	Female	14.12 [10.55-18.11]	17.39 [13.13-22.81]	23.16	3.58 [2.63-4.71]	4.8 [3.32-6.66]	-0.31 [-0.4 to -0.22]
Gabon	Female	0.16 [0.05-0.34]	0.26 [0.13-0.52]	62.5	4.54 [1.39-9.28]	4.78 [2.78-7.79]	-0.74 [-0.97 to -0.51]
Gambia	Female	0.1 [0.06-0.16]	0.24 [0.15-0.37]	140	4.17 [2.33-7.37]	3.4 [2.31-4.71]	-0.87 [-1.02 to -0.73]
Germany	Female	25.89 [19.51-32.96]	24.23 [18.04-33.11]	-6.41	4.28 [3.23-5.44]	4.7 [3.27-6.48]	0.52 [0.12 to 0.92]
Ghana	Female	2.03 [1.07-3.56]	4.12 [2.46-6.32]	102.96	4.37 [2.33-7.65]	8.35 [2.85-13.29]	-0.84 [-0.98 to -0.7]
Greece	Female	1.54 [1-2.17]	1.75 [1.11-2.5]	13.64	2.37 [1.54-3.37]	3.99 [2.55-5.78]	0.07 [-0.01 to 0.14]
Greenland	Female	0.01 [0-0.01]	0.01 [0.01-0.01]	0	2.67 [1.79-3.8]	3.08 [2.01-4.43]	0.04 [0.01 to 0.08]
Grenada	Female	0.02 [0.01-0.02]	0.04 [0.03-0.05]	100	4.34 [3.34-5.7]	12.71 [9.73-16]	2.02 [1.85 to 2.2]
Guam	Female	0.04 [0.01-0.06]	0.02 [0.01-0.03]	-50	9.44 [2.94-14.17]	3.43 [2.22-4.89]	-5.68 [-6.7 to -4.64]
Guatemala	Female	1.98 [1.36-2.76]	3.72 [2.65-5.32]	87.88	7.08 [4.95-9.38]	4.97 [3.75-6.57]	-1.32 [-1.67 to -0.97]
Guinea	Female	0.99 [0.5-1.76]	1.38 [0.86-2.21]	39.39	4.89 [2.44-8.68]	3.44 [2.31-4.94]	-1.16 [-1.26 to -1.06]
Guinea-Bissau	Female	0.2 [0.1-0.36]	0.24 [0.15-0.39]	20	6.9 [3.25-12.17]	3.93 [2.6-5.5]	-1.84 [-1.92 to -1.75]
Guyana	Female	0.15 [0.11-0.2]	0.34 [0.24-0.47]	126.67	5.48 [4.11-7.34]	11.6 [8.57-15.11]	1.57 [1.12 to 2.03]
Haiti	Female	2 [1.02-3.2]	4.54 [2.39-6.98]	127	8.44 [4.46-12.51]	11.3 [6.17-19.01]	0.5 [0.34 to 0.66]
Honduras	Female	0.84 [0.42-1.22]	1.81 [1.07-2.98]	115.48	5.48 [2.8-7.65]	6.93 [3.98-10.48]	-0.63 [-0.74 to -0.52]
Hungary	Female	23.09 [19.36-31.57]	8.58 [5.35-11.32]	-62.84	28.21 [23.62-38.49]	9.07 [6.76-11.68]	-3.49 [-4.21 to -2.76]
Iceland	Female	0.06 [0.05-0.08]	0.07 [0.06-0.09]	16.67	4.48 [3.51-5.56]	5.14 [3.68-6.85]	-1.1 [-1.2 to -1]
India	Female	301.5 [170.86-411.06]	445.88 [300.68-621.39]	47.89	10.27 [5.57-14.62]	8.58 [6.17-11.43]	-1.63 [-1.89 to -1.37]
Indonesia	Female	47.69 [14.38-80.66]	64.07 [20.9-113.36]	34.35	6.91 [2.11-11.28]	13.35 [4.15-19.56]	-1.08 [-1.24 to -0.91]
Iran (Islamic Republic of)	Female	6.19 [4.19-8.67]	15.45 [10.29-21.42]	149.6	3.22 [2.16-4.48]	4.38 [2.97-6.03]	0.36 [0.13 to 0.59]
Iraq	Female	1.84 [1.2-2.71]	4.91 [3.21-7.06]	166.85	3.3 [2.13-4.77]	4.07 [2.82-5.62]	-0.56 [-0.64 to -0.48]
Ireland	Female	0.61 [0.45-0.82]	1.18 [0.86-1.56]	93.44	3.12 [2.23-4.22]	4.91 [3.41-6.65]	0.38 [0.28 to 0.49]
Israel	Female	0.72 [0.52-0.99]	2.2 [1.63-2.98]	205.56	2.95 [2.1-4.09]	5.78 [4.15-7.7]	0.31 [-0.24 to 0.87]

Italy	Female	22.46 [17.2-27.86]	17.94 [13.49-23.7]	-20.12	5.53 [4.3-6.92]	5.18 [3.64-7]	-1.39 [-1.65 to -1.13]
Jamaica	Female	0.22 [0.14-0.3]	0.53 [0.37-0.77]	140.91	2.24 [1.47-3.21]	5.49 [4.02-7.43]	2.14 [1.76 to 2.53]
Japan	Female	28.28 [20.2-37.95]	68.55 [50.8-85.76]	142.4	3.31 [2.39-4.49]	5.97 [4.46-7.79]	1.01 [0.85 to 1.18]
Jordan	Female	0.36 [0.24-0.51]	1.81 [1.05-2.95]	402.78	3.19 [2.09-4.54]	5.19 [3.12-8.36]	1.15 [0.94 to 1.36]
Kazakhstan	Female	12.93 [7.79-20.56]	18.72 [13.23-24.11]	44.78	16.34 [9.85-25.93]	22.57 [17.82-28.08]	-0.18 [-0.55 to 0.2]
Kenya	Female	3.73 [2.43-5.83]	11.27 [6.87-19.53]	202.14	6.87 [4.39-10.8]	5 [3.18-8.12]	0.51 [0.43 to 0.6]
Kuwait	Female	0.14 [0.09-0.21]	0.62 [0.38-0.94]	342.86	2.7 [1.7-3.98]	3.7 [2.42-5.4]	0.25 [0.19 to 0.31]
Kyrgyzstan	Female	1.53 [1.19-2.05]	1.61 [1.15-2.19]	5.23	7.88 [6.1-10.49]	9.15 [6.89-11.96]	-1.55 [-2.14 to -0.95]
Lao People's Democratic Republic)	Female	2.12 [0.35-3.99]	2.14 [0.6-3.55]	0.94	14.69 [2.6-26.12]	12.56 [4.37-20.15]	-2.54 [-2.75 to -2.33]
Latvia	Female	5.28 [3.69-8.4]	3.18 [2.34-4.28]	-39.77	24.09 [17.13-37.44]	20.47 [15.73-25.88]	-2.34 [-2.65 to -2.03]
Lebanon	Female	0.37 [0.23-0.54]	0.82 [0.53-1.19]	121.62	2.67 [1.66-3.93]	3.54 [2.24-5.12]	0.31 [0.26 to 0.36]
Lesotho	Female	0.16 [0.09-0.25]	0.29 [0.13-0.45]	81.25	2.34 [1.27-3.7]	4.56 [2.93-6.37]	1.93 [1.6 to 2.27]
Liberia	Female	0.33 [0.17-0.54]	0.55 [0.36-0.83]	66.67	5.43 [2.77-8.74]	3.28 [2.29-4.53]	-1.7 [-1.92 to -1.48]
Libya	Female	0.34 [0.22-0.49]	1.06 [0.69-1.54]	211.76	2.74 [1.71-4.04]	3.76 [2.46-5.32]	0.53 [0.44 to 0.62]
Lithuania	Female	6.23 [5.04-8.19]	4.01 [3.08-5.25]	-35.63	24.16 [19.59-31.08]	17.2 [12.95-22.47]	-2.57 [-2.86 to -2.28]
Luxembourg	Female	0.09 [0.07-0.12]	0.13 [0.09-0.17]	44.44	3.46 [2.48-4.64]	5.01 [3.45-7.01]	-0.33 [-0.87 to 0.21]
Madagascar	Female	2.83 [1.35-5.61]	5.49 [2.82-10.4]	93.99	8.16 [4.1-14.81]	4.46 [2.57-7.56]	-0.57 [-0.64 to -0.5]
Malawi	Female	2.45 [1.14-4.93]	3.47 [1.93-6.26]	41.63	9.15 [4.56-16.84]	4.83 [2.84-8.01]	-1.08 [-1.22 to -0.95]
Malaysia	Female	1.83 [1.17-2.59]	5.36 [2.83-11.59]	192.9	3.34 [1.99-4.89]	5.7 [3.54-9.28]	0.6 [0.39 to 0.81]
Maldives	Female	0.01 [0.01-0.02]	0.04 [0.02-0.05]	300	2.38 [1.45-3.49]	4.69 [3.1-6.74]	-0.73 [-0.88 to -0.57]
Mali	Female	1.72 [0.83-3.09]	2.44 [1.53-4.09]	41.86	6.48 [3.09-10.65]	3.52 [2.46-4.77]	-1.8 [-1.94 to -1.66]
Malta	Female	0.12 [0.09-0.15]	0.15 [0.11-0.2]	25	5.22 [3.97-6.66]	6.02 [4.34-8.03]	0.15 [-0.11 to 0.42]
Marshall Islands	Female	0.01 [0-0.02]	0.02 [0.01-0.03]	100	9.31 [3.93-16.59]	7.83 [4.57-11.91]	-0.64 [-0.9 to -0.39]
Mauritania	Female	0.42 [0.21-0.72]	0.5 [0.31-0.78]	19.05	7.11 [3.31-12.1]	3.35 [2.3-4.77]	-2.38 [-2.48 to -2.27]
Mauritius	Female	0.11 [0.08-0.15]	0.18 [0.11-0.25]	63.64	2.24 [1.59-3]	4.12 [2.7-5.85]	-0.32 [-0.59 to -0.05]
Mexico	Female	27.23 [22.41-32.79]	70.4 [52.52-106.64]	158.54	9.62 [7.94-11.57]	11.41 [9.09-15.51]	0.55 [0.17 to 0.93]
Micronesia (Federated States of)	Female	0.04 [0.01-0.06]	0.04 [0.02-0.07]	0	11.68 [4.46-19.26]	7.98 [4.61-12.33]	-1.34 [-1.52 to -1.16]

Mongolia	Female	0.63 [0.32-1.11]	0.79 [0.51-1.12]	25.4	9.32 [4.47-17.58]	5.66 [3.79-7.87]	-3.56 [-4.13 to -2.98]
Montenegro	Female	0.12 [0.08-0.18]	0.14 [0.09-0.21]	16.67	3.62 [2.3-5.29]	3.63 [2.36-5.24]	0.01 [-0.02 to 0.03]
Morocco	Female	2.65 [1.69-3.85]	5.81 [3.79-8.3]	119.25	2.78 [1.74-4.09]	3.79 [2.47-5.41]	0.59 [0.53 to 0.65]
Mozambique	Female	3.89 [2.03-7.22]	6.81 [3.39-13.2]	75.06	9.58 [4.73-16.71]	5.93 [3.21-10.56]	-0.21 [-0.39 to -0.03]
Myanmar	Female	20.83 [4.09-37.16]	21.24 [6.33-41.57]	1.97	13.31 [2.71-22.78]	12.89 [4.63-20.46]	-2.26 [-2.59 to -1.92]
Namibia	Female	0.13 [0.07-0.2]	0.21 [0.13-0.31]	61.54	2.47 [1.28-3.85]	3.09 [2.17-4.16]	-0.8 [-0.96 to -0.65]
Nepal	Female	5.27 [3.26-7.59]	8.7 [5.51-13.87]	65.09	7.99 [4.87-12.5]	7.16 [4.85-10.59]	-0.76 [-1.08 to -0.44]
Netherlands	Female	8.26 [6.7-10.75]	6.93 [5.27-9.74]	-16.1	7.45 [6.07-9.39]	6.21 [4.59-8.04]	-1.95 [-2.21 to -1.69]
New Zealand	Female	1.96 [1.56-2.66]	2.28 [1.88-2.84]	16.33	9.92 [7.89-13.19]	7.3 [5.82-9.03]	-0.9 [-1.39 to -0.41]
Nicaragua	Female	0.25 [0.17-0.35]	0.94 [0.53-1.42]	276	2.16 [1.44-3.07]	3.68 [2.34-5.01]	1.69 [1.39 to 1.99]
Niger	Female	1.3 [0.61-2.71]	2.15 [1.42-3.17]	65.38	5.74 [2.96-10.1]	3.3 [2.22-4.49]	-2.07 [-2.23 to -1.91]
Nigeria	Female	11.17 [6.59-17.29]	20.22 [14.03-28.52]	81.02	4.11 [2.38-6.34]	2.98 [2.13-4]	-1.54 [-1.66 to -1.43]
North Macedonia	Female	0.39 [0.25-0.56]	0.48 [0.29-0.71]	23.08	3.71 [2.42-5.36]	3.67 [2.35-5.27]	-0.05 [-0.1 to 0]
Northern Mariana Islands	Female	0.01 [0.01-0.01]	0.01 [0.01-0.01]	0	6.4 [3.83-10.14]	3.78 [2.56-5.44]	-2.28 [-3.02 to -1.53]
Norway	Female	2.41 [1.95-3.11]	2.12 [1.57-2.91]	-12.03	7.16 [5.67-9.16]	8.64 [6.1-11.64]	-0.77 [-0.97 to -0.57]
Oman	Female	0.14 [0.09-0.2]	0.41 [0.27-0.6]	192.86	2.86 [1.83-4.21]	4.18 [2.72-6.04]	0.71 [0.6 to 0.82]
Pakistan	Female	29.54 [17.45-42.35]	62.59 [38.4-91.4]	111.88	8.32 [5.06-12.15]	9.34 [6.61-13.24]	-0.24 [-0.4 to -0.08]
Palestine	Female	0.25 [0.16-0.37]	0.56 [0.37-0.8]	124	3.52 [2.27-5.19]	3.88 [2.55-5.55]	-0.44 [-0.61 to -0.26]
Panama	Female	0.19 [0.13-0.26]	0.45 [0.3-0.63]	136.84	2.07 [1.39-2.91]	2.49 [1.69-3.48]	0.17 [-0.16 to 0.49]
Papua New Guinea	Female	1.12 [0.26-2.08]	2.34 [0.69-4.19]	108.93	8.61 [2.01-15.58]	6.42 [3.61-9.62]	-0.89 [-0.95 to -0.84]
Paraguay	Female	0.42 [0.29-0.6]	1.48 [0.78-2.12]	252.38	2.81 [1.94-4]	4.76 [2.78-6.69]	2.47 [2.07 to 2.88]
Peru	Female	3.47 [2.27-4.95]	7.4 [4.71-10.67]	113.26	4.31 [2.82-6.13]	5.53 [3.7-7.76]	-0.09 [-0.19 to 0.02]
Philippines	Female	29.14 [16.85-37.03]	59.19 [40.58-79.92]	103.12	14.79 [8.82-19.54]	22.66 [18.14-29.72]	-0.48 [-0.56 to -0.4]
Poland	Female	32.59 [26.05-44.03]	9.1 [6.7-12.03]	-72.08	13.86 [11.09-18.54]	3.33 [2.49-4.41]	-5.39 [-5.86 to -4.91]
Portugal	Female	1.9 [1.25-2.59]	3.95 [2.51-7.05]	107.89	2.93 [1.95-4]	4.87 [3.37-6.84]	1.65 [1.26 to 2.05]
Puerto Rico	Female	0.43 [0.28-0.62]	0.71 [0.49-0.96]	65.12	2.25 [1.49-3.25]	3.65 [2.51-5.17]	0.38 [0.12 to 0.65]
Qatar	Female	0.03 [0.02-0.04]	0.19 [0.12-0.29]	533.33	2.94 [1.89-4.36]	4 [2.58-5.95]	0.51 [0.38 to 0.64]
Republic of Korea	Female	5.88 [3.98-8.44]	13.31 [8.82-18.9]	126.36	3.04 [2.09-4.47]	4.99 [3.41-7.02]	0 [-0.15 to 0.14]
Republic of Moldova	Female	3.52 [2.58-4.7]	4.04 [2.86-5.24]	14.77	13.61 [10-18.27]	17.04 [12.47-22.23]	0.42 [0 to 0.83]

Romania	Female	5.57 [3.64-7.98]	5.2 [3.34-7.52]	-6.64	4.2 [2.77-6.04]	3.92 [2.55-5.52]	1.61 [1.06 to 2.17]
Russian Federation	Female	271.57 [224.81-337.31]	256.04 [197.35-317.17]	-5.72	25.34 [20.81-31.42]	24.65 [19.66-30.59]	-1.03 [-1.4 to -0.65]
Rwanda	Female	1.93 [0.4-4.17]	2.52 [0.92-5.2]	30.57	9.79 [1.7-20.48]	4.48 [1.69-9.06]	-2.21 [-2.5 to -1.91]
Saint Lucia	Female	0.04 [0.03-0.05]	0.12 [0.09-0.15]	200	7.58 [6.06-9.56]	7.97 [6.14-10.18]	1.15 [0.94 to 1.35]
Saint Vincent and the Grenadines	Female	0.01 [0.01-0.01]	0.02 [0.01-0.02]	100	2.26 [1.49-3.21]	7.75 [5.76-9.87]	0.3 [0.27 to 0.33]
Samoa	Female	0.05 [0.02-0.08]	0.05 [0.03-0.09]	0	8.98 [3.55-14.89]	6.14 [3.82-8.74]	-1.45 [-1.59 to -1.3]
Sao Tome and Principe	Female	0.02 [0.01-0.03]	0.03 [0.02-0.06]	50	4.76 [2.6-7.26]	3.63 [2.25-5.69]	-0.84 [-1.06 to -0.62]
Saudi Arabia	Female	1.15 [0.72-1.69]	4.06 [2.47-6.15]	253.04	2.7 [1.68-3.93]	3.69 [2.36-5.35]	0.22 [0.19 to 0.25]
Senegal	Female	1.07 [0.58-1.75]	1.63 [1.06-2.41]	52.34	5.21 [2.62-8.43]	3.24 [2.28-4.5]	-1.85 [-2 to -1.7]
Serbia	Female	2.6 [1.77-3.64]	2.81 [1.95-3.85]	8.08	4.72 [3.23-6.62]	4.48 [3.14-6.03]	0.84 [0.51 to 1.17]
Seychelles	Female	0.01 [0-0.01]	0.03 [0.01-0.05]	200	2.95 [1.57-4.22]	9.73 [4.17-14.71]	1.84 [1.58 to 2.1]
Sierra Leone	Female	0.49 [0.28-0.78]	0.86 [0.56-1.28]	75.51	4.21 [2.34-6.7]	3.2 [2.22-4.46]	-0.8 [-0.87 to -0.73]
Singapore	Female	0.62 [0.46-0.84]	0.9 [0.57-1.32]	45.16	4.57 [3.44-6.39]	4.48 [2.9-6.47]	-1.71 [-2.05 to -1.38]
Slovakia	Female	2.92 [1.97-3.99]	2.13 [1.49-3.09]	-27.05	9.27 [6.32-12.7]	5.2 [3.67-7.14]	-2.09 [-2.19 to -1.98]
Slovenia	Female	0.81 [0.53-1.42]	0.58 [0.39-0.83]	-28.4	6.05 [3.98-9.99]	3.92 [2.62-5.45]	-1.66 [-1.89 to -1.44]
Solomon Islands	Female	0.11 [0.04-0.21]	0.24 [0.09-0.51]	118.18	12.41 [4.58-21.32]	9.04 [5.06-14.12]	-0.64 [-0.83 to -0.44]
Somalia	Female	2.52 [1.07-5.56]	5.95 [2.47-12.2]	136.11	12.38 [5.27-25.12]	7.56 [3.37-14.06]	-0.08 [-0.15 to -0.02]
South Africa	Female	5.28 [3.44-7.06]	7.1 [5.24-9.44]	34.47	3.22 [2.19-4.27]	3.33 [2.48-4.27]	-0.85 [-1.25 to -0.44]
South Sudan	Female	1.06 [0.51-2]	1.63 [0.88-2.97]	53.77	7.07 [3.47-12.9]	3.96 [2.33-6.54]	-0.44 [-0.51 to -0.38]
Spain	Female	10.19 [7.27-13.03]	12.98 [9.4-18.54]	27.38	3.95 [2.87-5.11]	4.88 [3.37-6.74]	-0.5 [-0.63 to -0.37]
Sri Lanka	Female	1.19 [0.77-1.73]	2.72 [1.78-3.84]	128.57	1.68 [1.08-2.42]	4.07 [2.67-5.82]	0.82 [0.57 to 1.06]
Sudan	Female	1.94 [1.26-2.83]	4.62 [3.01-6.53]	138.14	2.89 [1.81-4.3]	3.83 [2.53-5.47]	0.53 [0.45 to 0.61]
Suriname	Female	0.05 [0.04-0.08]	0.15 [0.1-0.22]	200	3.43 [2.3-4.78]	11.95 [7.71-16.16]	1.04 [0.87 to 1.2]
Sweden	Female	3.38 [2.69-4.29]	2.96 [2.18-3.82]	-12.43	4.94 [3.87-6.29]	5.43 [4.02-7.14]	-0.66 [-1.08 to -0.24]
Switzerland	Female	2.15 [1.62-2.72]	1.98 [1.38-2.71]	-7.91	4.35 [3.27-5.6]	4.81 [3.19-6.79]	-1.16 [-1.28 to -1.04]
Syrian Arab	Female	1.15 [0.76-1.62]	2.11 [1.33-3.12]	83.48	2.85 [1.88-4.02]	3.67 [2.39-5.34]	-0.12 [-0.2 to -0.04]
Tajikistan	Female	1.02 [0.64-1.44]	2.31 [1.4-3.31]	126.47	5.28 [3.36-7.59]	11.56 [7.72-15.43]	0.9 [0.8 to 0.99]
Thailand	Female	16.63 [7.41-28.92]	39.76 [15.01-80.06]	139.09	8.65 [3.42-15.66]	12.1 [6.37-18.34]	-0.75 [-1.02 to -0.48]

Timor-Leste	Female	0.22 [0.05-0.4]	0.35 [0.1-0.69]	59.09	9.89 [2.24-17.55]	11.72 [4.11-19.36]	-1.2 [-1.44 to -0.96]
Togo	Female	0.45 [0.25-0.71]	0.91 [0.59-1.36]	102.22	4.74 [2.47-7.75]	3.21 [2.22-4.47]	-1.52 [-1.66 to -1.37]
Tonga	Female	0.01 [0.01-0.02]	0.01 [0.01-0.02]	0	3.4 [2.28-4.8]	5.37 [3.56-7.5]	-0.63 [-0.76 to -0.5]
Trinidad and Tobago	Female	0.35 [0.26-0.51]	0.92 [0.62-1.42]	162.86	7.28 [5.47-10.75]	18.05 [12.95-26.14]	2.67 [1.79 to 3.56]
Tunisia	Female	0.88 [0.56-1.29]	1.94 [1.24-2.85]	120.45	2.72 [1.72-4.01]	3.59 [2.33-5.18]	0.27 [0.23 to 0.32]
Turkey	Female	9.83 [6.69-13.89]	16.93 [11.66-23.19]	72.23	4.26 [2.85-6.06]	4.55 [3.22-6.24]	-0.37 [-0.55 to -0.2]
Turkmenistan	Female	0.62 [0.41-0.87]	1.84 [1.32-2.56]	196.77	4.42 [2.88-6.21]	8.95 [6.79-11.48]	2.43 [2.23 to 2.64]
Uganda	Female	2.51 [1.06-4.59]	5.96 [2.88-10.77]	137.45	5.79 [2.17-10.51]	4.04 [2.04-6.82]	-0.28 [-0.47 to -0.08]
Ukraine	Female	87.85 [65.62-113.18]	65.03 [44.12-85.11]	-25.98	21.47 [16.03-27.62]	22.63 [17.36-28.82]	-1.38 [-1.64 to -1.12]
United Arab Emirates	Female	0.11 [0.07-0.16]	0.85 [0.53-1.3]	672.73	2.88 [1.76-4.29]	4.28 [2.71-6.17]	0.86 [0.58 to 1.14]
United Kingdom	Female	24.66 [15.45-31.09]	34.85 [28.13-46.93]	41.32	5.79 [3.89-7.33]	8.21 [6.29-10.27]	0.86 [0.61 to 1.11]
United Republic of Tanzania	Female	4.97 [2.75-9.04]	10.79 [5.83-20.36]	117.1	6.93 [3.78-12.35]	4.19 [2.45-7.31]	-0.21 [-0.36 to -0.05]
United States of America	Female	80.62 [61.8-102.81]	105.36 [84.13-134.43]	30.69	5.16 [3.88-6.67]	4.49 [3.55-5.58]	-0.61 [-0.87 to -0.34]
United States Virgin Islands	Female	0.08 [0.05-0.11]	0.12 [0.08-0.17]	50	15.19 [10.46-21.99]	16.28 [12.06-21.55]	-0.19 [-0.32 to -0.06]
Uruguay	Female	0.73 [0.5-1.03]	1.13 [0.82-1.52]	54.79	4.25 [2.89-6.06]	6.03 [4.23-8.25]	0.65 [0.53 to 0.78]
Uzbekistan	Female	2.6 [1.66-3.85]	5.57 [3.47-8.26]	114.23	3.2 [2.03-4.74]	4.84 [3.1-6.96]	0.3 [0.26 to 0.34]
Vanuatu	Female	0.03 [0.01-0.05]	0.07 [0.04-0.12]	133.33	7.68 [3.39-13.06]	7.03 [4.21-11.22]	-0.64 [-0.82 to -0.46]
Venezuela (Bolivarian Republic of)	Female	4.06 [3.27-5.37]	12.5 [8.68-17.07]	207.88	5.65 [4.51-7.48]	8.27 [6.17-10.95]	0.53 [-0.12 to 1.19]
Viet Nam	Female	14.55 [4.63-23.62]	31.07 [13.06-47.86]	113.54	5.81 [1.76-9.62]	8.52 [4.68-12.4]	0.17 [-0.24 to 0.58]
Yemen	Female	1.12 [0.73-1.7]	3.52 [2.3-5.2]	214.29	2.81 [1.77-4.32]	3.86 [2.53-5.54]	0.58 [0.5 to 0.66]
Zambia	Female	2.26 [1.19-4.56]	3.99 [2.05-8.04]	76.55	10.88 [5.81-20.72]	5.26 [2.98-9.66]	-1.13 [-1.26 to -0.99]
Zimbabwe	Female	0.52 [0.34-0.72]	0.95 [0.63-1.34]	82.69	1.61 [1.02-2.3]	3.7 [2.35-5.27]	0.3 [0.21 to 0.38]

EAPCs: Estimated annual percentage changes; DALYs: Disability-adjusted life years; CI: Confidence interval; UI: Uncertainty interval.

Supplementary Table S7. Age distribution of death rate (per 100,000) for Urolithiasis in different countries in 2019.

Supplementary Table S7. Age distribution of death rate (per 100,000) for Urolithiasis in different countries in 2019.

Country	Sex	<5	5 to 9	10 to 14	15 to 19	20 to 24	25 to 29	30 to 34	35 to 39	40 to 44	45 to 49	50 to 54	55 to 59	60 to 64	65 to 69	70 to 74	75 to 79	80 to 84	85 to 89	90 to 94	95+
Afghanistan	Male	0	0.01	0.01	0	0	0	0	0.01	0.02	0.03	0.03	0.07	0.08	0.12	0.13	0.32	0.48	0.7	1.39	1.01
Afghanistan	Female	0	0.01	0.01	0	0.01	0.01	0.02	0.04	0.09	0.07	0.08	0.11	0.13	0.25	0.34	0.58	0.81	1.09	1.18	1.11
Albania	Male	0	0	0	0	0	0	0	0.01	0	0.01	0	0.01	0.02	0.04	0.08	0.18	0.33	0.48	0.87	1.08
Albania	Female	0.01	0	0	0	0	0	0	0	0	0	0	0	0.01	0.02	0.02	0.04	0.06	0.11	0.22	0.41
Algeria	Male	0	0	0	0	0	0	0	0	0	0	0	0.01	0.02	0.02	0.03	0.09	0.16	0.35	0.74	0.5
Algeria	Female	0	0	0	0	0	0	0	0.01	0.02	0.01	0.01	0.02	0.02	0.05	0.08	0.17	0.28	0.81	1.23	0.6
American Samoa	Male	0	0	0	0	0	0	0	0	0.11	0.04	0.11	0.32	0.51	0.18	0.22	1.42	5.18	1.79	8.1	1.6
American Samoa	Female	0	0	0	0	0	0	0.01	0.03	0.04	0.01	0.03	0.11	0.03	0.28	0.33	0.61	0.12	0.26	0.47	0.61
Andorra	Male	0	0	0	0.01	0.01	0.02	0.02	0.03	0.03	0.05	0.05	0.1	0.15	0.28	0.51	1.03	1.94	3.6	6.1	9.97
Andorra	Female	0	0	0	0.01	0.02	0.01	0.02	0.03	0.03	0.04	0.09	0.15	0.24	0.38	0.7	1.2	1.84	2.52	3.99	6.83
Angola	Male	0	0	0	0.01	0	0.01	0.02	0.04	0.06	0.12	0.18	0.38	0.43	0.81	0.81	1.35	2.74	1.64	3.29	4.52
Angola	Female	0	0	0	0	0	0	0.01	0.02	0.03	0.07	0.07	0.12	0.13	0.24	0.3	0.56	0.63	0.84	1.14	2.12
Antigua and Barbuda	Male	0	0	0	0	0	0	0	0.02	0.01	0.05	0.07	0.15	0.23	0.22	0.36	0.6	0.7	0.88	1.5	1.91
Antigua and Barbuda	Female	0	0	0	0	0.01	0.01	0.04	0.08	0.08	0.2	0.22	0.45	0.53	0.79	0.75	0.95	1.13	1.83	1.62	3.65
Argentina	Male	0	0	0	0	0	0	0.01	0.01	0.01	0.01	0.03	0.02	0.1	0.07	0.13	0.23	0.64	0.69	1.15	1.53
Argentina	Female	0	0	0	0	0	0	0.01	0.01	0.03	0.04	0.03	0.03	0.04	0.08	0.1	0.14	0.2	0.38	0.58	0.38
Armenia	Male	0	0	0	0	0	0	0	0	0.16	0.7	0.73	1.06	2.39	5.25	6.25	16.32	21.64	42.44	77.39	96.93
Armenia	Female	0	0	0	0	0	0	0.08	0.1	0.18	0.31	0.52	0.59	2.7	4.37	7.76	15.94	33.92	61.64	116.3	184.44
Australia	Male	0	0	0	0	0	0.01	0.01	0.01	0.01	0.03	0.04	0.09	0.09	0.26	0.35	0.7	1.35	2.91	5.9	9.17
Australia	Female	0	0	0	0	0	0	0.01	0.01	0.02	0.04	0.04	0.1	0.15	0.28	0.41	0.65	1.12	2.12	3.36	4.66
Austria	Male	0	0	0	0	0	0	0	0	0	0.01	0.02	0.05	0.12	0.2	0.37	0.73	1.26	2.25	4.1	5.71
Austria	Female	0	0	0	0	0	0	0.01	0	0.01	0.01	0.03	0.08	0.18	0.28	0.53	0.9	1.87	2.4	3.12	5.56
Azerbaijan	Male	0	0	0	0	0	0	0	0	0	0	0.01	0.01	0.14	0.09	0.1	0.71	0.32	0.51	0.72	0.61

Azerbaijan	Female	0	0	0	0	0	0	0	0	0	0	0.01	0.01	0.03	0.06	0.13	1.51	5.55	7.45	14.18	23.86
Bahamas	Male	0	0	0	0	0	0.01	0.02	0.08	0.06	0.19	0.2	0.34	0.43	0.5	0.69	1.04	0.77	1.13	2.18	2.08
Bahamas	Female	0	0	0	0	0.01	0.01	0.07	0.12	0.07	0.16	0.14	0.25	0.26	0.39	0.31	0.4	0.49	0.71	0.57	1.13
Bahrain	Male	0	0	0	0	0	0	0	0	0	0	0	0.02	0.03	0.04	0.09	0.38	1	2.42	5.05	7.89
Bahrain	Female	0	0	0	0	0	0	0	0	0	0	0.01	0.01	0.01	0.05	0.12	0.35	0.89	1.87	3.32	7.63
Bangladesh	Male	0	0.01	0.01	0	0	0.01	0.01	0.01	0.02	0.04	0.06	0.08	0.21	0.18	0.24	0.33	1.37	1.1	2.42	2.91
Bangladesh	Female	0	0	0.01	0	0	0	0.01	0.02	0.02	0.02	0.06	0.22	0.09	0.24	0.19	0.43	0.34	1	1.91	4.16
Barbados	Male	0	0	0	0	0.01	0.01	0.03	0.08	0.07	0.27	0.27	0.59	0.8	0.97	1.59	2.47	2.17	3.07	5.15	5.12
Barbados	Female	0	0	0.01	0	0.01	0.01	0.04	0.09	0.07	0.15	0.15	0.28	0.37	0.51	0.49	0.66	0.74	1.38	1.15	2.41
Belarus	Male	0	0	0	0	0.01	0.01	0.02	0.12	0.16	0.21	0.38	0.63	1.12	2.4	3.7	5.64	7.71	8.24	12.88	20.15
Belarus	Female	0	0	0.01	0	0.01	0.02	0.03	0.12	0.06	0.27	0.29	0.79	1.32	1.83	2.51	3.05	3.93	3.45	5.55	9.03
Belgium	Male	0	0	0	0	0	0.01	0.01	0.01	0.01	0.03	0.03	0.05	0.13	0.2	0.4	0.65	1.3	2.79	5.57	16.77
Belgium	Female	0	0	0	0.01	0.01	0.01	0.01	0.02	0.03	0.02	0.04	0.09	0.16	0.27	0.53	0.98	1.87	4.04	9.07	11.19
Belize	Male	0	0	0	0	0.01	0.01	0.03	0.09	0.08	0.26	0.28	0.58	0.69	0.72	1.01	1.34	1.28	1.67	2.48	2.94
Belize	Female	0	0	0	0	0.01	0.03	0.13	0.14	0.11	0.33	0.32	0.54	0.53	0.7	0.63	0.83	0.82	1.32	1.15	2.76
Benin	Male	0	0	0	0	0	0	0	0	0	0.01	0.01	0.02	0.1	0.25	0.17	0.4	0.52	0.88	1.88	1.33
Benin	Female	0.01	0	0	0	0	0	0	0.01	0.02	0.23	0.04	0.09	0.03	0.14	0.26	1.24	0.58	0.89	0.74	9.59
Bermuda	Male	0	0	0	0	0	0	0	0.01	0.01	0.04	0.05	0.1	0.13	0.18	0.28	0.49	0.44	0.68	1.18	1.29
Bermuda	Female	0	0	0	0	0.01	0.01	0.03	0.06	0.05	0.12	0.12	0.22	0.25	0.41	0.45	0.69	1	2.08	2.16	5.62
Bhutan	Male	0	0	0	0	0	0	0	0.01	0.03	0.04	0.07	0.12	0.23	0.46	0.92	1.47	2.37	3.18	5.14	4.82
Bhutan	Female	0	0	0.01	0	0	0	0.02	0.03	0.05	0.09	0.1	0.22	0.21	0.5	0.85	1.98	1.65	2.38	2.84	4.47
Bolivia (Plurinational State of)	Male	0	0	0	0	0.01	0	0.02	0.03	0.04	0.11	0.14	0.19	0.48	0.57	1.19	1.17	1.55	2.63	5.61	6.29
Bolivia (Plurinational State of)	Female	0	0	0	0	0.01	0	0.02	0.07	0.1	0.14	0.2	0.31	0.3	0.43	0.39	0.77	1.76	0.86	3.45	2.83
Bosnia and Herzegovina	Male	0	0	0	0	0	0	0	0	0	0.01	0.03	0.03	0.04	0.09	0.33	0.48	0.39	0.42	0.68	0.64
Bosnia and Herzegovina	Female	0	0	0	0	0	0	0	0	0.02	0.01	0.01	0.02	0.03	0.06	0.1	0.18	0.3	0.75	1.56	0.76

Botswana	Male	0	0	0	0.01	0.01	0.01	0.01	0.02	0.04	0.05	0.1	0.15	0.17	0.39	0.47	0.7	0.7	0.74	3.04	1.51
Botswana	Female	0.02	0	0	0	0.01	0.01	0.02	0.03	0.03	0.05	0.06	0.09	0.07	0.12	0.16	0.22	0.36	0.52	1.07	0.81
Brazil	Male	0	0	0	0.01	0.02	0.03	0.05	0.06	0.12	0.16	0.26	0.41	0.58	0.86	1.31	1.76	2.52	2.79	4.88	5.36
Brazil	Female	0	0	0	0.02	0.04	0.07	0.1	0.13	0.19	0.29	0.34	0.49	0.6	0.71	0.98	1.49	2.19	3.64	3.86	6.83
Brunei Darussalam	Male	0	0	0	0	0	0.02	0.01	0.01	0.02	0.02	0.07	0.12	0.23	0.34	1.35	2.83	7.35	24.41	35.64	43.3
Brunei Darussalam	Female	0	0	0.01	0	0.01	0	0.01	0	0.02	0.05	0.09	0.28	0.22	0.97	1.28	2.54	4.22	8.71	12.57	10.67
Bulgaria	Male	0	0	0	0	0	0	0	0	0.02	0.04	0.05	0.1	0.15	0.3	0.35	0.54	0.7	0.83	1.8	2.13
Bulgaria	Female	0	0	0	0	0	0	0	0.01	0.02	0.05	0.07	0.1	0.13	0.19	0.27	0.34	0.42	0.55	0.77	1.15
Burkina Faso	Male	0	0	0.01	0.01	0.02	0	0	0	0	0	0.01	0.02	0.06	0.16	0.09	0.21	0.23	0.34	0.76	0.57
Burkina Faso	Female	0.01	0	0	0.01	0	0	0	0	0.02	0.49	0.06	0.09	0.02	0.12	0.29	3.69	0.68	0.99	0.92	51.9
Burundi	Male	0	0	0	0.03	0	0	0	0	0	0.01	0.01	0.02	0.03	0.07	0.08	0.16	0.35	0.29	0.66	1.44
Burundi	Female	0	0	0	0.05	0	0	0	0	0.01	1	0.02	0.05	0.01	0.03	0.09	6.7	0.21	0.49	0.25	98.49
Cabo Verde	Male	0	0	0	0	0	0	0	0	0	0	0	0.01	0.05	0.14	0.08	0.22	0.27	0.48	0.96	0.52
Cabo Verde	Female	0	0	0	0	0	0	0	0	0	0.03	0.01	0.02	0	0.03	0.06	0.23	0.19	0.35	0.25	1.46
Cambodia	Male	0	0.01	0.01	0.01	0.01	0.04	0.03	0.1	0.19	0.34	0.68	0.76	1.93	3.13	3.21	6.35	8.85	8.6	11.39	17.18
Cambodia	Female	0	0.01	0.01	0.01	0.02	0.02	0.05	0.06	0.12	0.29	0.16	0.52	0.98	1.61	1.7	2.02	2.98	3.84	5.85	6.11
Cameroon	Male	0	0	0	0	0	0	0	0	0	0.01	0.01	0.03	0.12	0.3	0.2	0.44	0.55	0.97	1.94	1.44
Cameroon	Female	0	0	0	0.01	0	0	0	0.01	0.03	0.28	0.05	0.11	0.03	0.16	0.3	1.39	0.65	1.01	0.79	9.76
Canada	Male	0	0	0	0	0.01	0.01	0.01	0.01	0.02	0.02	0.04	0.09	0.17	0.28	0.42	0.87	2.04	3.34	6.33	11.62
Canada	Female	0	0	0	0	0	0	0.01	0.02	0.03	0.06	0.07	0.1	0.19	0.24	0.44	0.75	1.66	2.42	5	9.43
Central African Republic	Male	0	0.01	0.01	0.02	0.01	0.01	0.05	0.08	0.14	0.3	0.38	0.86	0.8	1.43	1.27	1.88	3.51	1.77	3.09	3.69
Central African Republic	Female	0.02	0.01	0.01	0	0.01	0.01	0.04	0.06	0.1	0.19	0.21	0.34	0.33	0.56	0.67	1.19	1.22	1.58	1.95	3.05
Chad	Male	0	0	0	0	0	0	0	0	0	0.01	0.01	0.03	0.11	0.29	0.18	0.4	0.49	0.79	1.67	1.13
Chad	Female	0.01	0	0	0.01	0	0	0	0.01	0.02	0.29	0.06	0.12	0.04	0.18	0.33	1.41	0.68	0.97	0.76	8.93
Chile	Male	0	0	0	0	0	0.01	0.01	0.01	0.03	0.03	0.09	0.06	0.18	0.19	0.26	0.54	1.1	1.37	3.46	5.41

Chile	Female	0	0	0	0	0	0.01	0.03	0.01	0.03	0.06	0.1	0.14	0.17	0.2	0.22	0.37	0.78	0.93	0.85	0.31
China	Male	0	0	0	0	0.01	0.01	0.02	0.04	0.05	0.07	0.15	0.22	0.34	0.55	1.1	2	3.07	7.73	12.45	12.85
China	Female	0	0	0	0	0.01	0.01	0.01	0.02	0.03	0.05	0.08	0.11	0.17	0.26	0.46	0.7	1.14	1.77	2.73	4.87
Colombia	Male	0	0	0	0	0.01	0.01	0.02	0.02	0.05	0.04	0.06	0.09	0.12	0.21	0.26	0.35	0.53	0.54	0.77	0.74
Colombia	Female	0	0.01	0	0	0.01	0.02	0.03	0.07	0.07	0.08	0.15	0.18	0.25	0.21	0.24	0.36	0.4	0.78	1.13	0.34
Comoros	Male	0	0	0	0.01	0	0	0	0	0	0	0.01	0.02	0.02	0.05	0.06	0.14	0.32	0.27	0.68	1.52
Comoros	Female	0	0	0	0.04	0	0	0	0	0.01	1.09	0.02	0.05	0.02	0.04	0.11	7.92	0.25	0.59	0.3	126.14
Congo	Male	0	0	0	0.01	0	0.01	0.02	0.03	0.05	0.1	0.14	0.33	0.35	0.7	0.72	1.19	2.46	1.52	3.32	4.42
Congo	Female	0	0	0	0	0	0.01	0.02	0.04	0.06	0.12	0.12	0.21	0.21	0.35	0.43	0.81	0.83	1.23	1.61	2.98
Costa Rica	Male	0	0	0	0	0	0	0	0	0.02	0	0.01	0.03	0.02	0.08	0.06	0.22	0.25	0.37	0.68	0.54
Costa Rica	Female	0	0	0	0	0	0	0	0	0.01	0.02	0.05	0.06	0.11	0.09	0.07	0.2	0.17	0.25	0.43	0.34
Côte d'Ivoire	Male	0	0	0	0	0	0	0	0	0	0.01	0.01	0.03	0.12	0.3	0.18	0.46	0.56	0.96	2.01	1.51
Côte d'Ivoire	Female	0	0	0	0	0	0	0	0.01	0.02	0.24	0.04	0.09	0.03	0.14	0.26	1.25	0.62	0.96	0.76	10.59
Croatia	Male	0	0	0	0	0	0	0	0	0.01	0.04	0.02	0.06	0.09	0.26	0.41	0.46	1.64	1.92	3.8	4.95
Croatia	Female	0	0	0	0	0	0	0	0.02	0.03	0.03	0.07	0.11	0.17	0.28	0.62	1.32	2.04	2.57	1.44	1.99
Cuba	Male	0	0	0	0	0	0	0.02	0.08	0.09	0.28	0.37	0.62	1	1.17	1.7	2.77	1.82	2.51	3.96	4.33
Cuba	Female	0	0	0	0	0.01	0.02	0.06	0.09	0.11	0.27	0.22	0.48	0.6	0.87	0.78	0.89	0.9	1.15	1.13	0.43
Cyprus	Male	0	0	0	0	0	0	0	0	0	0	0.01	0.01	0.03	0.1	0.22	0.43	1.07	1.51	1.75	2.43
Cyprus	Female	0	0	0	0	0	0	0	0.03	0.01	0.01	0.01	0.07	0.04	0.17	0.15	0.63	1.52	2.82	2.59	11.06
Czechia	Male	0	0	0	0	0.01	0	0.01	0.01	0.01	0.02	0.03	0.09	0.18	0.31	0.5	0.72	1.46	2.04	3.54	5.15
Czechia	Female	0	0	0	0	0	0	0	0.01	0.02	0.03	0.06	0.13	0.19	0.41	0.56	0.89	1.51	2.21	2.98	2.91
Democratic People's Republic of Korea	Male	0.01	0	0.01	0.03	0.04	0.05	0.07	0.09	0.14	0.23	0.48	0.66	0.95	1.37	2.11	3.31	3.87	6.6	8.7	9.67
Democratic People's Republic of Korea	Female	0	0	0	0.01	0.02	0.03	0.05	0.06	0.09	0.17	0.28	0.35	0.42	0.55	0.71	0.95	1.26	1.79	2.33	3.57
Democratic Republic of	Male	0	0	0	0.01	0	0.01	0.02	0.03	0.05	0.1	0.14	0.35	0.36	0.69	0.7	1.19	2.37	1.37	2.63	3.92

the Congo																					
Democratic Republic of the Congo	Female	0	0	0	0	0	0	0.01	0.02	0.04	0.08	0.09	0.16	0.16	0.3	0.37	0.68	0.73	0.96	1.26	2.49
Denmark	Male	0	0	0	0	0	0	0	0	0	0.05	0.05	0.11	0.29	0.42	0.99	1.63	3.85	9.77	13.1	20.54
Denmark	Female	0	0	0	0	0.01	0	0.01	0.02	0	0	0.05	0.08	0.18	0.25	0.6	1.11	1.77	3.88	5.56	17.94
Djibouti	Male	0	0	0	0.02	0	0	0	0	0	0.01	0.01	0.02	0.03	0.06	0.08	0.17	0.39	0.35	0.81	1.74
Djibouti	Female	0	0	0	0.03	0	0	0	0	0.01	1.07	0.02	0.05	0.02	0.04	0.12	9.45	0.3	0.73	0.36	145.86
Dominica	Male	0	0	0	0	0	0	0.01	0.03	0.02	0.06	0.1	0.22	0.22	0.32	0.57	0.73	0.68	0.97	1.63	1.83
Dominica	Female	0	0	0	0	0	0.01	0.02	0.03	0.03	0.07	0.04	0.08	0.12	0.17	0.17	0.24	0.28	0.43	0.4	0.89
Dominican Republic	Male	0	0	0	0	0	0	0	0	0.01	0.02	0.05	0.12	0.09	0.16	0.33	0.46	0.46	0.52	0.53	0.51
Dominican Republic	Female	0	0	0	0	0	0	0.01	0.01	0.01	0.01	0.01	0.03	0.04	0.03	0.07	0.07	0.09	0.2	0.2	0.32
Ecuador	Male	0	0	0	0	0.01	0	0.02	0.03	0.03	0.08	0.11	0.13	0.26	0.27	0.55	0.65	1.02	2.54	6.73	6.6
Ecuador	Female	0	0	0	0	0.01	0	0.02	0.05	0.05	0.08	0.09	0.14	0.14	0.17	0.1	0.25	0.53	0.29	2.68	2.73
Egypt	Male	0	0	0	0	0	0	0	0	0	0.01	0.01	0.02	0.03	0.04	0.04	0.09	0.12	0.14	0.26	0.18
Egypt	Female	0	0	0	0	0	0	0	0	0.01	0.01	0.01	0.01	0.01	0.03	0.05	0.09	0.12	0.17	0.21	0.23
El Salvador	Male	0	0	0	0	0	0	0	0.01	0.02	0.03	0.04	0.02	0.03	0.13	0.08	0.34	0.57	1.39	1.63	2.86
El Salvador	Female	0	0	0	0	0	0	0	0	0	0	0.02	0.01	0.03	0.05	0.09	0.18	0.18	0.4	0.53	0.24
Equatorial Guinea	Male	0	0	0	0	0	0	0.01	0.02	0.03	0.07	0.09	0.24	0.27	0.55	0.6	1.1	2.38	1.52	3.13	4.21
Equatorial Guinea	Female	0	0	0	0	0	0	0.01	0.02	0.03	0.07	0.07	0.13	0.14	0.27	0.37	0.77	0.88	1.18	1.6	2.84
Eritrea	Male	0	0	0	0.02	0	0	0	0.01	0	0.01	0.01	0.03	0.05	0.08	0.09	0.18	0.38	0.31	0.72	1.37
Eritrea	Female	0	0	0	0.03	0	0	0	0	0.02	1.38	0.03	0.06	0.02	0.04	0.12	8.53	0.26	0.66	0.33	114.86
Estonia	Male	0	0	0	0	0	0	0.01	0.05	0.06	0.04	0.07	0.09	0.24	0.8	1.13	2.57	4.33	7.06	10.17	16.09
Estonia	Female	0	0	0	0	0	0.01	0	0.02	0	0.03	0.01	0.11	0.39	0.61	1.01	1.51	3.05	3.41	5.48	8.49
Eswatini	Male	0	0.01	0.01	0.01	0.01	0.02	0.03	0.04	0.08	0.1	0.18	0.26	0.26	0.59	0.69	0.98	0.94	0.97	4.01	1.32
Eswatini	Female	0	0	0	0	0	0.01	0.01	0.03	0.03	0.05	0.07	0.09	0.07	0.12	0.17	0.23	0.38	0.55	1.22	0.69
Ethiopia	Male	0	0	0	0.02	0	0	0	0	0	0	0.01	0.02	0.03	0.07	0.08	0.18	0.41	0.31	0.72	1.5

Ethiopia	Female	0	0	0	0.04	0	0	0	0	0.01	0.89	0.02	0.05	0.02	0.04	0.14	10.03	0.32	0.71	0.35	142.21
Fiji	Male	0	0	0	0	0	0	0	0	0.03	0.01	0.06	0.13	0.21	0.08	0.11	0.7	2.73	1.08	4.37	0.79
Fiji	Female	0	0	0	0	0	0	0.01	0.03	0.04	0.01	0.02	0.09	0.03	0.27	0.26	0.42	0.08	0.18	0.34	0.47
Finland	Male	0	0	0	0	0	0	0.01	0	0.01	0	0	0.05	0.07	0.08	0.12	0.56	0.81	2.49	3.2	4.74
Finland	Female	0	0	0	0	0	0	0	0	0.01	0	0.01	0.02	0.05	0.05	0.24	0.48	0.69	0.85	1.53	1.48
France	Male	0	0	0	0	0	0	0	0.01	0.01	0.02	0.02	0.05	0.07	0.11	0.2	0.37	0.81	1.63	3.11	4.49
France	Female	0	0	0	0	0	0	0.01	0.01	0.02	0.03	0.05	0.06	0.09	0.16	0.26	0.45	0.89	1.76	2.89	3.85
Gabon	Male	0	0	0	0.01	0	0.01	0.02	0.04	0.06	0.12	0.16	0.38	0.43	0.83	0.88	1.44	3	1.86	3.7	5
Gabon	Female	0	0	0	0	0	0	0.02	0.03	0.05	0.11	0.11	0.19	0.2	0.37	0.49	1.01	1.18	1.68	2.35	4.41
Gambia	Male	0	0	0	0	0	0	0	0	0	0.01	0.01	0.03	0.13	0.33	0.2	0.46	0.56	1.03	2.13	1.41
Gambia	Female	0	0	0	0	0	0	0	0.01	0.03	0.29	0.06	0.12	0.03	0.17	0.31	1.46	0.71	1.26	0.95	10.83
Germany	Male	0	0	0	0	0	0	0	0.01	0.01	0.02	0.03	0.05	0.1	0.15	0.26	0.54	0.9	1.49	2.9	3.17
Germany	Female	0	0	0	0	0	0	0.01	0.01	0.02	0.02	0.04	0.08	0.1	0.18	0.35	0.58	1.25	1.77	2.4	2.3
Ghana	Male	0.01	0	0.01	0.03	0.04	0.01	0	0.02	0.04	0.13	0.2	0.38	1.39	3.26	2.48	5.06	7.12	14.77	28.98	26.6
Ghana	Female	0.01	0	0	0	0	0	0	0	0.01	0.27	0.05	0.1	0.08	0.21	0.35	1.76	0.82	1.38	1.13	16.76
Greece	Male	0	0	0	0	0	0	0	0	0	0	0.01	0.01	0.02	0.05	0.04	0.09	0.16	0.27	0.43	0.46
Greece	Female	0	0	0	0	0	0	0	0	0.01	0	0	0.01	0.01	0.02	0.05	0.07	0.15	0.26	0.36	0.44
Greenland	Male	0	0	0	0	0	0	0	0	0	0.01	0.01	0.01	0.02	0.04	0.07	0.15	0.34	0.62	1.44	1.33
Greenland	Female	0	0	0	0	0	0	0	0	0	0	0.01	0.01	0.02	0.05	0.08	0.14	0.21	0.3	0.3	0.27
Grenada	Male	0.01	0	0	0	0.01	0.01	0.04	0.16	0.15	0.49	0.55	1.17	1.69	2.52	3.69	5.13	4.17	5.48	8.35	10.47
Grenada	Female	0	0	0	0	0.01	0.02	0.08	0.15	0.1	0.21	0.21	0.44	0.52	0.78	0.7	0.82	0.95	1.55	1.3	2.63
Guam	Male	0	0	0	0	0	0	0	0	0.02	0.01	0.02	0.05	0.07	0.03	0.03	0.15	0.66	0.24	1.1	0.21
Guam	Female	0	0	0	0	0	0	0	0.02	0.02	0.01	0.02	0.05	0.02	0.15	0.12	0.28	0.06	0.14	0.2	0.27
Guatemala	Male	0	0	0	0	0	0.01	0.05	0.02	0.07	0.05	0.06	0.18	0.31	0.29	0.42	0.89	0.9	0.98	0.84	0.62
Guatemala	Female	0.01	0	0	0.01	0.02	0.03	0.02	0.05	0.1	0.12	0.17	0.23	0.44	0.56	0.53	0.56	0.78	1.89	4.44	5.67
Guinea	Male	0	0	0	0	0	0	0	0	0	0.01	0.01	0.03	0.13	0.32	0.2	0.45	0.55	0.9	1.89	1.35
Guinea	Female	0.01	0	0	0.01	0	0	0	0.01	0.03	0.3	0.06	0.12	0.04	0.18	0.32	1.35	0.64	0.95	0.71	9.03
Guinea-Bissau	Male	0	0	0	0	0	0	0	0	0.01	0.02	0.02	0.05	0.19	0.43	0.26	0.56	0.63	1.05	2.04	1.42
Guinea-Bissau	Female	0	0	0	0.01	0	0	0	0.02	0.05	0.46	0.08	0.17	0.05	0.21	0.39	1.62	0.76	1.22	0.91	9.78
Guyana	Male	0.01	0	0	0	0.01	0.02	0.05	0.18	0.18	0.51	0.56	1.02	1.29	1.43	2	2.67	2	2.55	4.18	5.1
Guyana	Female	0	0	0.01	0	0.02	0.04	0.13	0.22	0.17	0.4	0.36	0.65	0.67	0.9	0.8	0.87	0.9	1.37	1.47	3.42

Haiti	Male	0.07	0.02	0.01	0	0.01	0.01	0.02	0.07	0.12	0.36	0.5	1.11	1.33	1.69	2.54	3.58	3.07	3.84	5	4.91
Haiti	Female	0	0.01	0.02	0	0.02	0.03	0.13	0.21	0.19	0.32	0.31	0.64	0.69	0.9	0.86	0.99	1.09	1.55	1.14	2.36
Honduras	Male	0	0	0	0	0.01	0.01	0.03	0.04	0.14	0.12	0.24	0.45	0.7	1.4	1.82	3.06	4.22	6.21	6.83	3.54
Honduras	Female	0	0	0	0	0.01	0.01	0.02	0.06	0.08	0.11	0.24	0.25	0.23	0.28	0.46	0.59	0.63	0.8	1.41	1.04
Hungary	Male	0	0	0	0	0.01	0.01	0.01	0.02	0.02	0.07	0.11	0.37	0.46	1.01	1.59	3.01	4.7	6.85	9.98	15.81
Hungary	Female	0	0	0	0	0	0.01	0.02	0.03	0.05	0.07	0.21	0.31	0.56	1.18	1.7	3.09	4.18	6.07	7.61	9.98
Iceland	Male	0	0	0	0	0	0	0.01	0.01	0.02	0.04	0.05	0.1	0.17	0.31	0.62	1.26	2.27	4.13	7.3	13.71
Iceland	Female	0	0	0	0	0	0	0.01	0.01	0.02	0.02	0.03	0.05	0.09	0.16	0.3	0.59	0.99	1.69	2.98	4.87
India	Male	0	0.01	0	0	0	0.01	0.01	0.03	0.06	0.1	0.15	0.25	0.41	0.74	1.38	2.03	3.17	3.62	6.42	5.72
India	Female	0	0	0.01	0	0.01	0.01	0.03	0.04	0.06	0.1	0.15	0.27	0.26	0.55	0.78	1.55	1.3	1.51	2.04	3.38
Indonesia	Male	0	0.01	0.01	0.01	0.01	0.04	0.02	0.1	0.24	0.47	0.83	0.95	2.78	4.32	4.2	7.34	9.59	8.38	11.9	20.24
Indonesia	Female	0	0.01	0.01	0.01	0.02	0.01	0.02	0.03	0.09	0.15	0.09	0.28	0.6	0.88	0.74	0.88	1.17	1.68	2.97	3.47
Iran (Islamic Republic of)	Male	0	0	0	0	0	0	0	0.01	0.01	0.01	0.02	0.04	0.06	0.1	0.12	0.35	0.59	0.69	1.34	1.62
Iran (Islamic Republic of)	Female	0	0	0	0	0	0	0	0.01	0.01	0.01	0.02	0.03	0.05	0.12	0.2	0.39	0.59	0.83	0.96	0.44
Iraq	Male	0	0.01	0	0	0	0	0.01	0.01	0.02	0.05	0.05	0.08	0.08	0.12	0.17	0.37	0.6	0.92	2.87	0.85
Iraq	Female	0	0	0	0	0	0	0	0	0.01	0.01	0.02	0.03	0.02	0.08	0.09	0.14	0.22	0.21	0.29	0.76
Ireland	Male	0	0	0	0	0	0.01	0	0	0.01	0.02	0.03	0.04	0.08	0.11	0.18	0.45	0.7	1.65	3.46	5.09
Ireland	Female	0	0	0	0	0	0	0	0.02	0.02	0	0.05	0.04	0.09	0.2	0.51	0.73	1.2	1.95	3.25	4.79
Israel	Male	0	0	0	0	0	0	0	0.01	0.02	0.05	0.03	0.08	0.18	0.27	0.46	1.4	2.79	4.84	8.09	15.75
Israel	Female	0	0	0	0	0	0	0	0	0	0.01	0.02	0.06	0.14	0.22	0.39	1.26	1.9	3.98	7.03	14.4
Italy	Male	0	0	0	0	0	0	0.01	0.01	0.01	0.02	0.03	0.04	0.07	0.15	0.26	0.52	0.94	1.92	3.62	6.3
Italy	Female	0	0	0	0	0	0	0	0.01	0.02	0.02	0.03	0.05	0.07	0.12	0.19	0.38	0.69	1.07	1.63	2.83
Jamaica	Male	0	0	0	0	0	0.01	0	0	0	0.01	0.07	0.33	0.54	0.83	1.16	1.74	2.23	1.34	1.04	4.95
Jamaica	Female	0	0	0.01	0	0	0	0.01	0.08	0.05	0.01	0	0.01	0.03	0.52	0.32	0.17	0.82	1.53	0.57	4.64
Japan	Male	0	0	0	0	0	0.01	0.01	0.02	0.02	0.02	0.05	0.07	0.14	0.23	0.44	0.83	1.43	2.97	4.24	6.54
Japan	Female	0	0	0	0	0	0	0.01	0.01	0.02	0.03	0.04	0.1	0.11	0.28	0.42	1	2.22	4.43	8.11	19.21
Jordan	Male	0	0	0	0	0	0	0	0	0	0	0.01	0.01	0.02	0.03	0.04	0.12	0.19	0.25	0.54	0.44
Jordan	Female	0	0	0	0	0	0	0	0	0.01	0.01	0.01	0.01	0.02	0.06	0.08	0.11	0.17	0.22	0.32	0.38
Kazakhstan	Male	0	0	0	0	0	0.03	0.11	0	0.17	0.6	0.78	0.8	2.28	6.15	6.04	12.86	19.79	17.57	9.18	1.16

Kazakhstan	Female	0	0	0	0	0	0.03	0.06	0.01	0.1	0.15	0.68	1.1	1.66	4	4.68	7.94	13.32	8.95	11.89	22.81
Kenya	Male	0	0	0	0.02	0	0	0	0	0	0.01	0.01	0.02	0.03	0.06	0.07	0.16	0.36	0.29	0.66	1.34
Kenya	Female	0	0	0	0.02	0	0	0	0	0.01	1.08	0.02	0.05	0.01	0.03	0.1	8.02	0.26	0.64	0.32	128.29
Kuwait	Male	0	0	0	0	0	0	0	0	0	0	0	0.02	0.02	0.04	0.06	0.16	0.29	0.49	1.45	0.87
Kuwait	Female	0	0	0	0	0	0	0	0	0	0	0	0.01	0.01	0.03	0.05	0.09	0.22	0.25	0.41	0.4
Kyrgyzstan	Male	0.01	0.04	0.02	0.01	0.02	0.06	0.08	0.09	0.22	0.31	0.37	0.39	0.47	1.05	0.55	1.2	0.51	2.14	4.31	4.49
Kyrgyzstan	Female	0	0	0	0	0	0.01	0.04	0.02	0.06	0.06	0.08	0.08	0.24	0.3	0.35	0.49	0.62	0.3	0.43	0.54
Lao People's Democratic Republic	Male	0	0.01	0.01	0.01	0.01	0.04	0.03	0.1	0.17	0.29	0.57	0.6	1.54	2.48	2.61	4.9	7.21	7.21	9.84	15.45
Lao People's Democratic Republic	Female	0	0.01	0.01	0.01	0.02	0.02	0.06	0.07	0.13	0.26	0.14	0.45	0.86	1.39	1.41	1.75	2.56	2.99	4.64	4.92
Latvia	Male	0	0	0	0	0	0	0	0.04	0.11	0.13	0.23	0.48	0.54	2.82	4.29	8.55	13.29	17.6	28.78	32.08
Latvia	Female	0	0	0	0	0	0	0	0.05	0	0.22	0.27	0.61	1.13	1.79	2.44	4.59	7.36	7.91	13.71	12.08
Lebanon	Male	0	0	0	0	0	0	0	0	0	0	0	0.01	0.02	0.02	0.03	0.08	0.14	0.19	0.42	0.32
Lebanon	Female	0	0	0	0	0	0	0	0	0.01	0.01	0.01	0.01	0.02	0.04	0.06	0.12	0.19	0.27	0.37	0.33
Lesotho	Male	0	0.01	0.01	0.02	0.01	0.02	0.03	0.04	0.09	0.12	0.22	0.32	0.34	0.75	0.79	1.03	1.01	1.04	4.19	1.32
Lesotho	Female	0.01	0.01	0	0	0.01	0.02	0.02	0.05	0.05	0.08	0.11	0.15	0.11	0.19	0.24	0.32	0.52	0.73	1.55	0.87
Liberia	Male	0	0	0	0	0	0	0	0	0	0.01	0.01	0.02	0.08	0.21	0.14	0.33	0.44	0.79	1.72	1.23
Liberia	Female	0	0	0	0.01	0	0	0	0.01	0.03	0.3	0.06	0.12	0.04	0.18	0.31	1.55	0.73	1.21	0.87	11.21
Libya	Male	0	0	0	0	0	0	0	0	0.01	0.01	0.01	0.02	0.03	0.04	0.05	0.13	0.18	0.26	0.55	0.46
Libya	Female	0	0	0	0	0	0	0	0.01	0.02	0.02	0.02	0.03	0.04	0.09	0.12	0.22	0.25	0.36	0.47	0.44
Lithuania	Male	0	0	0	0	0	0.01	0	0.02	0.06	0.08	0.18	0.28	0.69	1.48	2.28	3.18	5.02	4.98	7.33	14.97
Lithuania	Female	0	0	0.01	0	0.01	0	0.02	0.1	0.04	0.11	0.2	0.44	0.77	1.57	2.18	3.24	6.02	6.38	6.96	7.14
Luxembourg	Male	0	0	0	0	0	0	0	0.01	0.01	0.01	0.02	0.03	0.06	0.12	0.23	0.45	0.83	1.58	2.89	4.77
Luxembourg	Female	0	0	0	0	0	0	0	0	0.01	0.01	0.02	0.03	0.06	0.1	0.19	0.32	0.53	0.77	1.27	1.94
Madagascar	Male	0	0	0	0.01	0	0	0	0	0	0	0.01	0.02	0.02	0.04	0.05	0.11	0.25	0.21	0.52	1.16
Madagascar	Female	0	0	0	0.04	0	0	0	0	0.01	1.08	0.03	0.05	0.02	0.04	0.1	6.6	0.21	0.5	0.25	92.3
Malawi	Male	0	0	0	0.03	0	0	0	0	0	0.01	0.01	0.03	0.04	0.08	0.09	0.2	0.45	0.35	0.77	1.65
Malawi	Female	0	0	0	0.03	0	0	0	0	0.01	0.8	0.02	0.04	0.01	0.04	0.12	8.89	0.29	0.64	0.33	128.74

Malaysia	Male	0	0	0	0	0	0	0	0.01	0.02	0.02	0.04	0.06	0.17	0.38	0.42	1.34	1.41	1.69	2.3	4.94
Malaysia	Female	0	0	0	0	0	0	0	0	0	0.03	0.01	0.16	0.19	0.7	0.86	1.16	1.51	1.71	3.79	4.45
Maldives	Male	0	0	0	0	0	0	0	0	0	0.01	0.01	0.02	0.06	0.12	0.15	0.36	0.67	0.97	1.77	3.59
Maldives	Female	0	0	0	0	0	0	0	0	0	0	0	0.01	0.02	0.05	0.07	0.14	0.31	0.47	0.85	1.09
Mali	Male	0	0	0	0	0	0	0	0	0	0.01	0.01	0.02	0.08	0.22	0.15	0.34	0.42	0.71	1.46	1.03
Mali	Female	0.01	0	0	0.01	0	0	0	0.01	0.03	0.35	0.07	0.15	0.05	0.21	0.4	1.73	0.86	1.25	0.91	11.23
Malta	Male	0	0	0	0	0	0	0.01	0.01	0.02	0.04	0.04	0.08	0.15	0.29	0.54	0.97	1.89	3.48	5.71	9.2
Malta	Female	0	0	0	0	0.01	0	0.01	0.01	0.02	0.02	0.06	0.09	0.15	0.27	0.5	0.87	1.4	2.01	3.02	4.16
Marshall Islands	Male	0	0	0	0	0	0.01	0	0.01	0.2	0.07	0.19	0.39	0.51	0.19	0.22	1.11	3.86	1.29	3.76	0.75
Marshall Islands	Female	0	0	0.01	0	0	0	0.07	0.32	0.36	0.1	0.19	0.69	0.14	1.29	1.26	1.99	0.35	0.76	1.28	1.71
Mauritania	Male	0	0	0	0	0	0	0	0	0	0	0	0.01	0.06	0.15	0.1	0.26	0.34	0.66	1.35	0.98
Mauritania	Female	0	0	0	0	0	0	0	0.01	0.02	0.29	0.06	0.14	0.04	0.21	0.4	1.87	0.96	1.6	1.18	13.67
Mauritius	Male	0	0	0	0	0	0	0	0	0	0	0.01	0.01	0.06	0.11	0.1	0.21	0.44	0.27	0.8	1.09
Mauritius	Female	0	0	0	0	0	0	0	0	0.01	0.01	0	0.01	0.05	0.08	0.05	0.07	0.14	0.22	0.38	0.37
Mexico	Male	0	0	0.01	0.01	0.02	0.04	0.07	0.11	0.24	0.26	0.46	0.69	0.89	1.35	1.63	2.71	3.34	4.39	6.72	6.57
Mexico	Female	0	0	0.01	0.01	0.02	0.04	0.07	0.12	0.22	0.3	0.62	0.84	0.92	1.07	1.24	1.63	2.07	2.93	4.82	4.55
Micronesia (Federated States of)	Male	0	0	0	0	0	0.01	0	0	0.18	0.07	0.22	0.42	0.53	0.18	0.22	1.13	3.81	1.26	3.68	0.71
Micronesia (Federated States of)	Female	0	0	0.01	0	0	0	0.08	0.28	0.27	0.11	0.24	0.81	0.17	1.51	1.48	2.42	0.43	0.92	1.6	2.17
Mongolia	Male	0	0	0	0	0	0	0.03	0.05	0.01	0.01	0.01	0.01	0.04	0.08	0.09	0.25	0.23	0.68	1.4	1.09
Mongolia	Female	0	0	0	0	0	0	0	0	0.01	0.18	0.03	0.05	0.43	0.13	0.13	0.19	0.29	0.43	0.92	1.24
Montenegro	Male	0	0	0	0	0	0	0	0	0	0	0	0.01	0.01	0.02	0.03	0.06	0.09	0.13	0.22	0.25
Montenegro	Female	0	0	0	0	0	0	0	0	0	0	0	0.01	0.01	0.01	0.02	0.03	0.04	0.06	0.07	0.09
Morocco	Male	0	0	0	0	0	0	0	0	0.01	0.01	0.01	0.02	0.03	0.05	0.06	0.16	0.27	0.36	0.72	0.59
Morocco	Female	0	0	0	0	0	0	0	0.01	0.02	0.02	0.02	0.03	0.04	0.09	0.13	0.25	0.35	0.47	0.56	0.55
Mozambique	Male	0	0	0	0.03	0	0.01	0.01	0.01	0.01	0.01	0.02	0.04	0.05	0.11	0.11	0.26	0.56	0.45	0.94	1.97

Mozambique	Female	0	0	0	0.05	0	0	0	0	0.01	1.23	0.03	0.06	0.02	0.04	0.13	9.76	0.31	0.79	0.4	151.16
Myanmar	Male	0.01	0.01	0.01	0.02	0.02	0.05	0.03	0.11	0.2	0.34	0.66	0.68	1.71	2.69	2.94	5.7	8.25	7.68	10.01	15.88
Myanmar	Female	0	0.01	0.01	0.01	0.02	0.02	0.06	0.06	0.11	0.24	0.12	0.4	0.75	1.27	1.34	1.72	2.75	3.28	4.88	5.33
Namibia	Male	0	0	0	0.01	0.01	0.01	0.01	0.01	0.03	0.04	0.09	0.15	0.17	0.39	0.5	0.73	0.74	0.76	3.13	1.12
Namibia	Female	0	0	0	0	0	0.01	0.01	0.02	0.01	0.03	0.04	0.05	0.04	0.08	0.12	0.17	0.3	0.42	0.85	0.52
Nepal	Male	0	0	0	0	0	0	0	0.01	0.03	0.05	0.09	0.15	0.29	0.58	1.06	1.58	2.41	3.32	6.09	4.89
Nepal	Female	0	0	0.01	0	0	0.01	0.02	0.04	0.06	0.12	0.13	0.26	0.25	0.57	0.9	2.05	1.64	2.38	3.11	4.76
Netherlands	Male	0	0	0	0	0	0	0	0.01	0.02	0.03	0.05	0.1	0.16	0.29	0.53	0.95	2.58	4.89	8.34	14.49
Netherlands	Female	0	0	0	0	0	0	0.01	0.02	0.01	0.02	0.07	0.1	0.24	0.32	0.67	1.29	2.49	4.16	6.45	11.34
New Zealand	Male	0	0	0	0	0	0.02	0.02	0.02	0.03	0.08	0.09	0.17	0.19	0.6	0.76	1.51	2.59	5.53	5.3	8.36
New Zealand	Female	0	0	0	0	0	0	0.05	0.07	0.09	0.17	0.16	0.47	0.46	0.79	0.97	1.75	2.05	3.27	3.68	3.65
Nicaragua	Male	0	0	0	0	0	0	0	0.01	0.03	0.04	0.07	0.1	0.17	0.39	0.52	0.66	0.94	2.32	3.22	1.34
Nicaragua	Female	0	0	0	0	0	0	0.01	0.01	0.01	0.06	0.16	0.23	0.14	0.17	0.4	0.22	0.63	0.46	0.71	1.93
Niger	Male	0	0	0	0	0	0	0	0	0	0.01	0.01	0.02	0.08	0.21	0.14	0.34	0.43	0.69	1.47	0.99
Niger	Female	0.01	0	0	0.01	0	0	0	0.01	0.02	0.27	0.06	0.12	0.04	0.18	0.33	1.5	0.71	1.05	0.82	10
Nigeria	Male	0	0	0	0	0	0	0	0	0	0.01	0.01	0.02	0.08	0.19	0.13	0.33	0.43	0.76	1.62	1.16
Nigeria	Female	0.01	0	0	0	0	0	0	0	0.01	0.15	0.03	0.06	0.02	0.1	0.2	1.02	0.48	0.79	0.63	8.16
North Macedonia	Male	0	0	0	0	0	0	0	0	0	0	0	0.01	0.01	0.02	0.03	0.05	0.08	0.12	0.21	0.25
North Macedonia	Female	0	0	0	0	0	0	0	0	0	0	0	0	0.01	0.01	0.01	0.03	0.04	0.06	0.1	0.1
Northern Mariana Islands	Male	0	0	0	0	0	0	0	0	0.02	0.01	0.02	0.06	0.1	0.04	0.05	0.28	1.14	0.39	1.34	0.35
Northern Mariana Islands	Female	0	0	0	0	0	0	0.01	0.03	0.03	0.01	0.03	0.1	0.03	0.29	0.35	0.85	0.16	0.37	0.6	0.82
Norway	Male	0	0	0	0	0.01	0	0	0.01	0.01	0.01	0.01	0.06	0.08	0.23	0.51	1.33	2.5	4.82	7.41	15.88
Norway	Female	0	0	0	0	0	0	0	0	0	0.02	0.01	0.07	0.19	0.28	0.54	1.35	1.96	3.25	5.5	9
Oman	Male	0	0	0	0	0	0	0	0	0	0	0.01	0.02	0.04	0.07	0.1	0.35	0.82	0.91	1.9	1.45
Oman	Female	0	0	0	0	0	0	0	0	0.01	0.01	0.01	0.03	0.04	0.11	0.23	0.41	0.6	0.61	0.73	0.6

Pakistan	Male	0.01	0.02	0.01	0	0	0.01	0.02	0.03	0.09	0.13	0.18	0.27	0.48	0.86	1.61	2.2	3.4	4.38	7.78	7.66
Pakistan	Female	0.01	0.01	0.02	0	0.01	0.01	0.05	0.08	0.11	0.18	0.19	0.39	0.38	0.79	1.23	2.52	1.89	2.54	3.36	5.73
Palestine	Male	0	0	0	0	0	0	0	0	0	0.01	0.01	0.03	0.05	0.13	0.12	0.31	0.61	0.97	3.86	2.42
Palestine	Female	0	0	0	0	0	0	0	0	0	0.01	0.01	0.03	0.04	0.11	0.16	0.31	0.37	0.46	0.5	0.59
Panama	Male	0	0	0	0	0	0	0	0	0.01	0.01	0.01	0.01	0.03	0.04	0.05	0.23	0.36	0.55	1.1	0.54
Panama	Female	0	0	0	0	0	0	0	0	0.02	0.01	0.02	0.03	0.02	0.03	0.1	0.18	0.25	0.37	0.69	0.39
Papua New Guinea	Male	0	0.01	0.01	0	0	0.01	0	0	0.11	0.04	0.11	0.25	0.34	0.13	0.17	0.84	2.76	0.9	2.39	0.5
Papua New Guinea	Female	0.02	0	0.02	0	0	0	0.05	0.2	0.25	0.08	0.15	0.49	0.13	1.06	1.01	1.53	0.3	0.6	0.95	1.33
Paraguay	Male	0	0	0	0	0	0	0	0	0.02	0.05	0.1	0.22	0.29	0.45	0.31	0.64	0.44	1.06	3.15	0.9
Paraguay	Female	0	0	0	0	0	0.01	0.02	0.05	0.08	0.16	0.14	0.21	0.21	0.37	0.29	0.4	0.78	0.71	0.35	0.42
Peru	Male	0	0	0	0	0	0	0.01	0.01	0.02	0.04	0.03	0.03	0.1	0.15	0.28	0.3	0.32	0.51	1.27	2.54
Peru	Female	0	0	0	0	0	0	0	0.02	0.03	0.03	0.05	0.09	0.07	0.1	0.13	0.18	0.59	0.37	1.53	1.45
Philippines	Male	0.01	0.03	0.02	0.02	0.04	0.1	0.09	0.28	0.43	0.65	1.14	1.19	2.62	4.45	5	9.17	13.19	10.94	12.33	18.6
Philippines	Female	0	0.02	0.01	0.01	0.04	0.05	0.12	0.13	0.2	0.43	0.24	0.69	1.16	1.85	2.06	2.63	4.12	5.57	10.89	12.57
Poland	Male	0	0	0	0	0	0	0	0.01	0.01	0.03	0.04	0.08	0.1	0.19	0.27	0.4	0.49	0.74	1.19	1.68
Poland	Female	0	0	0	0	0	0	0	0.01	0.02	0.02	0.05	0.06	0.08	0.13	0.17	0.24	0.3	0.41	0.74	0.94
Portugal	Male	0	0	0	0	0	0	0	0	0.01	0.04	0.04	0.06	0.1	0.22	0.32	0.62	1.09	1.91	3.18	3.82
Portugal	Female	0	0	0	0	0	0	0.01	0.03	0.04	0.02	0.07	0.1	0.07	0.2	0.46	0.66	1.56	2.14	4.01	7.44
Puerto Rico	Male	0	0	0	0	0	0	0	0	0.01	0.04	0.01	0.07	0.1	0.07	0.1	0.31	0.53	1.02	0.6	0.43
Puerto Rico	Female	0	0	0	0	0	0	0.01	0	0	0	0.02	0.06	0.11	0.12	0.13	0.18	0.29	0.67	0.36	2.12
Qatar	Male	0	0	0	0	0	0	0	0	0	0	0	0	0.01	0.01	0.01	0.06	0.21	0.48	1.13	0.86
Qatar	Female	0	0	0	0	0	0	0	0	0	0	0	0.01	0.03	0.1	0.19	0.42	0.88	1.36	1.01	1.79
Republic of Korea	Male	0	0	0	0	0	0	0	0	0	0	0.02	0.03	0.05	0.07	0.24	0.6	1.32	3.68	7.53	12.65
Republic of Korea	Female	0	0	0	0	0	0	0	0	0	0.01	0.01	0.04	0.03	0.12	0.23	0.64	1.48	2.99	4.99	5.53
Republic of Moldova	Male	0	0	0	0	0.01	0.01	0.01	0.05	0.12	0.15	0.26	0.43	0.84	1.39	2.48	2.66	2.42	4.05	7.63	7.29
Republic of	Female	0	0	0	0	0	0.02	0.03	0.1	0.06	0.25	0.28	0.55	0.98	1.38	2	1.82	2.28	2.83	2.53	3.76

Moldova																					
Romania	Male	0	0	0	0	0	0	0	0	0	0	0	0.01	0.01	0.03	0.05	0.1	0.18	0.26	0.43	0.29
Romania	Female	0	0	0	0	0	0	0	0	0	0	0	0.01	0.01	0.02	0.04	0.06	0.09	0.12	0.14	0.14
Russian Federation	Male	0	0	0	0.01	0.02	0.04	0.05	0.16	0.19	0.26	0.49	0.77	1.37	2.5	4.44	7.15	9.63	10.21	20.41	39.5
Russian Federation	Female	0	0	0.01	0	0.02	0.03	0.05	0.14	0.1	0.3	0.43	0.88	1.56	2.46	3.69	5.36	7.57	7.39	13.64	23.47
Rwanda	Male	0	0	0	0.02	0	0	0	0	0	0	0.01	0.02	0.03	0.05	0.06	0.15	0.33	0.3	0.69	1.52
Rwanda	Female	0	0	0	0.03	0	0	0	0	0.01	0.82	0.02	0.04	0.01	0.03	0.09	6.69	0.22	0.52	0.27	114.81
Saint Lucia	Male	0	0	0	0	0	0	0	0.01	0.01	0.05	0.05	0.09	0.12	0.13	0.23	0.33	0.28	0.46	0.87	1.05
Saint Lucia	Female	0	0	0.01	0	0.02	0.04	0.13	0.22	0.23	0.47	0.44	0.7	0.8	1.2	1.13	1.33	1.69	3.66	3.83	8.84
Saint Vincent and the Grenadines	Male	0.01	0	0	0	0.01	0.01	0.03	0.13	0.12	0.49	0.49	0.77	0.94	1.13	1.57	2.72	2.06	3.34	6.32	6.18
Saint Vincent and the Grenadines	Female	0	0	0	0	0	0	0.01	0.01	0.01	0.02	0.02	0.03	0.03	0.04	0.05	0.07	0.13	0.19	0.21	0.25
Samoa	Male	0	0	0	0	0	0	0	0	0.08	0.03	0.09	0.18	0.26	0.1	0.13	0.69	2.44	0.82	2.42	0.44
Samoa	Female	0	0	0.01	0	0	0	0.05	0.21	0.24	0.08	0.14	0.5	0.11	1.03	0.97	1.66	0.3	0.68	1.21	1.81
Sao Tome and Principe	Male	0	0	0	0	0	0	0	0	0	0	0.01	0.02	0.08	0.2	0.13	0.32	0.36	0.69	1.47	1.17
Sao Tome and Principe	Female	0	0	0	0.01	0	0	0	0.01	0.04	0.4	0.07	0.14	0.05	0.21	0.41	2	1.01	2.08	1.67	16.76
Saudi Arabia	Male	0	0	0	0	0	0	0	0	0	0	0	0.01	0.01	0.03	0.04	0.12	0.31	0.35	0.69	0.53
Saudi Arabia	Female	0	0	0	0	0	0	0	0	0	0	0	0.01	0.01	0.03	0.05	0.24	0.17	0.2	0.27	0.28
Senegal	Male	0	0	0	0	0	0	0	0	0	0.01	0.01	0.02	0.11	0.27	0.18	0.44	0.55	0.93	1.95	1.43
Senegal	Female	0	0	0	0	0	0	0	0.01	0.02	0.24	0.05	0.1	0.03	0.16	0.29	1.42	0.68	1.07	0.83	10
Serbia	Male	0	0	0	0	0	0	0	0.01	0	0.02	0.02	0.07	0.09	0.22	0.4	0.56	1.11	1.14	1.05	0.45
Serbia	Female	0	0	0	0	0	0	0.01	0	0.02	0.03	0.05	0.08	0.13	0.25	0.25	0.39	0.57	0.81	0.77	0.41
Seychelles	Male	0	0	0	0	0.01	0.02	0.02	0.06	0.13	0.21	0.38	0.42	1.06	2.02	1.8	4.2	6.65	5.94	9.33	16.17
Seychelles	Female	0	0	0	0	0.01	0.01	0.02	0.02	0.04	0.12	0.05	0.16	0.37	0.71	0.8	1.31	2.2	2.93	5.44	6.82

Sierra Leone	Male	0	0	0	0	0	0	0	0	0	0.01	0.01	0.02	0.09	0.23	0.14	0.35	0.47	0.76	1.58	1.1
Sierra Leone	Female	0.01	0	0	0	0	0	0	0.01	0.03	0.27	0.05	0.1	0.03	0.15	0.27	1.31	0.59	0.92	0.7	8.98
Singapore	Male	0	0	0	0	0	0	0	0	0	0	0.02	0.04	0.07	0.08	0.25	0.28	0.29	0.74	1.4	1.84
Singapore	Female	0	0	0	0	0	0	0	0	0	0.01	0.01	0.03	0.01	0.05	0.06	0.16	0.32	0.35	0.6	1
Slovakia	Male	0	0	0	0	0	0	0	0	0.01	0.02	0.02	0.05	0.11	0.15	0.3	0.59	0.83	0.95	1.68	1.38
Slovakia	Female	0	0	0.01	0	0	0	0	0.01	0.02	0.04	0.07	0.12	0.19	0.28	0.41	0.73	1.06	1.48	2.36	2.69
Slovenia	Male	0	0	0	0	0	0	0	0	0	0.01	0	0.02	0.03	0.05	0.14	0.32	0.49	0.83	2.45	3.03
Slovenia	Female	0	0	0	0	0	0	0	0	0	0	0.03	0.05	0.04	0.11	0.27	0.55	0.64	0.52	0.86	1.33
Solomon Islands	Male	0	0	0.01	0	0	0.01	0	0.01	0.28	0.1	0.25	0.46	0.55	0.19	0.21	0.98	3.11	0.92	2.26	0.81
Solomon Islands	Female	0	0	0.02	0	0	0.01	0.15	0.52	0.51	0.14	0.26	0.79	0.16	1.38	1.24	1.96	0.32	0.6	0.93	1.14
Somalia	Male	0	0	0	0.05	0	0.01	0.01	0.01	0.01	0.01	0.02	0.05	0.06	0.12	0.12	0.24	0.52	0.39	0.84	1.83
Somalia	Female	0.01	0.01	0	0.09	0.01	0	0	0.01	0.03	1.97	0.06	0.12	0.04	0.08	0.22	12.87	0.44	1.04	0.5	144.61
South Africa	Male	0	0	0	0	0.01	0.01	0.02	0.02	0.03	0.03	0.07	0.11	0.13	0.29	0.33	0.49	0.58	0.75	3.5	1.29
South Africa	Female	0	0	0	0	0	0.02	0.02	0.03	0.02	0.03	0.05	0.07	0.06	0.12	0.13	0.16	0.37	0.56	1.39	0.88
South Sudan	Male	0	0	0	0.01	0	0	0	0	0	0	0.01	0.02	0.03	0.05	0.07	0.15	0.34	0.28	0.66	1.4
South Sudan	Female	0.01	0	0	0.03	0	0	0	0	0.01	0.73	0.02	0.04	0.01	0.03	0.1	7.14	0.22	0.49	0.25	106.53
Spain	Male	0	0	0	0	0	0	0	0.01	0.01	0.02	0.03	0.04	0.07	0.13	0.26	0.43	0.83	1.83	2.97	6.95
Spain	Female	0	0	0	0	0	0	0	0	0.02	0.02	0.04	0.07	0.1	0.15	0.24	0.54	0.97	1.88	3.29	4.78
Sri Lanka	Male	0	0	0	0	0	0	0	0	0	0	0.01	0.01	0.07	0.11	0.09	0.28	0.41	0.83	1.75	3.88
Sri Lanka	Female	0	0	0	0	0	0	0	0	0	0.01	0	0.01	0.04	0.06	0.12	0.1	0.23	0.4	0.86	0.46
Sudan	Male	0	0	0	0	0	0	0	0	0.01	0.01	0.01	0.03	0.03	0.05	0.06	0.16	0.26	0.35	0.64	0.46
Sudan	Female	0	0	0	0	0	0	0	0.01	0.02	0.02	0.02	0.03	0.05	0.1	0.15	0.27	0.38	0.45	0.49	0.4
Suriname	Male	0.01	0.01	0.01	0	0.01	0.02	0.05	0.22	0.22	0.59	0.82	1.54	1.76	2.46	3.11	5.06	4.09	5.46	6.48	6.4
Suriname	Female	0	0	0.01	0	0.01	0.01	0.06	0.1	0.07	0.13	0.12	0.27	0.27	0.34	0.35	0.43	0.46	0.65	0.52	1.12
Sweden	Male	0	0	0	0	0	0	0.01	0.01	0.01	0.03	0.05	0.06	0.14	0.35	0.53	1.17	3.06	5.81	12.64	18.7
Sweden	Female	0	0	0	0	0.01	0	0.01	0.01	0.01	0.01	0.04	0.05	0.1	0.19	0.36	0.67	1.19	2.46	4.73	8.34
Switzerland	Male	0	0	0	0	0	0	0	0	0	0	0.02	0.01	0.02	0.08	0.17	0.31	1.04	2	1.39	0.53
Switzerland	Female	0	0	0	0	0	0	0	0.01	0.01	0	0.02	0.03	0.06	0.13	0.18	0.45	0.8	1.06	1.6	0.36
Syrian Arab	Male	0	0	0	0	0	0	0	0	0	0.04	0.02	0.15	0.04	0.04	0.04	0.09	0.14	0.2	0.42	0.31

Republic																					
Syrian Arab Republic	Female	0.01	0	0	0	0	0	0	0	0	0	0	0.01	0.01	0.03	0.04	0.08	0.13	0.26	0.41	0.29
Tajikistan	Male	0	0.01	0	0	0.05	0.18	0.28	0.18	0.04	0.09	0.33	0.55	1.19	0.79	2.3	5.85	6.62	4.67	5.64	5.83
Tajikistan	Female	0	0	0	0	0.02	0.08	0.01	0	0.01	0.01	0.02	0.04	0.72	0.98	0.51	2.38	4.42	0.79	0.71	0.81
Thailand	Male	0	0	0	0	0	0.03	0.02	0.07	0.17	0.17	0.37	0.56	1.14	2.09	2.97	6.05	8.18	9.45	16.69	41.52
Thailand	Female	0	0	0	0	0	0	0.01	0.02	0.03	0.07	0.08	0.2	0.33	1.24	1.62	3.59	6.26	9.76	21.18	36.75
Timor-Leste	Male	0	0.01	0.01	0.01	0.01	0.03	0.02	0.07	0.13	0.22	0.46	0.5	1.37	2.32	2.46	4.95	6.98	7.05	10.18	15.89
Timor-Leste	Female	0	0.01	0.01	0.01	0.02	0.01	0.04	0.05	0.09	0.23	0.14	0.47	0.81	1.4	1.47	1.83	2.81	3.4	5.34	5.35
Togo	Male	0	0	0	0	0	0	0	0	0	0.01	0.01	0.03	0.12	0.3	0.19	0.42	0.52	0.86	1.76	1.27
Togo	Female	0	0	0	0	0	0	0	0.01	0.02	0.25	0.05	0.1	0.03	0.15	0.27	1.28	0.63	1	0.74	9.27
Tonga	Male	0	0.01	0.01	0	0	0	0	0	0.12	0.05	0.16	0.4	0.58	0.27	0.38	2.1	7.4	2.5	8.1	1.92
Tonga	Female	0	0	0.01	0	0	0	0.01	0.04	0.04	0.01	0.03	0.11	0.03	0.29	0.26	0.43	0.08	0.18	0.34	0.5
Trinidad and Tobago	Male	0.06	0	0	0	0	0	0	0.02	0.14	0.72	1.21	2.04	3.17	3.99	4.97	6.68	5.09	7.79	19.89	3.03
Trinidad and Tobago	Female	0	0	0	0	0	0	0.09	0.17	0.29	0.61	0.61	0.81	0.46	0.81	0.89	1.9	2.43	2.86	0.7	8.5
Tunisia	Male	0	0	0	0	0	0	0	0	0	0	0	0.01	0.01	0.02	0.03	0.08	0.16	0.23	0.48	0.36
Tunisia	Female	0	0	0	0	0	0	0	0	0.01	0.01	0.01	0.01	0.02	0.04	0.07	0.13	0.21	0.26	0.33	0.31
Turkey	Male	0	0	0	0	0	0	0.01	0.01	0.01	0.04	0.05	0.08	0.14	0.16	0.27	0.56	0.84	1.15	2.07	1.89
Turkey	Female	0	0	0	0	0	0	0	0.01	0.02	0.03	0.04	0.06	0.07	0.16	0.31	0.61	0.8	1.22	0.97	1.15
Turkmenistan	Male	0	0.01	0.01	0	0	0.01	0.01	0.01	0.06	0.2	0.22	0.23	0.48	0.94	1.06	2.05	1.72	3.02	4.85	4.52
Turkmenistan	Female	0	0	0	0.01	0	0.02	0.06	0.03	0.11	0.16	0.24	0.27	0.68	0.85	1.24	1.6	1.97	0.7	0.93	1.49
Uganda	Male	0	0	0	0.02	0	0	0	0	0	0	0.01	0.02	0.02	0.05	0.06	0.14	0.3	0.26	0.59	1.35
Uganda	Female	0	0	0	0.02	0	0	0	0	0.01	0.63	0.01	0.03	0.01	0.03	0.08	6.53	0.2	0.49	0.25	105.05
Ukraine	Male	0	0	0	0	0.02	0.04	0.06	0.25	0.36	0.37	0.75	1.06	1.72	3.08	3.93	4.9	3.67	4.74	5.89	9.25
Ukraine	Female	0	0	0	0	0	0.01	0.03	0.09	0.09	0.39	0.56	1.1	1.64	1.94	2.23	2.32	1.72	1.11	0.79	0.65
United Arab Emirates	Male	0	0	0	0	0	0	0	0.01	0.01	0.02	0.02	0.04	0.05	0.08	0.08	0.24	0.39	0.53	1.02	1.18
United Arab Emirates	Female	0	0	0	0	0	0	0	0.01	0.02	0.02	0.03	0.04	0.06	0.14	0.18	0.32	0.56	0.63	0.66	1

United Kingdom	Male	0	0	0.01	0	0.01	0.02	0.02	0.03	0.04	0.06	0.08	0.14	0.23	0.4	0.74	1.34	2.64	4.87	8.83	14.34
United Kingdom	Female	0	0	0	0.01	0.02	0.01	0.03	0.04	0.06	0.07	0.15	0.23	0.35	0.51	0.97	1.65	2.6	3.72	6.49	11.08
United Republic of Tanzania	Male	0	0	0	0.01	0	0	0	0	0	0	0	0.01	0.01	0.02	0.02	0.05	0.11	0.13	0.39	0.95
United Republic of Tanzania	Female	0	0	0	0.03	0	0	0	0	0.01	0.82	0.02	0.04	0.01	0.03	0.09	6.7	0.2	0.47	0.25	118.27
United States of America	Male	0	0	0	0	0	0.01	0.01	0.02	0.03	0.03	0.05	0.08	0.15	0.24	0.4	0.66	1.31	2.24	3.44	3.92
United States of America	Female	0	0	0	0	0.01	0.01	0.02	0.02	0.03	0.05	0.09	0.14	0.21	0.3	0.46	0.77	1.46	2.16	3.07	3.93
United States Virgin Islands	Male	0	0	0	0	0.01	0.01	0.03	0.13	0.19	0.55	0.81	1.54	1.83	2.26	3.3	4.97	3.99	5.52	8.36	9.06
United States Virgin Islands	Female	0	0	0.01	0	0.02	0.03	0.11	0.23	0.24	0.53	0.55	1.08	1.2	1.77	1.93	2.74	3.46	6.05	5.7	11.66
Uruguay	Male	0	0	0	0	0	0	0	0	0	0	0.03	0.01	0.19	0.25	0.37	0.58	1.46	0.94	1.82	0.53
Uruguay	Female	0	0	0	0	0	0.02	0.04	0	0.04	0.11	0.06	0.07	0.13	0.2	0.28	0.63	1.17	1.32	1.28	3.22
Uzbekistan	Male	0	0	0	0	0	0	0	0	0	0	0	0.01	0.02	0.04	0.06	0.19	0.24	0.43	0.71	0.65
Uzbekistan	Female	0	0	0	0	0	0	0	0	0	0	0	0.01	0.02	0.03	0.05	0.1	0.25	0.26	0.29	0.39
Vanuatu	Male	0	0.01	0	0	0	0.01	0	0	0.19	0.06	0.17	0.36	0.46	0.18	0.2	1.03	3.54	1.21	3.42	0.67
Vanuatu	Female	0	0	0.01	0	0	0	0.05	0.24	0.27	0.09	0.17	0.56	0.12	1.18	1.1	1.72	0.3	0.65	1.11	1.61
Venezuela (Bolivarian Republic of)	Male	0	0	0	0	0.02	0.03	0.04	0.06	0.16	0.2	0.35	0.48	0.55	0.71	1.04	1.95	3.16	3.83	5.32	9.77
Venezuela (Bolivarian Republic of)	Female	0.01	0	0.01	0.03	0.06	0.14	0.13	0.15	0.21	0.17	0.33	0.34	0.53	0.5	0.59	1.04	1.45	1.47	1.73	1.49
Viet Nam	Male	0	0	0	0	0	0.01	0	0.02	0.05	0.08	0.16	0.16	0.43	0.64	0.57	1.08	1.68	1.63	2.26	3.33

Viet Nam	Female	0	0	0	0	0.01	0.01	0.02	0.02	0.05	0.1	0.06	0.18	0.36	0.62	0.72	1	1.52	2.47	4.37	5.74
Yemen	Male	0	0	0	0	0	0	0	0	0.01	0.01	0.01	0.03	0.04	0.05	0.06	0.17	0.25	0.34	0.66	0.49
Yemen	Female	0	0	0	0	0	0	0	0.01	0.03	0.02	0.03	0.04	0.05	0.11	0.15	0.26	0.38	0.46	0.5	0.43
Zambia	Male	0	0	0	0.02	0	0	0	0.01	0	0.01	0.01	0.03	0.04	0.07	0.08	0.17	0.4	0.33	0.8	1.77
Zambia	Female	0	0	0	0.04	0	0	0	0	0.02	1.34	0.03	0.07	0.02	0.04	0.13	8.7	0.28	0.69	0.37	142.64
Zimbabwe	Male	0	0.01	0.02	0.12	0.12	0.06	0.01	0.01	0.01	0.02	0.03	0.06	0.12	0.69	0.5	0.71	1.02	1.58	5.63	2.78
Zimbabwe	Female	0	0	0	0	0	0	0	0	0	0	0.01	0.01	0.01	0.03	0.08	0.63	0.25	0.22	0.51	0.6

Supplementary Table S8. Age distribution of DALYs rate (per 100,000) for Urolithiasis in different countries in 2019.

Supplementary Table S8. Age distribution of DALYs rate (per 100,000) for Urolithiasis in different countries in 2019.

Country	Sex	<5	5 to 9	10 to 14	15 to 19	20 to 24	25 to 29	30 to 34	35 to 39	40 to 44	45 to 49	50 to 54	55 to 59	60 to 64	65 to 69	70 to 74	75 to 79	80 to 84	85 to 89	90 to 94	95+
Afghanistan	Male	0.2	0.78	0.94	1.1	1.95	3.53	5.65	7.44	9.12	10.28	10.4	10.97	9.75	8.9	7.64	8.16	7.48	7.08	9.96	5.54
Afghanistan	Female	0.22	0.79	1.19	1.09	1.58	2.24	3.8	5.65	8.78	8.74	9.57	10.36	9.89	10.93	10.2	10.93	10.5	10.11	8.32	5.98
Albania	Male	0.47	0.72	0.83	1.33	2.33	3.23	4.2	5.1	5.3	6.46	7.24	8.23	8.95	9.65	10.19	9.72	9.21	8.37	8.65	7.36
Albania	Female	1.31	0.67	1.21	2.38	3.77	4.26	4.32	4.57	4.77	5.25	6.18	6.67	7.32	7.69	7.01	5.86	4.6	3.58	2.99	3
Algeria	Male	0.16	0.35	0.62	1.05	1.93	3.41	5.35	7.51	8.65	9.77	10.08	9.89	8.64	7.21	5.71	4.96	4	4.13	5.56	2.86
Algeria	Female	0.17	0.39	0.65	0.84	1.17	1.67	2.71	4.05	5.5	6.31	7.44	8.1	7.36	6.22	5.4	4.83	4.29	7.63	8.94	3.24
American Samoa	Male	0.12	0.23	0.42	0.65	1.22	2.66	4.29	6.06	12.51	10.73	14.8	21.31	24.33	12.12	10.58	26.49	64.48	18.19	57.09	8.91
American Samoa	Female	0.31	0.1	0.36	0.35	0.61	1.1	2.44	4.68	5.33	4.82	5.87	8.64	5.97	10.74	9.61	11.07	2.41	2.73	3.38	3.27
Andorra	Male	0.3	0.28	0.48	0.91	2.66	4.81	7.87	10.28	11.97	14.34	16.08	17.69	18.09	19.09	20.38	23.49	27.95	36.07	44.86	53.58
Andorra	Female	0.15	0.22	0.42	1.01	2.38	2.69	4.04	5.18	5.66	6.3	8.53	9.8	11.64	13.21	17.06	20.81	22.88	23.23	27.82	34.91
Angola	Male	0.18	0.37	0.52	1.02	1.21	2.13	3.7	4.97	6.19	9.03	11.28	17.6	16.95	23.55	19.09	22.9	33.39	15.4	23.13	24.28
Angola	Female	0.38	0.34	0.47	0.6	1	1.43	2.51	3.4	4.23	6.01	5.86	6.85	6.05	7.61	7.42	9.49	7.78	7.69	7.92	11.12
Antigua and Barbuda	Male	0.35	0.3	0.39	0.72	1.54	2.7	4.22	6.39	7.22	10.68	13.03	16.72	18.23	15.56	14.9	13.99	11.3	9.46	11.17	10.54
Antigua and Barbuda	Female	0.22	0.21	0.62	0.65	1.53	2.32	5.01	7.77	7.92	13.3	13.74	20.39	19.81	22.65	17.38	16.03	14.08	16.73	11.43	19.31
Argentina	Male	0.09	0.25	0.32	0.86	2.32	5.23	8.93	11.63	13.63	14.61	15.48	14.18	14.35	10.95	10.04	8.58	11	8.24	9.03	8.53
Argentina	Female	0.06	0.11	0.28	0.81	1.89	3.74	5.94	7.12	8.61	9.38	8.85	7.87	6.5	6.14	5.33	4.26	3.63	4.08	4.37	2.18
Armenia	Male	0.3	0.62	0.88	1.75	4	6.58	8.08	8.66	17.94	44.51	46.12	58.23	100.2	164.53	156.65	278.41	276.86	393.88	542.5	518.43
Armenia	Female	0.2	0.36	0.74	1.53	2.5	3.29	8.23	9.92	13.88	19.98	27.32	29.03	87.82	116.2	161.22	251.3	401.49	552.06	803.37	982.58
Australia	Male	0.07	0.09	0.17	0.49	1.56	3.72	5.58	7.58	9.21	12.01	14.37	16.86	16.13	18.77	17.88	19.28	21.92	30.19	44.24	50.06
Australia	Female	0.05	0.07	0.17	0.41	1.4	2.34	3.65	4.27	4.74	5.78	6.14	7.97	8.66	10.36	11.17	12.16	14.55	19.78	23.52	24.25
Austria	Male	0.08	0.1	0.21	0.75	2.71	6.11	10.54	15.54	19.72	23.31	25.85	27.67	27.33	26.25	25.31	25.12	23.47	24.88	31.42	31.39
Austria	Female	0.05	0.07	0.17	0.67	1.91	3.43	4.94	5.74	6.84	7.96	9.11	10.96	12.54	13.56	15.7	18.26	24.53	22.7	22.13	29.2
Azerbaijan	Male	0.3	0.6	1.01	1.77	3.98	6.76	7.93	8.44	9.2	11.13	12.7	14.33	18.6	16.82	14.64	19.92	10.13	8.93	7.31	4.44
Azerbaijan	Female	0.25	0.53	0.78	1.49	2.47	3.21	3.9	4.64	5.44	6.48	7.37	7.9	7.83	7.07	7.29	26.23	67.2	68.21	100.87	131.04
Bahamas	Male	0.33	0.3	0.42	0.73	1.74	2.97	5.19	9.31	9.66	16.7	18.22	23.44	24.35	22.08	21.32	20.74	12.01	11.56	15.74	11.31

Bahamas	Female	0.13	0.2	0.46	0.62	1.54	2.55	6.36	9.54	7.74	12.12	10.67	13.31	11.9	12.53	8.45	7.46	6.53	6.72	4.09	5.74
Bahrain	Male	0.16	0.39	0.71	1.08	1.94	3.4	5.34	7.25	8.59	9.72	10.14	9.82	9.06	7.29	6.66	9.43	14.1	23.08	35.92	43.3
Bahrain	Female	0.17	0.41	0.58	0.8	1.19	1.65	2.61	3.77	4.95	5.88	7.42	7.76	6.87	6.18	6.1	7.52	11.58	17.2	23.32	41.74
Bangladesh	Male	0.23	0.83	0.91	1.32	2.87	4.94	6.98	8.74	10.17	11.88	13.95	15.69	18.59	16.03	13.77	11.5	20.68	13.01	18.15	16
Bangladesh	Female	0.23	0.58	1.14	1.22	2.72	4.05	5.19	6.09	6.23	6.53	8.18	12.62	7.46	9.26	6.39	8.14	4.81	9.27	13.25	21.8
Barbados	Male	0.36	0.37	0.5	0.75	1.83	3.11	5.67	9.79	10.58	20.9	22.27	33.48	36.77	35.36	40.62	44.09	29.29	29.63	37.1	27.7
Barbados	Female	0.13	0.21	0.68	0.64	1.65	2.28	5.07	8.01	7.54	11.69	11.2	14.46	15.23	15.63	11.9	11.54	9.46	12.62	8.14	12.72
Belarus	Male	0.33	0.46	0.68	1.67	4.79	9.32	16.02	27.15	34.79	42	53.34	62.41	73.74	97.5	106	111.6	109.18	83.2	94.01	110.2
Belarus	Female	0.24	0.41	1.06	1.81	4.18	6.65	8.63	14.95	13.63	25.73	29.16	47.23	58.1	61.39	62.79	55.59	51.21	33.83	39.51	47.91
Belgium	Male	0.08	0.1	0.2	0.58	2.17	4.59	7.6	10.05	11.86	14.76	16.73	17.54	18.91	18.66	19.76	18.62	21.46	29.67	42.75	92.82
Belgium	Female	0.09	0.12	0.25	0.99	1.89	2.66	3.81	4.92	5.75	5.89	6.7	8.23	9.41	11.21	14.49	18.13	23.87	37.48	63.4	58.81
Belize	Male	0.51	0.36	0.46	0.75	1.88	3.26	5.86	10.37	10.87	19.81	22.2	32.58	32.95	27.85	27.59	25.41	17.98	16.35	17.85	15.28
Belize	Female	0.19	0.25	0.59	0.7	1.85	3.51	9.86	10.83	9.54	18.69	17.81	23.47	20.08	20.14	14.66	14.03	10.35	12.03	8.05	13.98
Benin	Male	0.14	0.18	0.32	0.65	1.13	1.69	2.36	2.94	3.48	4.34	5.42	6.91	10.62	14.26	10.33	11.18	9.37	9.88	14.03	7.44
Benin	Female	0.58	0.19	0.35	0.82	0.9	1.23	1.92	2.76	3.93	13.22	5.22	6.13	3.46	5.81	7.46	20.47	7.56	8.14	5.19	50.12
Bermuda	Male	0.21	0.25	0.38	0.72	1.48	2.62	4.16	5.74	7.1	10.23	12.7	15.34	15.59	14.8	14.06	12.72	8.57	7.86	9.07	7.16
Bermuda	Female	0.18	0.24	0.45	0.61	1.36	2.17	4.38	6.54	6.86	10.53	10.04	12.51	11.9	13.52	11.52	12.23	12.72	19.08	15.23	28.57
Bhutan	Male	0.44	0.64	0.7	1.34	2.87	4.83	6.57	8.41	10.41	12.44	14.62	16.65	19.29	23.02	27.18	28.55	32.14	31.13	36.88	25.77
Bhutan	Female	0.49	0.36	0.8	1.23	2.75	4.17	5.84	7	7.76	9.41	9.61	12.59	10.71	15.37	19.04	31.68	20.09	21.59	19.78	23.07
Bolivia (Plurinational State of)	Male	0.24	0.18	0.31	0.76	2.77	5.1	10.05	13.2	15.07	19.02	19.77	19.7	24.24	22.13	29.72	22.85	21.46	25.79	40.6	34.57
Bolivia (Plurinational State of)	Female	0.1	0.15	0.26	0.71	2.32	3.08	5.71	9.41	11.47	14.08	15.56	17.51	14.2	14.83	11.02	13.69	21.74	8.52	24.28	15.07
Bosnia and Herzegovina	Male	0.32	0.5	0.76	1.33	2.24	3.2	4.06	4.8	5.4	6.42	8.11	8.91	9.33	10.59	14.33	13.7	9.61	7.98	7.65	5.38
Bosnia and Herzegovina	Female	0.34	0.62	1.17	2.34	3.73	4.24	4.44	4.65	5.9	5.57	6.33	7.45	7.75	8.35	8.21	7.41	6.93	8.73	11.89	4.8
Botswana	Male	0.34	0.5	0.76	1.54	2.32	3.33	4.11	4.85	6.11	6.7	8.63	10.23	10.1	14.56	13.85	13.71	9.88	7.69	21.7	8.33
Botswana	Female	1.67	0.52	0.43	0.58	1.05	1.82	2.56	4.03	4.18	5.1	5.56	5.76	4.34	4.88	4.85	4.45	4.84	4.94	7.5	4.33

Brazil	Male	0.42	0.24	0.52	1.04	2.34	3.96	6.17	7.96	11.02	13.21	16.97	21.37	24.23	27.89	31.81	31.68	33.41	28.09	36.15	29.19
Brazil	Female	0.14	0.42	0.6	2.23	4.38	6.42	8.71	10.88	13.36	16.71	17.55	20.85	21.33	20.44	21.65	24.59	26.99	33.23	27.14	35.21
Brunei Darussalam	Male	0.12	0.27	0.52	1.02	2.17	4.96	7.44	10.35	13.34	15.73	18.26	18.4	18.54	18.65	35.76	51.58	93.17	227.48	258.66	239.03
Brunei Darussalam	Female	0.47	0.26	1.16	0.57	1.3	1.15	2.06	2.22	4.09	6.96	9.16	15.64	12.35	28.25	29.79	43.1	52.44	79.73	89.11	57.97
Bulgaria	Male	0.33	0.51	0.78	1.31	2.46	3.38	4.07	4.91	5.92	7.81	8.93	11.19	12.29	15.7	14.78	14.79	13.08	11.18	14.84	12.96
Bulgaria	Female	0.35	0.63	1.18	2.43	3.71	4.3	4.65	5.12	5.4	6.96	8.57	9.75	10.44	11.21	11.36	10.1	8.38	7.12	6.59	6.93
Burkina Faso	Male	0.15	0.21	0.71	1.39	2.15	1.8	2.35	2.91	3.41	4.2	5.25	6.7	9.55	12.25	9.01	7.98	6.18	5.16	6.28	3.48
Burkina Faso	Female	0.66	0.21	0.31	0.85	0.87	1.22	1.86	2.68	4.04	24.36	5.72	6.32	3.4	5.44	8.58	58.51	8.84	9.12	6.52	271.71
Burundi	Male	0.15	0.27	0.26	2.06	0.79	1.17	1.56	1.98	2.17	2.67	3.14	3.89	4.24	4.89	4.21	4.21	5.05	3.06	4.81	7.76
Burundi	Female	0.3	0.38	0.45	3.81	0.97	1.25	1.89	2.85	4.31	46.78	5.51	5.12	3.42	4.09	5.94	105.11	3.97	4.8	2.16	517.31
Cabo Verde	Male	0.1	0.14	0.25	0.59	1.08	1.66	2.35	2.9	3.4	4.29	5.23	6.68	9.39	12.29	9.31	8.74	6.97	6.83	8.02	3.43
Cabo Verde	Female	0.12	0.16	0.29	0.53	0.82	1.24	1.88	2.54	3.18	4.72	3.83	3.89	2.95	3.32	3.8	5.3	3.06	3.44	1.91	7.61
Cambodia	Male	0.31	0.99	1.05	1.47	2.16	5.59	6.77	13.11	20.05	29.8	44.71	45.85	76.96	96.03	78.4	108.72	111.7	81.47	81.73	93.65
Cambodia	Female	0.27	0.84	0.7	0.93	1.92	2.35	4.9	5.64	8.47	15.89	10.59	22.05	32.21	42.12	35.91	32.57	35.84	34.84	40.74	32.46
Cameroon	Male	0.13	0.18	0.31	0.7	1.16	1.69	2.37	2.97	3.51	4.47	5.38	6.89	11.18	15.37	10.95	11.9	9.81	10.65	14.46	8.05
Cameroon	Female	0.38	0.21	0.35	0.85	0.91	1.24	1.97	2.86	4.18	15.35	5.45	6.67	3.71	6.25	8.19	22.76	8.34	9.27	5.55	51.04
Canada	Male	0.07	0.18	0.41	0.65	1.75	3.17	3.92	4.83	5.66	6.42	8.18	10.92	14.02	17.34	19.36	23.92	33.3	37.66	49.85	64.39
Canada	Female	0.08	0.13	0.34	1.02	2.48	3.32	4.01	5.02	5.2	6.68	7.15	7.97	10.19	10.58	13.31	15.44	22.51	23.69	35.85	48.82
Central African Republic	Male	0.41	0.72	0.78	1.56	1.34	2.47	4.9	7.17	10	16.53	18.94	33.07	27.31	38.09	27.86	30.9	42.52	16.6	21.82	19.88
Central African Republic	Female	1.88	0.81	0.8	0.82	1.49	2.03	4.09	5.52	7.25	11.38	10.98	14.05	11.59	15.11	14.39	19.04	14.77	14.26	13.55	16.2
Chad	Male	0.12	0.2	0.36	0.69	1.15	1.68	2.34	2.95	3.48	4.41	5.34	6.81	10.57	15.1	10.59	11.22	9.01	9	12.5	6.41
Chad	Female	0.72	0.21	0.38	0.85	0.91	1.23	1.94	2.84	4.07	15.57	5.79	7.2	3.74	6.75	8.66	22.93	8.57	8.89	5.32	47.01
Chile	Male	0.09	0.16	0.33	0.87	2.36	5.38	9.12	12.34	14.3	15.99	18.46	15.73	16.98	14.29	12.58	13.58	16.88	15.04	25.8	28.68
Chile	Female	0.05	0.11	0.31	0.82	2.2	4.02	7	7.21	8.56	10.03	11.28	11.32	10.19	9.09	7.63	7.76	10.29	8.89	6.15	1.8
China	Male	0.37	0.28	0.33	0.63	1.62	2.83	4.74	6.75	8.47	10.62	14.57	16.26	18.24	21.16	28.45	36.2	40.38	72.86	90.81	71.77
China	Female	0.13	0.25	0.22	0.39	0.86	1.29	1.94	2.67	3.68	5.28	7.37	8.34	9.32	10.04	12.19	13.04	14.9	16.7	19.32	25.82
Colombia	Male	0.2	0.22	0.39	0.53	1.44	2.89	4.12	5.19	7.44	7.15	7.71	8.54	8.87	10.15	10.01	9.53	9.43	6.98	6.65	4.45

Colombia	Female	0.33	0.63	0.55	0.5	1.07	1.82	3.33	6.21	7.09	8.29	11.06	10.78	11.4	8.34	6.97	7	5.84	7.68	8.32	2.14
Comoros	Male	0.11	0.2	0.22	1.13	0.69	1.09	1.49	1.9	2.08	2.55	3.01	3.63	4	4.52	3.93	3.87	4.78	2.97	4.96	8.11
Comoros	Female	0.27	0.32	0.46	3.15	0.99	1.25	1.89	2.89	4.59	51.09	5.64	5.08	3.46	4.2	6.9	124.28	4.68	5.71	2.51	658.93
Congo	Male	0.13	0.27	0.41	0.85	1.11	1.96	3.36	4.47	5.62	8.15	9.84	15.85	14.97	20.86	17.39	20.38	30.12	14.3	23.35	23.83
Congo	Female	0.21	0.27	0.45	0.62	1.07	1.59	2.97	4.26	5.55	8.28	7.85	9.8	8.3	10.35	9.92	13.23	10.19	11.1	11.22	15.88
Costa Rica	Male	0.16	0.23	0.35	0.55	1.25	1.99	3.2	4.39	5.69	5.68	5.71	6.21	5.87	6.83	6.22	7.63	6.39	5.81	6.41	3.91
Costa Rica	Female	0.13	0.21	0.34	0.36	0.48	1.1	1.62	2.69	3.76	5.59	6.86	7.25	7.07	5.44	3.79	4.65	3.26	3.16	3.67	2.23
Côte d'Ivoire	Male	0.13	0.17	0.32	0.68	1.17	1.69	2.34	2.93	3.46	4.44	5.46	6.92	10.8	15.35	10.63	12.09	9.94	10.61	14.86	8.39
Côte d'Ivoire	Female	0.36	0.19	0.33	0.82	0.9	1.24	1.96	2.8	3.97	13.45	5.12	6.17	3.49	5.86	7.35	20.56	7.95	8.81	5.32	55.23
Croatia	Male	0.21	0.33	0.59	0.95	1.59	2.64	4.2	6.06	8.68	11.39	11.63	12.94	13.55	16.87	17.13	14.51	24.53	20.6	28.98	28.33
Croatia	Female	0.22	0.34	0.7	1.59	2.66	3.21	3.71	5.01	6.71	8	10.33	10.96	11.8	13.26	17.35	24.19	26.57	24.24	10.62	10.98
Cuba	Male	0.15	0.23	0.38	0.72	1.43	2.59	5.03	9.52	11.61	22.09	27.26	37.08	46.59	44.92	47.13	52.4	26.13	25.07	28.93	22.89
Cuba	Female	0.12	0.18	0.47	0.57	1.8	2.88	6.34	8.39	9.47	16.5	14.27	21.99	22.48	25.25	18.21	15.32	11.43	10.68	7.96	2.3
Cyprus	Male	0.06	0.08	0.15	0.47	1.5	3.44	5.61	7.57	9.08	11.15	12.52	13.25	12.7	13.08	13.51	13.12	17.42	16.79	14.03	14.14
Cyprus	Female	0.05	0.07	0.13	0.38	1.02	1.87	2.75	4.86	3.93	4.65	5.03	6.84	5.5	7.76	6.31	12.08	19.59	26.35	18.56	61.16
Czechia	Male	0.34	0.53	0.87	1.48	2.83	3.56	4.57	5.38	5.83	7.34	8.8	11.22	13.83	16.21	18.13	17.63	21.76	21.81	26.93	28.54
Czechia	Female	0.36	0.65	1.2	2.45	4.01	4.71	4.73	5.29	5.93	6.93	8.49	11.25	12.22	16.41	16.86	18.33	20.73	21.49	21.44	15.7
Democratic People's Republic of Korea	Male	0.82	0.36	0.6	2.16	3.81	5.23	7.72	10.18	13.14	18.62	28.7	32.91	37.53	42.21	48.96	56.29	49.95	62.35	62.93	53.46
Democratic People's Republic of Korea	Female	0.21	0.32	0.35	0.88	1.7	2.88	4.21	4.96	6.85	11.06	15.57	16.65	16.8	17.69	17.56	17.23	16.38	16.9	16.53	18.93
Democratic Republic of the Congo	Male	0.15	0.34	0.44	0.91	1.12	1.95	3.42	4.58	5.84	8.35	9.9	16.46	15.26	20.61	16.91	20.32	29.03	12.9	18.51	21.1
Democratic Republic of the Congo	Female	0.28	0.37	0.5	0.62	1.03	1.47	2.65	3.6	4.52	6.7	6.81	8.25	7.12	9.22	8.72	11.33	8.98	8.76	8.75	13.13

Denmark	Male	0.06	0.08	0.15	0.47	1.51	3.75	5.77	7.71	9.36	13.24	14.59	17.18	21.41	23.53	32.94	37.37	56.28	97.81	98.57	112.07
Denmark	Female	0.04	0.07	0.14	0.42	1.58	1.9	3.47	4.47	3.83	4.34	6.34	7.37	9.6	10	15.28	19.74	22.73	35.98	39.07	93.06
Djibouti	Male	0.15	0.21	0.24	1.69	0.75	1.14	1.55	1.95	2.13	2.64	3.12	3.82	4.22	4.82	4.23	4.38	5.6	3.66	5.85	9.34
Djibouti	Female	0.48	0.34	0.45	2.57	0.94	1.25	1.9	2.93	4.81	50.53	6.25	5.32	3.52	4.36	7.38	147.82	5.27	6.93	2.92	759.42
Dominica	Male	0.34	0.29	0.43	0.72	1.52	2.73	4.44	6.57	7.78	10.92	14.27	18.84	17.3	17.31	18.76	15.77	10.9	10.16	11.95	10.19
Dominica	Female	0.13	0.19	0.54	0.58	1.24	1.98	3.86	5.23	5.54	7.87	6.62	7.76	7.97	7.6	5.67	5.02	4.01	4.23	2.91	4.75
Dominican Republic	Male	0.2	0.32	0.43	0.73	1.56	2.6	4.09	5.35	7.05	9.24	12.02	15.58	14.38	13.98	14.37	12	8.49	6.24	4.48	3.11
Dominican Republic	Female	0.12	0.2	0.35	0.55	1	1.67	2.93	4.18	4.58	5.4	5.49	5.98	5.66	4.27	3.81	2.59	1.93	2.19	1.61	1.74
Ecuador	Male	0.11	0.17	0.27	0.59	2.67	5.16	11.01	14.89	16.2	18.95	20.43	19.58	20.46	18.05	19.36	16.48	16.69	26.92	49.82	39
Ecuador	Female	0.1	0.13	0.27	0.98	2.89	3.83	6.38	10.3	12.2	13.97	14.77	15.47	12.67	10.8	6.68	7.25	8.36	4.27	19.74	15.63
Egypt	Male	0.15	0.33	0.63	1.05	1.93	3.4	5.44	7.31	8.64	9.91	10.61	10.5	9.02	7.53	5.84	4.95	3.52	2.27	2.16	1.1
Egypt	Female	0.15	0.33	0.57	0.8	1.14	1.65	2.67	3.82	5.08	5.96	7.33	7.73	6.84	5.92	4.99	3.67	2.51	1.99	1.64	1.35
El Salvador	Male	0.15	0.21	0.31	0.54	1.11	1.97	3.47	4.7	6.06	6.45	6.64	5.91	5.89	7.91	6.5	9.17	9.66	14.37	12.59	15.3
El Salvador	Female	0.13	0.21	0.27	0.31	0.46	0.84	1.58	2.69	3.75	4.95	5.89	5.59	5.12	4.5	4.16	4.34	3.28	4.5	4.31	1.77
Equatorial Guinea	Male	0.12	0.22	0.35	0.81	1.08	1.85	3.05	4.07	5.02	6.99	8.2	13.05	12.63	17.59	15.22	19.28	29.26	14.47	22.12	22.53
Equatorial Guinea	Female	0.16	0.23	0.38	0.58	0.95	1.41	2.49	3.42	4.35	6.38	6.1	7.36	6.46	8.52	8.71	12.64	10.75	10.72	11.09	14.89
Eritrea	Male	0.16	0.21	0.24	2	0.82	1.23	1.62	2.07	2.19	2.76	3.3	4.22	4.6	5.23	4.39	4.48	5.46	3.26	5.23	7.44
Eritrea	Female	0.35	0.27	0.41	2.85	0.95	1.24	1.89	2.93	4.78	63.21	6.08	5.67	3.54	4.27	6.67	133.37	4.72	6.35	2.75	610.86
Estonia	Male	0.23	0.31	0.57	1.54	4.29	8.57	14.99	23.57	29.85	34.21	40.76	43.56	45.31	53.42	49.48	59.41	66.03	73.02	75.63	88.78
Estonia	Female	0.15	0.24	0.57	1.67	3.68	6.05	7.24	9.8	10.93	15.52	18.81	25.1	31.8	32.77	34.26	32.29	40.95	33.51	39.15	45.42
Eswatini	Male	0.22	0.68	0.98	1.87	2.75	3.96	4.9	5.9	8.13	9.03	11.49	13.84	12.95	19.45	18.13	17.88	12.65	9.74	28.48	7.3
Eswatini	Female	0.5	0.48	0.41	0.58	1.03	1.76	2.49	3.81	4.11	5.07	5.65	5.66	4.41	4.9	4.91	4.67	5.03	5.18	8.5	3.72
Ethiopia	Male	0.14	0.22	0.26	1.47	0.83	1.25	1.7	2.1	2.25	2.77	3.39	4.08	4.34	4.88	4.26	4.43	5.72	3.27	5.2	7.99
Ethiopia	Female	0.39	0.34	0.49	3.2	1.13	1.49	2.23	3.27	4.82	42.64	6.1	5.92	4.07	4.9	7.48	155.72	5.43	6.91	2.98	735.46
Fiji	Male	0.13	0.28	0.4	0.61	1.2	2.55	4.27	5.78	8.78	9.49	12.4	14.85	15.3	9.41	7.92	15.25	35.15	11.37	31.61	4.74
Fiji	Female	0.22	0.11	0.48	0.35	0.61	1.11	2.34	4.65	5.27	4.75	5.81	8.02	5.77	10.5	8.27	8.34	1.97	2.06	2.48	2.52
Finland	Male	0.06	0.08	0.15	0.47	1.49	3.41	5.87	7.76	9.52	11.01	12.59	14.46	14.04	12.8	11.79	15.32	14.32	25.94	24.27	25.66

Finland	Female	0.04	0.06	0.13	0.38	1.01	1.84	2.69	3.33	3.93	4.26	5.05	5.32	5.8	4.91	7.95	9.48	9.3	8.19	10.81	7.8
France	Male	0.07	0.09	0.2	0.56	1.82	4.01	6.82	9.11	10.93	13.41	14.89	16.36	16.04	15.17	14.35	13.09	14.44	17.98	23.76	24.97
France	Female	0.05	0.07	0.14	0.43	1.3	2.28	3.41	4	4.85	5.7	6.93	7.1	7.18	7.91	8.42	9.15	11.77	16.35	20.19	20.08
Gabon	Male	0.14	0.25	0.42	0.94	1.16	2.01	3.48	4.77	6.11	9.12	10.77	17.57	17.31	24.25	20.59	24.54	36.6	17.53	26.1	26.96
Gabon	Female	0.19	0.28	0.44	0.6	1.01	1.49	2.68	3.82	5.04	7.71	7.35	9.24	8.12	10.79	11.04	16.3	14.23	15.1	16.29	23.19
Gambia	Male	0.12	0.16	0.29	0.68	1.17	1.69	2.35	2.93	3.47	4.39	5.37	6.91	11.17	16.24	11.28	12.24	9.88	11.25	15.76	7.89
Gambia	Female	0.29	0.18	0.32	0.76	0.88	1.24	1.93	2.83	4.16	15.88	5.7	7.22	3.67	6.57	8.32	23.85	9.01	11.43	6.71	57.32
Germany	Male	0.06	0.08	0.16	0.49	1.59	3.61	5.97	8.51	10.36	12.47	14.37	15.07	15.42	15.45	15.09	15.52	15.1	16.71	21.98	17.76
Germany	Female	0.04	0.08	0.14	0.57	1.23	1.91	3.17	3.95	4.49	4.97	6.37	7.57	7.34	8.19	10.24	11.13	15.91	16.54	16.9	12.21
Ghana	Male	0.84	0.47	0.92	2.81	3.87	2.5	2.72	4.03	5.74	10.81	16.1	24.94	57.38	98.4	65.71	90.1	92.7	138.77	204.68	144.22
Ghana	Female	1.08	0.2	0.28	0.67	0.84	1.22	1.84	2.55	3.52	14.85	5.32	6.41	5.01	7.45	9.26	28.47	10.35	12.56	7.92	87.61
Greece	Male	0.07	0.09	0.17	0.52	1.69	3.84	6.3	8.64	10.4	12.56	14.33	14.7	14.65	13.82	11.78	9.35	7.15	6.08	5.24	3.81
Greece	Female	0.05	0.07	0.14	0.44	1.16	2.06	3.04	3.73	4.36	4.74	5.25	5.47	5.09	4.72	4.37	3.45	3.19	3.06	2.87	2.61
Greenland	Male	0.08	0.14	0.27	0.64	1.36	2.26	3.27	4.05	4.63	5.42	6.51	7.73	9.07	10.47	11.07	10.59	10.53	10.15	13.39	9.12
Greenland	Female	0.07	0.13	0.33	1	2.13	3.06	3.66	3.95	3.91	4.24	4.51	4.85	5.08	5.72	6.05	6.11	5.79	5.21	3.79	2.53
Grenada	Male	0.72	0.61	0.56	0.77	2.09	3.33	6.73	14.28	15.12	32.28	36	57.16	68.91	82.52	93.82	94.18	56.5	52.78	60.24	57.29
Grenada	Female	0.18	0.23	0.58	0.66	1.8	2.74	7.33	11.36	9.02	14.06	13.73	20.1	19.59	22.44	16.3	14.06	11.96	14.25	9.17	13.86
Guam	Male	0.11	0.15	0.26	0.52	1.19	2.45	4.29	5.92	8.12	9.18	11.11	12.6	12.1	8.99	7.2	7.28	10.99	3.87	8.41	1.6
Guam	Female	0.12	0.1	0.35	0.33	0.61	1.12	2.2	3.82	4.58	4.7	5.6	6.87	5.66	8.06	5.77	6.29	1.77	1.68	1.55	1.37
Guatemala	Male	0.43	0.46	0.34	0.54	1.06	2.87	5.78	5.21	8.55	7.39	7.75	11.29	13.89	11.76	13.03	17.34	13.39	10.71	7.33	4.48
Guatemala	Female	0.7	0.59	0.41	1.21	1.6	2.72	2.43	5.31	8.24	9.94	11.35	12.58	16.44	16.36	12.5	9.87	9.97	17.35	31.08	30.93
Guinea	Male	0.14	0.21	0.37	0.7	1.18	1.69	2.34	2.97	3.54	4.46	5.42	7.05	11.24	15.97	10.88	11.89	9.77	10.05	14.05	7.57
Guinea	Female	0.81	0.22	0.38	0.91	0.93	1.24	1.97	2.89	4.21	15.99	5.74	7.1	3.77	6.59	8.43	22.14	8.23	8.74	5.03	47.59
Guinea-Bissau	Male	0.11	0.17	0.35	0.75	1.23	1.71	2.34	2.97	3.56	4.74	5.76	7.6	12.73	18.71	11.88	13.32	10.47	11.3	15.15	7.98
Guinea-Bissau	Female	0.31	0.18	0.35	0.92	0.96	1.25	2.08	3.25	5.25	22.89	6.68	8.79	3.96	7.42	9.75	26.24	9.57	11.12	6.41	51.64
Guyana	Male	0.59	0.61	0.6	0.76	2.2	3.61	7.12	14.88	15.46	31.79	34.73	49.58	52.24	46.74	48.36	46.61	26.72	24.32	29.78	27.71
Guyana	Female	0.21	0.29	0.83	0.72	2.42	3.94	10.27	14.82	12.48	22.02	19.27	26.75	23.45	24.96	17.82	14.55	11.24	12.5	10.3	18.53
Haiti	Male	6.28	2.08	0.99	0.77	1.81	2.93	5.05	9	12.2	23.49	29.63	48.44	49.04	49.34	56.23	58.98	38.8	35.74	35.3	26.47
Haiti	Female	0.5	0.63	1.45	0.73	2.28	3.51	10.1	14.54	13.03	18.35	16.64	25.83	23.66	24.35	18.71	16.25	13.4	14.12	7.98	12.46
Honduras	Male	0.27	0.34	0.6	0.67	1.5	2.48	4.88	6.64	11.93	10.92	15.35	21.38	27.69	42.12	43.84	53.12	54.16	58.81	49.18	19.72
Honduras	Female	0.14	0.29	0.43	0.56	0.9	1.75	2.89	5.76	7.51	9.62	15.17	14.15	11.39	10.18	11.61	10.84	8.49	7.84	10.27	5.82

Hungary	Male	0.33	0.53	1	1.54	2.7	3.75	4.77	5.79	6.54	9.15	11.92	21.06	23.53	36.2	44.23	58.57	65.09	67.31	73.32	85.79
Hungary	Female	0.35	0.66	1.34	2.58	4.05	4.83	5.42	6.26	7.01	8.27	14.13	17.3	23.5	35.88	39.96	52.31	52.99	56.42	53.81	52.73
Iceland	Male	0.08	0.13	0.21	0.55	1.78	3.71	6	8.38	10.07	12.69	14.43	16.13	17.54	19.2	22.87	27.68	32.89	41.52	53.81	73.22
Iceland	Female	0.04	0.08	0.15	0.49	1.28	2.09	3.03	3.77	4.43	4.81	5.84	6.49	6.92	7.68	9.27	11.39	13	15.84	20.8	24.79
India	Male	0.44	0.82	0.96	1.71	3.81	6.49	9.36	11.65	13.95	16.41	19.52	23.19	25.76	29.91	36.42	37.49	41.59	35.2	45.49	30.75
India	Female	0.35	0.47	1.02	1.7	3.99	5.76	7.74	8.82	9.24	11.05	12.75	15.26	12.46	17	18.08	25.39	16.21	13.84	14.11	17.84
Indonesia	Male	0.29	0.83	1.12	1.23	1.79	4.49	5.31	11.02	19.94	31.87	46.16	47.21	94.05	117.45	93.57	121.15	118.68	79.1	85.51	110.84
Indonesia	Female	0.15	0.52	0.54	0.79	1.47	1.49	2.76	3.33	6.22	9.15	7.15	13.13	20.6	24	17.01	15.26	14.8	15.71	21	18.79
Iran (Islamic Republic of)	Male	0.27	0.61	0.97	1.31	2.43	4.08	6.15	8.2	9.56	10.68	11.61	11.8	10.39	9.3	7.62	9.01	9.11	7.21	9.68	8.42
Iran (Islamic Republic of)	Female	0.22	0.54	0.96	1.13	1.52	2.07	3.18	4.32	5.76	6.72	8.08	8.68	8	7.83	7.62	7.96	7.86	7.81	6.74	2.36
Iraq	Male	0.37	0.79	0.69	1.06	1.93	3.64	5.98	7.94	9.53	11.21	11.83	12.2	10.28	9.19	8.23	9.06	9.1	9.25	20.19	4.69
Iraq	Female	0.17	0.34	0.56	0.8	1.2	1.66	2.64	3.92	5.33	5.92	7.4	8.25	7.08	6.78	5.6	4.44	3.64	2.27	2.16	4.05
Ireland	Male	0.06	0.25	0.21	0.59	2.01	4.22	6.8	8.7	10.63	13.05	15.1	15.73	15.65	14.97	14.04	14.24	12.97	18.18	26.35	28.24
Ireland	Female	0.07	0.07	0.16	0.43	1.14	2.07	3.06	4.77	4.84	4.87	7.09	6.25	7.42	8.94	13.49	13.75	15.58	18.2	22.78	25.15
Israel	Male	0.07	0.1	0.2	0.53	1.69	3.81	6.44	9.09	11.08	14.63	15.17	17.33	19.54	19.54	21.23	31.86	41.3	49.09	60.28	84.26
Israel	Female	0.06	0.08	0.18	0.45	1.15	2.09	3.07	3.76	4.25	5.14	6	7.31	8.67	9.47	11.28	22.04	24.12	36.59	49.08	74.66
Italy	Male	0.12	0.2	0.29	0.59	1.6	3.28	5.74	8.71	11.3	14.38	17.37	19.03	18.55	18.07	16.89	15.9	16.07	20.59	27.12	34.56
Italy	Female	0.09	0.17	0.26	0.68	1.61	2.52	3.5	4.51	5.39	6.58	7.77	8.8	8.53	8.41	8.41	9.18	10	10.48	11.65	14.93
Jamaica	Male	0.15	0.29	0.52	0.75	1.67	3.26	4.08	5.28	6.49	8.64	13.51	23.84	28.82	32.22	32.48	33.81	31.14	13.95	8.11	25.52
Jamaica	Female	0.11	0.24	0.92	0.61	1.06	1.69	3	7.79	6.7	5.41	5.34	5.37	5.35	15.71	8.64	4.04	10.39	14.03	4.14	23.26
Japan	Male	0.14	0.27	0.45	0.78	2.03	4.39	6.96	10.75	14	16.54	18.65	17.99	17.1	16.44	17.61	19.54	21.76	30.34	31.87	34.94
Japan	Female	0.19	0.23	0.37	0.65	1.1	1.28	1.73	2.36	4.02	6.1	7.83	10.15	9.41	11.88	12.67	19.06	28.96	41.53	57.07	97.21
Jordan	Male	0.2	0.52	0.92	1.45	2.41	4.5	7.95	11.17	13.33	14.3	14.34	13.47	11.02	9.37	7.51	6.72	5.82	3.97	4.05	2.39
Jordan	Female	0.24	0.6	1.03	1.16	1.13	1.42	2.75	4.59	6.36	8.25	11.66	13.04	11.79	10.39	7.61	6.02	4.27	2.5	2.35	2.06
Kazakhstan	Male	0.31	0.5	0.84	1.74	4	8.5	14.15	8.58	17.8	37.14	42.8	41.7	85.49	172.12	141.28	215.45	246.91	163.64	66.05	6.93
Kazakhstan	Female	0.21	0.35	0.72	1.53	2.51	4.95	7.26	4.84	9.85	12.46	32.82	43.9	54.51	101.67	96.06	124.83	158.69	80.93	82.67	123.11
Kenya	Male	0.1	0.17	0.24	1.48	0.82	1.23	1.69	2.11	2.27	2.84	3.44	4.16	4.37	4.88	4.21	4.19	5.25	3.08	4.81	7.21
Kenya	Female	0.23	0.26	0.41	2.3	1.08	1.49	2.23	3.29	4.92	50.52	6	5.82	4.02	4.76	6.96	125.31	4.77	6.38	2.89	668.41
Kuwait	Male	0.16	0.34	0.69	1.04	1.94	3.43	5.43	7.29	8.84	9.61	10.04	10.24	8.6	7.47	6.27	6.13	5.64	5.52	10.52	4.64

Kuwait	Female	0.16	0.44	0.62	0.8	1.12	1.63	2.59	3.7	4.9	6.09	7.14	7.76	6.89	5.63	4.85	3.67	3.64	2.62	2.96	2.04
Kyrgyzstan	Male	1.55	4.11	2.52	2.81	5.98	11.54	13.92	14.61	21.46	26.87	29.53	29.36	30.52	41.33	23.99	27.22	11.84	22.5	31.44	24.52
Kyrgyzstan	Female	0.47	0.44	0.8	1.72	2.76	4.02	6.36	5.9	8.44	9.42	10.83	10.91	14.16	13.24	11.81	10.91	9.43	4.06	3.75	3.28
Lao People's Democratic Republic	Male	0.29	1.25	1.24	1.39	2.13	5.61	6.71	12.95	18.78	26.93	39.03	37.43	60.99	75.22	63.15	84.19	91	68.34	70.54	83.76
Lao People's Democratic Republic	Female	0.22	1.01	0.82	0.91	2.03	2.49	5.18	5.71	8.68	14.65	10	19.67	28.68	36.69	30.3	28.35	30.92	27.2	32.31	25.98
Latvia	Male	0.21	0.3	0.56	1.52	4.15	8.27	13.99	23.1	31.62	37.56	46.46	56.67	62.78	122.33	133.72	176.24	197.27	181.41	213.3	176.03
Latvia	Female	0.14	0.23	0.56	1.56	3.43	5.26	6.73	10.57	10.22	22.68	27.28	40	51.49	60.02	61.24	80.37	93.59	74.69	96.67	64.61
Lebanon	Male	0.16	0.3	0.57	1.05	1.94	3.41	5.35	7.15	8.48	9.48	9.98	9.87	8.7	7.09	5.7	4.91	3.83	2.76	3.32	1.92
Lebanon	Female	0.15	0.31	0.54	0.81	1.15	1.66	2.64	3.84	5.16	6.19	7.3	8.12	7.24	6.17	5.22	4.17	3.32	2.86	2.68	1.82
Lesotho	Male	0.24	0.77	1.07	2.07	2.82	4.02	5	6.01	8.65	9.73	13.53	15.84	15.17	22.75	19.8	18.55	13.37	10.25	29.64	7.27
Lesotho	Female	0.77	0.59	0.45	0.61	1.19	2.24	2.99	4.94	5.02	6.6	7.24	7.87	5.61	6.5	6.38	6.02	6.71	6.76	10.83	4.71
Liberia	Male	0.11	0.16	0.29	0.63	1.12	1.68	2.33	2.93	3.44	4.33	5.3	6.49	9.93	13.26	9.65	9.65	8.51	9.03	12.86	6.89
Liberia	Female	0.24	0.19	0.35	0.87	0.94	1.25	1.97	2.88	4.23	16.26	5.65	7.25	3.72	6.68	8.47	25.3	9.32	11.04	6.14	58.82
Libya	Male	0.17	0.33	0.59	1.06	1.93	3.37	5.26	7.44	8.8	9.63	10.3	10.19	8.87	7.39	5.89	5.53	4.28	3.3	4.19	2.54
Libya	Female	0.18	0.36	0.58	0.83	1.19	1.7	2.76	4.02	5.68	6.56	7.6	8.43	7.57	7.03	6.07	5.64	3.99	3.58	3.32	2.35
Lithuania	Male	0.23	0.32	0.61	1.59	4.67	9.09	15.07	22.69	30	36.27	44.47	49.31	58.66	71.68	73.27	68.95	73.67	51.46	54.64	82.78
Lithuania	Female	0.16	0.28	1.49	1.84	4.53	5.88	8.69	14.13	13.31	19.56	26.38	36.25	42.52	55.58	56.41	59.08	76.65	60.14	49.33	38.4
Luxembourg	Male	0.07	0.11	0.21	0.61	2.02	4.44	7.34	10.16	12.24	14.46	16.18	17.31	17.34	16.65	16.31	15.07	14.98	17.78	22.31	26.13
Luxembourg	Female	0.05	0.07	0.15	0.53	1.44	2.42	3.54	4.3	4.85	5.62	6.11	6.4	6.46	6.96	7.4	7.45	7.59	7.57	9.03	10.1
Madagascar	Male	0.1	0.17	0.2	1.33	0.7	1.1	1.49	1.9	2.11	2.56	3.01	3.61	3.96	4.33	3.74	3.42	3.91	2.43	3.82	6.24
Madagascar	Female	0.21	0.3	0.44	3.37	0.98	1.25	1.89	2.9	4.63	50.61	5.71	5.37	3.47	4.12	6.18	103.84	4.01	4.96	2.21	487.27
Malawi	Male	0.14	0.19	0.22	2.06	0.83	1.18	1.58	2	2.14	2.68	3.16	3.98	4.39	5.24	4.53	4.78	6.33	3.62	5.57	8.92
Malawi	Female	0.34	0.27	0.42	2.84	0.95	1.25	1.88	2.85	4.3	38.67	5.52	5.03	3.44	4.2	6.81	138.68	5	6.18	2.71	670.57
Malaysia	Male	0.11	0.16	0.26	0.54	1.28	2.92	5.21	7.89	11.03	14.41	17.9	19.47	22.07	25.09	21.3	29.6	22.41	18.81	18.1	26.7
Malaysia	Female	0.07	0.2	0.17	0.31	0.61	1.14	1.86	2.41	3	4.89	5.11	10.37	9.99	20.57	19.98	20	18.95	16.01	26.56	23.6
Maldives	Male	0.12	0.16	0.27	0.54	1.3	2.86	5.22	7.76	10.65	14.07	17.18	18.32	19.12	19.53	17.02	15.59	14.26	12.68	14.6	19.86
Maldives	Female	0.07	0.11	0.17	0.3	0.59	1.12	1.84	2.37	2.85	3.88	4.81	5.28	5.35	5.46	4.92	4.59	5.11	5.11	6.35	5.78

Mali	Male	0.17	0.19	0.33	0.64	1.11	1.68	2.35	2.92	3.45	4.35	5.33	6.83	10	13.57	10.22	9.91	8.31	8.37	11.04	5.84
Mali	Female	1.25	0.22	0.4	0.96	0.93	1.24	1.99	2.95	4.43	18.37	6.21	8.13	4.03	7.52	10.13	28.01	10.81	11.39	6.37	59.05
Malta	Male	0.11	0.19	0.33	0.7	2.13	4.53	7.4	10.44	12.29	15.15	17.11	18.63	19.76	21.44	23.66	25.07	29.28	36.08	43.02	50.03
Malta	Female	0.07	0.11	0.18	0.63	1.58	2.43	3.7	4.45	5.38	5.94	7.32	8.1	9.49	11	13.69	16.18	18.04	18.81	21.14	21.18
Marshall Islands	Male	0.14	0.52	0.61	0.71	1.27	3.12	4.43	6.05	16.8	11.79	17.53	23.7	24.55	12.21	9.88	21.85	48.8	13.39	27	4.4
Marshall Islands	Female	0.33	0.11	1.2	0.52	0.67	1.24	6.01	19.6	20.85	8.9	12.16	27.83	8.96	35.02	27.82	32.45	5.05	7.15	8.99	9.27
Mauritania	Male	0.1	0.16	0.26	0.59	1.08	1.67	2.34	2.92	3.42	4.21	5.13	6.48	9.39	12.37	9.64	8.77	7.33	7.98	10.32	5.55
Mauritania	Female	0.17	0.19	0.34	0.78	0.91	1.24	1.96	2.81	4.19	15.86	5.89	7.67	3.96	7.57	10.52	30.69	12.19	14.56	8.29	71.45
Mauritius	Male	0.12	0.19	0.3	0.54	1.29	2.82	5.16	7.44	10.16	13.66	16.86	17.7	18.95	18.86	15.88	13.21	11.63	6.72	7.94	7.01
Mauritius	Female	0.08	0.14	0.5	0.34	0.71	1.19	2.02	2.48	3.18	4.15	4.85	5.31	6	5.94	4.48	3.57	3.22	2.94	3.2	2.25
Mexico	Male	0.5	0.45	0.81	1.14	3.04	5.2	8.5	12.04	19.06	19.81	26.94	32.45	34.63	40.61	38.92	46.92	43.38	42.03	48.28	35.28
Mexico	Female	0.26	0.5	0.77	1	2.12	3.59	6.25	9.85	15.31	19.52	31.56	35.76	32.3	29.93	27.14	26.76	25.36	26.81	33.71	24.44
Micronesia (Federated States of)	Male	0.13	0.37	0.5	0.71	1.27	3.15	4.44	6.06	15.78	11.77	18.5	24.64	24.65	12.07	10.16	22.14	48.05	13.14	26.48	4.24
Micronesia (Federated States of)	Female	0.25	0.11	1.14	0.54	0.7	1.26	6.49	17.54	16.3	8.89	14.1	31.52	9.69	39.86	31.73	38.84	5.95	8.63	11.29	11.81
Mongolia	Male	0.29	0.52	0.87	1.77	4.04	6.62	9.57	10.7	9.48	11.42	12.64	14.49	15.75	16.58	15.16	13.99	9.36	9.96	11.65	6.76
Mongolia	Female	0.18	0.4	0.89	1.62	2.51	3.24	4.01	4.67	6.05	13.64	8.28	9.03	18.93	8.98	7.46	6.34	5.56	5.03	6.9	7.13
Montenegro	Male	0.32	0.5	0.75	1.31	2.22	3.2	4.07	4.84	5.22	6.04	7.18	7.95	8.38	9.1	9.04	8.19	6.63	5.93	4.93	3.59
Montenegro	Female	0.34	0.62	1.14	2.35	3.73	4.22	4.38	4.53	4.73	5.13	6	6.75	7.28	7.5	7.16	5.54	4.55	3.31	2.24	1.59
Morocco	Male	0.16	0.39	0.64	1.05	1.92	3.39	5.42	7.48	8.59	9.73	10.2	10.23	8.94	7.78	6.15	6.01	5.27	4.26	5.36	3.32
Morocco	Female	0.17	0.42	0.68	0.85	1.18	1.71	2.77	4.09	5.72	6.43	7.62	8.46	7.52	7.06	6.3	6.06	5.13	4.64	4	2.99
Mozambique	Male	0.14	0.23	0.25	2.59	0.89	1.28	1.65	2.13	2.27	2.88	3.37	4.36	4.83	5.83	4.95	5.68	7.6	4.53	6.76	10.63
Mozambique	Female	0.39	0.34	0.44	4.21	1.01	1.26	1.9	2.94	4.88	57.04	6.15	5.49	3.54	4.34	7.18	152.26	5.32	7.52	3.19	792.59
Myanmar	Male	0.64	1.17	1.27	1.72	2.32	6.01	7.06	13.84	20.89	29.74	43.67	42.48	68.8	83.49	71.47	97.39	104.07	73.02	71.95	85.95
Myanmar	Female	0.39	1.07	0.91	1.02	2.03	2.46	5	5.45	7.88	14.08	9.37	18.15	25.93	34.84	29.51	28.33	33.42	30.01	34.1	28.08
Namibia	Male	0.17	0.41	0.66	1.44	2.28	3.22	3.93	4.67	5.74	6.54	8.48	10.13	10.13	14.66	14.36	14	10.31	7.81	22.26	6.12
Namibia	Female	0.34	0.32	0.36	0.55	0.88	1.42	2.13	3.18	3.42	4.12	4.48	4.56	3.68	4.09	4	3.75	4.06	4.03	5.95	2.75
Nepal	Male	0.24	0.58	0.71	1.33	2.85	4.8	6.76	8.41	10.29	12.3	14.8	17.63	21.11	25.29	29.55	30.1	32.72	32.36	43.59	26.8

Nepal	Female	0.2	0.37	0.81	1.27	2.84	4.29	6.17	7.42	8.3	10.66	10.84	13.96	11.87	16.93	19.89	32.68	19.95	21.53	21.62	25.2
Netherlands	Male	0.07	0.09	0.2	0.63	1.98	4.03	6.76	9.14	11.29	13.97	16.04	18.47	18.68	20.16	22.51	24.12	38.81	51.32	63.03	81.06
Netherlands	Female	0.04	0.07	0.16	0.56	1.35	2.32	3.54	4.53	4.76	5.72	7.71	8.38	11.86	12.35	17.12	22.84	31.48	38.71	45.36	59.86
New Zealand	Male	0.07	0.11	0.15	0.46	1.43	4.3	6.27	8.09	10.1	13.95	15.43	19.31	19.12	27.08	26.84	33.13	37.71	54.34	39.92	45.23
New Zealand	Female	0.04	0.08	0.15	0.4	1.19	2.29	5.85	7.15	7.94	11.69	10.76	20.32	17.45	22.88	22.29	29.21	25.72	30.12	25.81	19.16
Nicaragua	Male	0.34	0.23	0.34	0.55	1.07	1.97	3.4	4.58	6.42	6.73	7.89	8.49	9.88	14.03	15	14.11	14.21	22.88	23.74	8.37
Nicaragua	Female	0.15	0.26	0.33	0.32	0.47	1.08	1.95	3.2	4.02	7.33	10.95	12.49	8.09	7.32	10.11	4.96	8.51	5.1	5.66	10.5
Niger	Male	0.14	0.18	0.33	0.62	1.1	1.67	2.35	2.95	3.47	4.33	5.29	6.7	9.94	13.43	9.96	9.89	8.46	8.13	11.11	5.65
Niger	Female	1.1	0.2	0.37	0.86	0.92	1.23	1.95	2.82	4.07	14.88	5.67	7.07	3.75	6.77	8.75	24.41	8.94	9.59	5.77	52.54
Nigeria	Male	0.17	0.22	0.38	0.73	1.25	1.88	2.64	3.27	3.72	4.77	5.76	7.42	10	12.74	9.58	9.79	7.98	8.39	11.89	6.41
Nigeria	Female	0.76	0.21	0.36	0.74	1	1.44	2.13	2.85	3.66	9.93	4.78	5.49	3.66	5.18	6.15	16.84	6.3	7.29	4.45	42.4
North Macedonia	Male	0.32	0.5	0.76	1.32	2.23	3.2	4.06	4.79	5.39	6.33	7.03	8.09	8.76	9.27	9.04	8.08	6.63	5.88	4.85	3.56
North Macedonia	Female	0.34	0.63	1.16	2.37	3.7	4.28	4.36	4.57	4.73	5.27	6.11	6.92	7.33	7.28	6.96	5.52	4.52	3.38	2.42	1.68
Northern Mariana Islands	Male	0.12	0.16	0.26	0.52	1.19	2.46	4.24	5.84	8.22	9.3	11.17	12.99	12.94	9.11	7.45	9.32	16.67	5.23	10.11	2.36
Northern Mariana Islands	Female	0.16	0.1	0.4	0.34	0.61	1.15	2.31	4.2	5.13	4.87	6.04	8.53	5.73	11.13	10.28	14.97	2.91	3.74	4.28	4.34
Norway	Male	0.16	0.24	0.44	1.18	3.51	6.68	11.64	16.06	18.99	22.81	26.04	28.76	27.18	28.47	31.15	37.77	43.12	53.48	58.56	88.69
Norway	Female	0.11	0.18	0.32	0.8	1.85	3.32	4.82	5.55	6.24	7.71	8.37	10.64	12.78	13.33	16.65	25.25	26.14	30.87	38.93	47.09
Oman	Male	0.18	0.41	0.75	1.1	1.95	3.41	5.34	7.4	8.55	9.49	10.25	10.04	9.31	8.18	7.08	9.03	12.01	9.4	13.62	8.27
Oman	Female	0.2	0.4	0.67	0.87	1.19	1.67	2.67	3.85	5.21	6.18	7.53	8.48	7.57	7.62	8.28	8.5	8.12	5.84	5.18	3.29
Pakistan	Male	1	1.95	1.26	1.65	3.38	5.82	7.98	10.32	13.81	16.14	19.33	22.09	25.69	30.07	38.12	38.29	42.88	41.05	54.68	41.24
Pakistan	Female	0.91	1.21	1.84	1.61	3.52	5.06	7.98	10.06	10.65	13.38	13.41	18.6	15.53	22.3	26.28	39.65	22.75	22.93	23.15	30.05
Palestine	Male	0.17	0.45	0.73	1.07	1.95	3.39	5.21	7.32	8.47	9.57	10.24	10.49	9.5	9.39	7.47	8.17	9.24	9.8	27.25	13.61
Palestine	Female	0.17	0.62	0.74	0.86	1.21	1.67	2.66	3.86	5	6.06	7.25	8.15	7.49	7.45	6.81	6.91	5.37	4.52	3.6	3.15
Panama	Male	0.16	0.24	0.43	0.55	1.07	1.97	3.19	4.18	5.3	5.84	6	5.73	6.06	5.58	6.05	7.68	7.49	7.2	9.13	3.91
Panama	Female	0.14	0.23	0.31	0.33	0.49	0.85	1.58	2.57	4.3	4.99	5.91	6.01	4.7	4.1	4.45	4.4	4.14	4.24	5.39	2.43
Papua New Guinea	Male	0.31	0.8	0.81	0.68	1.22	2.78	4.32	5.88	12.16	10.51	14.36	18.55	19	10.53	8.98	17.18	35.16	9.51	17.25	3.07

Papua New Guinea	Female	2	0.17	1.82	0.59	0.68	1.23	5.07	13.5	15.48	7.7	10.56	20.79	8.67	28.83	22.43	24.96	4.39	5.68	6.7	7.04
Paraguay	Male	0.1	0.15	0.29	0.62	1.58	2.63	4	5.04	6.44	8.29	10.87	14.57	15.23	17.6	12.27	14.99	9.88	13.23	24.61	6.85
Paraguay	Female	0.07	0.36	0.3	0.81	1.7	3.16	4.51	6.45	7.99	11.39	10.01	11.36	9.83	12.18	8.64	8.38	10.76	7.59	3.4	2.88
Peru	Male	0.17	0.17	0.3	0.75	2.42	5.1	9.43	12.59	14.31	16.32	15.86	14.64	13.9	12.7	12.72	9.5	7.57	7.24	10.47	13.84
Peru	Female	0.08	0.12	0.25	0.65	1.71	3.02	4.95	6.89	8.22	9.54	10.44	10.5	8.42	7.52	6.12	5.08	8.32	4.21	10.89	7.42
Philippines	Male	0.72	2.42	1.8	2.27	4.6	11.13	13.58	27.84	39.39	55.25	77.88	75.96	110.56	140.07	124.51	159.73	167.78	104.78	89.01	96.92
Philippines	Female	0.47	1.5	1.34	1.56	3.79	4.51	9.68	10.61	14.65	24.83	16.78	30.48	40.49	50.69	44.96	43.41	50.05	50.73	75.82	68.1
Poland	Male	0.31	0.4	0.57	0.94	1.69	2.33	2.83	3.49	4.01	5.33	6.03	7.63	8.32	10.15	10.86	11.09	10	10.08	10.61	10.32
Poland	Female	0.35	0.51	0.91	1.91	3.13	3.33	3.23	3.68	4.05	4.48	5.98	6.91	7.42	8.35	8.35	7.74	6.63	5.71	6.3	5.6
Portugal	Male	0.15	0.09	0.21	0.53	1.67	3.03	3.83	7.09	9.95	11.35	14.01	16	14.7	14.88	14.17	15.82	17.5	21.02	24.67	21.4
Portugal	Female	0.06	0.08	0.14	0.44	1.67	2.49	2.93	4.84	7.34	6.79	8.19	8.34	7.25	9.82	12.29	12.76	19.94	20.05	28.14	39.19
Puerto Rico	Male	0.15	0.32	0.41	0.73	1.43	2.56	4.05	5.28	7.22	10.09	10.88	14.26	14.71	11.89	10.39	9.85	9.63	10.86	5	2.58
Puerto Rico	Female	0.12	0.18	0.33	0.55	1.01	1.68	3.07	3.61	4.33	5.33	6.02	7.02	7.82	6.26	4.98	4.17	4.28	6.37	2.71	10.5
Qatar	Male	0.16	0.32	0.61	1.05	1.92	3.38	5.31	7.31	8.54	9.46	9.95	9.5	8.26	6.76	5.29	4.68	4.66	5.4	8.38	4.99
Qatar	Female	0.16	0.32	0.51	0.79	1.13	1.65	2.62	3.81	4.92	5.77	7.1	7.79	7.28	7.12	7.38	8.56	11.45	12.95	7.23	10.12
Republic of Korea	Male	0.1	0.19	0.35	0.79	1.92	3.95	6.64	9.7	12.79	15.26	16.62	15.6	13.53	11.58	13	15.57	20.49	37.1	55.92	68.92
Republic of Korea	Female	0.15	0.16	0.37	0.52	0.93	1.09	1.56	2.12	3.34	5.09	6.41	7.64	6.83	7.85	8.9	13.13	19.88	28.44	35.39	28.98
Republic of Moldova	Male	0.23	0.33	0.59	1.63	5.09	9.36	15.48	24.09	32.73	39.12	47.91	54.64	63.05	69.2	77.84	60.14	40.37	42.89	56.52	40.24
Republic of Moldova	Female	0.16	0.27	0.64	1.68	3.69	6.87	9.03	13.88	13.74	24.56	28.91	39	48.01	51.09	53.51	37.13	31.77	28.24	18.71	19.68
Romania	Male	0.33	0.51	0.76	1.32	2.25	3.22	4.07	4.82	5.31	6.42	7.07	8.22	8.77	9.4	9.35	8.72	7.6	6.69	6.02	3.63
Romania	Female	0.39	0.7	1.27	2.6	4.11	4.73	4.93	5.04	5.34	5.88	6.94	7.8	8.2	8.63	8	6.66	5.48	4.04	2.86	1.98
Russian Federation	Male	0.49	0.61	1.02	2.31	6.31	11.81	19.25	31.7	38.17	47.43	62.35	74.05	87.47	107.11	130.73	143.89	136.27	103.94	149.38	214.75
Russian Federation	Female	0.31	0.47	1.17	2.17	5.09	7.97	10.79	17.39	16.12	28.01	35.63	51.14	65.73	78.28	88.25	92.91	94.98	69.25	95.72	124.66
Rwanda	Male	0.15	0.18	0.21	1.62	0.73	1.1	1.5	1.91	2.1	2.58	3.04	3.66	4.05	4.57	4	4	4.9	3.17	5.03	8.15

Rwanda	Female	0.35	0.28	0.39	2.54	0.92	1.24	1.88	2.83	4.25	39.34	5.33	4.76	3.37	4.01	6.04	105.06	4.16	5.1	2.32	598.29
Saint Lucia	Male	0.18	0.24	0.38	0.72	1.48	2.64	4.29	5.93	7.04	10.36	11.99	14.83	14.81	13.25	12.19	10	6.58	5.71	6.76	6.03
Saint Lucia	Female	0.3	0.38	1.05	0.75	2.32	4.07	10.4	15.12	15.81	25.33	23.12	29.77	29.03	33.45	25.38	22.19	20.83	33.14	26.75	47.24
Saint Vincent and the Grenadines	Male	0.62	0.41	0.56	0.77	1.98	3.22	5.9	12.36	12.99	30.44	31.76	40.87	41.35	39.32	40.49	48.21	28.08	32.12	45.35	33.98
Saint Vincent and the Grenadines	Female	0.13	0.17	0.35	0.55	1.03	1.73	2.93	4.01	4.61	5.97	5.77	6.02	5.43	4.46	3.42	2.49	2.3	2.12	1.66	1.44
Samoa	Male	0.11	0.26	0.39	0.6	1.23	2.69	4.33	6.01	11.37	10.21	13.38	16.95	16.96	10.36	8.7	15.29	32.33	9.25	17.72	2.75
Samoa	Female	0.12	0.1	0.75	0.45	0.65	1.18	4.64	14.1	15.07	7.67	10.55	21.49	8.27	29.33	22.71	27.83	4.62	6.42	8.56	9.69
Sao Tome and Principe	Male	0.13	0.16	0.28	0.68	1.12	1.68	2.34	2.92	3.43	4.28	5.23	6.52	9.86	13.36	9.94	9.78	7.59	8.32	11.25	6.68
Sao Tome and Principe	Female	0.3	0.23	0.34	0.91	0.94	1.27	2.03	3.11	4.83	20.37	6.24	7.69	4.04	7.39	10.54	32.3	12.6	18.78	11.68	89.62
Saudi Arabia	Male	0.16	0.31	0.57	1.03	1.92	3.4	5.24	7.36	8.45	9.44	10.06	9.79	8.43	6.83	5.65	5.46	5.87	4.14	5.18	2.98
Saudi Arabia	Female	0.16	0.3	0.54	0.79	1.13	1.64	2.61	3.78	4.97	5.96	7.11	7.52	6.71	5.6	4.85	5.85	3.03	2.17	2.03	1.55
Senegal	Male	0.11	0.17	0.3	0.67	1.15	1.69	2.35	2.93	3.47	4.47	5.39	6.74	10.72	14.78	10.7	11.27	9.86	10.35	14.42	7.95
Senegal	Female	0.25	0.19	0.34	0.78	0.89	1.23	1.91	2.75	3.99	13.57	5.34	6.52	3.62	6.24	7.88	23.21	8.64	9.77	5.83	52.22
Serbia	Male	0.29	0.47	0.7	1.22	2.06	2.95	3.96	4.96	5.26	6.58	7.48	9.51	10.18	13.08	15.22	14.47	17.87	13.73	9.65	4.24
Serbia	Female	0.36	0.65	1.2	2.45	3.86	4.37	4.8	4.76	5.58	6.93	8.39	9.32	10.72	12.68	11	11.03	10.17	9.33	6.66	3.08
Seychelles	Male	0.17	0.45	0.56	0.79	1.78	4.29	6.13	11.02	16.76	23.17	31.19	32.25	48.76	66.39	49.13	74.96	86.43	57.68	67.77	88.25
Seychelles	Female	0.1	0.29	0.33	0.5	0.99	1.56	2.83	3.38	4.58	8.67	6.63	10.19	15.27	21.04	18.71	22.22	27.23	26.97	38.08	36.27
Sierra Leone	Male	0.15	0.19	0.33	0.65	1.13	1.69	2.36	2.94	3.46	4.3	5.24	6.57	10.29	13.89	10.02	10.13	8.89	8.84	11.89	6.24
Sierra Leone	Female	0.68	0.21	0.37	0.84	0.92	1.23	1.96	2.86	4.17	14.72	5.48	6.51	3.59	6.04	7.63	21.47	7.65	8.44	4.93	47.25
Singapore	Male	0.1	0.19	0.38	0.77	1.87	3.89	6.63	9.99	12.74	14.99	16.87	16.1	14.82	12.23	13.54	10.7	8.03	9.7	11.35	10.25
Singapore	Female	0.08	0.14	0.31	0.51	0.84	1.1	1.48	2.11	3.4	5.07	6.4	7.28	6.17	6.11	5.77	5.99	6.5	4.92	5.17	5.65
Slovakia	Male	0.38	0.63	0.88	1.48	2.54	3.62	4.61	5.41	6.23	7.95	8.57	10.67	12.58	13.23	14.91	16.1	14.88	12.44	14.15	8.83
Slovakia	Female	0.4	0.73	1.77	2.68	4.17	4.95	5.06	5.72	6.06	7.21	9.22	11.31	12.73	14.2	14.5	16.12	15.7	15.08	17.29	14.61
Slovenia	Male	0.32	0.5	0.78	1.32	2.28	3.21	4.09	4.81	5.24	6.38	7.32	8.63	9.19	9.8	11.03	11.38	10.53	11.22	19.55	17.34
Slovenia	Female	0.31	0.57	1.05	2.13	3.42	3.81	3.98	4.12	4.37	5.05	6.34	7.75	7.35	8.75	10.86	12.78	10.58	6.49	6.87	7.27

Solomon Islands	Male	0.15	0.55	0.69	0.76	1.3	3.32	4.47	6.29	20.65	13.26	19.51	26.22	25.08	12.22	9.9	19.58	39.91	9.86	16.54	4.81
Solomon Islands	Female	0.33	0.11	1.69	0.67	0.78	1.48	10.5	29.96	27.75	10.59	14.7	30.74	9.57	36.69	27.02	31.63	4.69	5.79	6.66	6.17
Somalia	Male	0.29	0.42	0.35	3.53	0.86	1.28	1.65	2.15	2.27	2.97	3.45	4.69	5.01	5.98	5.01	5.37	7.05	3.98	6.06	9.87
Somalia	Female	0.91	0.68	0.66	6.6	1.16	1.28	1.93	3.08	5.41	87.47	6.99	7.65	3.98	5.09	8.61	199.36	6.7	9.67	3.83	764.31
South Africa	Male	0.21	0.48	0.54	1.26	2.45	3.9	5.09	5.57	6.24	6.62	8.16	9.67	9.53	12.12	10.89	10.32	8.29	7.59	24.62	7.05
South Africa	Female	0.4	0.49	0.37	0.62	1.15	2.31	3.05	3.94	3.81	4.49	5.14	5.45	4.38	4.96	4.24	3.45	4.82	5.24	9.66	4.72
South Sudan	Male	0.23	0.22	0.22	1.29	0.72	1.1	1.51	1.92	2.1	2.55	3.03	3.65	4.04	4.53	4	4.05	4.98	2.96	4.8	7.49
South Sudan	Female	0.61	0.31	0.41	2.64	0.92	1.24	1.89	2.83	4.28	35.6	5.34	4.7	3.35	3.97	6.12	111.9	4.16	4.83	2.14	553.25
Spain	Male	0.07	0.1	0.18	0.52	1.72	3.92	6.61	9.22	11.03	13.04	15.39	15.97	16.04	15.9	15.81	14.45	15.08	20.44	23.33	38.74
Spain	Female	0.08	0.09	0.15	0.54	1.41	2.18	3.17	3.89	4.86	5.46	6.8	7.35	7.64	7.87	8.23	10.66	12.79	17.53	23.03	25.02
Sri Lanka	Male	0.12	0.17	0.27	0.68	1.27	2.83	5.2	7.47	10.19	13.85	16.7	17.79	19.15	19.11	16.02	14.54	11.38	11.8	14.67	21.97
Sri Lanka	Female	0.07	0.11	0.17	0.3	0.6	1.13	1.85	2.38	2.88	3.98	4.82	5.27	5.73	5.6	5.82	3.96	4.22	4.53	6.43	2.81
Sudan	Male	0.2	0.52	0.74	1.07	1.93	3.44	5.52	7.36	8.81	9.8	10.31	10.22	8.83	7.57	5.95	6.02	5.04	4.03	4.8	2.59
Sudan	Female	0.21	0.54	0.78	0.87	1.22	1.72	2.79	4.09	5.74	6.55	7.77	8.5	7.86	7.17	6.57	6.31	5.48	4.36	3.54	2.15
Suriname	Male	1.13	0.7	0.88	0.82	2.34	3.72	7.11	17	17.84	35.44	46.94	71.81	71.33	76.38	74.26	86.24	52.59	51.32	46.2	32.72
Suriname	Female	0.14	0.22	0.73	0.65	1.47	2.44	5.73	8.63	7.47	10.56	9.38	13.67	12.19	11.52	9.03	7.81	6.12	6.14	3.7	5.65
Sweden	Male	0.25	0.33	0.36	0.75	1.66	3.13	5.25	7.16	8.94	11.99	14.67	15.56	17.2	21.61	23.62	29.58	46.35	60.78	93.43	101.91
Sweden	Female	0.08	0.15	0.44	0.71	1.47	1.62	2.62	3.37	4.07	5.53	7.29	7.49	8.01	9.33	11.1	13.48	16.14	23.34	33.4	43.8
Switzerland	Male	0.07	0.09	0.18	0.57	1.9	4.21	7.05	9.7	11.39	13.76	16.07	16.28	15.64	15.55	15	13.06	18.23	22.43	12.07	4.11
Switzerland	Female	0.05	0.07	0.15	0.45	1.21	2.35	3.22	4.21	4.73	5.1	5.91	6.15	6.77	7.46	7.08	9.32	10.73	10.15	11.34	2.12
Syrian Arab Republic	Male	0.18	0.31	0.57	1.04	1.92	3.39	5.23	7.22	8.79	10.9	10.67	14.53	9.38	7.53	5.7	5.11	3.81	2.88	3.35	1.83
Syrian Arab Republic	Female	0.59	0.34	0.54	0.79	1.13	1.64	2.61	3.73	4.94	5.84	7.11	7.83	6.86	5.7	4.68	3.46	2.64	2.71	3.09	1.68
Tajikistan	Male	0.34	1.07	1.13	1.89	7.34	18.69	24.81	18.5	11.19	14.99	25.19	32.82	48.92	32.73	56.52	99.55	84.55	46.06	41.29	33.28
Tajikistan	Female	0.2	0.37	0.74	1.58	3.75	7.93	4.39	4.7	5.68	6.59	7.82	8.73	26.8	28.44	14.36	39.44	53.92	8.22	5.39	4.47
Thailand	Male	0.12	0.19	0.29	0.55	1.41	4.44	6.21	11.37	18.57	21.87	32.06	39.01	54.79	73.69	79.09	109.68	108.5	90.48	118.58	210.42
Thailand	Female	0.08	0.13	0.18	0.31	0.62	1.17	2.44	3.31	4.4	6.81	7.6	11.79	14.36	33.87	36.23	58.46	75.94	88.25	145.54	182.58
Timor-Leste	Male	0.37	0.85	0.88	1.19	1.92	4.8	6.26	11.2	16.17	22.82	33.17	33.22	54.56	69.13	58.6	83.76	87.56	66.61	72.76	86.22
Timor-Leste	Female	0.27	0.76	0.68	0.81	1.62	1.98	4.11	4.76	7.13	13.53	9.77	20.17	27.32	36.97	31.42	29.7	33.86	30.97	37.29	28.49
Togo	Male	0.11	0.17	0.3	0.66	1.15	1.69	2.33	2.93	3.5	4.43	5.36	6.89	11.08	15.46	10.76	11.52	9.48	9.75	13.15	7.13

Togo	Female	0.29	0.19	0.33	0.81	0.9	1.23	1.95	2.81	4.05	13.99	5.29	6.41	3.56	6.01	7.66	21.1	8.06	9.17	5.25	48.46
Tonga	Male	0.2	0.58	0.65	0.66	1.27	2.72	4.33	5.91	13.02	11.35	16.39	24.15	26.44	14.34	13.45	36.88	90.67	24.58	56.98	10.62
Tonga	Female	0.25	0.12	1.19	0.42	0.62	1.12	2.62	4.97	5.48	4.83	6.12	8.76	5.89	11.05	8.27	8.5	1.91	2	2.48	2.64
Trinidad and Tobago	Male	5.66	0.4	0.45	0.73	1.43	2.56	4.09	6.74	14.44	42.72	64.68	93.76	119.27	121.89	116.34	114.54	65.96	73.38	139.17	16.17
Trinidad and Tobago	Female	0.17	0.19	0.39	0.56	1.01	1.67	7.68	12.5	18.36	31.59	29.72	33.07	18.06	22.95	20.15	30.86	29.62	25.97	5.11	42.77
Tunisia	Male	0.16	0.32	0.59	1.04	1.93	3.41	5.31	7.21	8.82	9.81	10.32	10.12	8.55	7.02	5.59	4.92	4.05	3.13	3.71	2.1
Tunisia	Female	0.16	0.34	0.57	0.8	1.14	1.65	2.66	3.83	5.18	6.11	7.29	7.94	7.24	6.16	5.23	4.31	3.59	2.77	2.39	1.69
Turkey	Male	0.19	0.38	0.62	1.05	1.94	3.67	5.92	8.17	9.37	11.12	12.36	12.67	12.35	10.74	10.75	13.1	13.15	11.86	14.94	10.1
Turkey	Female	0.22	0.61	0.62	0.8	1.14	1.75	2.85	3.92	5.47	7.2	8.49	9.28	8.39	8.76	9.82	11.62	10.44	11.34	6.87	5.91
Turkmenistan	Male	0.33	1.34	1.23	1.86	4.1	6.93	8.91	8.78	12.09	19.41	20.62	21.41	28.2	36.27	31.89	38.96	24.94	29.92	34.81	24.6
Turkmenistan	Female	0.26	0.51	0.85	1.85	2.59	4.21	7.46	5.97	10.05	12.7	16.18	16.24	26.11	25.43	28.32	27.24	24.83	7.34	6.96	7.92
Uganda	Male	0.13	0.17	0.2	1.41	0.71	1.09	1.5	1.92	2.12	2.59	3.04	3.71	4.03	4.55	3.95	3.95	4.56	2.85	4.33	7.27
Uganda	Female	0.26	0.25	0.38	2.24	0.9	1.24	1.88	2.83	4.2	31.2	5.21	4.6	3.33	3.9	5.86	102.36	3.92	4.85	2.18	545.79
Ukraine	Male	0.3	0.43	0.79	1.92	6.15	12.15	19.82	37.3	47.39	53.23	72.6	82.2	91.68	110.93	105.69	91.76	51.74	47.02	43.4	50.96
Ukraine	Female	0.22	0.35	0.75	1.83	3.89	6.74	9.65	14.55	16.15	31.95	40.97	58.59	67.42	63.34	55.93	42.97	24.55	12.59	6.96	4.39
United Arab Emirates	Male	0.16	0.32	0.64	1.07	1.96	3.46	5.52	7.54	8.89	10.2	10.39	10.66	9.48	8.32	6.35	7.17	6.78	5.82	7.52	6.55
United Arab Emirates	Female	0.16	0.33	0.59	0.85	1.21	1.71	2.79	4.02	5.69	6.8	7.9	8.64	7.87	7.89	7.02	6.89	7.5	6.03	4.7	5.31
United Kingdom	Male	0.46	0.4	0.54	0.8	2.44	5.51	8.75	11.66	14.38	17.77	20.34	22.76	24.31	26.25	29.64	32.7	39.75	49.63	65.33	79.21
United Kingdom	Female	0.05	0.19	0.34	1.12	2.88	3.68	5.37	6.94	7.93	8.89	12.06	13.96	15.86	17.55	23.71	29.04	32.9	34.33	45.2	58.33
United Republic of Tanzania	Male	0.15	0.15	0.19	0.99	0.66	1.04	1.46	1.85	2.06	2.49	2.9	3.36	3.64	3.77	3.23	2.61	2.35	1.71	2.93	5.13
United Republic of Tanzania	Female	0.5	0.3	0.41	2.65	0.92	1.24	1.89	2.85	4.26	39.3	5.39	4.85	3.39	4.01	6.2	105.53	3.98	4.67	2.2	614.73
United States of America	Male	0.13	0.24	0.29	0.7	1.6	2.37	3.33	4.39	5.53	6.38	7.9	9.74	12.36	15.14	18.06	19.3	22.99	26.11	28.38	24.25
United States of America	Female	0.13	0.21	0.5	1.19	2.61	3.19	4.03	4.56	5.32	6.23	7.53	9.01	10.49	11.67	13.65	16	20.77	22.27	23.34	21.56

United States Virgin Islands	Male	0.38	0.5	0.59	0.78	2.08	3.24	5.96	12.43	16.62	34.02	46.12	71.52	73.68	72.14	79.86	87.17	52.63	52.62	59.89	49.29
United States Virgin Islands	Female	0.21	0.29	0.88	0.74	2.08	3.26	9.11	15.63	16.13	28.27	27.79	43.39	41.38	48.16	41.56	44.63	42.07	54.67	39.71	61.19
Uruguay	Male	0.09	0.21	0.42	0.87	2.35	5.12	8.48	11.32	13.21	14.46	15.72	14.08	17.53	15.59	14.92	14.52	21.38	10.89	13.78	3.4
Uruguay	Female	0.06	0.19	0.36	0.88	1.95	4.95	7.47	6.72	9.02	12.47	10.02	9.36	8.96	8.92	8.74	11.64	14.8	12.32	9.07	16.85
Uzbekistan	Male	0.29	0.47	0.82	1.74	3.99	6.4	8.06	8.24	9.28	11.23	12.52	14.2	15.53	15.9	14.82	13.06	9.61	8.02	7	4.42
Uzbekistan	Female	0.19	0.33	0.7	1.49	2.47	3.22	3.92	4.65	5.2	6.27	7.21	7.78	7.73	6.6	6.2	4.97	5.3	3.69	2.73	2.47
Vanuatu	Male	0.14	0.56	0.61	0.71	1.26	3.04	4.44	6.03	16.01	11.58	16.61	21.98	22.25	11.84	9.79	20.1	44.34	12.36	24.53	4.02
Vanuatu	Female	0.33	0.11	1.22	0.51	0.66	1.18	4.74	15.23	16.01	7.87	11.13	23.07	8.38	31.68	24.07	27.84	4.42	6.17	7.86	8.72
Venezuela (Bolivarian Republic of)	Male	0.56	0.44	0.65	0.74	2.1	3.81	5.82	7.66	12.48	14.79	19.74	22.62	21.91	22.88	25.78	35.32	41.68	37.46	38.99	51.67
Venezuela (Bolivarian Republic of)	Female	0.58	0.55	1.02	2.59	4.32	9.7	9.22	10.48	13.46	12.14	17.51	16.54	19.52	15.08	13.86	17.52	18.1	13.85	12.32	7.65
Viet Nam	Male	0.2	0.37	0.57	1.09	2.28	5.06	8.28	13.53	18.54	24.49	30.23	30.63	35.67	37.11	28.36	28.74	27.9	19.23	18.92	19.75
Viet Nam	Female	0.14	0.41	0.54	0.83	1.51	2.29	4.01	5.29	7.62	10.65	9.85	13.57	17.18	20.49	18.88	18.49	19.72	23.24	31.01	30.38
Yemen	Male	0.17	0.49	0.72	1.05	1.93	3.44	5.4	7.42	8.78	9.93	10.28	10.34	8.83	7.68	6.17	6.09	4.91	4.01	4.94	2.73
Yemen	Female	0.18	0.53	0.79	0.85	1.21	1.73	2.8	4.11	5.91	6.64	7.78	8.7	7.93	7.37	6.65	6.16	5.46	4.5	3.58	2.32
Zambia	Male	0.13	0.18	0.23	1.88	0.79	1.19	1.56	2	2.14	2.69	3.18	3.99	4.38	5.04	4.34	4.42	5.68	3.48	5.79	9.6
Zambia	Female	0.35	0.29	0.42	3.63	0.99	1.24	1.88	2.92	4.82	61.12	6.08	5.75	3.58	4.4	7.24	136.25	4.98	6.61	3.04	750.12
Zimbabwe	Male	0.39	0.99	1.69	10.15	10.55	6.85	3.95	4.28	4.73	5.31	6.13	7.04	9.38	22.83	14.72	13.85	13.72	15.35	39.87	15.29
Zimbabwe	Female	0.12	0.18	0.33	0.52	0.72	1.07	1.71	2.43	2.83	3.24	3.34	3.21	2.72	2.68	3.3	10.63	3.49	2.21	3.6	3.24

Supplementary figure 1

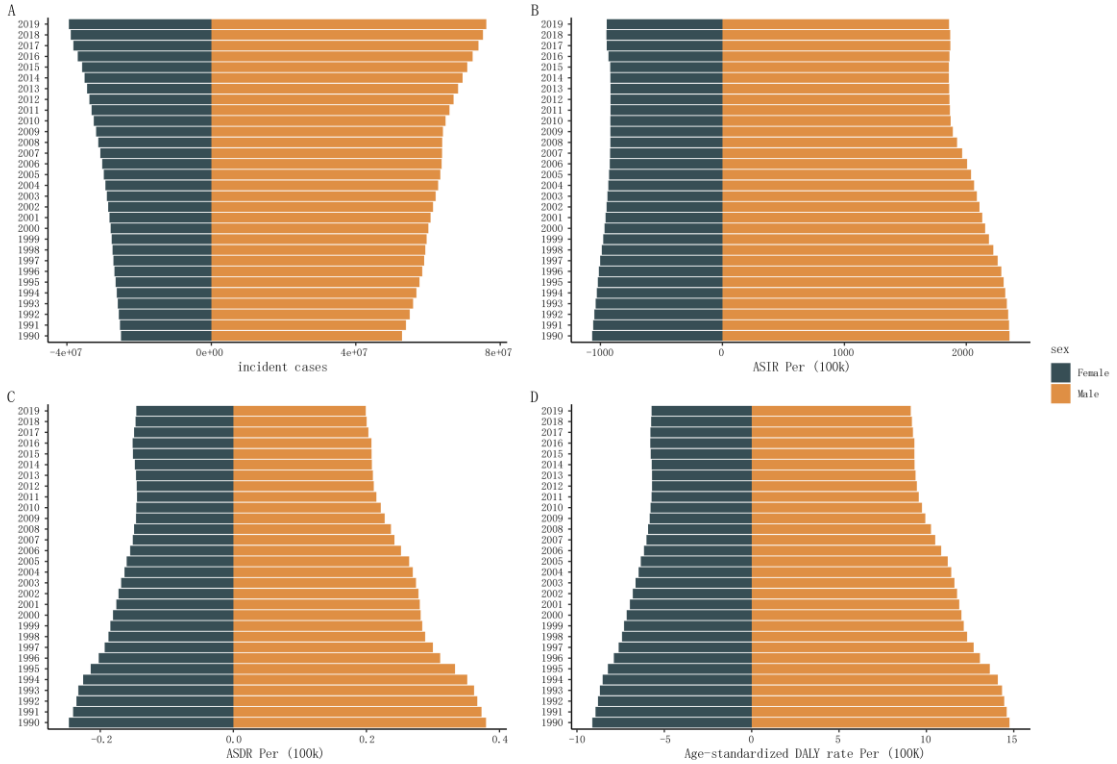

Supplementary figure 1. The incident cases (A), age standardized incidence (B), death (C) and DALYs (D) rates of urolithiasis between 1990 and 2019 both males and females.

Supplementary figure 2

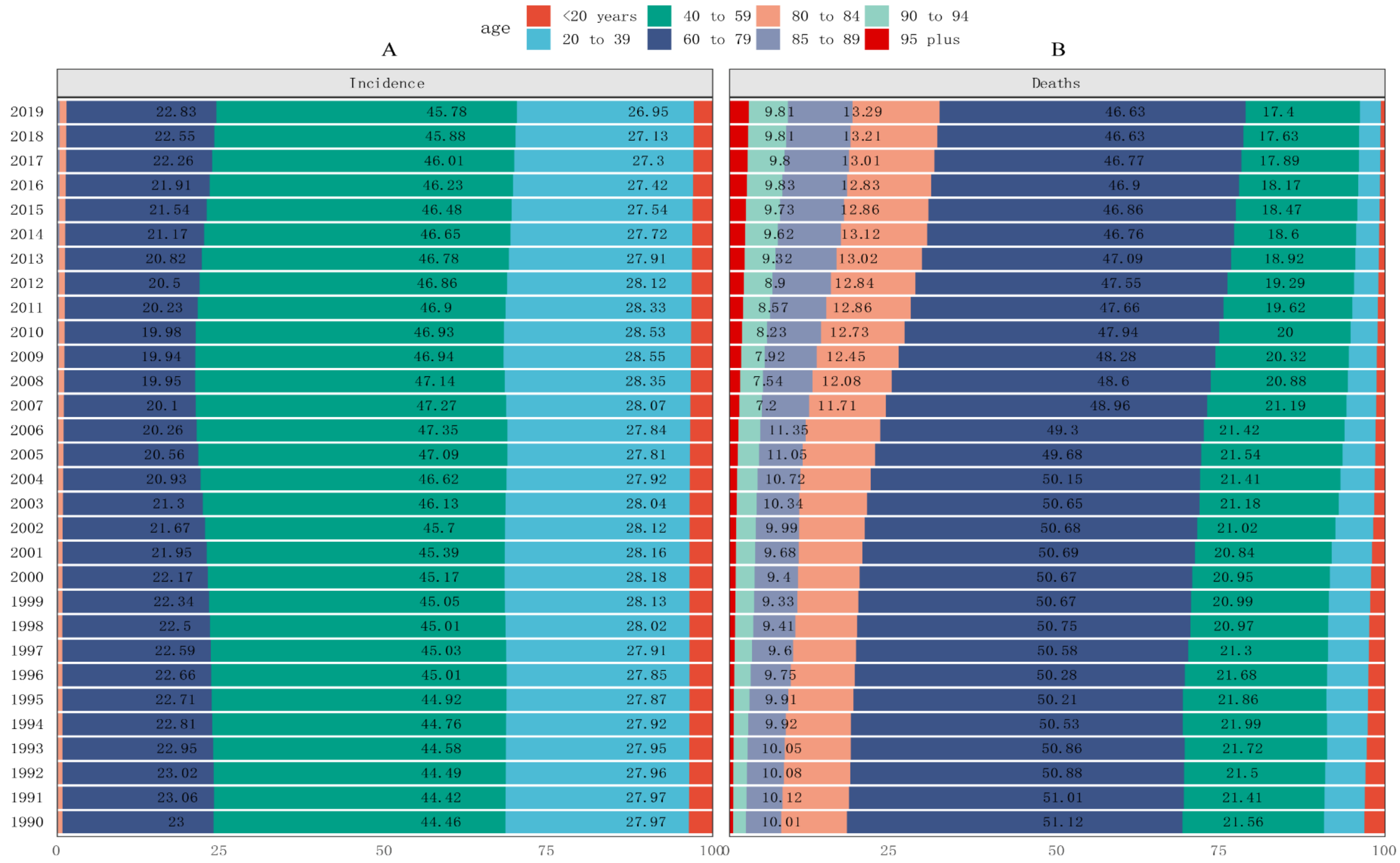

Supplementary figure 2. The proportion of different ages in urolithiasis incidence (A) and death (B) by years.

Supplementary figure 3

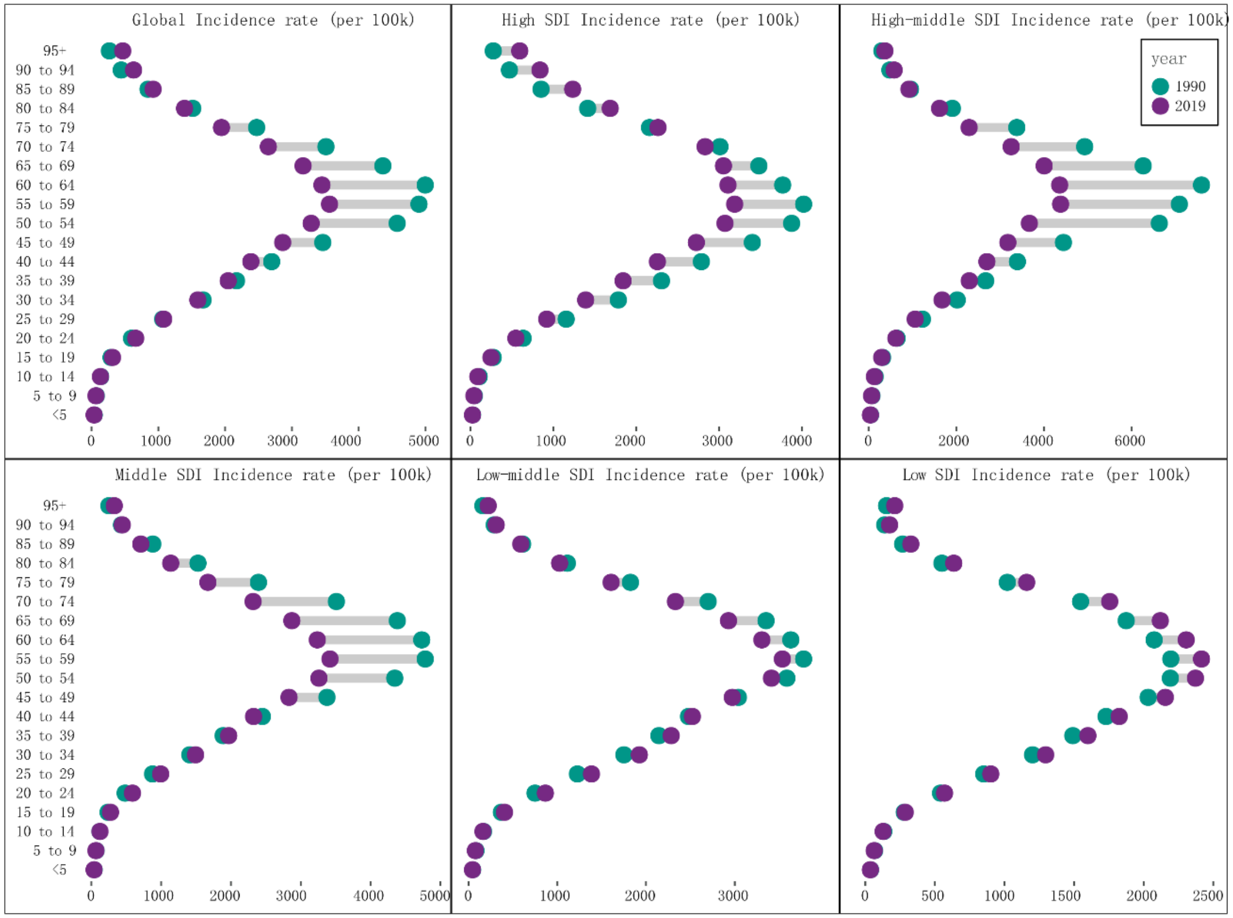

Supplementary figure 3. Distribution of different ages in urolithiasis incidence in global population.

Supplementary figure 4

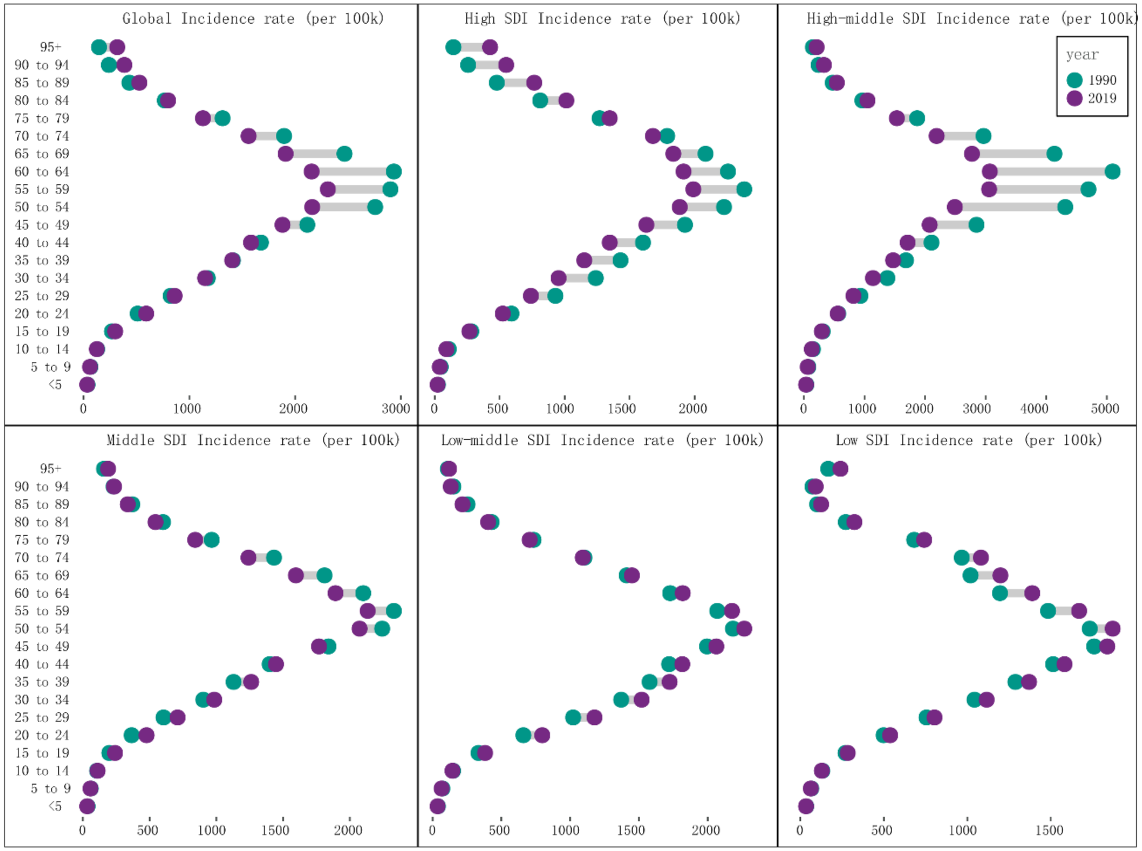

Supplementary figure 4. Distribution of different ages in urolithiasis incidence in female subjects.

Supplementary figure 5

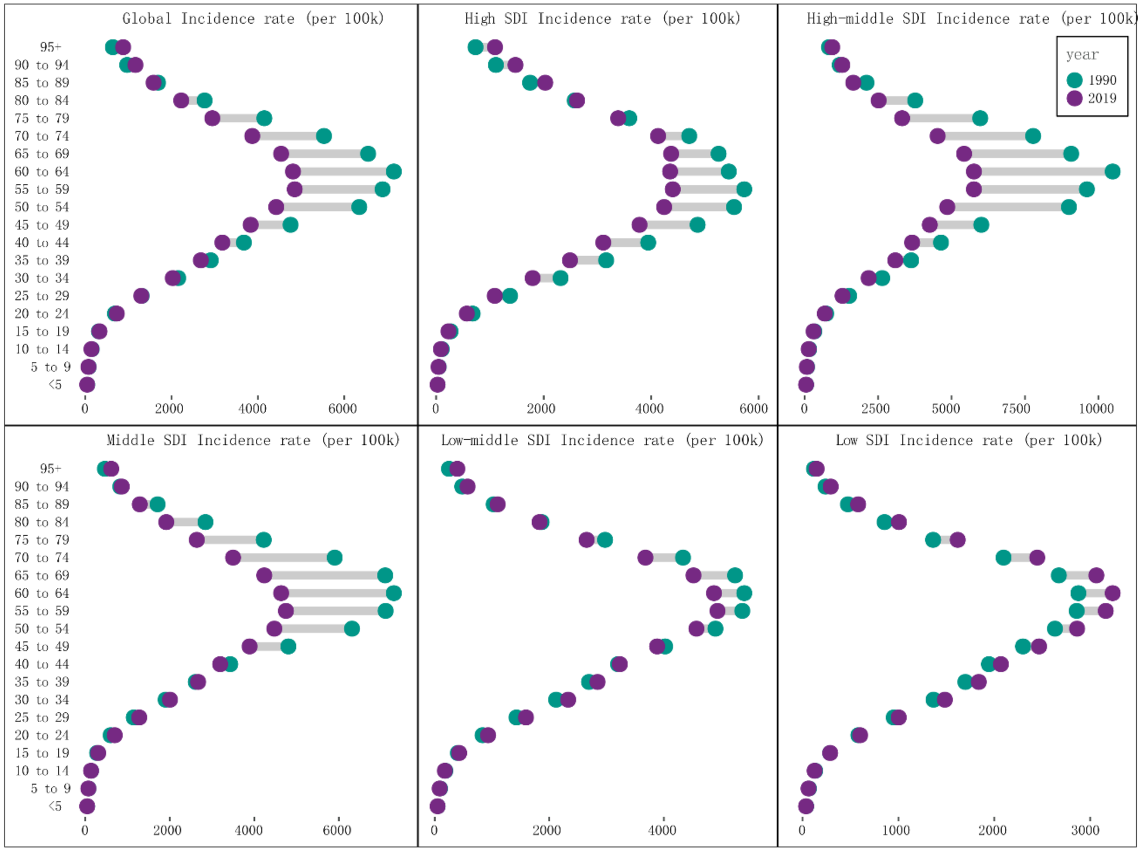

Supplementary figure 5. Distribution of different ages in urolithiasis incidence in male subjects.

Supplementary figure 6

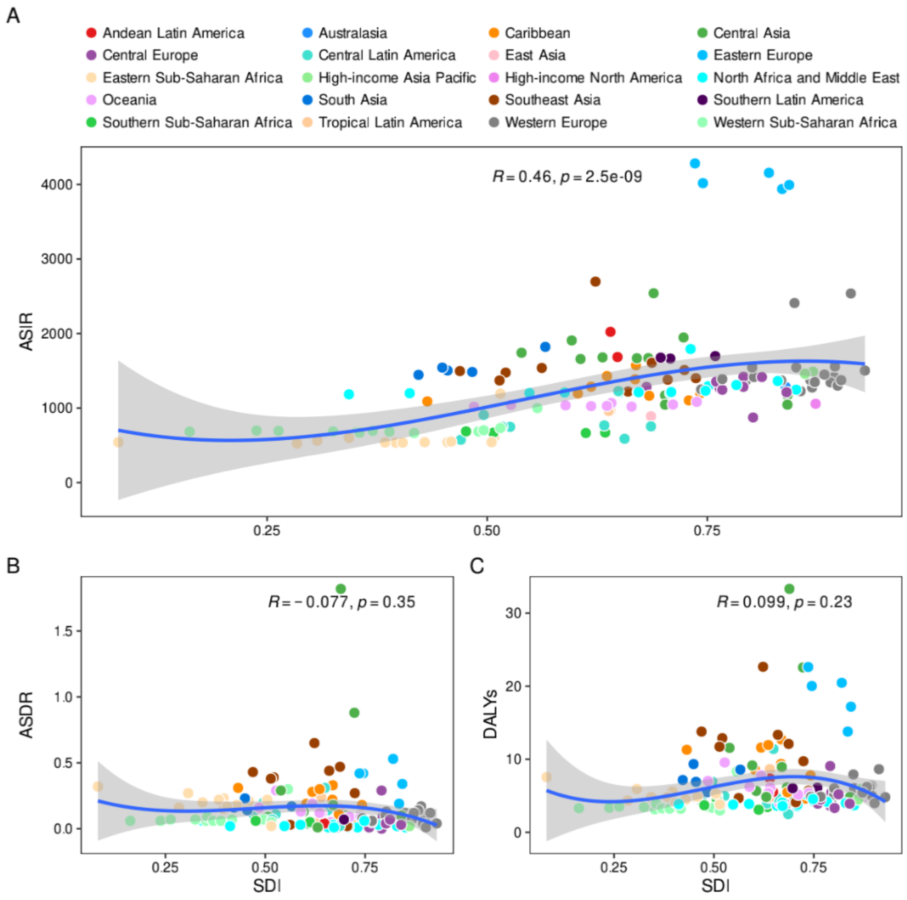

Supplementary figure 6. The age standardized incidence (A), death (B) and DALY (C) rates of urolithiasis per 100,000 population among regions based on SDI in 2019.

Supplementary figure 7

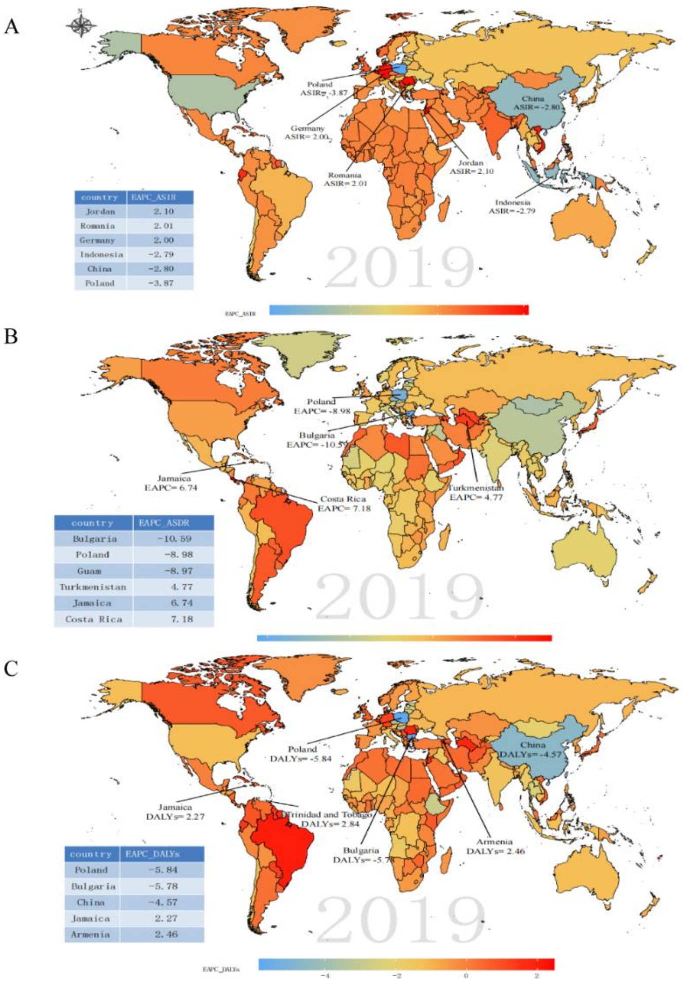

Supplementary figure 7 The global EAPC of Urolithiasis both sex in 192 countries. A. The EAPC of ASIR; B. The EAPC of ASDR; C. The EAPC of DALYs.

Supplementary figure 8

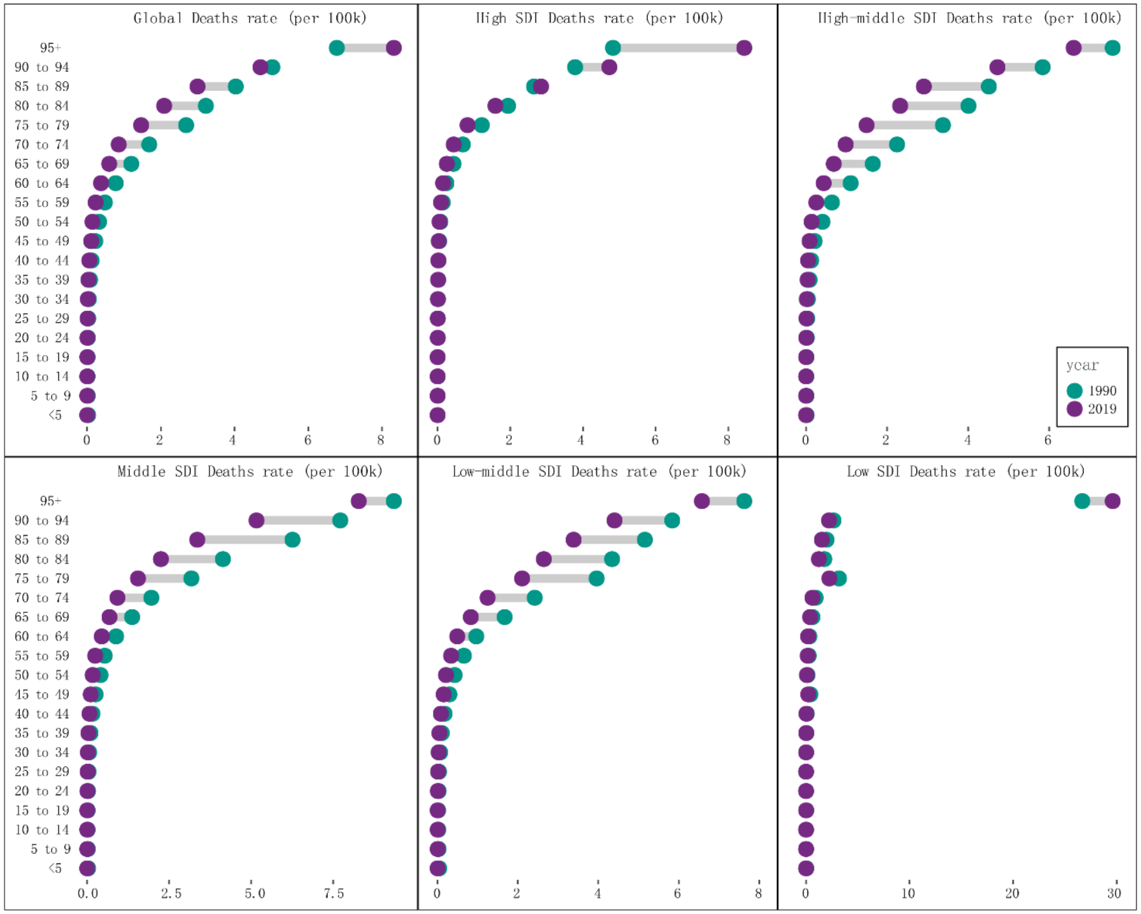

Supplementary figure 8. Distribution of different ages in urolithiasis death rate in global level.

Supplementary figure 9

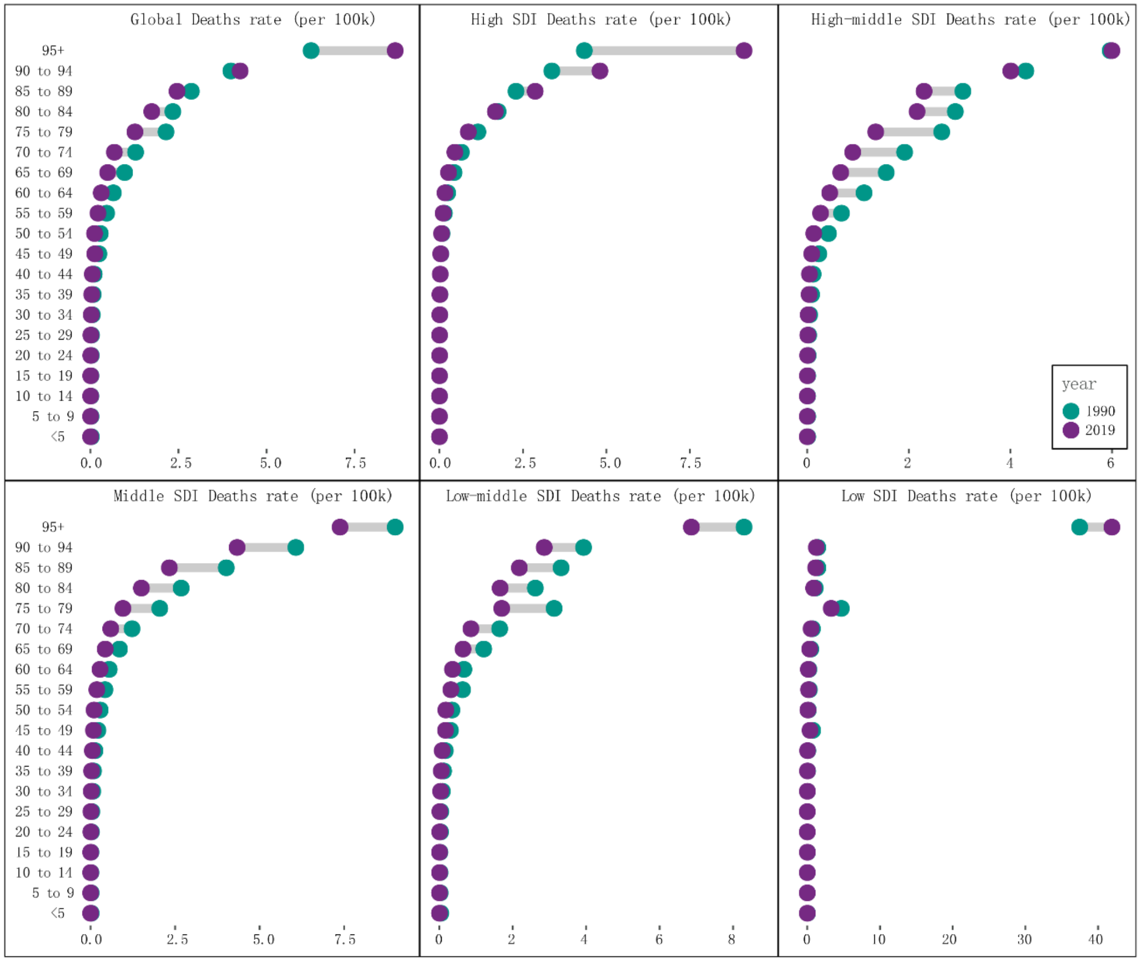

Supplementary figure 9. Distribution of different ages in urolithiasis death rate in females.

Supplementary figure 10

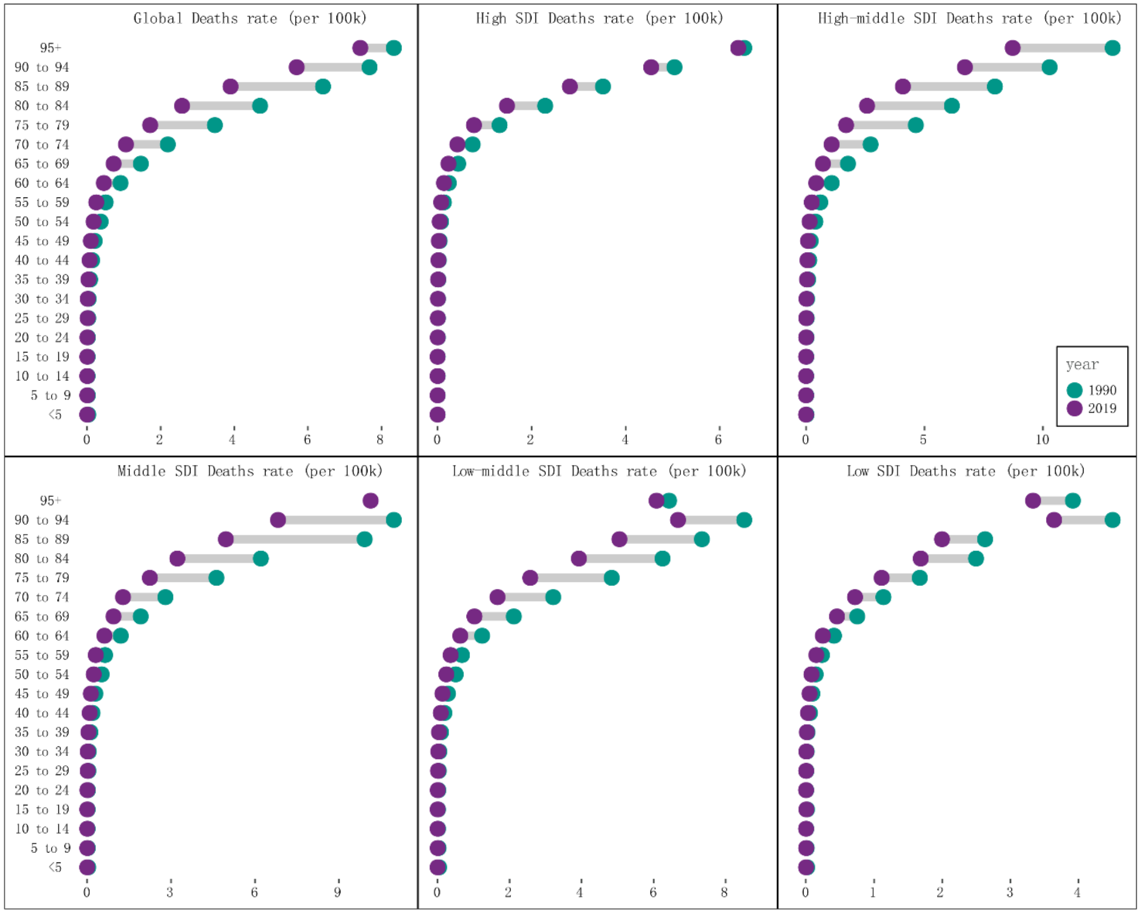

Supplementary Figure 10. Distribution of different ages in urolithiasis death rate in males.

Supplementary Figure 11

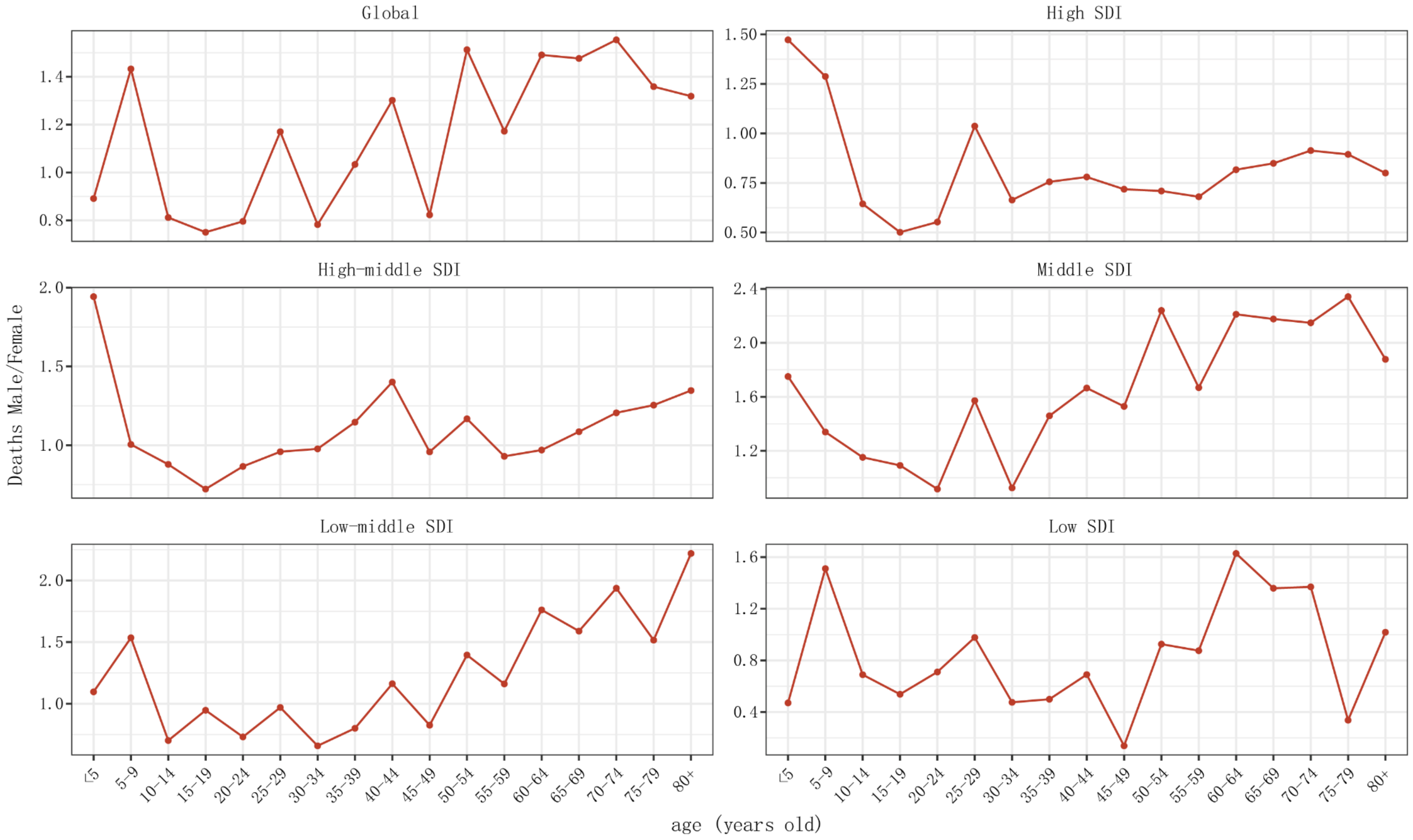

Supplementary figure 11. The ratio of male to female ASDR among different age groups in global. SDI, socio-demographic index.

Supplementary figure 12

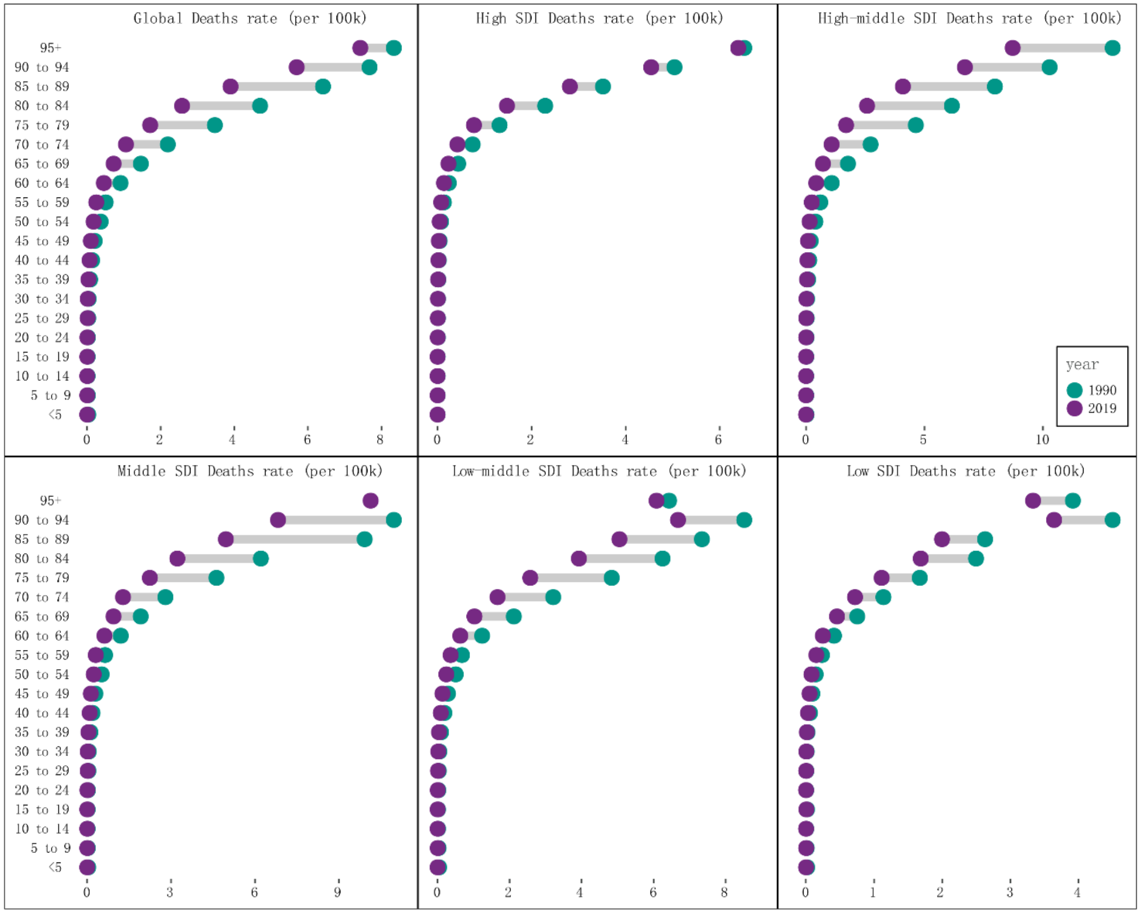

Supplementary figure 12. Distribution of different ages in urolithiasis DALYs in global level.

Supplementary figure 13

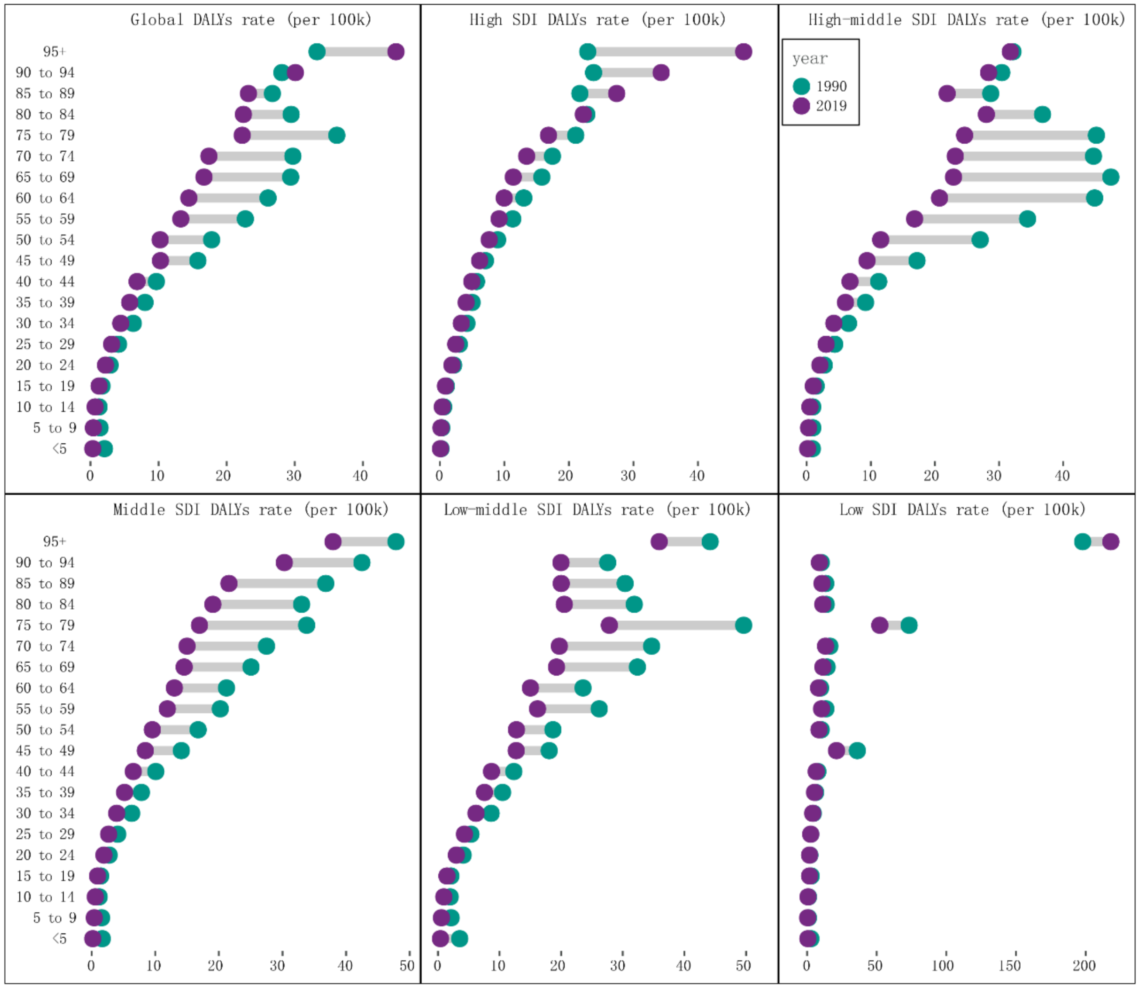

Supplementary figure 13. Distribution of different ages in urolithiasis DALYs in females.

Supplementary figure 14

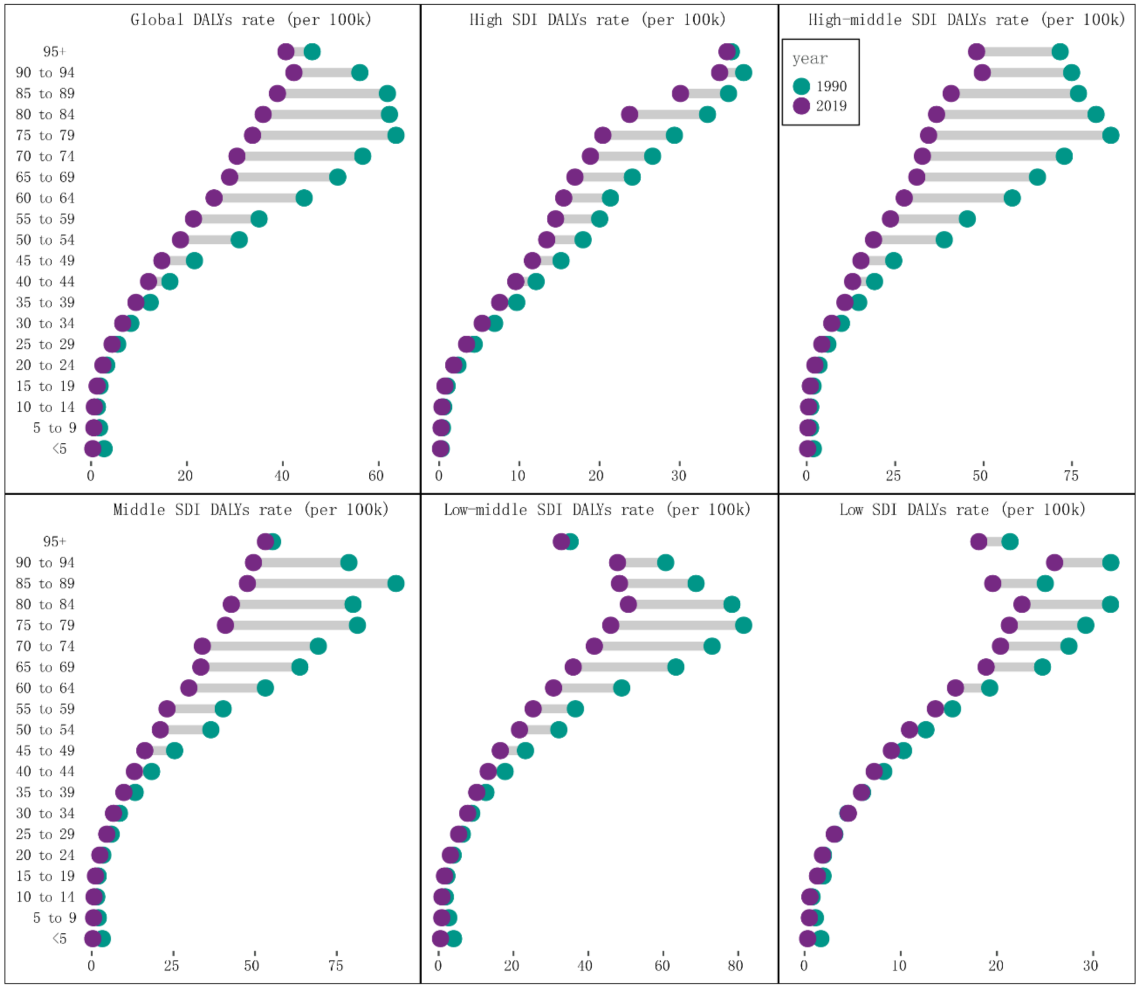

Supplementary Figure 14. Distribution of different ages in urolithiasis DALYs in males.

Supplementary figure 15

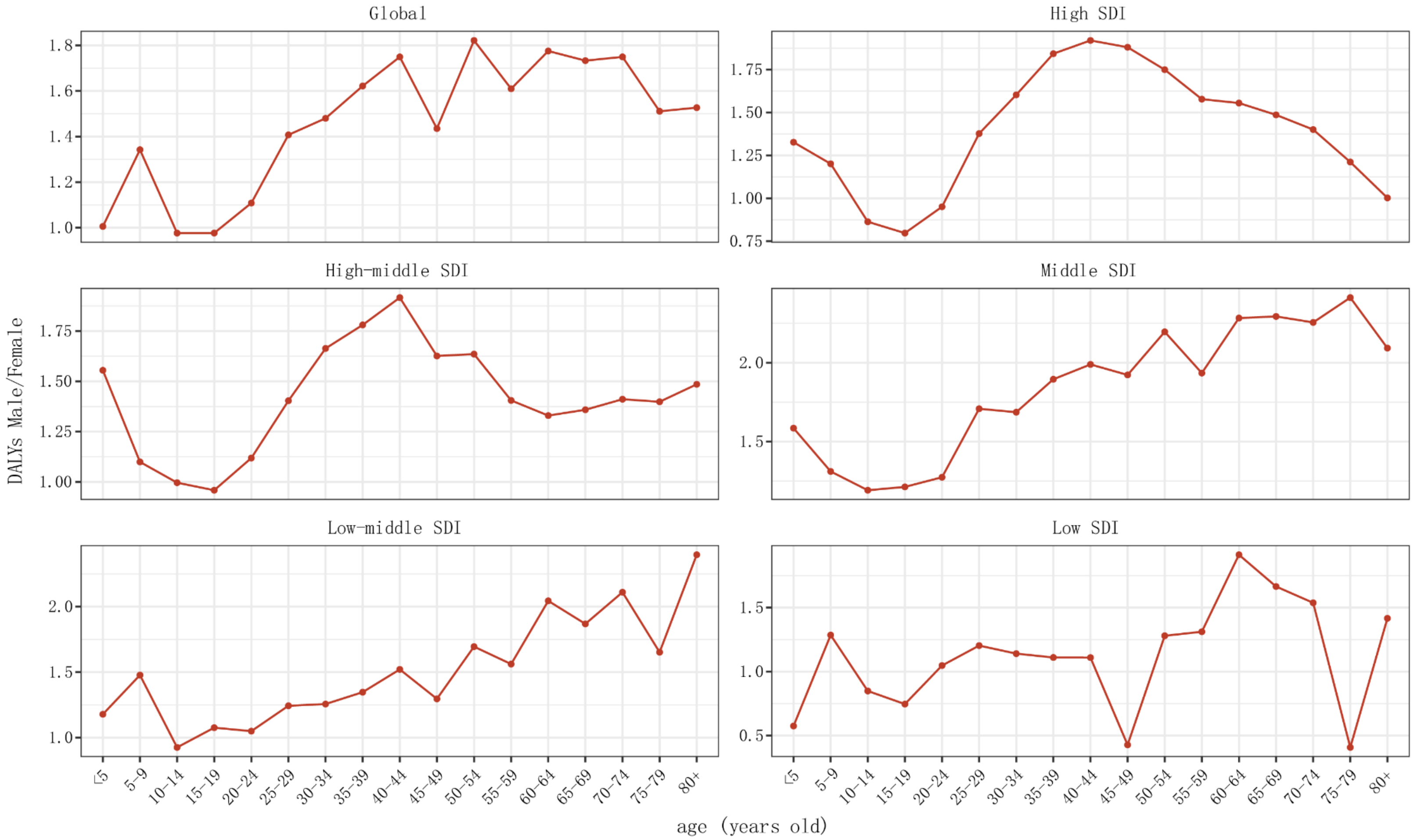

Supplementary figure 15. The ratio of male to female ASDR among different age groups in global. SDI, socio-demographic index.
