# Supplementary material for: Epidemiological Trends of Urolithiasis at the Global, Regional, and National Levels: A Population-Based Study
Source: Int J Clin Pract. 2022 Mar 30;2022:6807203. doi: 10.1155/2022/6807203 (PMC9159214; doi:10.1155/2022/6807203)
Supplement: Supplementary Materials — The supplementary material file 1: the supplementary tables and figures are already cited in our manuscript “Epidemiological trends of urolithiasis at the global, regional, and national levels: a population-based study.” The supplementary material file 2: figures are already cited in the manuscript “Epidemiological trends of urolithiasis at the global, regional, and national levels: a population-based study.” [file 6807203.f1.zip › 6807203.f1/The supplementary material file 2.docx]

ORIGINAL RESEARCH

Xiaoyuan Qian et al

**Epidemiological trends of urolithiasis at the global, regional, and national levels: a population-based study**

Author names: Xiaoyuan Qian^1†^, Junlai Wan^2†^, Jinzhou Xu^1^, Chenqian Liu^1^, Mingliang Zhong^1^, Mingling Zhong,^1^ Jiaqiao Zhang^1^, Ying Zhang^3*^, Shaogang Wang^1*^

^1^Author affiliation

Department of Urology Surgery, at Tongji Hospital, Tongji Medical College, Huazhong University of Science and Technology, Wuhan 430030, China;

^2^Author Affiliation

Department of Orthopedics, Tongji Hospital, Tongji Medical College, Huazhong University of Science and Technology, Wuhan 430030, China

^3^Author affiliation

Department of Nephrology, Tongji Hospital of Tongji Medical College, Huazhong University of Science and Technology, Wuhan 430030, China

Figure 1. The incidence, death, and DALY rates of Urolithiasis in different age groups. A. Incidence in 1990; B. Incidence in 2019; C. Death rate in 1990; D. Death rate in 2019; E. DALY rate in 1990; F. DALY rate in 2019.

Figure 2. The ratio of male to female incidence in different age groups in 2019. A. Global level; B. High SDI regions; C. High-middle SDI regions; D. Middle SDI regions; E. Middle-low SDI regions; F. Low SDI regions; SDI, socio-demographic index.

Figure 3. The change trends of age-standardized incidence (ASIR), age-standardized death (ASDR), and age-standardized incidence DALYs rate among different SDI countries. A – C: ASIR; D – F: ASDR; H – J: Age-standardized DALYs rate.

Figure 4. Distribution of different ages in Urolithiasis incidence/death patients by region. A. Incidence in 1990 and 2019. B. Death rate in 1990 and 2019.

Figure 5. The correlation between EAPCs and Urolithiasis ASR (incidence (A), death (B), and DALY (C)) in 2019 and HDI (incidence (D), death (E), and DALYs (F)) in 2019. The circles represent countries that were available on SDI values. The size of circles described the number of urolithiasis patients. The R indices Pearson’s correlation coefficient and p values were obtained from Pearson’s correlation analysis. ASR, age-standardized incidence/death/DALYs rate; EAPC, estimated annual percentage change; SDI, socio-demographic index.

Figure 6. The global disease burden of urolithiasis for both sexes in 192 countries. A: The ASIR of urolithiasis in 2019; B. The ASDR of urolithiasis in 2019; C. The age-standardized DALY rate of urolithiasis in 2019; ASIR, age-standardized incidence rate; ASDR, age-standardized death rate.

Figure 1


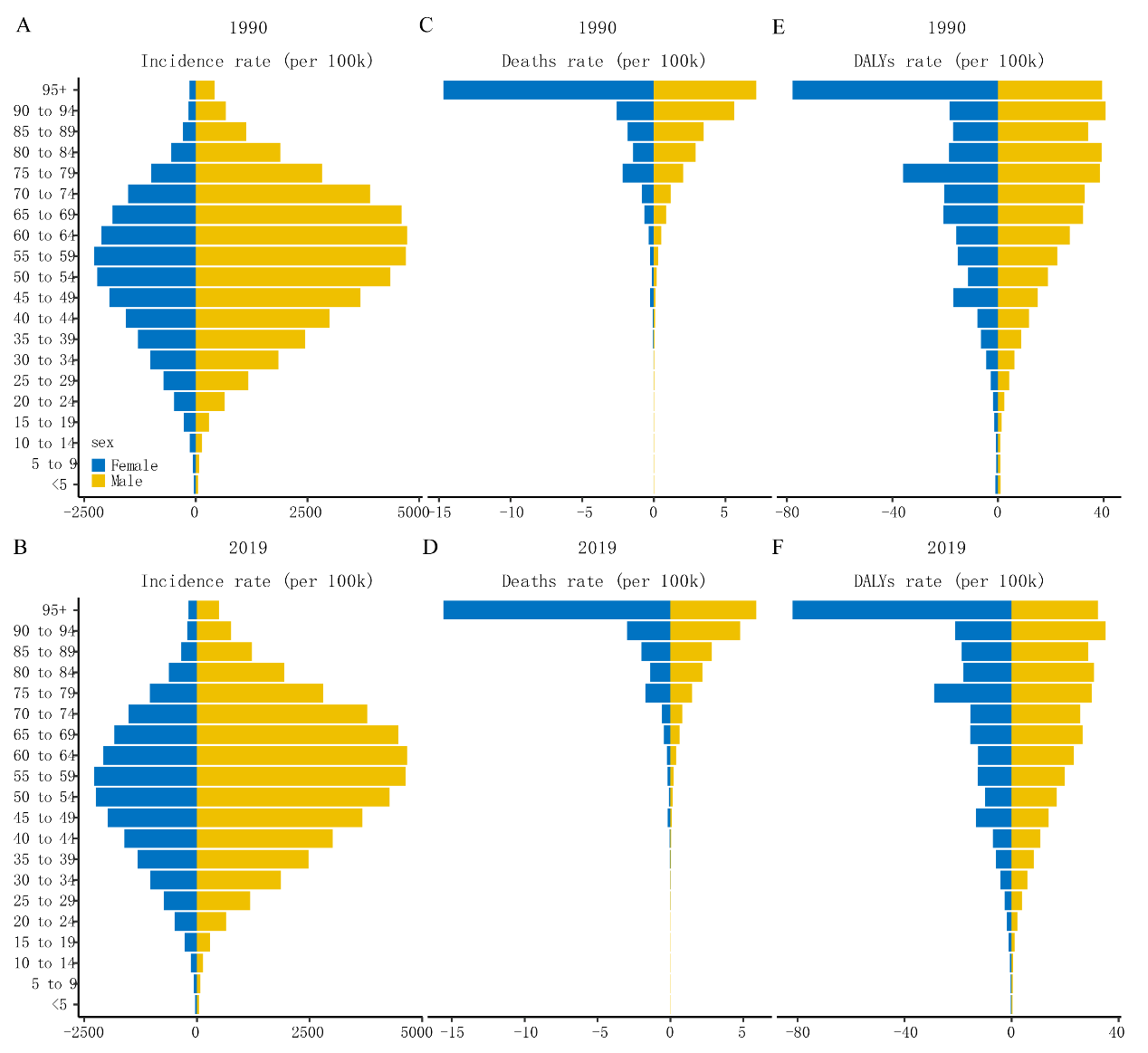


Figure 1. The incidence, death, and DALY rates of Urolithiasis in different age groups. A. Incidence in 1990. B. Incidence in 2019. C. Death rate in 1990. D. Death rate in 2019. E. DALY rate in 1990. F. DALY rate in 2019.

Figure 2


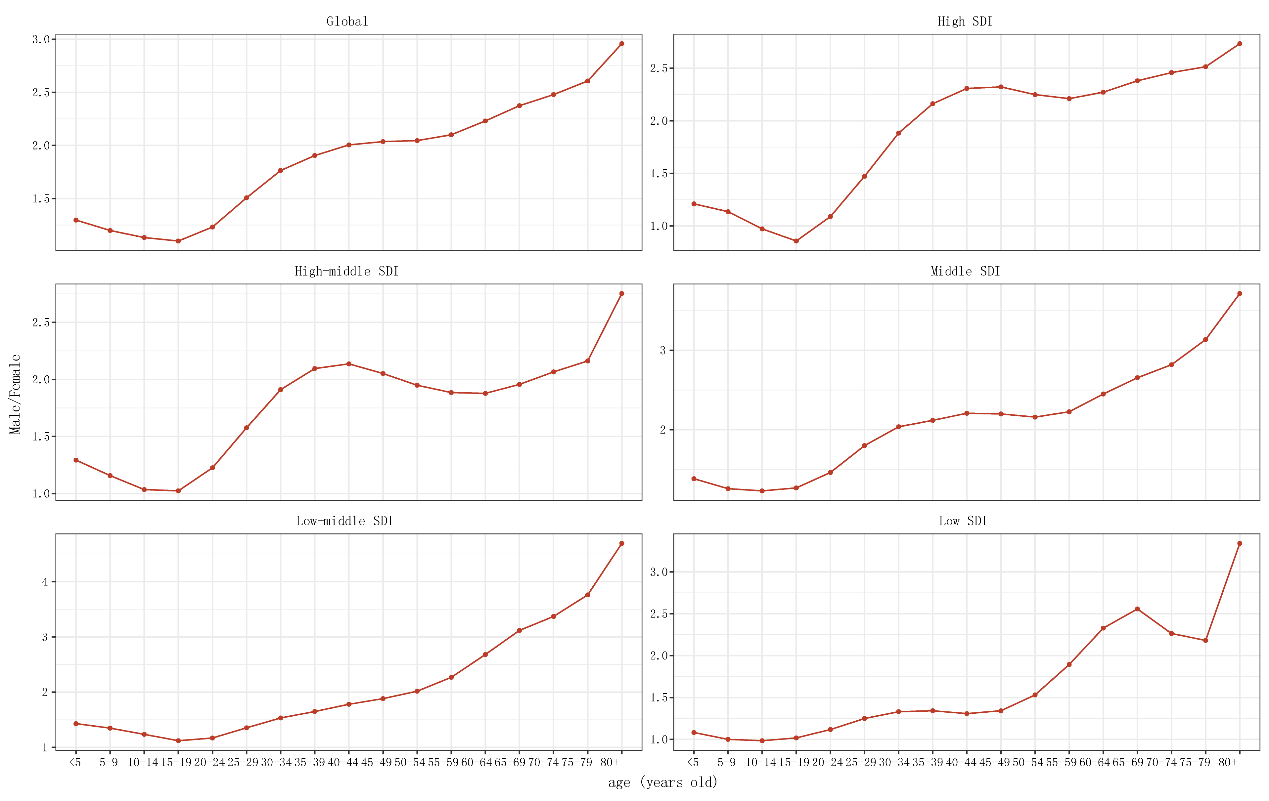


Figure 2. The ratio of male to female incidence in different age groups in 2019. A: Global. B High SDI regions. C. High-middle SDI regions. D. Middle SDI regions. E. Middle-low SDI regions. F. Low SDI regions. SDI, socio-demographic index.

Figure 3


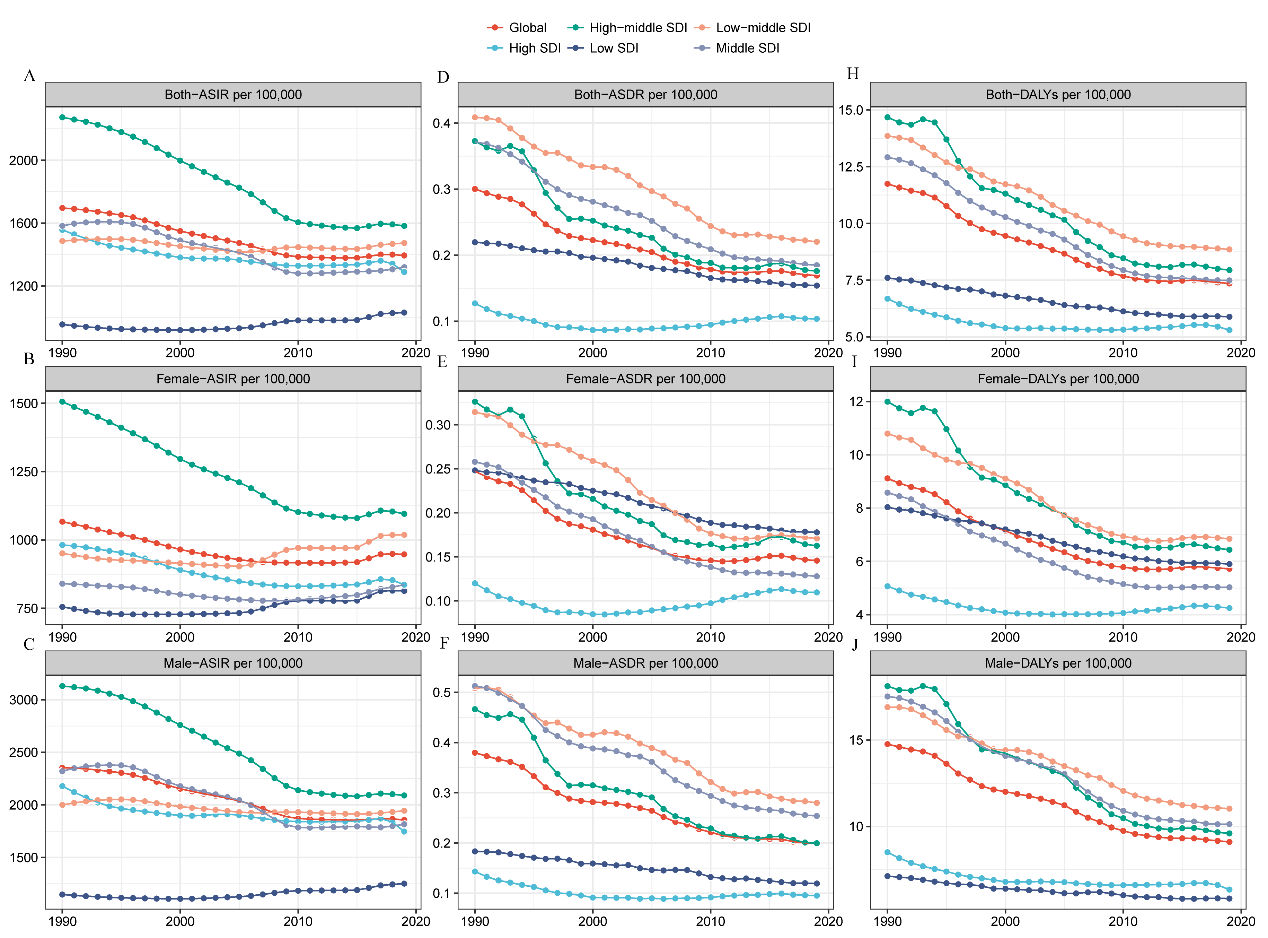


Figure 3. The change trends of age-standardized incidence (ASIR), age-standardized death (ASDR), and age-standardized incidence DALYs rate among different SDI countries. A - C: ASIR; D - F: ASDR; H – J: Age-standardized DALYs rate.

Figure 4.


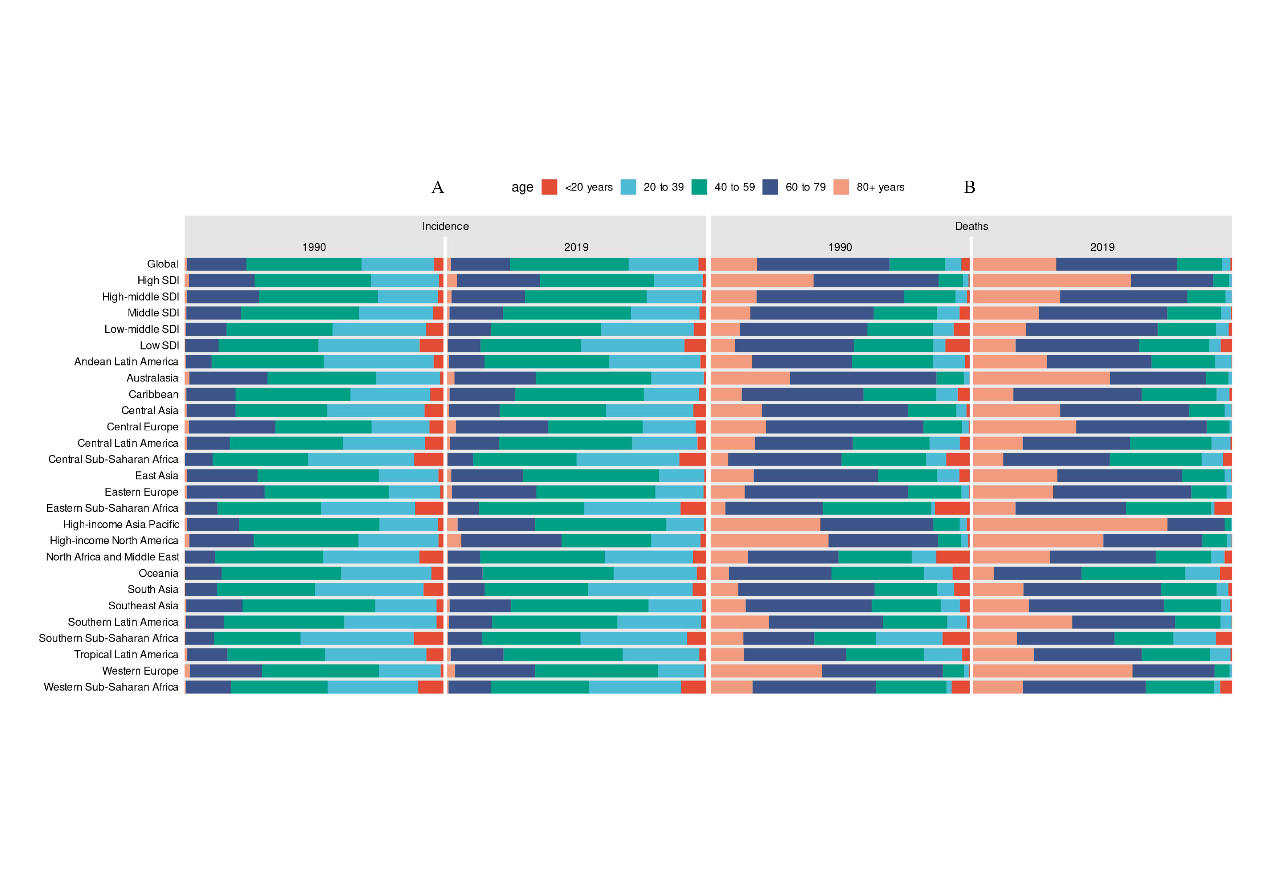


Figure 4. Distribution of different ages in Urolithiasis incidence/death patients by region. A. Incidence in 1990 and 2019. B. Death rate in 1990 and 2019.

Figure 5


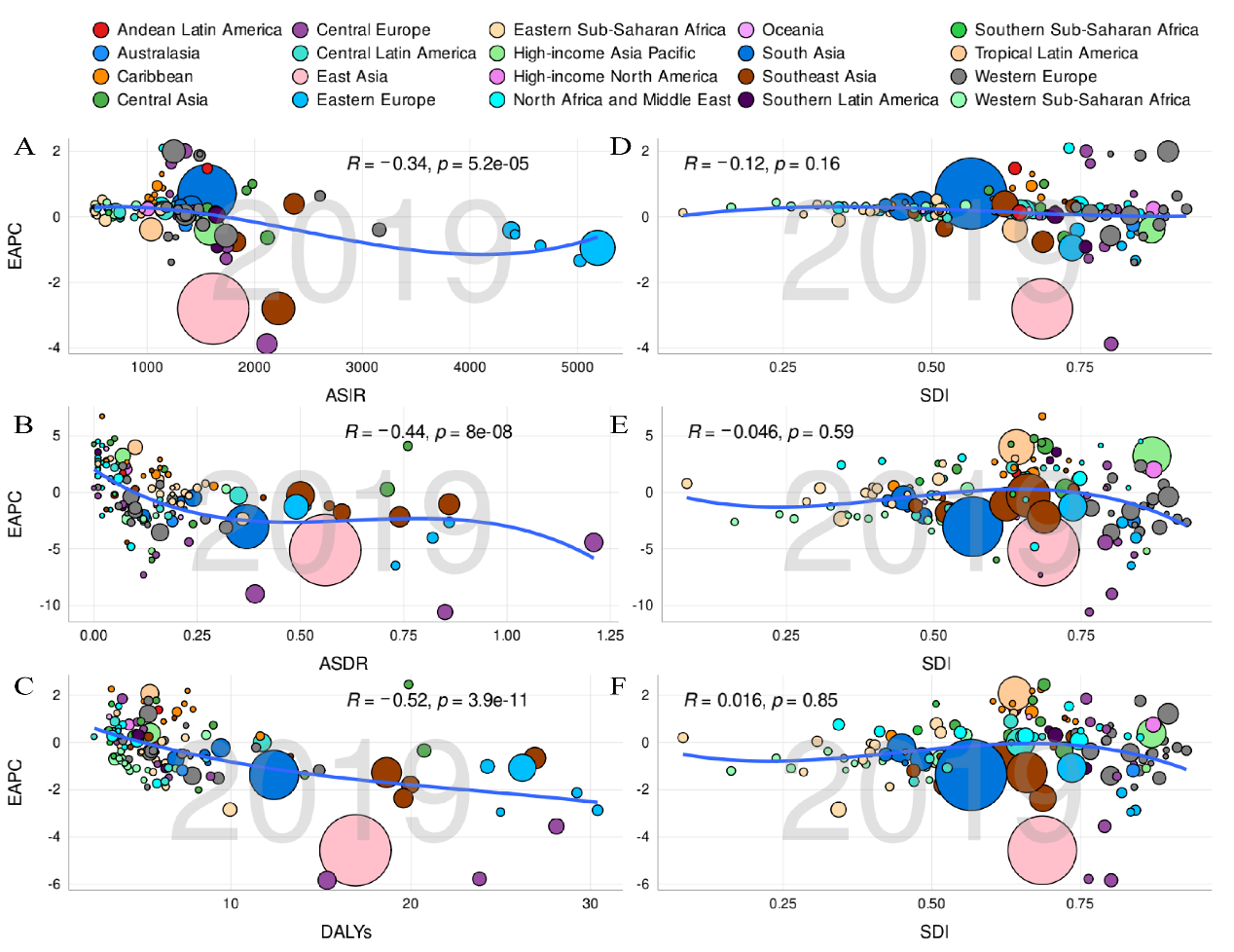


Figure 5. The correlation between EAPCs and urolithiasis ASR (incidence (A), death (B), and DALY (C)) in 2019 and HDI (incidence (D), death (E), and DALYs (F)) in 2019. The circles represent countries that were available on SDI values. The size of circles described the number of urolithiasis patients. The R indices Pearson’s correlation coefficient and p values were obtained from Pearson’s correlation analysis. ASR, age-standardized incidence/death/DALYs rate; EAPC, estimated annual percentage change; SDI, socio-demographic index.

Figure 6


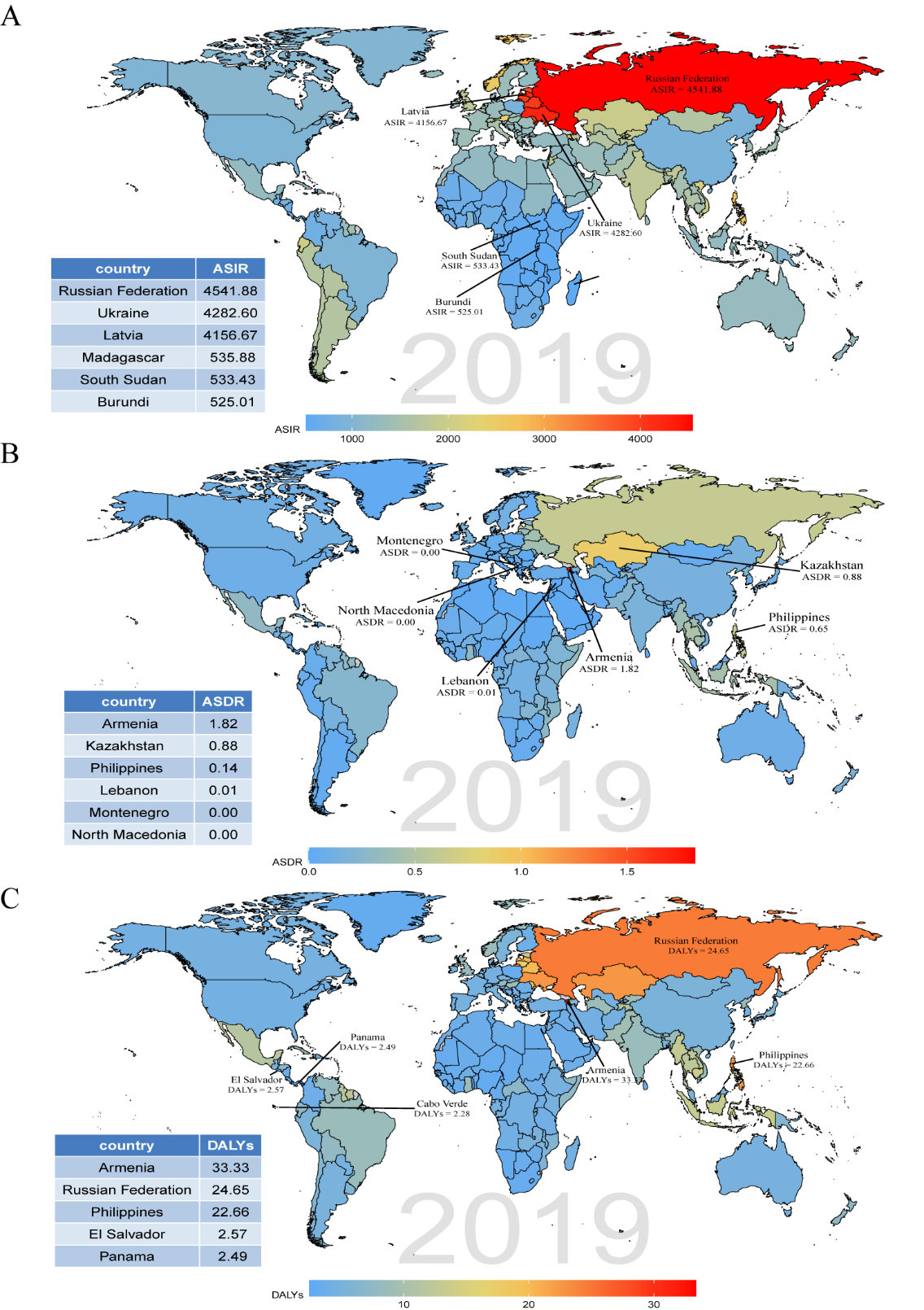


Figure 6. The global disease burden of urolithiasis for both sexes in 192 countries. A: The ASIR of urolithiasis in 2019; B. The ASDR of urolithiasis in 2019; C. The age-standardized DALY rate of urolithiasis in 2019; ASIR, age-standardized incidence rate; ASDR, age-standardized death rate.
