# Supplementary material for: Epidemiological Trends of Urolithiasis at the Global, Regional, and National Levels: A Population-Based Study
Source: Int J Clin Pract. 2022 Mar 30;2022:6807203. doi: 10.1155/2022/6807203 (PMC9159214; doi:10.1155/2022/6807203)
Supplement: Supplementary Materials — The supplementary material file 1: the supplementary tables and figures are already cited in our manuscript “Epidemiological trends of urolithiasis at the global, regional, and national levels: a population-based study.” The supplementary material file 2: figures are already cited in the manuscript “Epidemiological trends of urolithiasis at the global, regional, and national levels: a population-based study.” [file 6807203.f1.zip › 6807203.f1/Table1.doc]

| Table 1. The incident cases and ASIR in 1990 and 2019 and its current trends from 1990 to 2019 | | | | | |
| --- | --- | --- | --- | --- | --- |
|  | 1990 | | 2019 | | 1990-2019 |
|  | Incident cases No. *102 (95% UI) | ASIR per 100,000 No. (95% UI) | Incident cases No. *102 (95% UI) | ASIR per 100,000 No. (95% UI) | EAPC No. (95% CI) |
| Overall | 777757.57 [622391.16-951267.51] | 1696.18 [1358.11-2078.11] | 1155521.4 [930451.3-1401804.02] | 1394.03 [1126.4-1688.16] | -0.83 [-0.92 to -0.74] |
| Sex |  |  |  |  |  |
| Female | 249942.36 [199717.79-305779.86] | 1066.85 [851.17-1305.09] | 394466.96 [316443.64-479042.56] | 947.22 [761.21-1148.43] | -0.83 [-0.92 to -0.74] |
| Male | 527815.22 [421541.28-644728.87] | 2353.15 [1878.96-2879.17] | 761054.44 [610284.55-921683.98] | 1856.87 [1495.27-2245.34] | -0.83 [-0.92 to -0.74] |
| Socio-demographic index |  |  |  |  |  |
| High SDI | 145954.54 [115092.47-180095.64] | 1556.68 [1228.01-1924.36] | 175249.86 [143148.65-211860.66] | 1288.65 [1053.86-1544.09] | -0.47 [-0.56 to -0.38] |
| High-middle SDI | 261665.25 [209262.28-320011.49] | 2273.31 [1819.79-2776.75] | 295371.67 [234109.15-360701.08] | 1582.15 [1273.68-1924.87] | -1.52 [-1.66 to -1.39] |
| Middle SDI | 211170.93 [167561.15-259150.52] | 1582.66 [1255.22-1938.68] | 356833.63 [286464.56-435292.92] | 1319.12 [1062.99-1600.96] | -0.96 [-1.09 to -0.84] |
| Low-middle SDI | 124476.95 [99936.92-152749.55] | 1485.55 [1193.43-1813.78] | 244903.47 [195173.97-300902.16] | 1473.13 [1171.09-1804.8] | -0.12 [-0.18 to -0.05] |
| Low SDI | 34193.36 [26981.21-42070.53] | 954.92 [755.16-1176.74] | 82575.81 [64952.83-102069.74] | 1030.45 [809.77-1273.81] | 0.33 [0.24 to 0.43] |
| Region |  |  |  |  |  |
| Andean Latin America | 4592.35 [3666.61-5711.27] | 1609.5 [1290.27-1977.8] | 11073.03 [9169.33-13239.26] | 1772.43 [1472.6-2110.69] | 0.52 [0.44 to 0.6] |
| Australasia | 3140.31 [2465.53-3872.12] | 1405.3 [1096.4-1739.12] | 4769.7 [3737.57-5898.03] | 1283.37 [1004.68-1573.74] | -0.35 [-0.44 to -0.26] |
| Caribbean | 3141.27 [2478.56-3868.37] | 1056.51 [830.22-1310.52] | 6319.37 [4959.54-7881.91] | 1239.73 [979.28-1540.36] | 0.66 [0.63 to 0.7] |
| Central Asia | 10146.28 [8051.77-12407.22] | 1755.54 [1403.55-2151.02] | 16552.56 [13150.52-20320.44] | 1787.98 [1435.54-2174.91] | 0.05 [0.02 to 0.08] |
| Central Europe | 23202.84 [18302.32-28555.11] | 1657.22 [1324.62-2032.82] | 17735.73 [14611.9-21439.47] | 1178.91 [977.08-1400.97] | -0.71 [-1 to -0.42] |
| Central Latin America | 11169.98 [8874.72-13714.68] | 974.87 [774.06-1202.2] | 25837.65 [20622.08-31272.76] | 1012.43 [810.44-1222.61] | 0.13 [-0.21 to 0.48] |
| Central Sub-Saharan Africa | 1990.39 [1562.96-2465.6] | 533.21 [417.75-663.53] | 5315.77 [4130.91-6572.04] | 575.37 [446.63-711.22] | 0.31 [0.24 to 0.37] |
| East Asia | 168619.46 [132241.51-208877.18] | 1592.83 [1245.33-1984.66] | 185313.78 [147859.26-226692.4] | 901.81 [727.27-1088.77] | -2.68 [-2.96 to -2.4] |
| Eastern Europe | 138768.69 [112392.18-168155.88] | 5143.77 [4155.8-6201.33] | 127338.57 [102014.28-156010.91] | 4433.72 [3542.49-5414.66] | -0.69 [-0.85 to -0.53] |
| Eastern Sub-Saharan Africa | 6479.93 [5146.44-7948.33] | 548.62 [431.96-674.35] | 15432.53 [12133.22-19039.5] | 565.68 [444.29-692.31] | 0.15 [0.09 to 0.2] |
| High-income Asia Pacific | 31236.93 [23822.16-39297.37] | 1536.37 [1181.32-1920.86] | 39470.15 [31610.91-48358.91] | 1475.15 [1172.93-1795.88] | -0.27 [-0.35 to -0.18] |
| High-income North America | 50604.28 [39648.82-62952.81] | 1621.04 [1270.22-2010.69] | 47402.27 [40694.01-55617.51] | 982.95 [843.8-1137.38] | -2.02 [-2.34 to -1.69] |
| North Africa and Middle East | 29136.66 [22817.49-36153.48] | 1159.44 [904.44-1445.58] | 74482.11 [58347.67-93542.37] | 1250.71 [985.87-1553.28] | 0.29 [0.26 to 0.32] |
| Oceania | 441.17 [343.55-553.77] | 978.39 [758.56-1220.19] | 1096.74 [847.74-1389.26] | 1033.1 [799.54-1296.18] | 0.14 [0.09 to 0.19] |
| South Asia | 130374.21 [102728.35-162126] | 1518.02 [1206.38-1880.74] | 307303.25 [241418.47-382421.15] | 1757.72 [1382.75-2184.63] | 0.64 [0.54 to 0.74] |
| Southeast Asia | 63038.83 [50129.6-76385.12] | 1904.34 [1511.83-2313.01] | 118032.07 [95434.17-142760.02] | 1652.63 [1348.2-1979.06] | -0.82 [-0.95 to -0.69] |
| Southern Latin America | 7819.94 [6065.58-9817.15] | 1646.58 [1275.16-2070.5] | 12394.17 [9577.27-15596.51] | 1674.47 [1295.15-2119.25] | -0.21 [-0.28 to -0.14] |
| Southern Sub-Saharan Africa | 2887.71 [2272.83-3570.66] | 701.37 [552.78-863.37] | 5461.43 [4302.99-6791.1] | 725.49 [574.19-893.26] | 0.11 [0.07 to 0.15] |
| Tropical Latin America | 12924 [10385.26-15774.68] | 1034.66 [833.81-1253.92] | 24369.28 [19765.47-29260.19] | 969.93 [789.43-1165.79] | -0.36 [-0.45 to -0.28] |
| Western Europe | 69354.2 [54788.18-85768.48] | 1490.85 [1183-1846.74] | 88055.46 [70368.82-108735.24] | 1490.21 [1181.37-1829.24] | 0.53 [0.38 to 0.69] |
| Western Sub-Saharan Africa | 8688.15 [6911.46-10654.41] | 688.96 [543.93-847.52] | 21765.77 [17278.95-26674.36] | 735.82 [579.31-902.54] | 0.28 [0.22 to 0.34] |
| ASIR: age-standardized incidence rate; EAPC: estimated annual percentage change; CI: confdence interval; UI: Uncertainty interval. | | | | | |
