# Supplementary material for: Epidemiological Trends of Urolithiasis at the Global, Regional, and National Levels: A Population-Based Study
Source: Int J Clin Pract. 2022 Mar 30;2022:6807203. doi: 10.1155/2022/6807203 (PMC9159214; doi:10.1155/2022/6807203)
Supplement: Supplementary Materials — The supplementary material file 1: the supplementary tables and figures are already cited in our manuscript “Epidemiological trends of urolithiasis at the global, regional, and national levels: a population-based study.” The supplementary material file 2: figures are already cited in the manuscript “Epidemiological trends of urolithiasis at the global, regional, and national levels: a population-based study.” [file 6807203.f1.zip › 6807203.f1/Table2.doc]

| Table 2. The death cases and ASDR in 1990 and 2019 and its up-to-date trends from 1990 to 2019 | | | | | |
| --- | --- | --- | --- | --- | --- |
|  | 1990 | | 2019 | | 1990-2019 |
|  | Deaths cases No. *10^2^ (95% UI) | ASDR per 100,000 No.(95% UI) | Deaths cases No. *10^2^(95% UI) | ASDR per 100,000 No.(95% UI) | EAPC No. (95% CI) |
| Overall | 113.38 [72.78-137.77] | 0.3 [0.2-0.37] | 132.79 [106.16-162.67] | 0.17 [0.14-0.21] | -2.05 [-2.25 to -1.85] |
| Sex |  |  |  |  |  |
| Female | 51.84 [35.12-61.16] | 0.25 [0.17-0.29] | 63.48 [51.75-82.92] | 0.15 [0.12-0.19] | -2.05 [-2.25 to -1.85] |
| Male | 61.54 [30.77-80.34] | 0.38 [0.2-0.5] | 69.31 [44.97-91.42] | 0.2 [0.13-0.26] | -2.05 [-2.25 to -1.85] |
| Socio-demographic index |  |  |  |  |  |
| High SDI | 13.33 [11.03-16.31] | 0.13 [0.1-0.16] | 22.41 [17.67-30.28] | 0.1 [0.08-0.14] | -0.09 [-0.53 to 0.34] |
| High-middle SDI | 37.9 [29.85-43.71] | 0.37 [0.29-0.43] | 35.05 [30.24-43.39] | 0.18 [0.15-0.22] | -2.76 [-3.08 to -2.44] |
| Middle SDI | 34.58 [16.81-44.28] | 0.37 [0.18-0.48] | 41.14 [28.21-52.7] | 0.18 [0.13-0.24] | -2.61 [-2.74 to -2.47] |
| Low-middle SDI | 22.93 [8.3-33.21] | 0.41 [0.15-0.62] | 27.55 [13.96-36.62] | 0.22 [0.11-0.29] | -2.38 [-2.52 to -2.24] |
| Low SDI | 4.59 [2.68-6.9] | 0.22 [0.12-0.36] | 6.57 [4.16-9.61] | 0.15 [0.1-0.23] | -1.35 [-1.4 to -1.29] |
| Region |  |  |  |  |  |
| Andean Latin America | 0.15 [0.07-0.21] | 0.07 [0.04-0.1] | 0.36 [0.17-0.5] | 0.06 [0.03-0.09] | 0.16 [-0.05 to 0.38] |
| Australasia | 0.47 [0.37-0.56] | 0.2 [0.16-0.24] | 0.55 [0.44-0.74] | 0.1 [0.08-0.14] | -2.15 [-2.74 to -1.56] |
| Caribbean | 0.35 [0.27-0.44] | 0.13 [0.1-0.17] | 0.92 [0.68-1.23] | 0.18 [0.13-0.24] | 1.6 [1.43 to 1.77] |
| Central Asia | 1.2 [0.84-1.85] | 0.27 [0.19-0.42] | 2.43 [1.87-3.56] | 0.44 [0.32-0.69] | 1.74 [1.38 to 2.11] |
| Central Europe | 5.94 [5.14-8.83] | 0.42 [0.36-0.62] | 1.44 [1.14-1.85] | 0.07 [0.05-0.08] | -6.31 [-7.33 to -5.27] |
| Central Latin America | 1.85 [1.49-2.17] | 0.22 [0.17-0.26] | 4.91 [3.94-6.88] | 0.21 [0.17-0.29] | -0.14 [-0.38 to 0.09] |
| Central Sub-Saharan Africa | 0.31 [0.15-0.54] | 0.14 [0.06-0.23] | 0.56 [0.29-0.95] | 0.11 [0.05-0.19] | -0.78 [-0.92 to -0.63] |
| East Asia | 40.73 [18.5-51.36] | 0.55 [0.25-0.69] | 26.96 [18.32-36.57] | 0.15 [0.1-0.2] | -4.93 [-5.23 to -4.63] |
| Eastern Europe | 17.99 [15.39-23.08] | 0.65 [0.56-0.83] | 18.93 [15.49-22.84] | 0.55 [0.45-0.66] | -0.84 [-1.3 to -0.37] |
| Eastern Sub-Saharan Africa | 1.4 [0.82-2.44] | 0.23 [0.13-0.37] | 2.3 [1.32-4.12] | 0.2 [0.12-0.34] | -0.54 [-0.58 to -0.5] |
| High-income Asia Pacific | 1.23 [0.92-1.52] | 0.07 [0.05-0.09] | 7.05 [5.04-9.64] | 0.11 [0.09-0.15] | 2.6 [2.32 to 2.88] |
| High-income North America | 3.35 [2.65-4.14] | 0.09 [0.07-0.11] | 6.24 [5.12-8.49] | 0.09 [0.08-0.13] | 0.8 [0.43 to 1.18] |
| North Africa and Middle East | 0.48 [0.28-0.81] | 0.03 [0.01-0.06] | 1.16 [0.7-1.51] | 0.03 [0.02-0.04] | 1.06 [0.78 to 1.35] |
| Oceania | 0.05 [0.02-0.08] | 0.17 [0.06-0.28] | 0.09 [0.03-0.15] | 0.12 [0.05-0.2] | -1.22 [-1.32 to -1.12] |
| South Asia | 16.18 [7.39-25.81] | 0.33 [0.15-0.56] | 20.82 [12.24-30.82] | 0.16 [0.1-0.25] | -2.65 [-2.76 to -2.54] |
| Southeast Asia | 12.07 [3.2-17.8] | 0.5 [0.15-0.74] | 21.85 [6.95-29.47] | 0.4 [0.13-0.55] | -0.88 [-1.01 to -0.74] |
| Southern Latin America | 0.12 [0.1-0.16] | 0.03 [0.02-0.04] | 0.34 [0.26-0.44] | 0.04 [0.03-0.05] | 1.13 [0.72 to 1.54] |
| Southern Sub-Saharan Africa | 0.17 [0.11-0.23] | 0.05 [0.04-0.07] | 0.29 [0.2-0.39] | 0.05 [0.04-0.07] | -0.17 [-0.86 to 0.52] |
| Tropical Latin America | 0.88 [0.67-1.06] | 0.1 [0.07-0.12] | 5.52 [4.33-9.31] | 0.23 [0.18-0.39] | 4 [3.75 to 4.25] |
| Western Europe | 7.68 [5.71-8.68] | 0.13 [0.1-0.15] | 8.84 [7.29-12.37] | 0.08 [0.07-0.12] | -1.11 [-1.57 to -0.66] |
| Western Sub-Saharan Africa | 0.79 [0.3-1.32] | 0.11 [0.04-0.19] | 1.24 [0.53-1.75] | 0.08 [0.03-0.11] | -1.29 [-1.39 to -1.2] |
| ASDR: age-standardized death rate; EAPC: estimated annual percentage change; CI: confdence interval; UI: Uncertainty interval. | | | | | |
