# Supplementary material for: Epidemiological Trends of Urolithiasis at the Global, Regional, and National Levels: A Population-Based Study
Source: Int J Clin Pract. 2022 Mar 30;2022:6807203. doi: 10.1155/2022/6807203 (PMC9159214; doi:10.1155/2022/6807203)
Supplement: Supplementary Materials — The supplementary material file 1: the supplementary tables and figures are already cited in our manuscript “Epidemiological trends of urolithiasis at the global, regional, and national levels: a population-based study.” The supplementary material file 2: figures are already cited in the manuscript “Epidemiological trends of urolithiasis at the global, regional, and national levels: a population-based study.” [file 6807203.f1.zip › 6807203.f1/Table3.doc]

| Table 3. The DALYs and age-standardized DALYs rate in 1990 and 2019 and its up-to-date trends from 1990 to 2019 | | | | | |
| --- | --- | --- | --- | --- | --- |
|  | 1990 | | 2019 | | 1990-2019 |
|  | DALY No. *10^3^ (95% UI) | Age-standardized DALY Rate per 100,000 No. (95% UI) | DALY No. *10^3^ (95% UI) | Age-standardized DALY Rate per 100,000 No. (95% UI) | EAPC No. (95% CI) |
| Overall | 5167.31 [3741.33-6357.17] | 11.75 [8.57-14.39] | 6043.09 [4773.54-7451.94] | 7.35 [5.82-9.04] | -1.77 [-1.92 to -1.63] |
| Sex |  |  |  |  |  |
| Female | 2081.58 [1524.74-2490.71] | 9.11 [6.73-10.91] | 2420.08 [1939.13-2990.58] | 5.72 [4.58-7.07] | -1.77 [-1.92 to -1.63] |
| Male | 3085.73 [2071.94-3928.78] | 14.76 [9.92-18.82] | 3623.01 [2744.39-4545.58] | 9.1 [6.92-11.34] | -1.77 [-1.92 to -1.63] |
| Socio-demographic index |  |  |  |  |  |
| High SDI | 646.46 [496.31-818.12] | 6.68 [5.07-8.53] | 802.64 [626.22-1005.97] | 5.3 [3.99-6.75] | -0.5 [-0.69 to -0.31] |
| High-middle SDI | 1639.97 [1331.4-1970.73] | 14.68 [11.99-17.61] | 1525.21 [1218.89-1871.38] | 7.94 [6.33-9.79] | -2.41 [-2.62 to -2.21] |
| Middle SDI | 1587.67 [1017.33-1981.05] | 12.91 [8.23-16.13] | 1943.09 [1505-2400.21] | 7.49 [5.81-9.17] | -2.12 [-2.26 to -1.98] |
| Low-middle SDI | 1047.82 [568.37-1355.54] | 13.86 [7.44-18.79] | 1359.64 [975.4-1738.97] | 8.85 [6.24-11.25] | -1.74 [-1.83 to -1.64] |
| Low SDI | 243.48 [169.25-332.3] | 7.6 [5.21-10.43] | 409.03 [295.81-547.9] | 5.88 [4.33-7.87] | -0.96 [-1.01 to -0.9] |
| Region |  |  |  |  |  |
| Andean Latin America | 17.25 [11.89-23.21] | 6.32 [4.46-8.49] | 39.33 [28.3-52.39] | 6.4 [4.62-8.44] | 0.28 [0.19 to 0.37] |
| Australasia | 17.77 [14.28-21.92] | 7.78 [6.25-9.65] | 21.81 [16.67-28.01] | 5.37 [4.04-6.95] | -1.13 [-1.42 to -0.83] |
| Caribbean | 19.29 [14.76-24.78] | 6.61 [5.13-8.3] | 41.58 [32.06-53.23] | 8.14 [6.26-10.45] | 1.08 [0.98 to 1.18] |
| Central Asia | 57.19 [42.34-76.35] | 10.83 [8.05-14.57] | 95.98 [75.68-120.04] | 12.42 [9.88-15.89] | 0.29 [0.12 to 0.47] |
| Central Europe | 193.89 [159.39-263.24] | 13.51 [11.06-18.26] | 74.32 [56.83-96.06] | 4.52 [3.4-5.95] | -3.18 [-3.6 to -2.76] |
| Central Latin America | 88.28 [74.03-105.22] | 8.21 [6.86-9.78] | 205.88 [167.4-263.13] | 8.24 [6.7-10.57] | 0.06 [-0.2 to 0.32] |
| Central Sub-Saharan Africa | 16.32 [10.42-26.29] | 5.02 [3.11-7.83] | 32.89 [21.94-47.33] | 4.33 [2.77-6.34] | -0.54 [-0.64 to -0.45] |
| East Asia | 1621.28 [961.22-2002.29] | 16.61 [9.81-20.36] | 1075.6 [841.94-1343.94] | 5.35 [4.17-6.69] | -4.43 [-4.69 to -4.16] |
| Eastern Europe | 809.3 [661.25-979.1] | 29.44 [24.01-35.53] | 729.69 [586.18-893.37] | 23.61 [18.69-29.23] | -1.09 [-1.36 to -0.82] |
| Eastern Sub-Saharan Africa | 67.41 [44.29-111.06] | 6.37 [4.24-10.05] | 106.78 [69.37-162.09] | 4.83 [3.18-7.5] | -1.09 [-1.16 to -1.03] |
| High-income Asia Pacific | 109.59 [77.09-148.23] | 5.48 [3.91-7.35] | 188.89 [145.15-237.34] | 5.7 [4.18-7.45] | 0.25 [0.16 to 0.34] |
| High-income North America | 197.48 [147.12-256.27] | 6.16 [4.55-8.08] | 234.06 [187.71-290.06] | 4.52 [3.59-5.63] | -1.01 [-1.29 to -0.72] |
| North Africa and Middle East | 98.26 [67.05-135.2] | 3.95 [2.73-5.46] | 235.59 [159.04-328.15] | 4.1 [2.82-5.65] | 0.2 [0.16 to 0.25] |
| Oceania | 3.03 [1.69-4.27] | 7.31 [3.99-10.28] | 6.2 [3.82-8.72] | 6.25 [3.86-8.8] | -0.58 [-0.63 to -0.52] |
| South Asia | 842.78 [536.3-1134.41] | 11.47 [7.08-16.51] | 1355.04 [979.19-1806.73] | 8.33 [6-11.1] | -1.2 [-1.31 to -1.1] |
| Southeast Asia | 530.34 [243.98-712.9] | 17.17 [7.93-23.59] | 857.45 [460.68-1090.19] | 13.06 [6.92-16.54] | -1.15 [-1.25 to -1.06] |
| Southern Latin America | 24.78 [16.71-34.52] | 5.23 [3.53-7.3] | 41.49 [28.43-57.6] | 5.54 [3.78-7.74] | -0.07 [-0.17 to 0.02] |
| Southern Sub-Saharan Africa | 15.18 [11.11-19.23] | 3.71 [2.85-4.68] | 25.02 [18.71-32.63] | 3.46 [2.63-4.44] | -0.22 [-0.6 to 0.15] |
| Tropical Latin America | 64.63 [51.08-80.92] | 5.43 [4.34-6.76] | 211.87 [169.2-298.86] | 8.55 [6.84-12.13] | 2.07 [1.93 to 2.2] |
| Western Europe | 326.16 [254-411.6] | 6.55 [5.04-8.41] | 368.39 [275.06-474.37] | 5.55 [3.99-7.28] | -0.03 [-0.21 to 0.14] |
| Western Sub-Saharan Africa | 47.11 [29.76-65.05] | 4.12 [2.55-5.79] | 95.22 [65.48-126.5] | 3.61 [2.42-4.79] | -0.45 [-0.49 to -0.42] |
| DALYs: disability adjusted life-years; EAPC: estimated annual percentage change; CI: confdence interval; UI: Uncertainty interval. | | | | | |
